# Evaluation of Methane Sources in Groundwater in Northeastern Pennsylvania

**DOI:** 10.1111/gwat.12056

**Published:** 2013-04-05

**Authors:** Lisa J Molofsky, John A Connor, Albert S Wylie, Tom Wagner, Shahla K Farhat

**Affiliations:** 2 GSI Environmental Inc.Houston, TX 77373; 3 Cabot Oil and Gas CorporationPittsburgh, PA 15276

## Abstract

Testing of 1701 water wells in northeastern Pennsylvania shows that methane is ubiquitous in groundwater, with higher concentrations observed in valleys vs. upland areas and in association with calcium-sodium-bicarbonate, sodium-bicarbonate, and sodium-chloride rich waters—indicating that, on a regional scale, methane concentrations are best correlated to topographic and hydrogeologic features, rather than shale-gas extraction. In addition, our assessment of isotopic and molecular analyses of hydrocarbon gases in the Dimock Township suggest that gases present in local water wells are most consistent with Middle and Upper Devonian gases sampled in the annular spaces of local gas wells, as opposed to Marcellus Production gas. Combined, these findings suggest that the methane concentrations in Susquehanna County water wells can be explained without the migration of Marcellus shale gas through fractures, an observation that has important implications for understanding the nature of risks associated with shale-gas extraction.

## Introduction

Significant media attention has been focused on the potential for methane impacts in drinking water wells located within areas of hydraulic fracturing activities for shale-gas development. Distinguishing among the various sources of methane gas that may affect drinking water wells requires proper assessment of background conditions. In this study, we review the results of background methane and groundwater quality surveys, in conjunction with geologic and historical information, to develop a better understanding of the potential sources of methane levels in drinking water wells in Susquehanna County in northeastern Pennsylvania.

Susquehanna County has experienced substantial gas extraction activities in the Marcellus shale since 2006. Prior to that time, there was not a significant history of oil and gas operations in this county, thereby providing a unique opportunity to evaluate the potential effects of shale-gas extraction on groundwater resources in the Appalachian basin. Other researchers have suggested that elevated methane concentrations in water wells in Susquehanna County are the result of regional impacts from shale-gas extraction activities (e.g., Osborn et al. 2011). To test this hypothesis, we have evaluated data from the sampling and testing of 1701 water wells throughout Susquehanna County to assess the prevalence and distribution of methane concentrations in groundwater. We have also evaluated isotopic and molecular analyses of hydrocarbon gases in the Dimock Township of Susquehanna County, an area of focused sampling by the Pennsylvania Department of Environmental Protection (DEP) and the U.S. Environmental Protection Agency, to determine whether reported methane concentrations above the Pennsylvania DEP action level (7000 µg/L) in local water wells exhibit signatures consistent with Marcellus production gases, or overlying Middle and Upper Devonian gases sampled in annular spaces of local gas wells.

Our research indicates that shale-gas extraction has not resulted in regional impacts on groundwater quality in Susquehanna County, a finding which suggests that hydraulic fracturing is not responsible for the creation or enhancement of wide-spread pathways by which Marcellus shale gas can rapidly travel to the surface.

## Methods

Our study focused on characterizing the geologic and hydrogeologic context of methane occurrence in water wells in northeastern Pennsylvania. For this purpose, we have collected and reviewed the following types of information: (1) geologic data on regional structure and stratigraphy, (2) studies on aquifer dynamics and geochemical characteristics, and (3) historical documentation regarding the occurrence of hydrocarbon gases in the shallow and deep subsurface. Within this context, we have evaluated data collected from 1701 water wells in Susquehanna County to determine the prevalence and distribution of elevated methane concentrations and other groundwater parameters. We have also assessed isotopic and molecular analyses of gases from 15 water wells sampled as part of an ongoing stray gas investigation in the Dimock Township to determine the origin of methane concentrations above the Pennsylvania DEP action level.

**Figure 1:**
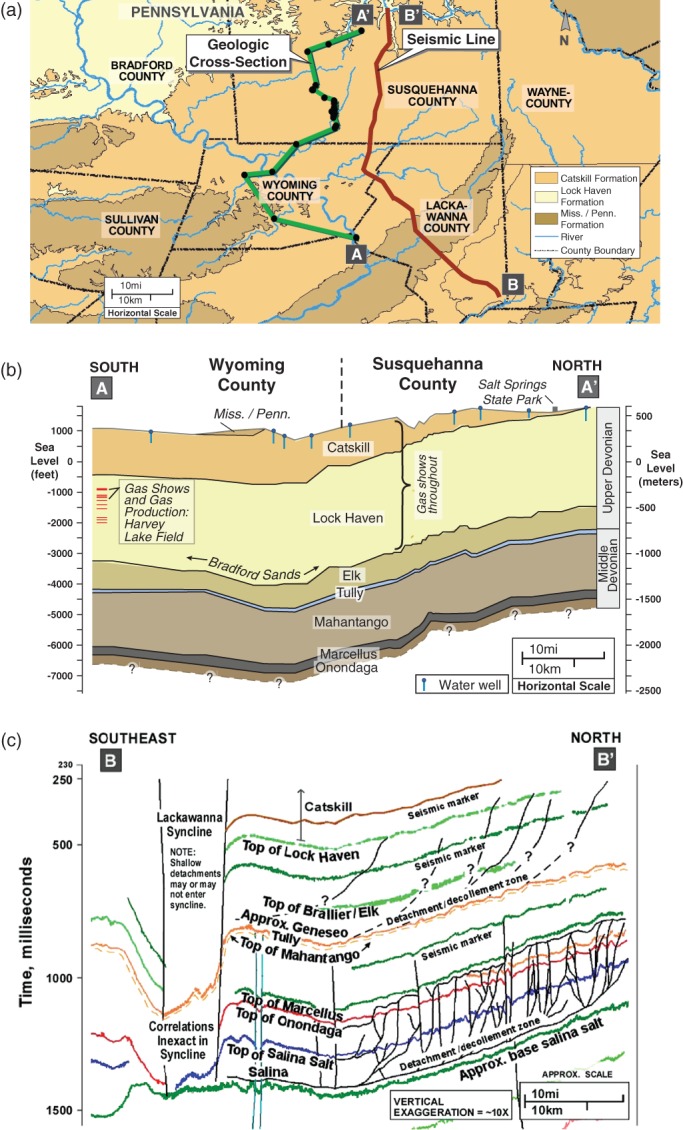
(a) Geographic map showing transect of wells in the cross section and seismic line. (b) Generalized cross section of Upper and Middle Devonian formations in Susquehanna and Wyoming County. (c) North-south 2D Seismic Line through Susquehanna, Wyoming, and Lackawanna County (Evans Geophysical 2007).

Geologic information discussed in this article was compiled from prior studies on the stratigraphy and structure of the northeastern portion of the Appalachian basin, as well as new data acquired during exploration activities associated with recent Marcellus shale gas development. Specifically, stratigraphic information recorded during the drilling of 24 shale-gas wells located in Susquehanna and Wyoming counties facilitated the development of the geologic cross-section presented in Figure 1b. The seismic line shown in Figure 1c is based on interpretation of 57 miles of 2D seismic data acquired by Evans Geophysical (2007). Historical notations regarding the presence of shallow gas shows, historical gas fields, and bubbling springs and/or water wells in northeastern Pennsylvania were gathered from a detailed review of over a dozen documents dating back to the early 1800s.

The 1701 water samples analyzed as part of an extensive predrill water well survey from 2008 through 2011 were collected in the field and analyzed in NELAC accredited laboratories in accordance with procedures detailed in the supporting information. Statistical analyses of predrill data were conducted using Statistical Software ProUCL 4.1.01, which was developed and provided to the public by the United States Environmental Protection Agency (US EPA). Molecular and isotopic analyses of gases in shale-gas wells and local water wells in the Dimock Township were collected as part of an ongoing stray gas investigation by the Pennsylvania DEP and Cabot Oil and Gas Corporation in 2009 through 2011 using procedures detailed in the supporting information. This article also reports molecular and isotopic data from dissolved gases in Susquehanna County water wells collected by Osborn et al. (2011) and the US EPA (2012). Details of sample collection and analyses of these samples are presented in the respective studies, for which citations are provided.

## Regional Geologic and Hydrologic Conditions

### Deep and Shallow Stratigraphy

Knowledge of naturally occurring sources of hydrocarbon gases in both shallow and deep stratigraphic units provides critical information for evaluating potential sources and migration pathways of methane into local water wells. Natural gas in the subsurface can be both *microbial* in origin, that is, formed from the microbial breakdown of organic material, and *thermogenic* in origin, that is, formed from the abiotic degradation of organic material in formations under high temperatures and pressures at depth. Microbial methane commonly occurs in shallow bedrock and surficial deposits of alluvium (sands, silts, gravel, peat, and clay) and glacial drift (glacial till and outwash) (Coleman 1994). In Susquehanna County, these deposits can range from a few meters thick in elevated areas to tens of meters thick in lowlands (Aber 1974; Sevon et al. 1975; Inners and Fleeger 2002; Braun 2006). In areas with organic-rich shale formations, recent studies have also suggested an association between microbial methane concentrations and groundwater with longer residence times (in which progressive microbially mediated redox reactions can lead to methane production) (Kresse et al. 2012).

Surficial deposits in Susquehanna County are underlain by the Catskill and Lock Haven Formations, a series of interbedded sandstone, siltstone, and shale deposits formed during the Upper Devonian period (359 to 385 million years ago) (Figure 1b) (ICS 2010). The Catskill and Lock Haven Formations have historically been explored for reservoirs of thermogenic natural gas. These Upper Devonian deposits attained deepest burial depths at approximately 250 million years ago, at which time organic matter entrained in discrete lenses and distributed throughout the formations attained temperatures sufficient to crack to thermogenic gas (Evans 1995). Such elevated past temperatures are evidenced by the vitrinite reflectance of organic material (greater than 2.0%) observed in seams and lag deposits currently exposed in numerous outcrops and sandstone (bluestone) quarries in Susquehanna County (Laughrey et al. 2004; Weatherford Laboratories 2011).

The Bradford Sands, a series of thick sandstone deposits, form the base of the Lock Haven Formation, and are underlain by the Brallier Formation (locally known as the Elk Formation) and the Trimmers Rock Formation, which are comprised of interbedded sandstone, siltstone, and shale stratum of Upper Devonian age (Carter and Harper 2002). The Tully limestone is the youngest unit of the Middle Devonian age strata, which include the Mahantango Formation, consisting of laminated siltstone, sandstone and shales, and the organic-rich Marcellus shale, estimated to contain as much as 127 trillion cubic feet of thermogenic gas (Engelder 2009). The volume of gas contained in the Marcellus shale represents over 5 million times the volume of natural gas consumed in the United States in 2011 (24.4 million cubic feet; EIA 2012).

### Fault and Fracture Systems

An extensive natural fracture network is present in both the Catskill and Lock Haven Formations, with penetrating north-south oriented vertical planar fractures bisected by multiple inferior fracture sets and bedding planes (Hollowell and Koester 1975; Geiser and Engelder 1983; Taylor 1984; Callaghan et al. 2010).

On a larger scale, seismic data provides valuable information regarding the structural setting and presence and/or absence of large-scale faults that could serve as conduits for the transport of fluids or gases. An interpretation of the stratigraphic and structural framework beneath Susquehanna and Lackawanna Counties from a portion of a regional 2D seismic line that transects the area is depicted in Figure 1c (Evans Geophysical 2007). The Upper Devonian section from the Catskill through the base of the Elk Sands shows shallow south-dipping stratigraphy interrupted by low-angle thrust faults that dip from 25° to 40° to the south. These thrust faults flatten and become bed parallel above the Tully limestone. In contrast, the Middle Devonian Marcellus to Silurian Salina Formations are interpreted to be bisected by frequent steep dipping (60° to 90°) reverse faults, back thrusts, and compressional and/or salt-related features that terminate near the top of the Marcellus or in the lower units of the Mahantango Formation in Susquehanna County (Davis and Engelder 1985; Scanlin and Engelder 2003). These faults rarely extend to the Tully stratigraphic level. This seismic section illustrates a disconnection between the distinct structural styles above and below the Tully horizon, interpreted to be a regional structural detachment that separates the shallow Catskill-Lock Haven stratigraphic section from the deeper Mahantango-Marcellus stratigraphic section. Given the presence of overpressured conditions in the Mahantango Formation and absence of such pressure above the Tully Limestone, this newly observed detachment zone, or the Tully Formation itself, is interpreted to act as a restrictive barrier to the present-day upward movement of deep formation fluids and methane from gas-charged units in the Middle Devonian Mahantango and Marcellus Formations in Susquehanna County.

### Aquifer Dynamics/Water Well Construction

The great majority of water wells in Susquehanna County penetrate the fractured Catskill Formation, in which groundwater flow in unweathered bedrock appears to be controlled by secondary permeability primarily through vertical to near vertical north-south oriented fractures (as indicated by water seepage and flow, iron staining, and mineral precipitation) (Hollowell and Koester 1975; Geyer and Wilhusen 1982; Geiser and Engelder 1983; Taylor 1984). Most of the bedrock water wells are unsealed open-hole completions, with casing terminating in the shallow bedrock in order to draw water from multiple horizons at typical depths of 100 to 500 feet beneath ground surface (Lohman 1937, 1939; Taylor 1984; PaGWIS 2011). Median yields of 1146 water wells in the Catskill Formation were reported to be 0.76 and 2.2 L/s (12 and 35 gal./min.) for domestic and nondomestic wells, respectively (Taylor 1984). Valleys and streams in Susquehanna County commonly follow a pattern coincident with the presentation of vertical crosscutting joint sets and lineaments, dense fractures, and fold trends (e.g., the orthogonal drainage patterns of Wyalusing Creek, a tributary of the Susquehanna River) (Taylor 1984; Engelder et al. 2009). Salt springs are also frequently located along linear trends associated with faulting or fracturing, suggesting the potential for connection to the deeper, and more brackish, Lock Haven Formation (Lohman 1937).

### Historical Evidence for Occurrence of Shallow Natural Gas

Historical documentation suggests that the presence of methane gases in the shallow subsurface has been observed for over 200 years in Susquehanna County, long before the expansion of shale-gas fracturing in this area in 2006. For example, there are several dozen instances of flammable effervescing springs and water wells dating back to the late 1700s (Blackman 1873; Lohman 1939; Soren 1963; Breen et al. 2007; Susquehanna County Historical Society 2010; Williams 2010; Table S7 for a full list of citations). In addition, water well drillers have frequently reported encountering gas during drilling, particularly in valleys and other low-lying areas (Bell Brothers Well Drilling, Creswell Drilling, Beavers Well Drilling, Karp & Sons Drilling, JIMCON Drilling, Drake Drilling, personal communication, 2010).

Several gas fields in the past century have produced from formations less than 3000 feet below surface in northeastern Pennsylvania (e.g., Shrewsbury Gas Field, Lovelton Gas Field, Harveys Lake Gas Field), and there are numerous reports of gas shows between 80 and 800 feet during the drilling of oil and gas wells (Ashley and Robinson 1922; Hopbottom Well Record 1957; Soren 1961). For example, the 1881 publication “The Geology of Susquehanna and Wayne County” reported significant volumes of gas during the drilling of an oil boring in the Catskill Formation to a depth of 680 feet (White 1881). The presence of methane in natural springs and water wells has also been cited in adjacent New York State, where a survey of 239 water wells from 1999 to 2011 showed that 9% of water wells contained dissolved methane concentrations exceeding 10 mg/L (Kappel and Nystrom 2012). Due to potential gas contamination from natural sources, guidelines issued by the Pennsylvania DEP and other state agencies recommend the routine venting of water wells (PA DEP 2004).

## Results of Sampling and Testing Programs

In addition to researching the hydrogeology, geology, and history of Susquehanna County in an effort to better understand natural groundwater conditions, we have also collected and analyzed water samples from this area to assess the presence of methane, ethane, and other water quality parameters. The results of this analysis provide additional information with regards to the distribution of hydrocarbon gases, and their association with physical geography, gas production areas, and groundwater quality.

### Predrill Methane Data

This article evaluates data from 1701 water wells sampled in Susquehanna County to characterize baseline groundwater conditions during the period of 2008 to 2011. Over 900 of these samples were collected in accordance with Pennsylvania DEP regulations (Oil and Gas Act 13), which stipulates that an operator must conduct a “predrill or prealteration” water survey prior to the drilling of each gas well in order to maintain the right to contest any subsequent claims of groundwater impact. These samples were analyzed for both dissolved gas concentrations and general water quality parameters related to primary and secondary drinking water standards. Several hundred additional water well samples were collected from an 80-mile^2^ area within Brooklyn, Harford, and Gibson Townships (southeast Susquehanna County), which did not have significant gas development operations at the time. These samples were intended to establish baseline methane concentrations in groundwater in areas of Susquehanna County without substantial gas extraction activities and were primarily analyzed for concentrations of dissolved gases (methane, ethane, and propane).

Collectively, these 1701 samples are referred to as “predrill” because they were sampled prior to the drilling of specific *proposed* wells; however, several of the water wells sampled were located in close proximity to *previously* drilled gas wells. As a result, 322 of the water wells have been characterized as located in a “gas production area” (defined as an area within 1 km of gas wells drilled prior to the time of sampling), while 1379 of the water wells are considered to be located in a “nonproduction area” (i.e., with no gas well drilled prior to sampling within 1 km). The 1 km radius used here to define gas production areas is considered a conservative distance, consistent with the radius utilized by Osborn et al. (2011) in northeastern Pennsylvania and New York, and larger than the current predrill sampling radius (2500 feet = 0.76 km) stipulated by the Pennsylvania DEP. Combined, 78% of the 1701 water wells sampled in either gas production or nonproduction areas contained detectable concentrations of methane, with approximately 3.4% exceeding the 7000 µg/L level at which corrective action is recommended by the Pennsylvania DEP. (As a note, the saturation concentration for methane is approximately 28,000 µg/L at atmospheric pressure, though it can vary with groundwater temperature, pressure, and salinity.)

### Predrill Methane Data: Valleys vs. Uplands

**Figure 2:**
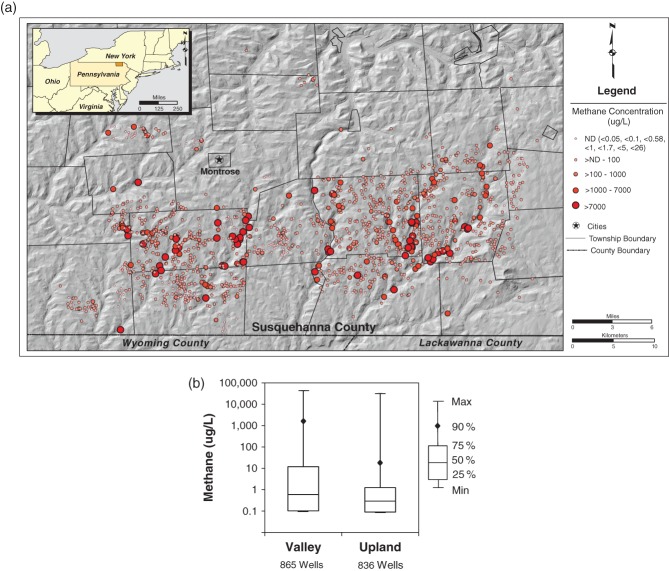
(a) LiDAR bare-earth elevation map showing dissolved methane concentrations from 1701 “predrill” water wells sampled in Susquehanna County. (b) Population distribution of methane concentrations in “predrill” water samples from valley and upland water wells in Susquehanna County.

To evaluate the possibility of a relationship to topography, dissolved methane concentrations measured in water wells were plotted on a light detection and ranging (LiDAR) bare-earth elevation map overlain with the National Hydrography Dataset (NHD). As indicated in Figure 2a, the distribution of methane concentrations appears to be correlated to surface topography, with the higher dissolved methane concentrations located in topographic lows (valleys).

To support statistical analysis of this observed correlation, the water well population was subdivided into wells located in valleys (defined as the area within 1000 feet of a major NHD flowline or 500 feet of minor tributaries to NHD flowlines) vs. wells located in uplands (greater than 1000 feet or 500 feet from a major or minor drainage, respectively). As shown in Figure 2b, the median concentrations of the two datasets differ moderately (0.67 vs. 0.34 µg/L of methane in lowland vs. upland water wells, respectively). However, the 90th percentile concentrations differ much more significantly (1800 vs. 17 µg/L of methane in lowland vs. upland water wells, respectively), indicating that upper range methane concentrations occur considerably more frequently in valley wells. Furthermore, although valley wells represent only 51% of the total water well population, they comprise 88% of the water wells with methane concentrations that exceed 7000 µg/L (the current Pennsylvania DEP action level). A one-way Mann-Whitney U Test supports a statistically significant difference between methane levels in lowland (865 locations) vs. upland water wells (836 locations), where valley water wells contain statistically elevated methane concentrations (p-value < 0.001).

### No Regional Association of Methane with Gas Production

In order to evaluate whether elevated methane concentrations in the data set exhibited a relationship to gas development activities, methane concentrations in water wells located in gas production areas were compared to those located in nonproduction areas. Of the water wells in gas production areas (322 wells), approximately 3.7% contained methane concentrations that exceeded 7000 µg/L, while 3.3% of water wells in nonproduction areas (1379 wells) contained methane concentrations above 7000 µg/L. This slight difference may be attributable to the fact that gas production areas contain a greater percentage of valley water wells (61% of 322 water wells) than nonproduction areas (49% of 1379 water wells). To evaluate whether the prevalence of valley water wells in gas-production areas was a factor, the methane concentrations of valley water wells in gas production areas were exclusively compared to those of valley water wells in nonproduction areas. Using a one-way Mann-Whitney U-test, methane concentrations in valley gas-production valley areas were found to be less than or equal to those in valley nonproduction valley areas (*p*-value = 0.007). A similar comparison of methane concentrations in upland water wells showed that methane concentrations in upland gas production areas were approximately equal (i.e., no statistically significant difference was observed) to those in upland nonproduction areas (two-way Mann-Whitney U-test *p*-value = 0.154). Furthermore, as a whole, no statistically significant difference was observed between methane concentrations in water wells in gas production areas vs. water wells in nonproduction areas (regardless of topographic location) (two-way Mann-Whitney U-test *p*-value = 0.503).

## Origin of Methane Concentrations in Water Wells

Determining the microbial vs. thermogenic (or mixed) origin of methane concentrations in the predrill water well samples can narrow the list of potential sources. One conventional approach to assessing thermogenic vs. microbial origin relies upon the ratios of methane to ethane, where gases with ratios less than 100 have been characterized as thermogenic, while those with ratios greater than 1000 have been characterized as microbial (with ratios between 100 and 1000 typically characterized as an indeterminate origin or a mixture of thermogenic/microbial gases) (Bernard et al. 1976; Schoell 1980; Taylor et al. 2000).

However, comparison of methane to ethane ratios to available isotopic data in predrill water well samples in nearby Bradford County (located directly west of Susquehanna County) suggest that the conventional approach for characterizing thermogenic vs. microbial origin based on methane to ethane ratios may not apply in the geologic context of northeastern Pennsylvania. Specifically, Baldassare (2011) observed methane to ethane ratios ranging from 55 to 6900 in over a dozen water well samples from Bradford County that displayed traditionally thermogenic carbon and hydrogen isotopic signatures (i.e., δ^13^C > –50‰, δ^2^H > –250‰). This suggests that these water wells could contain a notable thermogenic component, despite their traditionally microbial methane to ethane ratio.

In Susquehanna County, 1540 (91%) of the 1701 predrill water well samples were analyzed for both methane and ethane concentrations. Exactly 217 of these samples contained *detected* methane and ethane, and of those samples, approximately 72% (156 samples) displayed ratios of methane to ethane greater than 1000 (Figure S2). Isotopic analyses were not performed on these predrill samples. However, several water wells in Susquehanna County were sampled as part of a stray gas investigation after nearby shale-gas extraction activities (section *Discussion* below of isotopic and molecular analyses in the Dimock Township). Samples from three of these water wells exhibited classically microbial (i.e., greater than 1000) methane to ethane ratios but traditionally thermogenic carbon and hydrogen isotopic signatures. Consequently, at this time, the thermogenic, microbial, mixed thermogenic/microbial, or microbially altered (i.e., oxidized) origin of the predrill dissolved gas samples from water wells cannot be determined.

### Predrill Data: Correlation Between Methane and Other Groundwater Quality Parameters

To assess potential differences in groundwater sources, predrill water well samples in Susquehanna County were classified with regard to the relative concentrations of major cations (calcium, magnesium, potassium, and sodium) and anions (bicarbonate, chloride, and sulfate). The cation/anion composition of the groundwater is a product of the aquifer matrix (e.g., sandstone vs. shale), groundwater residence and/or rock-water interaction times, and the prominence of redox processes (Kresse et al. 2012). Consequently, for groundwater containing elevated methane levels, the water type can prove useful to understanding the flow path and retention time of water within the subsurface (i.e., stratum(s) of origin).

**Figure 3:**
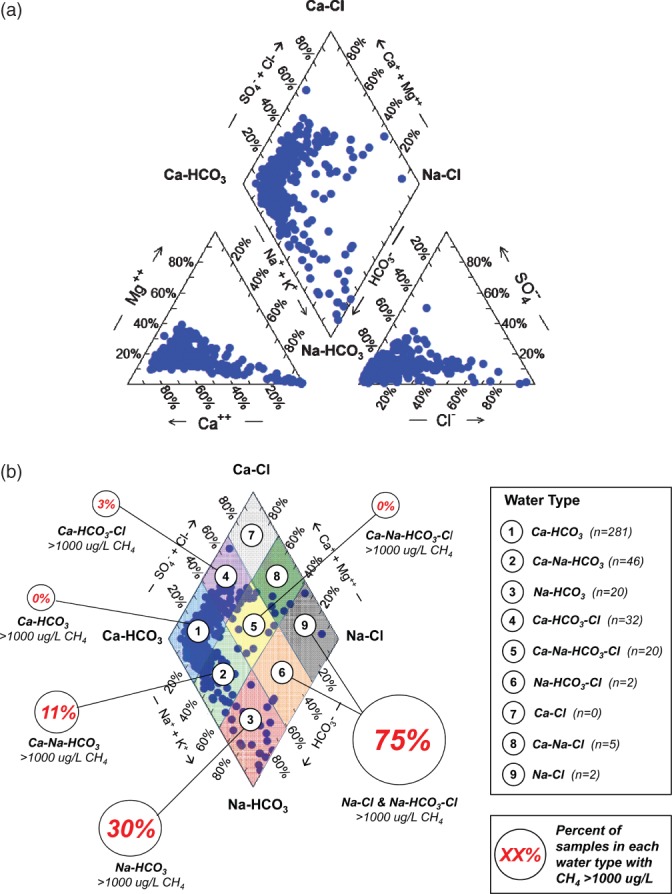
(a) Piper diagrams of major cation and anion composition of 408 predrill water samples. *Note: HCO_3_ concentrations estimated from reported alkalinity as CaCO_3_. (b) Percent of samples in each water type exceeding 1000 µg/L of methane. Water types determined based on those presented in Deutsch (1997).

The Piper diagrams shown in Figure 3a and 3b plot the results for the population of 408 predrill samples for which all of the major cations and anions were analyzed. As shown, each water sample is characterized with regards to water “type” based upon the relative milliequivalent concentrations of major ions. In this sample population, five predominant water types have been identified: Ca-HCO_3_ (281 samples), Ca-Na-HCO_3_ (46 samples), Na-HCO_3_ (20 samples), Ca-HCO_3_-Cl (32 samples), and Ca-Na-HCO_3_-Cl (20 samples). To characterize the relationship between water type and dissolved methane concentrations, the distribution of methane concentrations has been determined for each of the five principal water types. As shown in Figure 3b, the percentage of water samples exceeding 1000 µg/L of dissolved methane differs dramatically among water types, with 11 and 30% of the samples matching Ca-Na-HCO_3_ and Na-HCO_3_ water types, respectively, exceeding this level. By contrast, none of the 281 Ca-HCO_3_ water type samples exceed 1000 µg/L of methane. Only a limited number of groundwater samples matched the Na-HCO_3_-Cl and Na-Cl water types (four samples total); nevertheless, these samples exhibit several of the highest methane concentrations observed in the predrill dataset, with three of the four reported concentrations exceeding 10,000 µg/L.

Concentrations of individual groundwater quality parameters measured during predrill sampling were also evaluated to assess a possible correlation with dissolved methane concentrations. These data provide a comprehensive data set for the following parameters: alkalinity, barium, calcium, chloride, magnesium, manganese, pH, potassium, sodium, strontium, sulfate, and TDS. Data for an additional eight groundwater analytes (i.e., arsenic, aluminum, boron, bromide, iron, lead, nitrate, and sulfide) contained greater than 50% nondetect values and were therefore not amenable to this analysis (Figure S1).

To detect possible water quality trends related to methane, the concentration distribution for each parameter was determined for four ranges of methane concentrations: nondetect, detected to 1000 mg/L, greater than 1000 µg/L to 7000 µg/L, and greater than 7000 µg/L. The median concentrations of the following parameters were found to increase with increasing methane levels: barium, chloride, manganese, pH, sodium, strontium, and TDS (Figure S1). Median concentrations of alkalinity and potassium showed slight increases with increasing methane concentration, while median magnesium concentrations exhibited a minor decrease. Median sulfate and, to a lesser degree, calcium concentrations in groundwater were observed to inversely correlate with dissolved methane concentrations (i.e., lower sulfate at higher dissolved methane levels).

### Dimock Township: Stray Gas Investigation

Isotopic and molecular analyses of dissolved gases can provide a means to differentiate between: (1) microbial vs. thermogenic gas sources, (2) distinct sources of microbial gas, and (3) distinct sources of thermogenic gas (e.g., Fuex 1977; Schoell 1980; Clayton 1991; Coleman 1994; Wiese and Kvenvolden 1994; Baldassare and Laughrey 1997). In this study, we have evaluated isotopic and molecular data from 7 gas wells, and 15 water wells sampled by the Pennsylvania DEP and Cabot Oil and Gas Corporation in the Dimock Township of Susquehanna County as part of a site-specific stray-gas investigation in 2009 through 2011. In addition, we reviewed data from 9 water wells sampled by researchers from Duke University (Osborn et al. 2011) and 11 water wells sampled by the USEPA (USEPA 2012) in the same area from 2010 through 2012. One gas sample from a bubbling salt spring approximately 13 miles northeast of Dimock, in nearby Franklin Township, was also included in our evaluation. The goal of the evaluation presented here is to utilize available molecular and isotopic data to assess whether gases in Dimock area water wells were originating from the Marcellus Shale or overlying Middle and Upper Devonian deposits.

### Potential Source Gases: Isotopic and Molecular Analyses, 2009 to 2011

In 2009 and 2010, several water wells in the Dimock Township of Susquehanna County were reported to contain methane concentrations above the Pennsylvania DEP action level of 7000 µg/L (PA DEP 2009). To characterize potential proximate sources of gas migration, the Pennsylvania DEP conducted molecular and isotopic analyses of gas samples from the well cellar, the annular spaces surrounding casing strings, and the production pipeline and production casing of three nearby shale-gas extraction wells (PA DEP 2010a, 2010b).

Shale-gas wells are constructed of a series of steel casing strings (i.e., long sections of steel pipe) of various diameters that are installed concentrically within the wellbore, in a telescoping fashion. These casing strings serve the dual purpose of providing structural integrity of the wellbore and isolating surrounding formations (e.g., drinking water aquifers) from the underlying production zone. The space between the casing string and the wellbore is referred to as the “annular space,” which is commonly cemented in shallower casing strings, but may be left uncemented (i.e., “open”) in deeper sections, depending on site-specific conditions. The annular space of a gas well is in contact with surrounding formations, and can therefore contain gases from adjacent strata that may be present naturally above the targeted gas-producing shale formation.

In the three Dimock area gas wells, gas samples were collected from annular spaces surrounding the (1) shallow casing string adjoining potential freshwater aquifers, (2) the intermediate casing string, which penetrates to a depth several thousand feet beneath surface, and (3) the production casing string, which terminates within the Marcellus shale. Within these gas wells, the annular space surrounding the production casing string was cemented from the bottom of the wellbore to a depth above the Marcellus shale. The cementing of this portion of annular space is designed to prevent Marcellus formation gases and fluids from entering the overlying section of wellbore via an open annulus. Consequently, gases sampled from annular spaces surrounding the casing strings in Dimock Township gas wells were considered to represent a mixture of those originating in formations above the Marcellus Shale (i.e., Middle Devonian Mahantango and Upper Devonian Formations), which were penetrated in some sections by open annular spaces. One gas sample was also collected from the well cellar, a sunken pit that provides access to the gas wellhead which serves as a functional interface for the casing strings and the gas production line at the surface. This sample is considered to be a composite of gases found within the annular spaces of the casing strings.

The Pennsylvania DEP also collected Marcellus shale gas (two samples, one gas well) from inside the production casing and production pipeline of a local gas well. These samples are supplemented by Marcellus shale gas (four samples, four gas wells) collected by Cabot (a primary operator in the area) from within the production casing of additional gas wells in the Dimock Township. Collectively, these analyses represent 12 samples of potential source gases from 7 shale-gas wells located within the greater Dimock Township area (see Table S2 for a full data table).

As described previously, ratios of methane to ethane can be used as an initial tool to evaluate possible differences between hydrocarbon gas sources (Bernard et al. 1976; Schoell 1980). Gases originating from the production pipeline/production casing, and annular spaces/well cellar all exhibit methane to ethane ratios less than 60, which is consistent with the anticipated thermogenic origin of these samples (i.e., methane to ethane ratio < 100). However, methane to ethane ratios of the six samples from the production pipeline/production casing (Marcellus shale gas) are slightly lower (43 to 47) than those displayed by gases from annular spaces and well cellar, where all but one of the six samples contain methane to ethane ratios between 49 and 56.

The use of δ^13^C-CH_4_ values in combination with δ^2^H-CH_4_ values can provide another method to distinguish different thermogenic (and microbial) gases. Values of δ^13^C-CH_4_ and δ^2^H-CH_4_ represent the difference between the ^13^C/^12^C and ^2^H/^1^H isotopic ratios of a sample, and that of a recognized standard. More negative values of δ^13^C-CH_4_ and δ^2^H-CH_4_ are said to be isotopically “depleted” in the heavier isotopes (i.e., ^13^C and ^2^H), whereas more positive values are said to be isotopically “enriched.”

**Figure 4:**
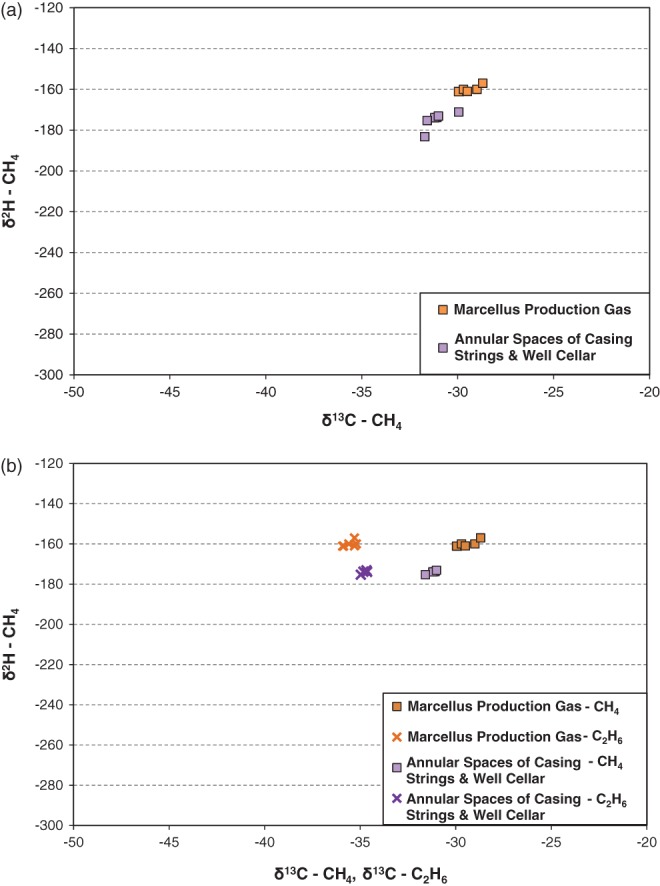
(a) Schoell plot of gases sampled by the Pennsylvania DEP and Cabot from production casing/production pipelines, the annular spaces of casing strings, and the well cellar of gas wells in the Dimock Township. (b) Plot of δ^13^C values of methane and ethane (*x*-axis) vs. δ^2^H value of methane (*y*-axis) in gases sampled by the Pennsylvania DEP and Cabot from production casing/production pipelines, the annular spaces of casing strings, and the well cellar of gas wells in the Dimock Township. *Only samples with reported values of both δ^13^C-CH_4_ and δ^13^C-C_2_H_6_ are shown.

As shown in Figure 4a, the δ^13^C-CH_4_ and δ^2^H-CH_4_ values of Marcellus shale gas and overlying Middle and Upper Devonian gases sampled from the annular spaces of casing strings in the Dimock Township plot along a continuous trend. Based on the well-documented occurrence of less thermally mature methane in Middle and Upper Devonian Formations throughout the Northern Appalachian basin (Jenden et al. 1993; Osborn and McIntosh 2010), this trend may be interpreted to represent increasing thermal maturity, where Marcellus shale gas (buried at greatest depths) displays the most enriched isotopic signature (i.e., greatest thermal maturity).

It is important to note that mixing lines among gases of differing thermal maturities can, in certain instances, produce a pattern similar to that of a thermal maturity trendline. In this regard, our study faces similar challenges to prior studies on the origin of thermogenic gases in the Appalachian basin (e.g., Jenden et al. 1993). Nevertheless, the differences in δ^13^C-CH_4_ and δ^2^H-CH_4_ values for Marcellus production gases in the Dimock Township vs. Middle and Upper Devonian gases from the annular spaces or well cellar of local gas wells indicates that, although plotting along a continuous trend, the two sources may be distinguished.

The δ^13^C value of ethane has also been utilized to provide additional information on the source of thermogenic hydrocarbon gases in the subsurface (Coleman 1994). Of the four samples of annular space and well cellar gases for which the δ^13^C value of ethane were measured, reported values range between –34 and –35‰. The six samples of Marcellus gas from the production pipeline/production casing display similar, though slightly depleted, δ^13^C-C_2_H_6_ values ranging between –35 and –36‰ (Figure 4b). All reported δ^13^C-C_2_H_6_ values of gases from annular spaces/well cellar, and production pipeline/production casing are more depleted than the corresponding δ^13^C-CH_4_ value, and therefore are said to exhibit an “isotope reversal” (i.e., a reversal of the trend traditionally observed in conventional hydrocarbon gases in which ethane is isotopically heavier, or more “enriched” in heavier isotopes, than methane). Such isotope reversals have commonly been observed in Marcellus and other Middle Devonian gases below the Tully limestone (Baldassare 2011). Comprehensive ethane isotope data are not currently available for gases present throughout the stratigraphic section in the Dimock Township. However, the magnitude of the methane-ethane δ^13^C isotope reversal in samples from the annular spaces and well cellar (3 to 4‰ difference between methane and ethane δ^13^C values) and those from the production pipeline/production casing (5 to 7‰ difference) suggests that Marcellus production gases may be distinguished from Middle and Upper Devonian gases in the annular spaces/well cellar of local gas wells.

It is important to note that, because of local variations in thermal maturity and possible source material, the isotopic and molecular signatures of formation gases in the Dimock Township cannot be extrapolated to represent the signatures of gases from the similar stratigraphic units in different parts of Susquehanna County or Pennsylvania. Consequently, the signatures of gases from the Marcellus and overlying Middle and Upper Devonian Units, as presented in Figure 4a and 4b, should only be considered representative of those found in the local Dimock area.

### Water Well and Salt Spring Gas Samples: Isotopic and Molecular Analyses, 2009 to 2010

In addition to characterizing gases originating from the Marcellus shale and overlying deposits in the Dimock Township, the Pennsylvania DEP and Cabot analyzed groundwater samples from 15 local Dimock area water wells for this same suite of isotopic analyses (details of well depth and construction provided in Table S1). Cabot also sampled one salt spring in Salt Springs State Park, a known historical source of methane gas located approximately 13 miles northeast of the Dimock Township. Although this sample is not representative of the immediate Dimock area, it does serve as an illustrative example of the composition of gases that have been migrating naturally in the shallow subsurface in south-central Susquehanna County prior to commencement of shale-gas extraction activities. In several instances, multiple samples were obtained over the 2-year period, resulting in 23 total water well samples (8 samples of dissolved gas, 15 samples of free gas) and 1 salt spring sample (free gas) analyzed at Isotech Laboratories in 2009 and 2010.

**Figure 5:**
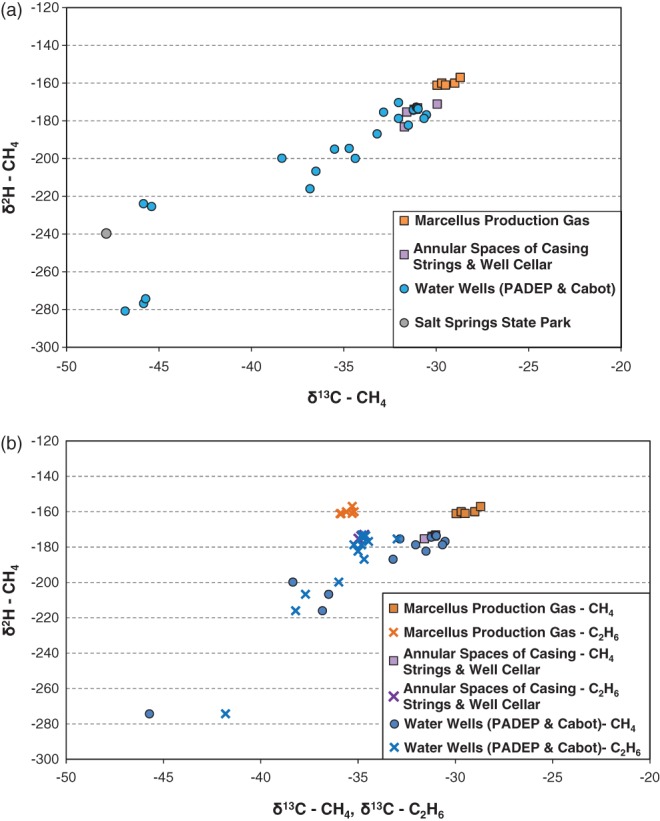
(a) Schoell plot showing Pennsylvania DEP and Cabot isotope data on methane samples from Dimock area water wells and Salt Springs State Park (Franklin Township). (b) Plot of δ^13^C values of methane and ethane (*x*-axis) vs. δ^2^H value of methane (*y*-axis) in gases sampled by the Pennsylvania DEP and Cabot from water wells in the Dimock Township. *Only samples with reported values of both δ^13^C-CH_4_ and δ^13^C-C_2_H_6_ are shown.

For 15 of the 23 water well samples, the ratio of methane to ethane is less than 100, suggesting thermogenic origin. The remaining eight water wells and one salt spring contain methane to ethane ratios ranging from 100 to over 6000. This wide spread of values could represent several distinct thermogenic and microbial gas sources, a mixture of different sources, and/or gases that have undergone alteration during migration. The δ^13^C and δ^2^H values of methane for these same samples are presented in Figure 5a. Each of the water well samples and the salt spring sample exhibit δ^13^C values (less than or equal to –30‰) and δ^2^H values (less than or equal to 170‰) that appear consistent with those of thermogenic Middle and Upper Devonian gases sampled from the annular spaces/well cellar, microbial gases, or a mixture of the two. Based on the δ^13^C-CH_4_ and δ^2^H-CH_4_ values alone, samples from at least 2 of the 15 water wells contain a clear microbial component (i.e., samples from these two wells plot close to or within the traditional Schoell “microbial-fermentation” gas zone: (i.e., δ^13^C-CH_4_ between –45 and –65‰, δ^2^H-CH_4_ ≤ –275‰)_._

The δ^13^C value of ethane was measured in 13 of the water well samples (originating from 10 different water wells). Of these 13 samples, 11 exhibited a methane-ethane δ^13^C isotope reversal (Figure 5b). Nine of these samples display combined methane and ethane δ^13^C values that appear most consistent with Middle and Upper Devonian gases sampled in the annular spaces of casing strings and the well cellar. An additional two samples still show a methane-ethane δ^13^C isotope reversal, but exhibit more depleted methane and/or ethane δ^13^C values than annular space/ well cellar gases. Lastly, two water samples did not exhibit an isotope reversal, and displayed notably more depleted methane and ethane δ^13^C values than gases sampled in the annular spaces and well cellar.

### Isotopic and Molecular Analyses by Osborn et al. (2011)

**Figure 6:**
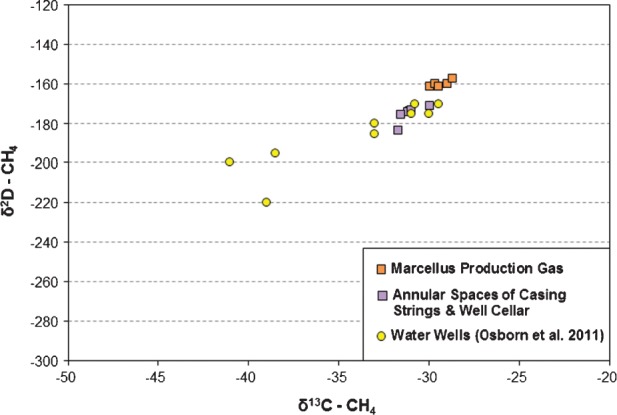
Schoell plot showing Osborn et al. (2011) isotope data on dissolved methane samples from Dimock area water wells.

In 2011, Osborn et al. (2011) presented δ^2^H and δ^13^C values for dissolved gases in nine water wells sampled the previous year in “active-gas extraction areas” (i.e., within 1 km of an active gas well) in Susquehanna County. A map of sampling locations in Susquehanna County (provided in Osborn et al. 2011) indicates that a majority of the water wells sampled in active gas extraction areas were located in close proximity to the Dimock Township. These dissolved gases exhibited δ^2^H and δ^13^C values of methane that are generally more depleted than, or on the border of, the range characterized by local Marcellus production gases sampled by the Pennsylvania DEP and Cabot in the Dimock area, consistent with the signatures of Middle and Upper Devonian gases in the annular spaces/well cellar (Figure 6).

### Isotopic and Molecular Analyses by USEPA (2012)

The USEPA also analyzed dissolved gases in 11 water wells in the Dimock Township in 2012 as part of follow-up sampling to the original Pennsylvania DEP stray-gas investigation (Table S6). Eight of the 11 water wells display combined δ^2^H and δ^13^C values of methane that are distinct (i.e., more depleted) from local Marcellus production gases. However, 3 of the 11 water wells contain methane with δ^2^H and δ^13^C values that plot within the elevated range of values that characterizes Marcellus production gas in the Dimock Township (δ^13^C-CH_4_ > –30‰, δ^2^H-CH_4_ > –162‰). On this basis alone, these dissolved gases appear to match production gas samples from the Marcellus shale. However, closer examination of the data from these three water wells suggests that microbial oxidation in the subsurface is likely responsible for the elevated δ^2^H and δ^13^C values.

Specifically, samples from two of these three water wells (House 4 and 14) exhibit δ^13^C-CH_4_ values that are slightly more enriched (δ^13^C-CH_4_ = –25 to –27‰) than those exhibited by Marcellus production gas samples (δ^13^C-CH_4_ = –28 to –30‰). However, these same samples display δ^2^H-CH_4_ values that are significantly more enriched (–122 and –140‰) than local Marcellus production gas (δ^2^H-CH_4_ = –161 to –157‰). The particularly elevated δ^2^H signatures of the methane in these two samples is suggestive of microbial oxidation of methane, whereby both the δ^2^H and δ^13^C values of methane are altered to be enriched, but the change in the δ^2^H value of methane is 8 to 14 times greater than the change in the δ^13^C value of methane (Coleman et al. 1981).

The sample from the third water well (House 2) exhibits δ^2^H and δ^13^C values (–160.5‰ and –29.4‰, and respectively) that plot in the depleted end of the isotopic range characterized by Marcellus shale gas. However, the sample from House 2 does not exhibit an isotope reversal between the δ^13^C values of methane and ethane that characterizes Marcellus shale gas samples.

Evaluation of a more complete suite of geochemical analyses over time could help discern the degree to which mixing of different gas sources and alteration processes in the subsurface (e.g., oxidation) have affected the signatures of water well gas samples collected by various parties in Susquehanna County.

## Discussion

### Isotopic and Molecular Analyses in Dimock Township

The δ^2^H and δ^13^C values of gases in Dimock area water wells sampled by the Pennsylvania DEP, Cabot, Osborn et al., and the USEPA indicate that hydrocarbon gases present in the majority of the water wells are consistent with gases in the well cellar and annular spaces of casing strings that intersect Middle and Upper Devonian formations above the Marcellus. The presence of a δ^13^C methane-ethane isotope reversal in numerous water wells would suggest a source below the Tully Limestone in the Middle Devonian Mahantango Formation penetrated by several of the annular spaces sampled. These findings support the conclusion that the methane concentrations in these water wells can be explained with no necessary contribution from deeper Marcellus shale gas.

Isotopic and molecular analyses of hydrocarbon gases can provide valuable information on the source of natural gases, but not necessarily the mechanism of migration. In this article, for the Dimock Township, we have evaluated available isotopic and molecular analyses of local water well samples with the primary goal of assessing gas origin. To identify gas migration pathways, geochemical data should be assessed in conjunction with other lines of evidence which are not within the scope of this article. These lines of evidence include details on the construction, completion, and integrity of local gas wells and water wells. In addition, the variation of methane concentrations and groundwater quality in potentially impacted water wells over time, as well as the spatial distribution of methane concentrations in groundwater relative to potential locations of gas sources, can provide crucial information on potential pathways of migration.

The isotopic and molecular analyses presented in this paper primarily represent measurements of hydrocarbon gases in local strata and water wells in the Dimock Township. Without additional analyses of samples throughout the greater Susquehanna County area, the signatures of hydrocarbon gases in local area gas and water wells cannot be extrapolated to represent those of methane or ethane that may be found in strata or water wells throughout the region. However, the evaluation of Dimock area isotope and molecular analyses of methane and ethane provide valuable insight into the complexity associated with using geochemical fingerprinting to identify the origin of hydrocarbon gases in regions where multiple thermogenic gas sources are present naturally.

### Consideration of Regional Lines of Evidence Regarding Sources of Methane in Groundwater

Methane has been present naturally throughout Susquehanna County in the shallow subsurface for at least 200 years, as indicated by the well-documented history of hydrocarbon gas shows during water well and gas well drilling, as well as natural seeps of hydrocarbon gases observed in the form of bubbling springs, ponds, and water wells. Our evaluation of 1701 recent predrill water well samples from Susquehanna County confirms that methane is common in drinking water aquifers today, with approximately 78% of sampled water wells exhibiting detected methane concentrations, and 3.4% exceeding the 7000 µg/L Pennsylvania DEP action level. Elevated methane concentrations show a clear relation to topography, with levels above the Pennsylvania DEP action level disproportionately found in valleys.

Furthermore, methane concentrations in valley water wells in gas production areas (i.e., located within 1 km of an active gas well) versus valley water wells in nonproduction areas (i.e., greater than 1 km from a gas well) show no statistical difference, indicating that the regional presence of elevated methane concentrations in valleys is a natural phenomenon. This is not an original finding, as the presence of elevated methane levels in valley water wells in the Appalachian basin has also been observed in a study in West Virginia by Mathis and White (2006), which found that methane concentrations from 170 water wells that exceeded 10,000 µg/L occurred in wells located in valleys and hillsides, as opposed to hill tops (Mathis and White 2006).

The correlation of methane concentrations with topography, rather than areas of active shale gas extraction, indicates that the use of hydraulic fracturing for shale gas in northeastern Pennsylvania has not resulted in widespread gas migration into the shallow subsurface. Certainly, as described in the 1980s by Harrison (1983, 1985), and by Gorody (2012) and King (2012), there have been instances of stray gas migration associated with the accumulation of gas pressure within and around the sides of the annular spaces of gas well casing strings in Pennsylvania, Ohio, and New York. However, the absence of a regional-scale relationship of methane concentrations to shale-gas well locations is consistent with the experience that gas well integrity and over-pressurization problems commonly result in localized, rather than regional, effects on water quality (van Stempvoort et al. 2005).

Valley water wells in Susquehanna County exhibit median methane concentrations similar to that of upland water wells; however, the 90th percentile concentrations of methane in valley wells is significantly elevated relative to upland wells. This observation suggests that some valley water wells access natural sources of elevated methane via interconnection with specific groundwater units and/or enhanced pathways of methane migration. As shown in Figures 3a, 3b, and S1, the elevated methane concentrations observed in this data set are predominantly associated with Ca-Na-HCO_3_ or Na-HCO_3_ water types, which in combination comprise only 16% of the sample population, yet represent 69% of the methane concentrations greater than 1000 µg/L. In addition, although only four water samples match a Na-HCO_3_-Cl or Na-Cl water type, three of these samples exhibit methane concentrations exceeding 10,000 µg/L. Samples with methane concentrations greater than 1000 µg/L also exhibit relatively elevated levels of barium, chloride, manganese, pH, sodium, strontium, and TDS, and relatively lower levels of sulfate and calcium. Similar geochemical relationships with elevated methane concentrations were previously reported in a study by Perry et al. (2012) for over 14,000 predrill water well samples in Pennsylvania, Ohio, and West Virginia, as well as a study by Warner et al. (2012) for 426 shallow groundwater samples from six counties in northeastern Pennsylvania.

The association of sodium and sodium-chloride rich water types with elevated methane concentrations suggest that wells exhibiting higher methane levels are connected to deeper groundwater aquifers, which have experienced longer groundwater residence times (and therefore, longer rock-water interaction times), and/or are in contact with sodium-chloride rich waters that occur in deeper bedrock and aquifers. Specifically, water in the deeper parts of sandstone aquifers that contain carbonate components (e.g., shells or carbonate rich shale lenses) commonly transitions from Ca-HCO_3_ type water to Na-HCO_3_ or Na-Cl-HCO_3_ type water as a result of (1) the mixing of circulating freshwater with sodium-chloride water and/or brine that occurs in deeper bedrock and aquifers, and/or (2) progressive cation exchange, whereby calcium, magnesium (and to a lesser degree, potassium) ions replace sodium on mineral exchange sites, thereby liberating sodium in groundwater (Cushing et al. 1973, Kresse et al. 2012). It follows that longer rock-water interaction results in increased cation exchange, and a transition towards sodium-bicarbonate water, containing greater concentrations of barium, boron, chloride, lithium, strontium, and TDS associated with the dissolution of carbonate and other minerals from the rock matrix. At the same time, concentrations of calcium and magnesium will decrease in groundwater with increased residence time in the subsurface, as these ions replace sodium in mineral exchange sites (Kresse et al. 2012).

Longer groundwater residence times are also associated with anaerobic groundwater conditions related to microbial consumption of oxygen and other electron acceptors (e.g., sulfate, manganese, and iron oxides) in the presence of an organic substrate. Evidence for the occurrence of these redox processes can include increased concentrations of manganese and iron from the reduction of iron and manganese oxides, as well as decreased sulfate concentrations (and associated sulfide odors) related to sulfate reduction (Kresse et al. 2012).

In Susquehanna County, deeper groundwater with longer residence times may be preferentially accessed by water wells that penetrate to the depth of deeper groundwater units or are in communication with fault and fracture networks. Comprehensive information on the depth of water wells sampled in our predrill dataset is not available. However, available information on aquifer characteristics in northeastern Pennsylvania indicates that the Lock Haven Formation contains discrete saline groundwater zones, which have been noted to produce methane gas and/or hydrogen-sulfide (Williams et al. 1998). Analysis of water quality by Warner et al. (2012) in northeastern Pennsylvania showed that water wells drawing from the Lock Haven Formation (45 wells) exhibit significantly elevated chloride and sodium concentrations (75thpercentile values of 73 and 103 mg/L, respectively) compared to water wells drawing from the Catskill Formation (102 wells, with 75th percentile values of 15 and 16 mg/L, respectively). Consequently, water wells that are in contact with Lock Haven groundwater, via either their greater depth within the overlying Catskill formation and/or fracture flow networks are more likely to exhibit decreased water quality and higher methane concentrations.

Valley formation in Susquehanna County has been associated with a greater density of fractures and lineaments, which likely serve as enhanced pathways for both water and gas migration. In West Virginia, Rauch (1984) found that water wells located within 0.1 km of photolineaments (short straight lineaments representing linear stream channel or valley segments related to the surface presentation of fault and fracture networks) had significantly higher water yields than other water wells. This increased water yield was not simply related to the influence of topography on groundwater flow, as valley water wells located near photolineaments exhibited greater water flow than all other valley water wells (Rauch 1984). It follows that water wells penetrating fault and fracture networks are not only likely to produce greater quantities of water, but higher methane levels as well.

It is important to note that the presence of methane itself can alter the concentrations of certain groundwater parameters. Specifically, the oxidation of methane is associated with the reduction of oxidizing reactants (i.e., sulfate, manganese, and iron oxide), an increase in pH and bicarbonate (product of CH_4_ oxidation) concentrations, and a decrease in oxidation-reduction potential (Kelly and Matisoff 1985). However, microbial oxidation should not affect the prevalence of chloride, which is a conservative ion (i.e., an ion that is not utilized in redox reactions, does not sorb readily to mineral surfaces or complex with other ions, and is not easily removed from solution) (Kresse et al. 2012). Consequently, the association of increasing chloride concentrations with methane concentrations strongly suggests the contribution of a deeper groundwater source with elevated chloride concentrations.

The significant history of gas production from the Lock Haven (and, to a lesser extent, the Catskill Formation) in northeastern Pennsylvania suggests that methane naturally present in many Susquehanna County water wells contains thermogenic components from the Catskill and Lock Haven formations. In addition, microbial methane, produced by methanogenesis in anaerobic groundwater units, may also be a strong component of the methane migrating into local drinking water wells. As discussed earlier, the present-day migration of deep thermogenic gases from the Mahantango and Marcellus Formations into the shallow subsurface in Susquehanna County appears to be limited by the presence of a regional structural detachment above the Tully stratigraphic level, which has been noted in local seismic interpretations. The data presented in this paper show that elevated methane concentrations exhibit a common association with (1) topographic lows and (2) groundwater geochemistry that reflects longer residence times and/or mixing with deeper NaCl waters. This observation suggests that elevated sources of either thermogenic or microbial methane from the Catskill and Lock Haven Formations are accruing in deep groundwater units, and/or within long discrete groundwater flow paths to lowland discharge points, that are preferentially accessed by certain valley water wells.

This evaluation represents a regional-scale assessment of trends observed in groundwater geochemistry in a large dataset during a 3-year period. However, for an individual water well, it is important to remember that factors such as proximity to roads (and therefore, the application of sodium and calcium chloride during the winter season) can result in localized variations in chloride, total dissolved solids, and other water quality parameters. In addition, seasonal fluctuations in aquifer recharge (i.e., increased influx of meteoric water between November and May), residential water use, changing weather patterns, and local well construction can also affect site-specific water quality.

## Conclusion

Our evaluation of 1701 groundwater quality analyses shows that methane is common in Susquehanna county water wells and is best correlated with topography and groundwater geochemistry, rather than shale-gas extraction activities. This finding suggests that shale-gas extraction in northeastern Pennsylvania has not resulted in regional gas impacts on drinking water resources and that, in turn, the hydraulic fracturing process has not created extensive pathways by which gas from the Marcellus shale could rapidly migrate into the shallow subsurface.

Our evaluation of site-specific isotopic and molecular data from water wells in the Dimock Township suggests that hydrocarbon gases present in these water wells are consistent with Middle and Upper Devonian gases above the Marcellus sampled in the annular spaces of local gas wells. This evaluation also emphasizes the complexity associated with differentiating between multiple thermogenic gas sources that may exhibit subtle variations. Isotopic analyses were not performed on the 1701 predrill water well samples to determine the origin of elevated methane concentrations observed throughout Susquehanna County. However, consideration of regional geology, historical publications, structural data, water well completion records, and water quality data suggest that methane naturally migrating into Susquehanna County water wells is either thermogenic, likely originating from Upper Devonian deposits overlying the Marcellus shale, or microbial, originating from anaerobic groundwater units with long residence times.

## Supporting Information

**Appendix S1.** Supporting Information for L.J. Molofsky et al. (2013).

**Figure S1.** Concentration distribution of groundwater analytes. Plots showing the concentrations of alkalinity, barium, calcium, chloride, magnesium, manganese, pH, potassium, sodium, strontium, sulfate, and TDS by methane concentration range (i.e., nondetect (ND), greater than ND to 1000 μg/L, greater than 1000 to 7000 μg/L, greater than 7000 μg/L).

**Figure S2.** Methane concentrations vs. methane to ethane ratios of water well samples for which both methane and ethane were detected.

**Figure S3.** Map of water and gas wells sampled for isotopic and molecular analyses in the Dimock township.

**Figure S4.** Locations of Historical Gas Wells, Gas Fields, Salt/Mineral Springs, and Shows of Saline Water and Combustible Gases in Water Wells.

**Table S1.** Construction Details of Water Wells Sampled for Isotopic and Molecular Analyses of Gases in the Dimock Township

**Table S2.** Isotopic and Molecular Analyses of Gas Samples from Water and Gas Wells in the Dimock Township

**Table S3.** Isotopic and Molecular Analyses of Split Gas Samples from Water Wells and Salt Springs State Park Analyzed at GeoMark Research, Ltd.

**Table S4.** Isotopic and Molecular Analyses of Desorption Canister Gas Samples from Gas Wells in the Dimock Township

**Table S5.** Osborn et al. (2011) Plotted Isotopic Data of Dissolved Gases in Water Wells Penetrating the Catskill Aquifer in an “Active Gas Extraction Area”

**Table S6.** U.S. EPA (2012) Isotopic and Molecular Analyses of Dissolved Gases in Water Wells Located in the Dimock Residential Groundwater Site

**Table S7.** Details of Historical References to Locations of Gas Wells/Fields (yellow), Water Wells Containing Combustible Gases (red), Water Wells Containing Saline Water (green), and Salt/Mineral Springs (blue)

**Table S8.** Groundwater Quality Data for 1701 Water Well Samples from Susquehanna County, Pennsylvania
