# Supplementary material for: Evaluation of Methane Sources in Groundwater in Northeastern Pennsylvania
Source: Ground Water. 2013 Apr 5;51(3):333–49. doi: 10.1111/gwat.12056 (PMC3746116; doi:10.1111/gwat.12056)
Supplement: Supplementary file 1 [file gwat0051-0333-SD1.pdf]

SUPPORTING INFORMATION

Evaluation of Methane Sources in Groundwater in Northeastern Pennsylvania

Lisa J. Molofsky, John A. Connor, Albert S. Wylie, Tom Wagner, Shahla K. Farhat

1. Collection and Analysis of Water Quality Samples

Pre-Drill Water Well Sampling Guidelines – History

Prior to February 2012, Pennsylvania Department of Environmental Protection (DEP) regulations stipulated that oil and gas operators in Pennsylvania sample water wells within 1000 feet of a proposed gas well prior to drilling in order to maintain the right to contest any subsequent claims of groundwater impact. These "pre-drill" water well samples were intended to establish baseline conditions in an area prior to the drilling of each specific gas well. In February of 2012, the Pennsylvania DEP expanded the recommended sampling radius to 2500 feet (PA DEP 2012). In anticipation of this change, several oil and gas operators voluntarily began sampling water wells within a 2500 foot radius of proposed gas wells several months prior to the updated regulations.

Collection and Laboratory Analysis of Pre-Drill Water Well Samples

Between 2008 and 2011, Cabot Oil and Gas Corporation (hereafter "Cabot") directed the collection of over 900 pre-drill samples in Susquehanna County from water wells within the recommended 1000 foot and 2500 foot radius of proposed gas wells. Several hundred additional water wells located in an 80-square mile area within Brooklyn, Harford, and Gibson Townships in Susquehanna County were also sampled in 2010 and 2011 to establish baseline dissolved gas concentrations in areas which did not have significant existing gas development operations at the time. Collectively, these samples comprised a dataset of 1701 water well samples evaluated in this paper.

Samples were collected by independent environmental consultants from the first available point of access, which included the spigot at the wellhead or before the pressure/treatment tank, the spigot at the base of the pressure tank, or from the sink tap within the residence. Water was allowed to flow for approximately 15 minutes prior to sample collection, or until stabilization of the following groundwater parameters: conductivity, pH, and temperature. The pre-drill analytical suite utilized by Cabot evolved during the sampling period of 2008 through 2011. Prior to late 2010, pre-drill water well samples were primarily analyzed for dissolved metals, BTEX, surfactants, dissolved gases, coliform, and general groundwater quality parameters (i.e., pH, alkalinity, chloride, sulfate, TDS). Water well samples were filtered in the field and preserved, or in instances where field filtration was not possible, unpreserved samples were filtered at the laboratory prior to analysis.

In the last months of 2010, Cabot standardized the selected suite of pre-drill analyses with the recommended list of analytes provided by the Marcellus Shale Coalition (MSC), which included total, rather than dissolved, metals (MSC, 2010). As a result, field or laboratory filtration was no longer conducted prior to preservation and analysis of pre-drill water well samples. For those water wells located in the 80-square mile area within Brooklyn, Harford, and Gibson Townships, samples were primarily analyzed exclusively for concentrations of dissolved gases (methane, ethane, propane). After collection, water samples were placed in a cooler and maintained on ice for shipment to the analytical laboratory. Water samples were analyzed at the following NELAC

accredited laboratories: American Westech Inc., Quantum Labs, Test America Labs, and Benchmark Analytics.

Evaluation of Pre-drill Water Quality Data

Methane concentrations were analyzed for in all 1701 pre-drill water samples. However, because the pre-drill analytical suite evolved over time, comprehensive data-sets are not available for every groundwater analyte. A complete table of pre-drill data is provided in Table S8 at the end of this supporting information.

Statistical analyses of pre-drill data were conducted using Statistical Software ProUCL 4.1.01, which is publically provided by the United States Environmental Protection Agency (US EPA). Approximately 22% of the 1701 methane samples exhibited non-detect values. As a result, the populations of methane concentrations in valley and uplands, as well as gas-production and non-production areas, were not normally distributed. Consequently, a non-parametric Mann-Whitney U test was utilized to statistically compare the methane concentrations in these groups.

To evaluate the presence of an association between various groundwater analytes and methane concentrations, the population of pre-drill groundwater samples was divided into four groups based on methane concentration: non-detect (ND), >ND to 1000 ug/L, >1000 to 7000 ug/L, and >7000 ug/L (where 7000 mg/L is the current Pennsylvania DEP action level). Concentrations of dissolved and total metals were combined for the purposes of determining population distributions. The minimum, 25th percentile, 50th percentile, 75th percentile, and maximum concentrations were determined for the population of reported values for the following analytes: alkalinity, barium, calcium, chloride, magnesium, manganese, pH, potassium, sodium, strontium, sulfate, and TDS. If greater than 25% of the reported values were not detected, a Kaplan Meier analysis was utilized to determine the population distribution. Box and whisker plots showing the distribution of concentrations for alkalinity, barium, calcium, chloride, magnesium, manganese, pH, potassium, sodium, strontium, sulfate, and TDS are provided in Figure S1.

The relative concentrations in milliequivalents per liter of major cations (calcium, magnesium, potassium, and sodium) and anions (bicarbonate, chloride, and sulfate) were utilized to determine water type for each of the 408 pre-drill samples for which all major ions were measured, according to the characteristic ion compositions of water types presented in Deutsch (1997). Bicarbonate concentrations were not measured; however, carbonate alkalinity was reported for each sample and converted to bicarbonate concentrations under the assumption that bicarbonate represented the vast majority of alkalinity present in solution at the pH values displayed by the great majority of samples (<8.4). The distribution of groundwater types present within the dataset of 408 pre-drill samples is shown on Figure 3b in the body of the manuscript.

Figure S1. Concentration Distribution of Groundwater Analytes. Plots showing the concentrations of alkalinity, barium, calcium, chloride, magnesium, manganese, pH, potassium, sodium, strontium, sulfate, and TDS by methane concentration range (i.e. non-detect (ND), >ND to 1000 ug/L, >1000 to 7000 ug/L, >7000 ug/L).

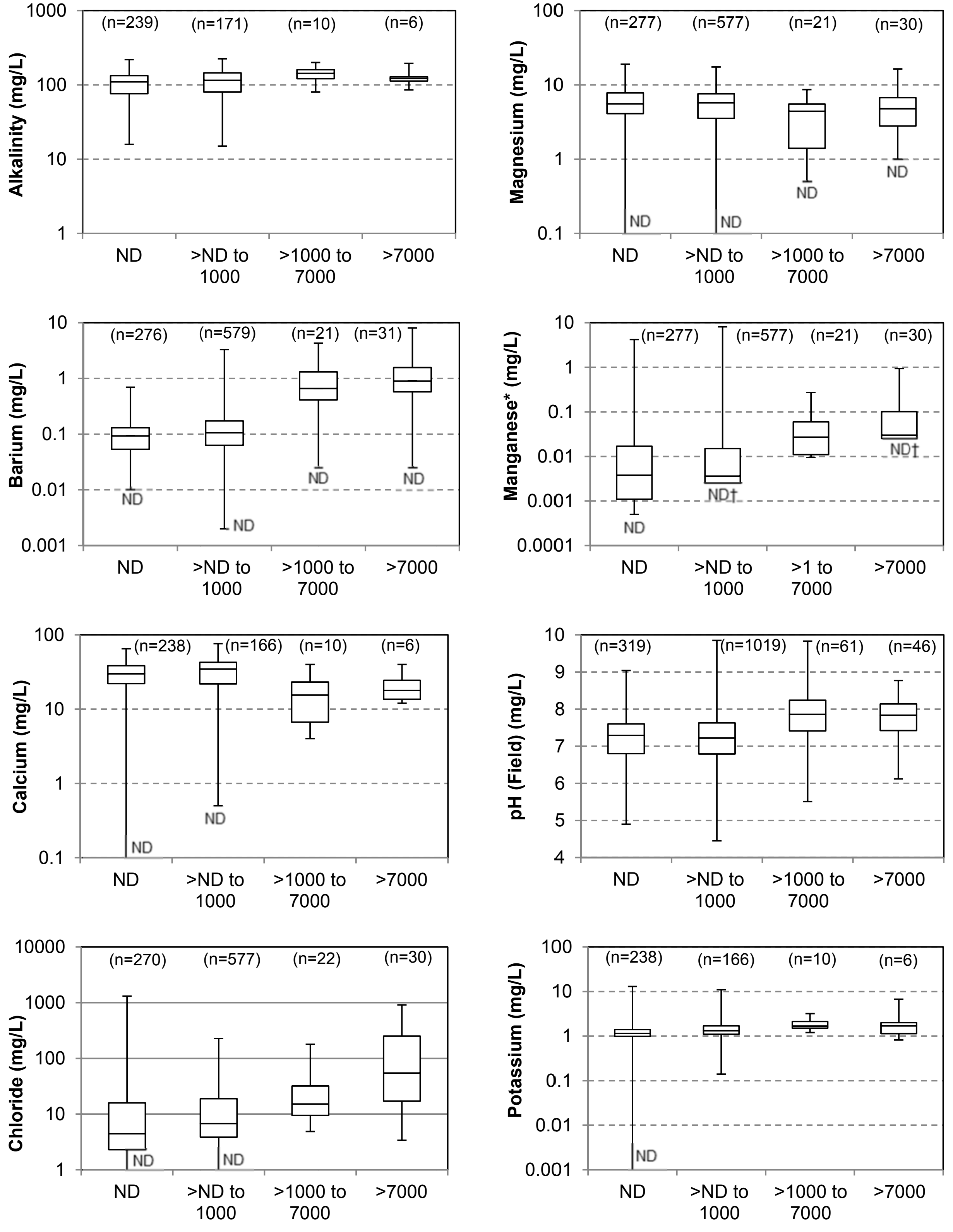

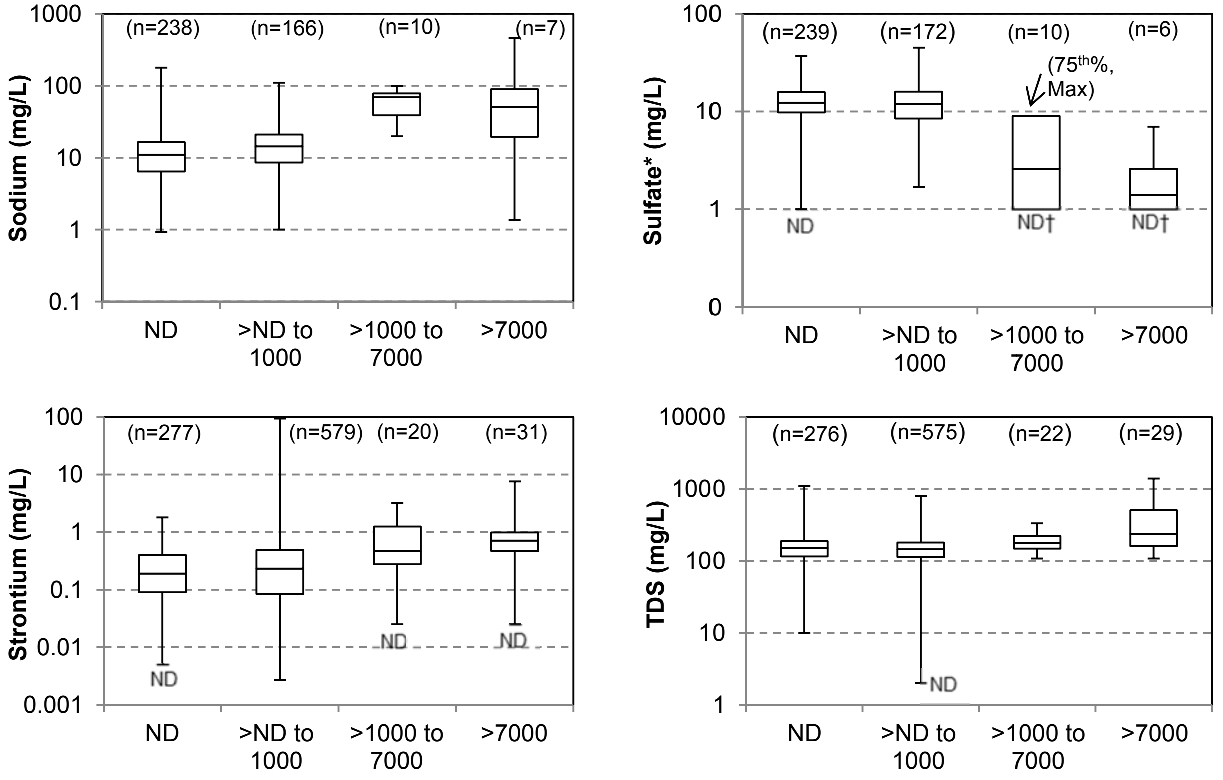

Notes:

1. (*) indicates compounds for which statistics were conducted using the Kaplan-Meier method.
2. (ND) indicates a non-detect value, with the detection limit depicted on the plot.
3. (†) indicates a non-detect value reported for both the 25th percentile and minimum.
4. (n =) indicates the total number of reported values.

Identification of Gas Production vs. Non-Production Areas, and Valleys vs. Uplands

For the purposes of this paper, pre-drill samples were grouped into those obtained from water wells located in “gas production areas” (defined as the area within 1 kilometer of an active gas well) versus “non-production areas” (i.e., located greater than 1 kilometer from an active gas well). For this determination, the coordinates and completion dates of gas wells drilled through October 2011 in Susquehanna County were obtained from the Pennsylvania Department of Conservation and Natural Resources (PA DCNR 2011). The location and sampling date of each pre-drill water well sample was then compared to the location and completion date of local gas wells. Using this method, 322 of the 1701 water wells were characterized as located in a “gas production area” at the time of sampling (i.e., gas well drilled within 1 kilometer of the water well prior to the sampling event), while 1379 of the 1701 water wells are considered to be located in a “non-production area.” Pre-drill sample locations were also plotted on a Light Detection and Ranging (LiDAR) bare-earth elevation map overlain with the National Hydrography Dataset (NHD) to determine their topographic location (i.e., valleys versus uplands). Valleys were categorized as those areas located within 1000 feet of a major NHD flowline (i.e., first-order named streams; e.g., “Tunkhannock Creek”), or within 500 feet of minor tributaries to NHD flowlines (i.e., unnamed streams and creeks).

Methane to Ethane Ratios of Pre-Drill Water Well Samples

Of the 1701 pre-drill samples, 1540 samples were analyzed for both methane and ethane concentrations. Figure S2 shows the methane concentrations and methane to ethane ratios for exactly 217 of these samples that contained both ***detected*** methane and ethane. These ratios are discussed in detail in the body of this paper. The dashed red line in Figure S2 represents the most common ethane detection limit (0.025 ug/L) reported by laboratories. Since Figure S2 only contains samples for which both methane and ethane were detected, the ethane detection limit serves to restrict the ratios of methane to ethane that are plotted for any given concentration of methane. For example, if a sample contains a methane concentration of 100 ug/L, the largest possible methane to ethane ratio for that sample, given a reported ethane detection limit of 0.025 ug/L, would be 4000 (assuming that both methane and ethane were detected). Consequently, it follows that much of the pre-drill data would plot below the dashed red line defined by the 0.025 ug/L ethane detection limit, if the ethane detection limit were lower. It is important to note that a small percentage of the pre-drill water well samples were analyzed at laboratories with reported ethane detection limits below 0.025 ug/L. These samples plot below the dashed red line in Figure S2.

Figure S2. Methane Concentrations Versus Methane to Ethane Ratios of Water Well Samples for Which Both Methane and Ethane Were Detected.

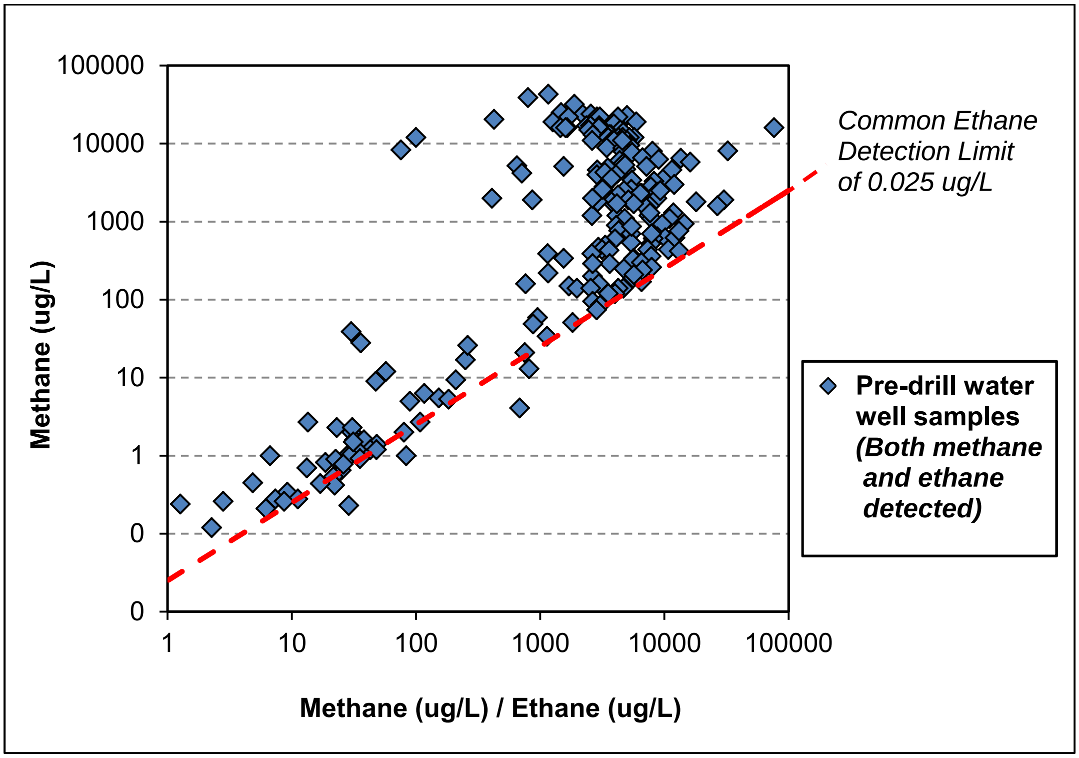

2. Collection and Analysis of Isotopic and Molecular Data

Collection of Free and Dissolved Gas Samples from Water Wells and Salt Spring

Samples of water well gases were collected by the Pennsylvania DEP and Cabot for isotopic and molecular analyses in 2009 and 2010 as part of an ongoing stray gas investigation in the Dimock Township. The Pennsylvania DEP collected 13 free-gas samples from 11 water wells during this period. All free-gas samples were collected in Tedlar bags, which were typically purged with sample gases three times prior to collection. In instances where methane comprised more than 2% of gas in the headspace of the water well, samples were collected in Tedlar bags directly from the ports on accessible vents or sanitary seals on the water wells. Where headspace gas did not contain greater than 2% methane, well water was first pumped into a confined container, and methane was subsequently collected from the container headspace. Tedlar bags were shipped to Isotech Laboratories for analysis.

Cabot contracted Civil and Environmental Consultants (CEC) to collect an additional 8 samples of dissolved gas in water wells in the Dimock Township in November 2010. Water was purged from the well for approximately 15 minutes prior to sample collection, and samples were collected from the nearest accessible sampling port to the water wellhead prior to treatment. Water samples were collected in an Isotech dissolved gas bottle using the inverted bottle method, as detailed by Isotech Laboratories (2011). At the time of sampling, CEC also collected 6 duplicate samples for an intralaboratory comparison of isotopic and molecular analytical results between Isotech and GeoMark Research, Ltd. (GeoMark) Laboratories. Duplicate samples were collected in an identical manner to, and immediately following, the collection of the initial sample for shipment to Isotech. However, a small volume of atmospheric air was left at the top of the dissolved gas bottle intended for shipment to GeoMark, in accordance with GeoMark's procedures for analyzing gases within the existing headspace of the sample bottle.

All dissolved gas samples were maintained on ice and shipped within 48 hours of collection overnight to Isotech or GeoMark Laboratories for analysis. One sample of free-gas was also collected by Cabot from an effervescing salt spring in Salt Springs State Park, located in Franklin Township in Susquehanna County. A bag was first placed over the effervescing portion of the salt spring, and atmosphere was purged from the bag through an inserted tube until hydrocarbon gases were detected using a portable gas chromatograph. Sample flow was then diverted into an IsoTube gas sampler. The sample was subsequently shipped to Isotech Laboratories for analysis. A duplicate sample was collected immediately following the first sample for shipment to GeoMark Laboratories for analysis. Construction details and the geographic location of the water wells sampled for isotopic and molecular analyses are provided in Table S1 and Figure S3.

Table S1. Construction Details of Water Wells Sampled for Isotopic and Molecular Analyses of Gases in the Dimock Township.

Resident Number	Topographic Location (Valley vs. Upland)	Information Source	Year Drilled	Depth (ft)	Casing Depth (ft)	Casing Diameter (in)	Depth to Bedrock (ft)	Pump Depth (ft)
1	Valley (major drainage)	Homeowner	---	---	---	---	---	---
2	Valley (major drainage)	Homeowner	---	250	---	---	---	---
3	Upland, but on edge of valley (major drainage)	PAGWIS Water Well Record	2001	300	40	6	25	---
4	Valley (major drainage)	Homeowner	---	250	---	---	---	---
5	Valley (major drainage)	Homeowner	---	250	200	---	---	---
6	Upland (approx. 1000 ft. away from valley (major drainage))	Homeowner	1971	425	---	6	---	385
7	Upland (approx. 1150 ft. away from valley (major drainage))	Homeowner	---	540	---	---	---	---
8	Upland (approx. 608 ft. away from valley (major drainage))	Homeowner	---	500	---	---	---	---
9	Valley (major drainage)	PAGWIS Water Well Record	1992	225	147	6	133	---
10	Valley (major drainage)	Homeowner	1992	230	170	---	---	170
11	Valley (major drainage)	Homeowner	1989/1990	175	92	6	---	---
12	Valley (major drainage)	Homeowner	1993	120	---	---	---	---
13	Valley (major drainage)	Homeowner	Before 1970	30	---	---	---	---
14	Valley (major drainage)	Cabot (Pump Pulled)	---	225	---	---	---	170
15	Valley (major drainage)	Homeowner	1994	250	30	6	---	---

Notes

1. (---) indicates the information is unknown.

Figure S3. Map of Water and Gas Wells Sampled for Isotopic and Molecular Analyses of Gases in the Dimock Township.

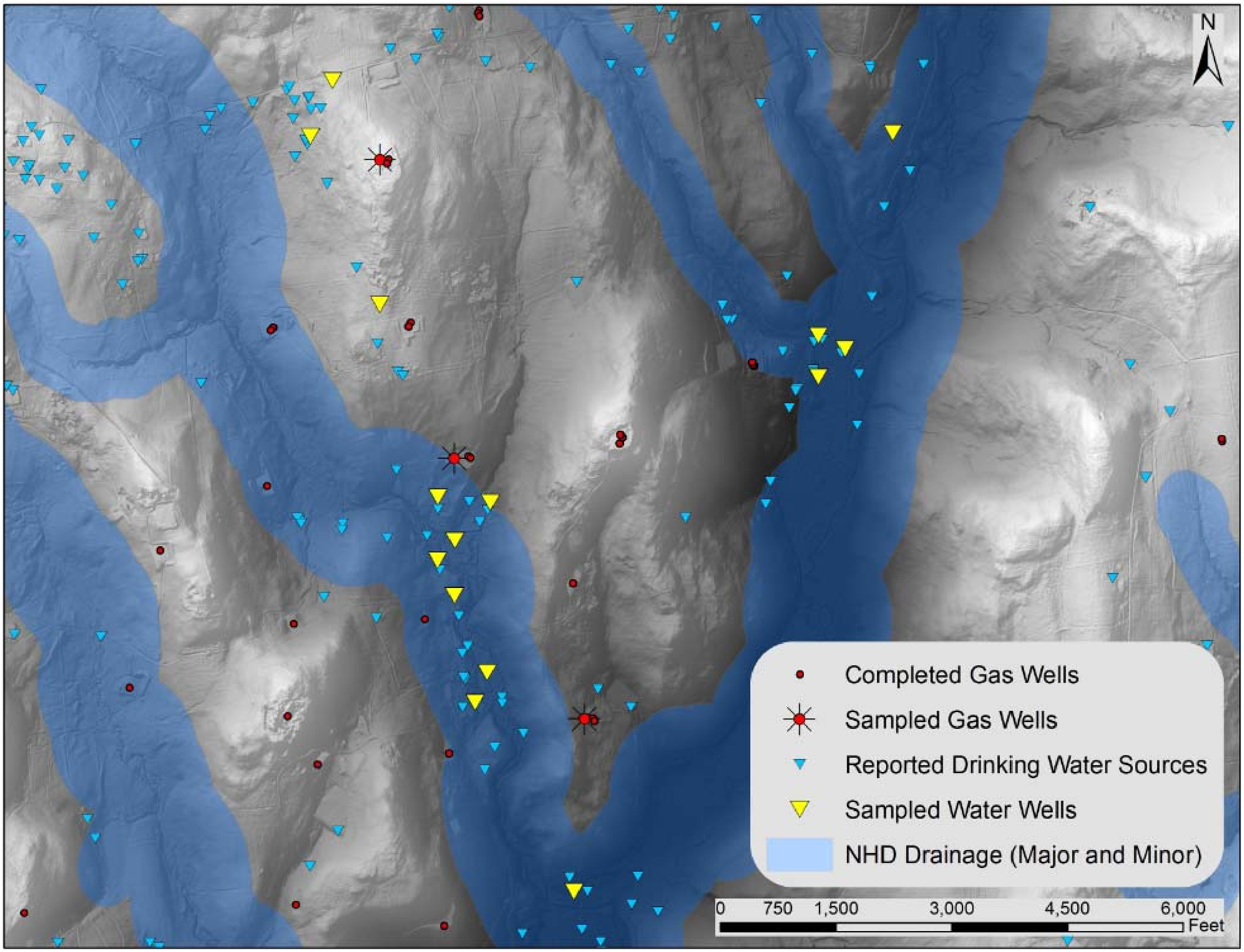

Collection of Free Gas Samples from Gas Wells and Wellbores

The Pennsylvania DEP also collected 1 sample of gas from a Marcellus gas well cellar, 5 samples of gas from the annular spaces adjacent to casing strings in Marcellus gas wells, and 2 samples of gas from within the production casing and production pipeline of a Marcellus gas well in the Dimock Township in 2009. Samples of gas from the annular spaces of casing strings and the production casing/ production pipeline were collected in Tedlar sampling bags directly from valves on the gas wellhead. Two Tedlar bag volumes were typically purged prior to sample collection. The gas well cellar sample was collected via a canister placed over the cellar, through which gas was pulled into a hose using an aspirator suction bulb until the presence of combustible gas was detected, at which time the sample was collected in a Tedlar bag. Cabot collected an additional 4 samples of free gas from Marcellus gas well production casings in the Dimock Township in 2011. These gas well samples were collected in IsoTubes using a gas sampling manifold. Samples collected by both the Pennsylvania DEP and Cabot from gas wells were shipped to Isotech Laboratories for analysis.

Lastly, Cabot collected 9 desorption canister samples of Marcellus gas from 2 wellbores that were later completed as shale gas extraction wells. Rock core from the wellbore was retrieved at the surface, where 1-foot sections from different members of the Marcellus Formation were cut and placed inside a sealed wax preserved canister. This canister was subsequently heated to either the mud circulating temperature, maximum bath temperature, or reservoir temperature (whichever was less). Each of the 9 desorption canister samples represents the first sample of desorbed gas collected in the field in gas sampling tubes, which were subsequently shipped to Isotech Laboratories for analysis.

Laboratory Analysis of Free and Dissolved Gas Samples from Water Wells, Salt Spring State Park, and Gas Wells

Samples of free gas from water wells and Salt Spring State Park (15 samples and 1 sample, respectively), dissolved gas from water wells (8 samples), and free gas from gas wells (12 samples) were analyzed at Isotech Laboratories for molecular and isotopic analyses. Isotopic analyses were conducted using either a Dual-Inlet Isotope Ratio Mass Spectrometer (Dual-Inlet IRMS), with reported $\delta^{13}C$ and $\delta^{2}H$ errors of ±0.1‰ and ±2‰, respectively, or a Gas Chromatograph Combustion Isotope Ratio Mass Spectrometer (GC-C-IRMS), with reported $\delta^{13}C$ and $\delta^{2}H$ errors of ±0.4‰ and ±5‰, respectively. Gas composition was analyzed at Isotech Laboratories using a Shimadzu Model GC-2010 Gas Chromatograph, with reported errors of ±2% of the reported value.

At the time of sampling, Cabot also sent 7 duplicate samples (6 samples of dissolved gas from water wells, and 1 sample of free-gas from a salt spring) to GeoMark Laboratories for isotopic and molecular analysis. Carbon and hydrogen isotopic analyses were conducted using a Gas Chromatograph Combustion Isotope Ratio Mass Spectrometer (GC-C-IRMS) with reported $\delta^{13}C$ and $\delta^{2}H$ errors of ±0.3‰ and ±1‰, respectively. Gas composition was analyzed using an Agilent Technologies 7890A Gas Chromatograph with reported errors of ±0.05 mole %. Reported $\delta^{13}C$-CH_4 results for duplicate samples analyzed at both Isotech and GeoMark Laboratories were within 2‰ of each other. However, $\delta^{2}H$-CH_4 results from GeoMark Laboratories were between 8 and 43‰ heavier (more enriched) than those reported by Isotech Laboratories. This disparity may be attributable to differences in the calibration and performance of analytical equipment (i.e., Dual-Inlet IRMS vs. GC-C-IRMS), or potentially, the laboratory protocol for sampling dissolved gases (Isotech injects helium to create headspace in the sample

bottle, whereas GeoMark Laboratories samples an existing headspace of atmospheric air left in the sample bottle at the time of collection). In the body of this paper, isotopic and molecular analyses from Isotech are exclusively presented (see Table S2), although results from GeoMark split samples are provided in Table S3.

Laboratory Analysis of Desorption Canister Samples from Gas Wellbores

Samples of gas from desorption canisters were analyzed separately for the molecular gas composition by Weatherford (WTF) Laboratories using an Agilent Technologies G6890N Gas Chromatograph with maximum reported errors of ±5% of the reported value. Carbon and hydrogen isotopic analyses were conducted by Isotech Laboratories using a GC-C-IRMS with reported $\delta^{13}C$ and $\delta^{2}H$ errors of ±0.4‰ and ±5‰, respectively. Reported $\delta^{13}C$-CH_4 values for several of these desorption canister samples were notably more enriched (i.e., $\delta^{13}C$-CH_4 values as much as 7.2‰ more positive) than Marcellus Formation gas samples from the production casing and production pipeline. In addition, desorption canister gas samples displayed significantly smaller ratios of methane to ethane (<31) than Marcellus production gas samples (universally >40). Such enrichment in ethane relative to methane, and $^{13}CH_4$ relative to $^{12}CH_4$, has been documented during the progressive desorption of coal gas (Strapoc et al. 2005; Yee et al. 1993). These changes appear to be related to the relative sorption strength of ethane versus methane and $^{13}CH_4$ versus $^{12}CH_4$ on the parent rock. As a result, analyses of desorption canister gases were not utilized to characterize isotopic and molecular values of Marcellus Formation gases in the body of this paper. However, these analyses are provided in Table S4 below.

Table S2. Isotopic and Molecular Analyses of Gas Samples from Water and Gas Wells in the Dimock Township.

Sampling Party & Analytical Laboratory	Sample Description	Sample Type	Sample Date	$\delta^{13}C$ - CH_4 (‰)	$\delta^{2}H$ - CH4 (‰)	$\delta^{13}C$ - C_2H_6 (‰)	H_2S (%)	CO (%)	He (%)	H_2 (%)	Ar (%)	O_2 (%)	CO_2 (%)	N_2 (%)	C_1 (%)	C_2 (%)	C_2H_4 (%)	C_3 (%)	iC_4 (%)	nC_4 (%)	iC_5 (%)	nC_5 (%)	C_6 (%)	C_{3+} (%)	Ratio C_1: C_2
**Water Well Samples**																									
DEP (Isotech)	Water Well 1	Free Gas	1/18/2009	-32.04	-170.3	NA	NA	0	0.0017	0	0.945	20.83	0.050	75.53	2.60	0.0410	0	0.0018	0	0	0	0	0	--	63.4
DEP (Isotech)	Water Well 1	Free Gas	10/6/2010	-33.20	-186.9	-34.69	ND	ND	0.0081	ND	0.326	2.4	0.060	21.36	74.58	1.26	ND	0.0018	ND	ND	ND	ND	ND	--	59.2
CEC (Isotech)	Water Well 1	Dissolved Gas	11/13/2010	-32.04	-178.7	-35.21	NA	0.00	0.0173	0	0.441	0.23	0.06	26.67	71.23	1.35	0.00	0.00	0.00	0.00	0.00	0.00	0.00	--	52.8
DEP (Isotech)	Water Well 2	Free Gas	10/6/2010	-30.53	-176.8	-34.46	ND	ND	ND	ND	0.725	12.50	0.17	42.87	42.77	0.968	ND	0.0012	ND	ND	ND	ND	ND	--	44.2
CEC (Isotech)	Water Well 2	Dissolved Gas	11/9/2010	-31.51	-182.3	-35	NA	0.00	0.018	0	0.345	0.16	0.13	17.76	79.98	1.60	0.00	0.00	0.00	0.00	0.00	0.00	0.00	--	50.0
CEC (Isotech)	Water Well 3	Dissolved Gas	11/12/2010	-31.24	-174.4	-34.77	NA	0.00	0.0225	0	0.28	0.072	0.01	14.99	83.46	1.15	0.00	0.01	0.00	0.00	0.00	0.00	0.00	--	72.6
DEP (Isotech)	Water Well 4	Free Gas	1/21/2009	-31.24	-174.1	NA	NA	0	0.0029	0	0.913	20.38	0.040	73.84	4.74	0.0828	0	0.0033	0	0	0	0	0	--	57.2
CEC (Isotech)	Water Well 5	Dissolved Gas	11/9/2010	-32.85	-175.4	-32.99	NA	0.00	NA	NA	1.31	0.93	0.34	78.66	18.59	0.17	0.00	0.00	0.00	0.00	0.00	0.00	0.00	--	109.4
DEP (Isotech)	Water Well 6	Free Gas	1/21/2009	-31.08	-172.7	NA	NA	0	0.0186	0	0.572	11.53	0.32	45.69	41.07	0.767	0	0.0263	0	0.0020	0	0.0023	0	--	53.5
DEP (Isotech)	Water Well 6	Free Gas	10/6/2010	-36.83	-216.0	-38.21	ND	ND	0.0058	ND	0.897	19.88	0.26	73.96	4.92	0.0796	ND	0.0013	ND	ND	ND	ND	ND	--	61.8
CEC (Isotech)	Water Well 6	Dissolved Gas	11/7/2010	-36.51	-206.7	-37.7	NA	0.00	NA	NA	0.554	1.16	3.44	26.58	66.94	1.31	0.00	0.01	0.00	0.00	0.00	0.00	0.00	--	51.1
DEP (Isotech)	Water Well 7	Free Gas	1/8/2009	-31.03	-173.1	-34.80	NA	0	0.0265	0	0.317	6.28	0.28	26.32	66.51	1.22	0	0.0416	0	0.0026	0	0	0	--	54.5
DEP (Isotech)	Water Well 8	Free Gas	1/12/2009	-30.66	-178.7	-34.82	0	0	0.0245	0	0.404	8.26	0.27	33.47	58.47	1.06	0	0.0355	0	0.0019	0	0	0	--	55.2
CEC (Isotech)	Water Well 9	Dissolved Gas	11/20/2010	-30.98	-173.6	-34.61	NA	0.00	NA	NA	0.626	0.46	0.22	34.41	63.01	1.27	0.00	0.00	0.00	0.00	0.00	0.00	0.00	--	49.6
CEC (Isotech)	Water Well 10	Dissolved Gas	11/9/2010	-38.35	-199.8	-35.99	NA	0.00	NA	NA	0.98	2.38	0.33	57.37	38.80	0.14	0.00	0.00	0.00	0.00	0.00	0.00	0.00	--	277.1
DEP (Isotech)	Water Well 11	Free Gas	10/6/2010	-34.71	-194.6	NA	ND	ND	0.0023	ND	0.538	5.32	0.077	36.60	57.45	0.0110	ND	ND	ND	ND	ND	ND	ND	--	5222.7
DEP (Isotech)	Water Well 11	Free Gas	6/16/2010	-35.50	-195.0	NA	NA	0	0.0014	0	0.906	19.87	0.045	74.91	4.27	0.0008	0	0	0	0	0	0	0	--	5337.5
DEP (Isotech)	Water Well 12	Free Gas	6/16/2010	-45.83	-276.8	NA	NA	0	0.0055	0	0.919	20.39	0.044	76.49	2.13	0.0195	0	0	0	0	0	0	0	--	109.2
DEP (Isotech)	Water Well 13	Free Gas	8/18/2010	-46.83	-280.8	NA	NA	0	0.0152	0.0207	0.892	20.05	0.042	74.25	4.66	0.0882	0	0.0017	0	0	0	0	0	--	52.8
CEC (Isotech)	Water Well 13	Dissolved Gas	11/10/2010	-45.72	-274.3	-41.81	NA	0.00	NA	NA	0.202	0.53	0.12	7.98	89.37	1.77	0.00	0.03	0.00	0.00	0.00	0.00	0.00	--	50.5
DEP (Isotech)	Water Well 14	Free Gas	*2/15/2010	-45.40	-225.4	NA	NA	0	0.0031	0	0.917	20.15	0.05	76.9	2	0.0003	0	0	0	0	0	0	0.0001	--	6666.7
DEP (Isotech)	Water Well 15	Free Gas	*8/24/2010	-45.84	-223.9	NA	NA	0	0.001	0	0.925	20.1	0.05	77	2.10	0	0	0	0	0	0	0	0	--	--
DEP (Isotech)	Water Well 15	Free Gas	6/16/2010	-34.38	-199.9	NA	NA	0	0	0	0.914	20.38	0.045	76.15	2.51	0.0009	0	0	0	0	0	0	0	--	2788.9
**Salt Spring Sample**																									
Cabot (Isotech)	Salt Springs State Park Spring	Free Gas	11/10/2010	-47.85	-239.7	NA	ND	ND	0.0056	ND	0.882	19.29	0.052	74.74	5.01	0.0178	ND	ND	ND	ND	ND	ND	ND	--	281.5
**Gas Well Samples: Annular Spaces Adjacent to Casing Strings and Well Cellar**																									
DEP (Isotech)	Gas Well 5: Composite Air Sample from Well Cellar	Free Gas	1/8/2009	-31.09	-173.8	-34.83	NA	0	0.0315	0.140	0.115	2.61	0	9.80	85.59	1.65	0	0.0599	0	0.0040	0	0	0	--	51.9
DEP (Isotech)	Gas Well 5: 20" X 13-3/8" Casing – Annular Space of Freshwater Casing String	Free Gas	1/13/2009	-31.20	-173.9	-34.64	0	0	0.0345	0.166	0.0270	0.573	0	2.40	94.91	1.82	0	0.0536	0.0019	0.0048	0	0	0	--	52.1
DEP (Isotech)	Gas Well 5: 13-3/8" X 9-5/8" Casing – Annular Space of Intermediate Casing String	Free Gas	1/13/2009	-31.59	-175.3	-34.97	0	0	0.0237	0.107	0.375	8.35	0.015	31.88	58.07	1.14	0	0.0411	0.0015	0.0027	0	0	0	--	50.9
DEP (Isotech)	Gas Well 6: 7" X 4-1/2" Casing – Annular Space of Production Casing String	Free Gas	2/3/2009	-29.95	-171.1	NA	NA	0	0.0326	0.0978	0.0089	0.163	0.011	0.78	96.36	2.42	0	0.112	0.0027	0.0079	0	0	0	--	39.8
DEP (Isotech)	Gas Well 1: 7" X 4-1/2" Casing – Annular Space of Production Casing String	Free Gas	1/8/2009	-31.01	-173.1	-34.68	NA	0	0.03333	0.012	0.0081	0.162	0	0.79	97.08	1.74	0	0.0576	0.0016	0.0044	0	0	0	--	55.8
DEP (Isotech)	Gas Well 1: 7" X 4-1/2" Casing – Annular Space of Production Casing String	Free Gas	2/2/2009	-31.72	-183.2	NA	NA	0	0.0397	0.215	0.0229	0.491	0	2.00	95.24	1.91	0	0.0711	0.0024	0.0049	0	0	0	--	49.9
**Gas Well Samples: Production Casing/ Production Pipeline**																									
DEP (Isotech)	Gas Well 1: 4-1/2" Production Casing	Free Gas	1/7/2009	-29.91	-161.1	-35.92	NA	0	0.0186	0.0208	0.0430	0.950	0.036	3.67	93.16	2.03	0	0.0691	0	0.0029	0	0	0	--	45.9
DEP (Isotech)	Gas Well 1: 2" Collection Pipeline	Free Gas	1/7/2009	-29.96	-161.1	-35.87	NA	0	0.0190	0.0222	0.0378	0.830	0.034	3.26	93.69	2.03	0	0.07	0	0.0031	0	0	0	--	46.2
Cabot (Isotech)	Gas Well 2H: 5-1/2" Production Casing	Free Gas	11/4/2011	-29.7	-160	-35.6	NA	ND	0.0206	0.0191	ND	ND	0.027	0.25	97.54	2.07	ND	0.0693	0.001	0.0032	0.0001	0.0001	0.0002	--	47.1
Cabot (Isotech)	Gas Well 4H: 5-1/2" Production Casing	Free Gas	11/4/2011	-29.0	-160	-35.2	NA	ND	0.0207	0.0057	ND	0.006	0.022	0.27	97.33	2.25	ND	0.0887	0.0015	0.0049	0.0002	0.0001	ND	--	43.3
Cabot (Isotech)	Gas Well 1V: 4-1/2" Production Casing	Free Gas	11/4/2011	-28.7	-157	-35.3	NA	ND	0.0203	0.0067	ND	ND	0.024	0.25	97.49	2.13	ND	0.0737	0.0011	0.0034	0.0001	0.0001	ND	--	45.8
Cabot (Isotech)	Gas Well 5H: 5-1/2" Production Casing	Free Gas	11/4/2011	-29.5	-161	-35.3	NA	ND	0.0197	0.0094	ND	ND	0.024	0.25	97.49	2.13	ND	0.0719	0.001	0.0031	0.0001	0.0001	0.0001	--	45.8

Notes

1. * = GC Date
2. NA = Not Analyzed; ND = Not Detected; -- = Not Reported
3. DEP = Pennsylvania Department of Environmental Protection; CEC = Civil and Environmental Consultants, Inc.; Cabot = Cabot Oil and Gas Corporation
4. Isotech = Isotech Laboratories, Inc.; GeoMark = GeoMark Research, Ltd.; Weatherford = Weatherford Laboratories
5. Isotech samples analyzed via offline preparation and dual inlet isotope ratio mass spectrometry (Dual-Inlet IRMS) are reported to two decimal places for $\delta^{13}C$ and to one decimal place for $\delta^{2}H$.
6. Isotech samples analyzed via continuous flow GC-C-IRMS are reported to one decimal place for $\delta^{13}C$ and with no decimal for $\delta^{2}H$.
7. DEP data was obtained from Pennsylvania Department of Environmental Protection records through a Freedom of Information Act (FOIA) request (PA DEP 2010a, 2010b).
8. Data values are presented as reported in Pennsylvania DEP data tables, and laboratory reports from Isotech Laboratories, GeoMark Research, Ltd., and Weatherford Laboratories provided by Cabot Oil and Gas Corporation.

Table S3. Isotopic and Molecular Analyses of Split Gas Samples from Water Wells and Salt Springs State Park Analyzed at GeoMark Research, Ltd.

Sampling Party & Analytical Laboratory	Sample Description	Sample Type	Sample Date	$\delta^{13}C$ - CH_4 (‰)	$\delta^{2}H$ - CH4 (‰)	$\delta^{13}C$ - C_2H_6 (‰)	H_2S (%)	CO (%)	He (%)	H_2 (%)	Ar (%)	O_2 (%)	CO_2 (%)	N_2 (%)	C_1 (%)	C_2 (%)	C_2H_4 (%)	C_3 (%)	iC_4 (%)	nC_4 (%)	iC_5 (%)	nC_5 (%)	C_6 (%)	C_{3+} (%)	Ratio C_1: C_2
**Water Well & Salt Spring Split Samples Analyzed at GeoMark Research, Ltd.**																									
Cabot (GeoMark)	Water Well 1	Dissolved Gas	11/13/2010	-33.5	-170	-35.4	0.00	NA	NA	NA	NA	NA	0.08	45.76	53.28	0.89	NA	0.00	0.00	0.00	0.00	0.00	0.00	--	59.9
Cabot (GeoMark)	Water Well 2	Dissolved Gas	11/9/2010	-33.1	-172	-34.3	0.00	NA	NA	NA	NA	NA	0.14	22.04	76.43	1.38	NA	0.00	0.00	0.00	0.00	0.00	0.00	--	55.4
Cabot (GeoMark)	Water Well 3	Dissolved Gas	11/2/2010	-32.7	-160	-35.4	0.00	NA	NA	NA	NA	NA	0.01	16.46	82.45	1.08	NA	0.01	0.00	0.00	0.00	0.00	0.00	--	76.3
Cabot (GeoMark)	Water Well 6	Dissolved Gas	11/7/2010	-38.2	-193	-37.3	0.00	NA	NA	NA	NA	NA	1.15	86.20	12.43	0.22	NA	0.00	0.00	0.00	0.00	0.00	0.00	--	56.5
Cabot (GeoMark)	Water Well 10	Dissolved Gas	11/5/2010	-39.2	-187	-35.9	0.00	NA	NA	NA	NA	NA	0.18	92.35	7.44	0.03	NA	0.00	0.00	0.00	0.00	0.00	0.00	--	248.0
Cabot (GeoMark)	Water Well 13	Dissolved Gas	11/10/2010	-46.4	-254	-41.9	0.00	NA	NA	NA	NA	NA	0.07	87.43	12.28	0.22	NA	0.00	0.00	0.00	0.00	0.00	0.00	--	55.8
Cabot (GeoMark)	Salt Springs State Park Spring	Free Gas	11/10/2010	-47.5	-197	-41.5	0.00	NA	NA	NA	NA	NA	0.05	97.07	2.87	0.01	NA	0.00	0.00	0.00	0.00	0.00	0.00	--	287.0

Notes

1. NA = Not Analyzed; ND = Not Detected; -- = Not Reported
2. Cabot = Cabot Oil and Gas Corporation
3. GeoMark = GeoMark Research, Ltd.
4. Data values are presented as reported in laboratory reports from GeoMark Research, Ltd. provided by Cabot Oil and Gas Corporation.

Table S4. Isotopic and Molecular Analyses of Desorption Canister Gas Samples from Gas Wells in the Dimock Township.

Sampling Party & Analytical Laboratory	Sample Description	Sample Type	Sample Date	$\delta^{13}C$ - CH_4 (‰)	$\delta^{2}H$ - CH4 (‰)	$\delta^{13}C$ - C_2H_6 (‰)	H_2S (%)	CO (%)	He (%)	H_2 (%)	Ar (%)	O_2 (%)	CO_2 (%)	N_2 (%)	C_1 (%)	C_2 (%)	C_2H_4 (%)	C_3 (%)	iC_4 (%)	nC_4 (%)	iC_5 (%)	nC_5 (%)	C_6 (%)	C_{3+} (%)	Ratio C_1: C_2
**Desorption Canister Samples: Marcellus Gas Wells**																									
Cabot (Weatherford)	Gas Well 3 Core Purcell Limestone	Desorption Canister	8/3/2011	-29.8	-172	-36.0	NA	NA	NA	0.00	NA	NA	2.66	NA	89.45	3.48	0.00	2.29	0.25	1.12	0.24	0.29	0.22	--	25.7
Cabot (Weatherford)	Gas Well 3 Core Lower Marcellus	Desorption Canister	8/3/2011	-22.7	-166	-36.6	NA	NA	NA	0.00	NA	NA	0.51	NA	94.76	4.48	0.00	0.16	0.01	0.04	0.01	0.01	0.01	--	21.2
Cabot (Weatherford)	Gas Well 3 Core Lower Marcellus	Desorption Canister	8/3/2011	-22.4	-162	-34.9	NA	NA	NA	0.00	NA	NA	0.43	NA	95.81	3.71	0.00	0.05	0.00	0.00	0.00	0.00	0.00	--	25.8
Cabot (Weatherford)	Gas Well 3 Core Top Union Springs	Desorption Canister	8/4/2011	-24.3	-171	-34.9	NA	NA	NA	0.00	NA	NA	0.20	NA	96.55	3.19	0.00	0.05	0.00	0.00	0.00	0.00	0.00	--	30.3
Cabot (Weatherford)	Gas Well 3 Core Middle Union Springs	Desorption Canister	8/4/2011	-27.3	-163	-35.5	NA	NA	NA	0.00	NA	NA	0.18	NA	96.19	3.52	0.00	0.09	0.00	0.00	0.00	0.00	0.02	--	27.3
Cabot (Weatherford)	Gas Well 3 Core Middle Union Springs	Desorption Canister	8/4/2011	-25.4	-168	-35.9	NA	NA	NA	0.00	NA	NA	0.30	NA	96.14	3.51	0.00	0.05	0.00	0.00	0.00	0.00	0.00	--	27.4
Cabot (Weatherford)	Gas Well 3 Core Base Union Springs	Desorption Canister	8/4/2011	-21.6	-165	-34.7	NA	NA	NA	0.00	NA	NA	0.62	NA	93.33	5.95	0.00	0.09	0.00	0.00	0.00	0.00	0.00	--	15.7
Cabot (Weatherford)	Gas Well 4 Core Upper Marcellus	Desorption Canister	5/27/2008	-24.78	-156.1	NA	NA	NA	NA	0.00	NA	0.00	0.925	0.00	89.527	9.194	NA	NA	NA	NA	NA	NA	NA	0.355	9.7
Cabot (Weatherford)	Gas Well 4 Core Lower Marcellus	Desorption Canister	5/31/2008	-21.54	-158.7	NA	NA	NA	NA	0.00	NA	0.00	0.252	0.00	93.263	6.147	NA	NA	NA	NA	NA	NA	NA	0.337	15.2

Notes

1. NA = Not Analyzed; ND = Not Detected; -- = Not Reported
2. Cabot = Cabot Oil and Gas Corporation
3. Weatherford = Weatherford Laboratories
4. Data values are presented as reported in laboratory reports from Weatherford Laboratories provided by Cabot Oil and Gas Corporation.

Table S5. Osborn et al. (2011) Plotted Isotopic Data of Dissolved Gases in Water Wells Penetrating the Catskill Aquifer in an "Active Gas Extraction Area".

Water Well	$\delta^{13}C$ - CH_4 (‰)	$\delta^{2}H$ - CH_4 (‰)
Unknown #1	-41	-200
Unknown #2	-39	-220
Unknown #3	-38.5	-195
Unknown #4	-33	-180
Unknown #5	-33	-185
Unknown #6	-31	-175
Unknown #7	-30.8	-170
Unknown #8	-30	-175
Unknown #9	-29.5	-170

Notes

1. All isotopic values presented are based on visual identification of data points on Fig. S2 in Osborn et al. (2011).
2. Water well locations were not provided in Osborn et al. (2011). For purpose of internal reference only, well names shown above were assigned by authors of Molofsky et al. (2013).

Table S6. U.S. EPA (2012) Isotopic and Molecular Analyses of Dissolved Gases in Water Wells Located in the Dimock Residential Groundwater Site.

Water Well	Sample Date	$\delta^{13}C$ - CH_4 (‰)	$\delta^{2}H$ - CH_4 (‰)	$\delta^{13}C$ - C_2H_6 (‰)	$\delta^{2}H$ - C_2H_6 (‰)	H_2S (%)	CO (%)	He (%)	H_2 (%)	Ar (%)	O_2 (%)	CO_2 (%)	N_2 (%)	C_1 (%)	C_2 (%)	C_2H_4 (%)	C_3 (%)	C_3H_6 (%)	iC_4 (%)	nC_4 (%)	iC_5 (%)
House 1	1/25/2012	-36.80	-202.4	-31.58	-177	NA	ND	0.0747	ND	0.683	0.20	0.005	49.91	48.69	0.432	ND	0.0004	0.0001	ND	ND	ND
House 2	1/25/2012	-29.36	-160.5	-28.83	-169	NA	ND	0.0110	ND	0.636	1.12	0.10	41.09	56.36	0.683	ND	ND	0.0001	ND	ND	ND
House 2 (Dup)	1/25/2012	-29.30	-160.6	-28.6	-166	NA	ND	0.0112	ND	0.628	0.80	0.094	40.72	57.06	0.687	ND	ND	0.0001	ND	ND	ND
House 4	1/24/2012	-24.98	-121.8	-31.2	-187	NA	ND	NA	ND	1.50	2.28	2.01	84.37	9.76	0.0796	ND	0.0004	ND	ND	ND	ND
House 5	1/26/2012	-33	-162.9	NA	NA	NA	ND	NA	ND	1.54	4.82	0.40	84.97	8.24	0.0259	ND	ND	ND	ND	ND	ND
House 6	1/26/2012	-31.07	-169.0	-34.43	-195.0	NA	ND	0.0248	0.0222	0.503	1.04	0.008	32.03	65.62	0.746	ND	0.0068	0.0001	ND	ND	ND
House 8a	1/25/2012	-36.58	-209.9	-35.9	-189	NA	ND	NA	ND	0.746	5.31	3.22	36.31	53.64	0.767	ND	0.0030	ND	ND	ND	ND
House 12	1/26/2012	-35.90	-196.7	-35.33	-204.0	NA	ND	0.0434	ND	0.115	0.16	0.073	4.54	94.06	0.987	ND	0.0221	0.0002	0.0006	0.0012	ND
House 14	1/26/2012	-26.58	-140.3	-26.6	-157	NA	ND	NA	ND	1.46	2.70	4.99	72.02	18.74	0.0899	ND	ND	ND	ND	ND	ND
House 17	1/27/2012	-31.54	-167.8	-32.9	-169	NA	ND	NA	ND	1.49	2.06	0.43	80.93	14.97	0.118	ND	0.0011	ND	ND	ND	ND
House 24	1/27/2012	-53.8	-165	NA	NA	NA	ND	NA	ND	1.58	1.29	0.017	94.00	3.11	ND	ND	ND	ND	ND	ND	ND
House 60	3/5/2012	-35.20	-193.0	NA	NA	NA	ND	0.0233	ND	0.516	0.066	0.053	29.47	69.86	0.0119	ND	ND	ND	ND	ND	ND

Notes

1. Data is publically available at: http://www.epaosc.org/site/doc_list.aspx?site_id=7555
2. NA = Not Analyzed; ND = Not Detected
3. Data values are presented as reported in laboratory reports from Isotech Laboratories provided by the U.S. EPA.

3. Historical Information Regarding the Presence of Natural Gases and/or Saline Water in the Shallow Subsurface

Historical notations regarding the presence of shallow gas shows, gas fields, salt and mineral springs, and water wells containing combustible gases and/or saline water in northeastern Pennsylvania were gathered from a detailed review of over a dozen documents dating back to the early 1800s. Locations of historical accounts are shown in Figure S4 and detailed in Table S7.

Figure S4. Locations of Historical Gas Wells, Gas Fields, Salt/ Mineral Springs, and Shows of Saline Water and Combustible Gases in Water Wells.

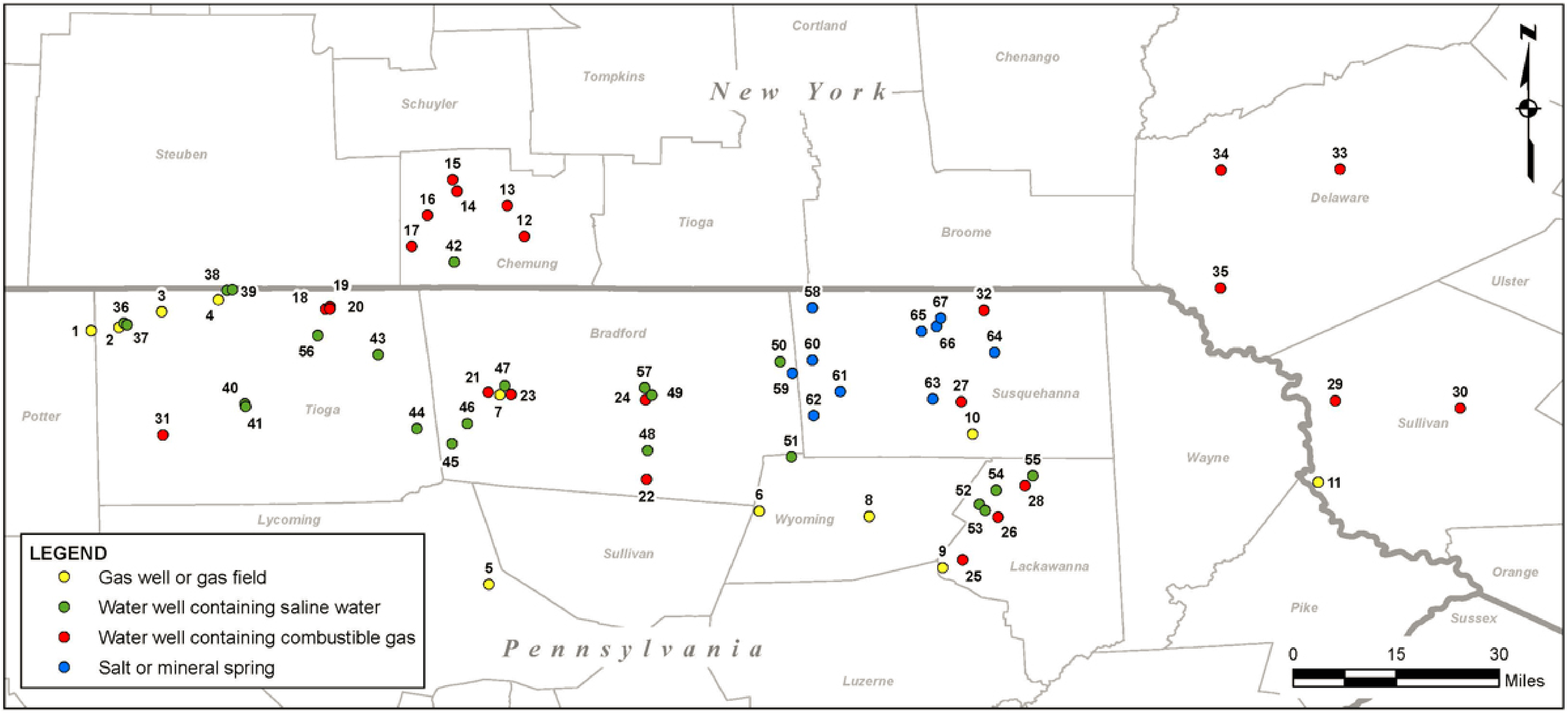

Table S7. Details of Historical References to Locations of Gas Wells/ Fields (yellow), Water Wells Containing Combustible Gases (red), Water Wells Containing Saline Water (green), and Salt/ Mineral Springs (blue).

Map Number	Color	Date	Township, County	Detailed Location	Historical Description	Total Depth (ft) of Gas or Water Well	Formation	Reference
1	Yellow	1905	Tioga Co.	Potter Brook Gas Field, Disc. 1905	Produces natural gas from Lock Haven Formation	1500 est.	Lock Haven	Carter and Harper 2002
2	Yellow	1895	Tioga Co.	Westfield Gas Field, Disc. 1895	Produces natural gas from Lock Haven Formation	1500 est.	Lock Haven	Carter and Harper 2002
3	Yellow	1917	Tioga Co.	Knoxville Gas Field, Disc. 1917	Produces natural gas from Lock Haven Formation	1500 est.	Lock Haven	Carter and Harper 2002
4	Yellow	1895	Tioga Co.	Elkland Gas Field, Disc. 1895	Produces natural gas from Lock Haven Formation	1500 est.	Lock Haven	Carter and Harper 2002
5	Yellow	1969	Lycoming Co.	Shrewsbury Gas Field, Disc. 1969	Produces natural gas from Lock Haven Formation	2500 est.	Lock Haven	Carter and Harper 2002
6	Yellow	1965	Wyoming Co.	Lovelton Gas Field, Disc. 1965	Produces natural gas from Lock Haven Formation	2000 est.	Lock Haven	Carter and Harper 2002
7	Yellow	1987	Bradford Co.	Brace Creek Oilfield, Disc. 1987	Produces natural gas from Lock Haven Formation	2000 est.	Lock Haven	Carter and Harper 2002
8	Yellow	1881 - 1882	Wyoming Co.	Gas well, Wyoming County (1922)	Gas well drilled to depth of 2089 ft.	2089	Lock Haven	Ashley and Robinson 1922
9	Yellow	1956	Lackawanna Co.	Harveys Lake Gas Field, Disc. 1956	Produces natural gas from Lock Haven Formation, gas shows in Catskill Formation	2800	Lock Haven	Carter and Harper 2002
10	Yellow	1957	Susquehanna Co.	Gas well, Susquehanna County	Third gas well drilled in Susquehanna County contains gas show at 522 ft.	2583	Catskill	PA DEP well record
11	Yellow	Pre-1922	Sullivan Co., New York	Gas well, Sullivan County (1922)	Gas well encountered gas at depth of 800 ft.	800	Lock Haven	Ashley and Robinson 1922

Map Number	Color	Date	Township, County	Detailed Location	Historical Description	Total Depth (ft) of Gas or Water Well	Formation	Reference
12	Red	1959	Chemung Co.	Well CM #281 (1959)	Water well contains hydrogen sulfide and natural gas	80	Unknown	Wetterhall 1959
13	Red	1959	Chemung Co.	Well CM #52 (1959)	Water well contains salty water and natural gas	172	Unknown	Wetterhall 1959
14	Red	1959	Chemung Co.	Well CM #174 (1959)	Water well contains hydrogen sulfide and natural gas	80	Unknown	Wetterhall 1959
15	Red	1959	Chemung Co.	Well CM #579 (1959)	Water well contains ignitable gas	96	Unknown	Wetterhall 1959
16	Red	1959	Chemung Co.	Well CM #237 (1959)	Water well contains natural gas	100	Unknown	Wetterhall 1959
17	Red	1959	Chemung Co.	Well CM #89 (1959)	Water well contains natural gas	128	Unknown	Wetterhall 1959
18	Red	2007	Lawrence, Tioga Co.	Well CM #T1589 (12) (2007)	Bedrock water well containing gases comprised of 8-9% combustible gas	83	Lock Haven	Breen, et. al. 2007
19	Red	2007	Lawrence, Tioga Co.	Well CM #T1586 (2007)	Bedrock water well containing gases comprised of 1-1.3% combustible gas	135	Lock Haven	Breen, et. al. 2007
20	Red	2007	Lawrence, Tioga Co.	Well CM #T1580 (21) (2007)	Bedrock water well containing gases comprised of 17-20% combustible gas	220	Lock Haven	Breen, et. al. 2007
21	Red	1939	Troy, Bradford Co.	Well #37 (1939)	Water well contains fresh water above 180 ft., salty water and natural gas below 180 ft.	412	Lock Haven	Lohman 1939
22	Red	1939	Albany, Bradford Co.	Well #87 (1939)	Water well contains hydrogen sulfide and inflammable gas	138	Lock Haven	Lohman 1939
23	Red	1939	Troy, Bradford Co.	Well #35 (1939)	Water well contains salty water, hydrogen sulfide, and natural gas that ignites	800	Lock Haven	Lohman 1939
24	Red	1939	Wysox, Bradford Co.	Well #58 (1939)	Water well contains slightly salty water and inflammable gas	600	Lock Haven	Lohman 1939
25	Red	1975	Lackawanna Co.	Well #LK 38 (1975)	Methane present in water well	260	Catskill	Hollowell and Koester 1975

Map Number	Color	Date	Township, County	Detailed Location	Historical Description	Total Depth (ft) of Gas or Water Well	Formation	Reference
26	Red	1975	Lackawanna Co.	Well #LK 234/238 (1975)	Methane present in water well	300/16	Catskill/Drift	Hollowell and Koester 1975
27	Red	1899	Susquehanna Co.	Boring in Brooklyn Township	Boring in the Brooklyn Township struck gas	Unknown	Unknown	Susquehanna Historical Society Website 2010
28	Red	1975	Lackawanna Co.	Well #LK 150 (1975)	Natural gas present in water well, 67.7% methane	175	Catskill	Hollowell and Koester 1975
29	Red	1961	Sullivan Co.	Well #D289 (1961)	Water well driller penetrated pocket of natural gas	208	Lock Haven	Soren 1961
30	Red	1961	Sullivan Co.	Well #SV109 (1961)	Water well contains methane gas @ 460 ft, salt water @ 462 ft.; two explosions during drilling	462	Lock Haven	Soren 1961
31	Red	1939	Shippen, Tioga Co.	Well #499 (1939)	Water well contains salty water and some natural gas	411	Lock Haven	Lohman 1939
32	Red	1908	Hallstead, Susquehanna Co.	Oil Well, Hallstead Township	Oil well drilled to 400 ft, encountering a small amount of natural gas and a vein of salt water	400	Lock Haven	Susquehanna Historical Society Website 2010
33	Red	1963	Delaware Co.	Well #D32 (1963)	Water reported to contain flammable gas, hydrogen sulfide, and significant sediment	505	Catskill	Soren 1963
34	Red	1963	Delaware Co.	Well #D102 (1963)	Well penetrated small pocket of natural gas; see drillers log	420	Catskill	Soren 1963
35	Red	1963	Delaware Co.	Well #D353 (1963)	Water well contains flammable gas	296	Catskill	Soren 1963
36	Green	1939	Westfield, Tioga Co.	Well #456 (1939)	Water well contains slightly salty water	~200	Lock Haven	Lohman 1939
37	Green	1939	Westfield, Tioga Co.	Well #458 (1939)	Water well contains salty water	250	Lock Haven	Lohman 1939
38	Green	1939	Elkland, Tioga Co.	Water Well #470 (1939)	Water well contains salty water	175	Lock Haven	Lohman 1939

Map Number	Color	Date	Township, County	Detailed Location	Historical Description	Total Depth (ft) of Gas or Water Well	Formation	Reference
39	Green	1939	Elkland, Tioga Co.	Water Well #475 (1939)	Water well contains very salty water	107	Lock Haven	Lohman 1939
40	Green	1939	Wellsboro, Tioga Co.	Well #507 (1939)	Water well contains salty water	215	Lock Haven	Lohman 1939
41	Green	1939	Wellsboro, Tioga Co.	Well #506 (1939)	Water well contains salty water	580	Lock Haven	Lohman 1939
42	Green	1959	Chemung Co.	Well CM #489 (1959)	Water well contains salty water	159	Lock Haven	Wetterhall 1959
43	Green	1939	Rutland, Tioga Co.	Well #483 (1939)	Water well contains manganese, hydrogen sulfide, significant iron, and slightly salty water	102	Glacial Drift	Lohman 1939
44	Green	1939	Ward, Tioga Co.	Well #519 (1939)	Water well contains salty water @ 1900 ft.	3600	Lock Haven	Lohman 1939
45	Green	1939	Canton, Bradford Co.	Well #78 (1939)	Water well contains hydrogen sulfide	201	Lock Haven	Lohman 1939
46	Green	1939	Alba, Bradford Co.	Well #77 (1939)	Water well contains hydrogen sulfide	186	Lock Haven	Lohman 1939
47	Green	1939	Troy, Bradford Co.	Well #36 (1939)	Water well contains fresh water above 180 ft., salty water below 200 ft.	207	Lock Haven	Lohman 1939
48	Green	1939	Monroe, Bradford Co.	Well #69 (1939)	Water well contains hydrogen sulfide	108	Unknown	Lohman 1939
49	Green	1939	Towanda, Bradford Co.	Well #49 (1939)	Water well contains salty water below 288 ft.	720	Lock Haven	Lohman 1939
50	Green	1939	Pike, Bradford Co.	Well #63 (1939)	Water well contains hard water (94 ppm) and TDS (140 ppm)	138	Lock Haven	Lohman 1939
51	Green	1937	Braintrim, Wyoming Co.	Well #210 (1937)	Water well contains salty water and hydrogen sulfide	122	Lock Haven	Lohman 1937
52	Green	1935	Lackawanna Co.	Well #LK 222 (1975)	Water well contains salty water	381	Catskill	Hollowell and Koester 1975

Map Number	Color	Date	Township, County	Detailed Location	Historical Description	Total Depth (ft) of Gas or Water Well	Formation	Reference
53	Green	1935	Lackawanna Co.	Well #LK 244 (1975)	Water well contains salty water	565	Catskill	Hollowell and Koester 1975
54	Green	1975	Lackawanna Co.	Well #LK 235 (1975)	Water well contains salty water	300	Catskill	Hollowell and Koester 1975
55	Green	1975	Lackawanna Co.	Well #LK 241 (1975)	Water well contains salty water	323	Catskill	Hollowell and Koester 1975
56	Green	1939	Tioga, Tioga Co.	Well #488 (1939)	Water well contains iron & salt in water	410	Lock Haven	Lohman 1939
57	Green	1939	N. Towanda, Bradford Co.	Well #46 (1939)	Water well contains hard water (196 ppm) and TDS (233 ppm)	77	Glacial Drift	Lohman 1939
58	Blue	Pre-1937	Middletown, Susquehanna Co.	Salt Spring	Salt springs reported in Apolacon, Auburn and Franklin Townships that were used by early settlers as sources of salt	Unknown	Lock Haven	Blackman 1873; Stocker 1887
59	Blue	1833	Bradford Co.	Salt Manufacturing Company, Salt Spring Susquehanna/ Bradford County	A salt manufacturing company was established in Susquehanna county, at a salt spring on the dividing line with Bradford county.	Unknown	Unknown	Hazard 1833
60	Blue	1828	Franklin	Water well drilled to 500 ft.	Water well was drilled to a total depth of 500 ft in bedrock and produced about one bushel of salt from 50 gallons of water	500	Lock Haven	Blackman 1873; Stocker 1887
61	Blue	1860's -1870's	Rush, Susquehanna Co.	Mineral Spring, Rush	Near Rush in western Susquehanna County, a cold water mineral spring with reported medicinal qualities was frequented in the mid-1860-1870s	Unknown	Unknown	Blackman 1873

Map Number	Color	Date	Township, County	Detailed Location	Historical Description	Total Depth (ft) of Gas or Water Well	Formation	Reference
62	Blue	1871	Susquehanna Co.	Riley Creek Mineral Spring, 1871	Mineral springs were reported to occur in 1871 or earlier along Riley Creek on the John Riley farm	Unknown	Catskill	Blackman 1873
63	Blue	1871	Dimock, Susquehanna Co.	Mineral Spring, Rosencrantz Farm	Mineral spring contains sulfur and iron	Unknown	Catskill	Blackman 1873
64	Blue	1830	New Milford, Susquehanna Co.	Salt Lick Creek	Salt licks found along Salt Lick Creek are likely sourced to seepage from salt water springs	Unknown	Lock Haven	Hazard 1830
65	Blue	1937	Susquehanna Co.	Well #4 (1937)	A well in Lawsville Center (well 4) obtains very salty water from the Chemung formation	Unknown	Lock Haven	Lohman 1937
66	Blue	Late 1700's	Franklin	Salt Springs State Park	Salt spring present pre-1800's, contains bubbling methane gas	Hundreds of feet	Lock Haven	Blackman 1873; Stocker 1887
67	Blue	1830	Liberty, Susquehanna Co.	Salt Spring, Lawsville	Salt spring located in Snake Creek near Lawsville	Unknown	Unknown	Hazard 1830

SUPPORTING INFORMATION REFERENCES

Deutsch, W.J. 1997. Groundwater Geochemistry: Fundamentals and Applications to Contamination. CRC Press LLC.

Isotech 2011. Collection of Ground Water Samples from Domestic and Municipal Water Wells for Dissolved Gas Analysis. Isotech Laboratories Inc., Accessed October 2011 at: http://www.isotechlabs.com/customersupport/samplingprocedures/DGbottle.pdf.

Marcellus Shale Coalition (MSC) 2010. Summary of Aqueous Predrill Constituents. Generated by Marcellus Shale Coalition Workgroup, 2010.

Pennsylvania Department of Conservation and Natural Resources (PA DCNR) 2011. Customized WIS Query Request for Locations of Oil and Gas Wells Drilled in Susquehanna County, Pennsylvania since 1800 to Present-day (October 26, 2011). Pennsylvania Department of Conservation and Natural Resources, Topographic and Geological Survey, Issued October 2011.

Pennsylvania Department of Environmental Protection (PA DEP) 2012. Pennsylvania Department of Environmental Protection, Act 13 of 2012, House Bill no. 1950, issued 14 February 2012. Available online at: http://www.ctbpls.com/www/PA/11R/PDF/PA11RHB01950CC1.pdf.

United States Geologic Survey, National Hydrography Dataset (NHD) 2011. Available online at: http://nhd.usgs.gov/.

U.S. EPA 2011. ProUCL Version 4.1.01, a Statistical Software, National Exposure Research Lab, EPA, Las Vegas Nevada. ProUCL Version 4.1.01 can be freely downloaded from the EPA website: http://www.epa.gov/nerlesd1/tsc/tsc.htm.

Strapoc, D., Schimmelmann, A., and Mastalerz, M. 2005. Carbon isotopic fractionation of CH_4 and CO_2 during canister desorption of coal. *Organic Geochemistry* 37**:** 152-164.

Yee, D., Seidle, J.P., and Hanson, W.B. 1993. Chapter 9: Gas Sorption on Coal and Measurement of Gas Content. Chapter in AAPG Studies in Geology #38: Hydrocarbons from Coal, edited by B.E. Law and D.D. Rice. Published by the American Association of Petroleum Geologists, ISBN 0-89181-046-3.

References for Table S1

Pennsylvania Groundwater Information System (PaGWIS) 2011. PaGWIS Access Relational Database. Accessed July 2011 at: http://www.dcnr.state.pa.us/topogeo/groundwater/PaGWIS/help.aspx.

References for Table S2

Pennsylvania Department of Environmental Protection (PA DEP) 2010a. Isotopic Sample Data Sheet: 10/19/2010, Carter Road Gas Migration, Dimock Twp./ Susquehanna County, Document provided in response to Freedom of Information Act (FOIA) request in August 2011.

Pennsylvania Department of Environmental Protection (PA DEP) 2010b. Pennsylvania DEP Internal Memo Re: Stray Gas Investigation, Dimock Township, PA, Susquehanna County, produced on 26 September 2010 by Fred Baldassare, former Geologist at the Pennsylvania Dept. of Environmental Protection, Document provided in response to Freedom of Information Act (FOIA) request in August 2011.

References for Tables S5 and S6

Osborn S.G., Vengosh A., Warner N.R., and Jackson R.B. 2011. Methane Contamination of Drinking Water Accompanying Gas-Well Drilling and Hydraulic Fracturing, *Proceedings of the National Academy of Sciences* 108: 8172-8176.

U.S. EPA 2012. EPA in Pennsylvania: Dimock, PA Activities, Accessed September 2012 at: http://www.epa.gov/aboutepa/states/pa.html, Isotopic Data Accessed at: http://www.epaosc.org/site/doc_list.aspx?site_id=7555.

References for Table S7

Ashley, G.H., and Robinson, J.F. 1992. The oil and gas fields of Pennsylvania: The Pennsylvania Geological Survey 4th Series, v. 1, 44 p.

Blackman, E.C. 1873. History of Susquehanna County, Pennsylvania: Claxton, Remsen and Haffelfinger, Philadelphia, PA, 685 p.

Breen, K.J., Revesz, K., Baldassare, F.J., and McAuley, S.D. 2007. Natural gases in ground water near Tioga Junction, Tioga County, North-Central Pennsylvania—Occurrence and use of isotopes to determine origins, 2005: U.S. Geological Survey Scientific Investigations Report Series 2007-5085, 65 p.

Carter, K.M., and Harper, J.A. 2002. Oil and gas prospects in northeastern Pennsylvania in: Inners, J.D. and Fleeger, G.M., eds., 2002, From Tunkhannock to Starrucca: bluestone, glacial lakes, and great bridges in the "Endless Mountains" of northeastern Pennsylvania: Guidebook, 67th Annual Field Conference of Pennsylvania Geologists, Tunkhannock, PA, p. 15-31.

Hazard, S. 1830. Hazard's Registry of Pennsylvania, Devoted to the Preservation of Facts and Documents, and Every Kind of Useful Information, Volume 5. Published by WM. F. Geddes, Philadelphia.

Hazard, S. 1833. Hazard's Registry of Pennsylvania, Devoted to the Preservation of Facts and Documents, and Every Kind of Useful Information, Volume 11. Published by WM. F. Geddes, Philadelphia.

Hollowell, J.R., and Koester, H.E. 1975. Ground-water resources of Lackawanna County, Pennsylvania: Pennsylvania Geologic Survey, Fourth Series, Water Resource Report 41, 106 p.

Lohman, S.W. 1937. Ground water in northeastern Pennsylvania: Progress Report W4, Pennsylvania Geological Survey Fourth Series.

Lohman, S.W. 1939. Groundwater in North-Central Pennsylvania: Commonwealth of Pennsylvania, Topographic and Geologic Survey Bulletin W4.

Pennsylvania Department of Environmental Protection (PA DEP) 2012. Water Well Record Morgan No. 1, API No. 3711520003000, drilled 1957, 3 p. Pennsylvania Department of Environmental Protection PA IRIS website. Accessed at: http://www.dcnr.state.pa.us/topogeo/econresource/oilandgas/pa_iris_home/index.htm.

Soren, J. 1961. The ground-water resources of Chemung County, New York: U.S. Geological Survey Bulletin GW-46, prepared in cooperation with the New York Water Resources Commission, 66 p.

Soren, J. 1963. The ground-water resources of Sullivan County, New York: U.S. Geological Survey Bulletin GW-46, prepared in cooperation with the New York State Water Resources Commission, 58 p.

Stocker, R. M. 1887. The Centennial History of Susquehanna County, Pennsylvania. Published by R.T. Peck, Philadelphia.

Susquehanna Historical Society Website 2010. Accessed July 19, 2010 at: www.susqcohistsoc.org.

Wetterhall, W.S. 1959. The ground-water resources of Chemung County, New York: State of New York Department of Conservation Water Power and Control Commission, U.S. Geological Survey Bulletin GW-40, 58 p.
