# Supplementary material for: Evaluation of Methane Sources in Groundwater in Northeastern Pennsylvania
Source: Ground Water. 2013 Apr 5;51(3):333–49. doi: 10.1111/gwat.12056 (PMC3746116; doi:10.1111/gwat.12056)
Supplement: Supplementary file 2 [file gwat0051-0333-SD2.pdf]

Table S.8. Groundwater Quality Data for 1701 "Pre-Drill" Water Well Samples from Susquehanna County, Pennsylvania

Water Well ID	Sample Date	Sampling Port	Topo-graphic Location	Gas Extraction Area (Within 1 km of Active Gas Well)	Groundwater Type (If Determined)	Alkalinity (mg/L)	Aluminum (mg/L)	Arsenic (mg/L)	Barium (mg/L)	Boron (mg/L)	Bromide (mg/L)	Cadmium (mg/L)	Calcium (mg/L)	Chloride (mg/L)	Chromium (mg/L)	Conductivity - Field (μs/cm)
1	6/3/2011		Valley		Calcium-Bicarbonate	44	<0.03	<0.001	0.047			<0.001	15	2	<0.002	
2	5/27/2010	Pressure Tank	Valley					0.018	1.28							364
3	6/29/2011		Upland			110	<0.03	<0.001				<0.001	29		<0.002	0.072
4	6/29/2011		Upland		Calcium-Sodium-Bicarbonate	50	< .03	< .001	0.066		< .02	< .001	6.2	1.7	< .002	
5	6/17/2010	Outside Spigot	Upland	Yes												379
6	6/17/2010	Kitchen Sink	Valley													153.5
7	6/17/2010	Kitchen Sink	Upland													142.7
8	4/10/2010	Pressure Tank	Upland				< .1	< .003	0.105	< .1				12.2		105.7
9	4/10/2010	Basement At Pressure Tank	Upland				< .1	< .003	0.131	< .1				4.41		201
10	4/10/2010	Pressure Tank	Valley				< .1	< .003	0.13	< .1				5.2		153.5
11	4/11/2010	Outside Hydrant Directly From Well	Upland				< .1	< .003	0.097	< .1				6.41		151.8
12	4/11/2010		Valley				< .1	< .003	0.058	< .1				6.36		
13	4/11/2010	Kitchen Sink	Valley				< .1	0.038	0.3	< .1				5.93		257
14	4/11/2010	Kitchen Sink	Upland	Yes			< .1	< .003	0.154	< .1				5.84		190.4
15	4/11/2010	Pressures Tank	Upland				< .1	< .003	0.146	< .1				42.6		284
16	4/11/2010	Kitchen Sink	Valley				< .1	< .003	1.62	0.168				9.14		255
17	4/11/2010	Kitchen Sink	Upland				< .1	< .003	0.4	< .1				6.64		
18	4/11/2010	Outside Hydrant Direct From Well	Upland				< .1	< .003	0.614	< .1				3.72		
19	4/15/2010	Outside Hydrant	Upland				< .1	< .003	0.165	< .1				5.04		
20	4/15/2010	Kitchen Sink	Upland				< .1	< .003	0.264	< .1				7.73		
21	4/15/2010	Outside Hydrant	Valley				< .1	< .003	0.064	< .1				20.8		
22	4/15/2010	Pressure Tank	Valley				< .1	< .003	0.105	< .1				7.94		
23	4/19/2010	Laundry Room Sink	Upland				< .1	< .003	0.069	< .1				7.3		

Table S.8. Groundwater Quality Data for 1701 "Pre-Drill" Water Well Samples from Susquehanna County, Pennsylvania

Water Well ID	Sample Date	Sampling Port	Topo-graphic Location	Gas Extraction Area (Within 1 km of Active Gas Well)	Groundwater Type (If Determined)	Alkalinity (mg/L)	Aluminum (mg/L)	Arsenic (mg/L)	Barium (mg/L)	Boron (mg/L)	Bromide (mg/L)	Cadmium (mg/L)	Calcium (mg/L)	Chloride (mg/L)	Chromium (mg/L)	Conductivity - Field (µs/cm)
24	4/19/2010	Hydrant At Well Head	Valley				< .1	< .003	0.11	< .1				8.35		
25	4/19/2010	Diversion Ditch	Upland	Yes			0.159		< .025					4.96		
26	4/22/2010	Kitchen Sink	Valley				0.1	< .003	< .191	< .1				47.7		
27	4/22/2010	Kitchen Sink	Upland	Yes			< .1	< .003	0.051	< .1				7.48		
28	4/22/2010	Basement Sink	Upland				< .1	< .003	0.196	< .1				8.65		
29	6/24/2010	Pond	Upland													84.4
30	6/24/2010	Pressure Tank	Upland													209
31	5/14/2011		Upland		Calcium-Bicarbonate-Chloride	92	< .03	< .001	0.099		< .02	< .001	41	73	< .002	0.351
32	6/24/2010	Kitchen Sink	Valley													273
33	6/24/2010	Outside Spigot	Upland				< .1	< .003	0.105	< .1				4.33		174.1
34	4/25/2010	Bathroom Faucet	Upland	Yes			< .1	< .003	0.114	< .1				< 2		143.7
35	4/25/2010		Upland	Yes			< .1		0.191					19.1		
36	4/24/2010	Outside Spigot	Valley				< .1	< .003	0.22	< .1				153		
37	4/24/2010	Kitchen Sink	Upland	Yes			< .1	< .003	0.053	< .1				7.61		87.1
38	4/24/2010		Upland				< .1	< .003	0.084	< .1				8.26		
39	4/24/2010	Kitchen Bar Sink	Upland				< .1	< .003	0.139	< .1				38.5		
40	4/24/2010	Outside Spigot	Upland				< .1	< .003	0.282	< .1				2.43		
41	4/24/2010	Outside Spigot	Upland				< .1	< .003	0.084	< .1				2.37		
42	4/29/2010	Outside Spigot	Upland				< .1	< .003	0.174	< .1				2		
43	4/29/2010	Spring Culvert	Upland				< .1	< .003	< .029	< .1				< 2		
44	4/29/2010	Basement At Pressure Tank	Valley				< .1	< .003	0.091	< .1				2.53		
45	7/2/2010	Kitchen Sink	Valley													172
46	7/2/2010	Bathroom Sink	Valley													194
47	7/2/2010	Kitchen Sink	Valley													238
48	7/2/2010	Kitchen Sink	Valley													248
49	7/2/2010	Kitchen Sink	Upland													231

Table S.8. Groundwater Quality Data for 1701 "Pre-Drill" Water Well Samples from Susquehanna County, Pennsylvania

Water Well ID	Sample Date	Sampling Port	Topo-graphic Location	Gas Extraction Area (Within 1 km of Active Gas Well)	Groundwater Type (If Determined)	Alkalinity (mg/L)	Aluminum (mg/L)	Arsenic (mg/L)	Barium (mg/L)	Boron (mg/L)	Bromide (mg/L)	Cadmium (mg/L)	Calcium (mg/L)	Chloride (mg/L)	Chromium (mg/L)	Conductivity - Field (µs/cm)
50	7/2/2010	Kitchen Sink	Upland													203
51	7/2/2010	Kitchen Sink	Valley													239
52	7/6/2010	Kitchen Sink	Upland				< .1	< .003	0.078	< .1				3.54		242
53	7/6/2010	Garage	Valley				< .1	0.006	0.41	< .1				4.05		263
54	7/6/2010	Kitchen Sink	Valley				< .1	< .003	0.104	< .1				3.1		127
55	7/6/2010	Kitchen Sink	Upland				< .1	< .003	0.235	0.159				5.4		210
56	7/6/2010	Kitchen Sink	Valley				< .1	< .003	1.64	0.103				52.6		303
57	7/6/2010		Valley				< .1	< .003	0.768	< .1				3.01		
58	7/6/2010	Kitchen Sink	Valley				< .1	< .003	0.129	< .1				5.08		194
59	7/6/2010	Basement At Pressure Tank	Valley				< .1	< .003	0.094	< .1				6.27		131
60	7/6/2010		Upland				< .1	< .003	0.053	< .1				29.5		
61	7/7/2010	Kitchen Sink	Valley				< .1	< .003	0.103	< .1				17.8		263
62	7/7/2010	Spring Head	Valley				< .1	< .003	0.043	< .1				4.26		83
63	7/7/2010	Kitchen Sink	Valley				< .1	< .003	0.074	< .1						197
64	7/7/2010	Kitchen Sink	Valley				< .1	0.005	0.51	< .1				4.59		241
65	7/7/2010	Kitchen Sink	Valley				< .1	< .003	0.062	< .1				2.83		146
66	7/7/2010	Basement Pressure Tank	Valley				< .1	< .003	0.165	< .1				3.58		141
67	7/7/2010	Kitchen Sink	Valley				< .1	< .003	0.52	< .1				18		191
68	7/8/2010	Basement Sink	Valley				< .1	< .003	0.164	< .1				7.64		249
69	7/8/2010	Kitchen Sink	Upland				< .1	< .003	0.149	< .1				3.22		207
70	7/8/2010	Kitchen Sink	Valley				< .1	< .003	0.071	< .1				8.52		288
71	7/9/2010	Kitchen Sink	Valley				< .1	< .003	0.104	< .1				9.32		300
72	7/9/2010	Kitchen Sink - Debonis Property	Valley				< .1	< .003	0.172	< .1				38.8		249
73	7/7/2010	Kitchen Sink	Valley				< .1	< .003	0.287	< .1				3.9		216
74	7/7/2010	Outside Garage Spigot	Valley				< .1	< .003	0.063	< .1				3.14		153

Table S.8. Groundwater Quality Data for 1701 "Pre-Drill" Water Well Samples from Susquehanna County, Pennsylvania

Water Well ID	Sample Date	Sampling Port	Topo-graphic Location	Gas Extraction Area (Within 1 km of Active Gas Well)	Groundwater Type (If Determined)	Alkalinity (mg/L)	Aluminum (mg/L)	Arsenic (mg/L)	Barium (mg/L)	Boron (mg/L)	Bromide (mg/L)	Cadmium (mg/L)	Calcium (mg/L)	Chloride (mg/L)	Chromium (mg/L)	Conductivity - Field (µs/cm)
75	7/8/2010	Kitchen Sink	Valley				< .1	< .003	< .025	< .1				7.01		
76	7/7/2010	Kitchen Sink	Valley				< .1	< .003	0.117	< .1				4.21		181
77	7/7/2010	Kitchen Sink	Valley				< .1	< .003	0.054	< .1				3.32		113
78	7/8/2010	Kitchen Sink	Valley				< .1	< .003	0.887	< .1				3.65		159
79	7/8/2010	Kitchen Sink	Upland				< .1	< .003	0.176	< .1				8.49		229
80	7/8/2010	Kitchen Sink	Valley				< .1	< .003	0.132	< .1				14.7		282
81	7/8/2010	Kitchen Sink	Valley				< .1	< .003	0.152	< .1				46.8		187
82	7/9/2010		Valley	Yes			< .1	< .003	0.174	< .1				42.8		
83	7/9/2010	Kitchen Sink	Valley				< .1	< .003	0.197	< .1				168		840
84	7/9/2010	Kitchen Sink	Valley				< .1	< .003	0.132	< .1				6.8		234
85	7/9/2010	Kitchen Sink	Upland				< .1	< .003	0.083	< .1				5.58		213
86	7/9/2010	Kitchen Sink	Valley				< .1	0.004	0.74	0.106				2.92		269
87	7/9/2010		Valley				< .1	< .003	0.058	< .1				2.97		
88	7/10/2010		Valley				< .1	< .003	3.83	< .1				194		
89	7/8/2010	Pressure Tank	Upland				< .1	< .003	0.096	< .1				3.35		146
90	5/20/2010	Kitchen Sink	Valley				< .1	< .003	0.047	< .1				2.43		414
91	7/11/2010	Kitchen Sink	Valley				< .1	< .003	0.185	< .1				9.99		327
92	7/11/2010	Barn Hose	Valley				<0.1	<0.003	0.041	<0.1				14.8		
93	7/8/2010	Kitchen Sink	Upland	Yes			< .1	< .003	0.179	< .1				11.5		228
94	4/8/2009		Upland				<0.05		0.113					3.53		
95	7/9/2010		Valley				< .1	< .003	0.187	< .1				4.59		
96	7/10/2010	Kitchen Sink	Valley				< .1	< .003	0.145	< .1				6.49		296
97	7/10/2010	Kitchen Sink	Upland				< .1	0.004	0.028	< .1				4.7		109
98	7/12/2010	Artesian Well	Valley				< .1	< .003	0.088	< .1				15.4		
99	7/12/2010		Valley				< .1	< .003	1.54	0.141				49.6		
100	7/12/2010	Kitchen Sink	Valley				< .1	0.003	0.693	< .1				19.3		278
101	7/12/2010		Valley				< .1	< .003	4.62	0.136				394		
102	7/12/2010		Valley				< .1	< .003	0.076	< .1				6.91		
103	7/11/2010	Kitchen Sink	Valley	Yes			< .1	< .003	1.5	< .1				18.5		280

Table S.8. Groundwater Quality Data for 1701 "Pre-Drill" Water Well Samples from Susquehanna County, Pennsylvania

Water Well ID	Sample Date	Sampling Port	Topo-graphic Location	Gas Extraction Area (Within 1 km of Active Gas Well)	Groundwater Type (If Determined)	Alkalinity (mg/L)	Aluminum (mg/L)	Arsenic (mg/L)	Barium (mg/L)	Boron (mg/L)	Bromide (mg/L)	Cadmium (mg/L)	Calcium (mg/L)	Chloride (mg/L)	Chromium (mg/L)	Conductivity - Field (µs/cm)
104	7/12/2010	Kitchen Sink	Valley				< .1	< .003	0.08	< .1				3.26		
105	7/13/2010		Upland				< .1	< .003	0.169	< .1				24.1		
106	7/12/2010	Kitchen Sink	Valley				< .1	< .003	0.517	0.239				17.2		
107	7/17/2010	Kitchen Sink	Valley				< .1	< .003	0.155	< .1				5.77		187
108	7/17/2010	Kitchen Sink	Valley				< .1	< .003	0.086	< .1				5.3		269
109	7/17/2010	Kitchen Sink	Valley				< .1	< .003	0.051	< .1				2.98		119
110	7/11/2010		Upland				< .1	< .003	0.092	< .1				3.62		
111	7/13/2010	Kitchen Sink	Valley				< .1	< .003	0.069	< .1				2.39		
112	7/13/2010	Kitchen Sink	Valley				< .1	0.003	0.381	0.108				11.1		
113	7/12/2010		Valley				< .1	< .003	0.189	< .1				23.5		
114	7/25/2011	Kitchen Sink	Upland													394
115	8/17/2011	Bathroom Sink	Valley													332
116	7/5/2011		Valley													228
117	3/12/2009	Barn	Valley				< .05		0.721					11.7		
118	2/20/2009	Basement	Upland				< .05		0.112					12		
119	7/1/2010		Valley				< .1	< .003	0.041	< .1				4.43		
120	7/1/2010		Upland				< .1	0.005	0.241	< .1				7.21		
121	2/20/2009		Upland				< .05		0.136					< 2		
122	8/3/2010	Hand Dug Well	Upland				< .1	< .003	0.201	< .1				6.99		307
123	2/20/2009	Basement	Valley				< .05		0.05					2.33		
124	8/7/2010	Kitchen Sink	Upland				< .1	< .003	1.36	< .1				16.6		332
125	8/7/2010	Hand Dug Well	Upland				0.147	0.004	0.044	< .1				2.69		225
126	8/8/2010	Pressure Tank	Valley				<0.1	<0.003	0.134	<0.1				3.65		
127	8/8/2010	Kitchen Sink	Valley				< .1	< .003	< .025	0.105				28.1		393
128	8/8/2010	Bathroom Sink	Valley				< .1	< .003	< .025	< .1				6.56		357
129	8/8/2010	Kitchen Sink	Upland				< .1	< .003	0.051	< .1				3.81		136
130	7/30/2010	Bathroom Sink	Valley	Yes			< .1	< .003	0.462	< .1				3.44		184
131	8/13/2010	Outside Spigot	Upland				< .1	< .003	0.202	< .1				3.26		157
132	7/20/2010	Kitchen Sink	Valley				< .1	< .003	0.175	< .1				4.77		365
133	7/20/2010	Heefer Barn	Upland				< .1	< .003	0.113	< .1				8.75		317
134	8/11/2010	House #2	Valley				< .1	< .003	< .025	< .1				3.73		197
135	4/15/2009		Upland				< .1		0.042					5.76		

Table S.8. Groundwater Quality Data for 1701 "Pre-Drill" Water Well Samples from Susquehanna County, Pennsylvania

Water Well ID	Sample Date	Sampling Port	Topo-graphic Location	Gas Extraction Area (Within 1 km of Active Gas Well)	Groundwater Type (If Determined)	Alkalinity (mg/L)	Aluminum (mg/L)	Arsenic (mg/L)	Barium (mg/L)	Boron (mg/L)	Bromide (mg/L)	Cadmium (mg/L)	Calcium (mg/L)	Chloride (mg/L)	Chromium (mg/L)	Conductivity - Field (µs/cm)
136	8/11/2010	Main House Kitchen Sink	Valley				< .1	< .003	0.148	< .1				2.89		210
137	8/19/2010		Upland	Yes	Calcium-Bicarbonate	90	< .05	< .003	0.05			< .002	35.5	4.74	< .005	
138	8/19/2010	Barn Well Milkhouse Sink	Upland	Yes	Calcium-Bicarbonate	70	< .05	< .003	0.055			< .002	36.7	17.2	< .005	238
139	6/24/2009	Basement Pressure Tank	Valley				< .1		0.029					2.66		
140	8/19/2010	Pressure Tank	Upland		Calcium-Bicarbonate	110	< .034	0.005	0.143			< .002	32.9	2.51	< .005	224
141	8/19/2010	Kitchen Sink	Upland	Yes	Calcium-Bicarbonate	130	0.068	< .003	0.174			< .002	52.5	6.34	< .005	811
142	8/19/2010	Spigot Under Mobile Home	Upland	Yes	Calcium-Bicarbonate	105	< .05	< .003	0.11			< .002	41	9.39	< .005	229
143	8/20/2010	Laundry Room Sink	Upland		Calcium-Bicarbonate-Chloride	195	< .05	< .003	0.639			< .002	19.7	270	< .005	
144	6/24/2009	Well Head	Valley				< .1		< .025					< 2		
145	6/24/2009	Kitchen Sink	Valley				< .1		0.039					< 2		
146	8/26/2010	Outside Spigot	Upland		Calcium-Bicarbonate	100	< .05	< .003	0.151			< .002	42.9	39.2	< .005	377
147	7/29/2010		Valley				< .1	< .003	0.133	< .1				3.44		
148	7/29/2010	Kitchen Sink	Upland	Yes			< .1	0.005	0.121	< .1				4.36		238
149	8/28/2010	Pressure Tank	Valley		Calcium-Bicarbonate	180	< .05	< .003	< .122			< .002	< 41.6	4.22	< .005	389
150	8/28/2010	Outside Spigot	Valley		Calcium-Sodium-Bicarbonate	150	< .05	< .003	< .025			< .002	43.8	2.52	< .005	378
151	9/2/2010	Kitchen Sink	Upland		Calcium-Bicarbonate	140	< .05	< .002	0.11			< .002	49.3	< 2	< .005	292
152	6/13/2011	Kitchen Sink	Valley													152
153	9/10/2010	Kitchen Faucet	Valley		Sodium-Bicarbonate	35		< .003	0.153			< .002	15.5	4.05	< .01	300
154	7/20/2010	Tenant House Kitchen Sink	Upland				< .1	< .003	0.246	< .1				4.07		258
155	2/27/2010	Kitchen Sink	Upland				< .1		0.068					7.07		179.4
156	2/22/2010	Basement At Pressure Tank	Valley				< .1		1.18					2.46		

Table S.8. Groundwater Quality Data for 1701 "Pre-Drill" Water Well Samples from Susquehanna County, Pennsylvania

Water Well ID	Sample Date	Sampling Port	Topo-graphic Location	Gas Extraction Area (Within 1 km of Active Gas Well)	Groundwater Type (If Determined)	Alkalinity (mg/L)	Aluminum (mg/L)	Arsenic (mg/L)	Barium (mg/L)	Boron (mg/L)	Bromide (mg/L)	Cadmium (mg/L)	Calcium (mg/L)	Chloride (mg/L)	Chromium (mg/L)	Conductivity - Field (µs/cm)
157	9/17/2010	Kitchen Sink	Valley		Sodium-Bicarbonate	145		< .003	1.92			< .002	12.3	52.2	< .005	345
158	1/10/2010	Basement Pressure Tank	Upland				< .1		0.093					4.54		284
159	10/3/2010	Kitchen Sink	Valley	Yes	Calcium-Bicarbonate	160	< .05	< .003	0.086			< .002	49.2	26.2	< .005	
160	1/31/2011	Kitchen Sink	Upland		Calcium-Bicarbonate	70	< .01	< .001	0.13			< .0001	24	5.71	< .002	131
161	4/8/2009	Laundry Sink	Upland				< .05		0.135					2.15		
162	10/7/2010	Kitchen Sink	Upland				< .05	< .003	0.083			< .002	42.8		< .005	237
163	10/7/2010	Kitchen Sink	Valley	Yes			< .05	< .003	0.031			0.002	40		< .005	230
164	10/7/2010		Valley	Yes	Calcium-Bicarbonate	125	< .05	< .003	0.11			< .002	42.8	3.15	< .005	
165	4/8/2009	Boiler Room	Upland				< .05		0.172					8.19		
166	7/14/2011	Kitchen Sink	Upland													296
167	7/14/2011	Kitchen Sink	Upland													281
168	10/8/2010	Kitchen Faucet	Valley	Yes	Calcium-Bicarbonate	115	< .05	< .002	0.128			< .002	29.7	5.02	< .005	274
169	10/8/2010	Outside Spigot	Valley	Yes	Calcium-Bicarbonate	130	< .05	< .002	0.129			< .002	41.8	2.99	< .005	300
170	7/22/2010	Pressure Tank	Upland	Yes			< .1	< .003	0.971	< .1				3.76		243
171	4/8/2009	Career & Tech Bldg Pressure Tank	Upland				< .05		0.205					6.13		
172	4/8/2009	Boiler Room	Upland				< .05		0.127					7.17		
173	10/1/2010	Kitchen Sink	Valley	Yes	Sodium-Bicarbonate-Chloride	125	< .05	< .002	0.997			< .002	12.8	56.5	< .005	471
174	11/5/2009	Pressure Tank	Upland	Yes			< .1		0.117					8.16		
175	7/14/2011	Spigot On Side Of House	Valley													214
176	7/14/2011	Kitchen Sink	Valley													185
177	7/14/2011	Kitchen Sink	Valley													198
178	7/14/2011	Kitchen Sink	Upland													194
179	7/14/2011	Kitchen Sink	Upland													178

Table S.8. Groundwater Quality Data for 1701 "Pre-Drill" Water Well Samples from Susquehanna County, Pennsylvania

Water Well ID	Sample Date	Sampling Port	Topo-graphic Location	Gas Extraction Area (Within 1 km of Active Gas Well)	Groundwater Type (If Determined)	Alkalinity (mg/L)	Aluminum (mg/L)	Arsenic (mg/L)	Barium (mg/L)	Boron (mg/L)	Bromide (mg/L)	Cadmium (mg/L)	Calcium (mg/L)	Chloride (mg/L)	Chromium (mg/L)	Conductivity - Field (µs/cm)
180	10/10/2010	Kitchen Sink Rental Property	Valley	Yes	Calcium-Sodium-Bicarbonate-Chloride	95	< .05	< .002	0.027			< .002	57.3	94.3	< .005	616
181	4/15/2009	Pressure Tank	Valley	Yes			< .1		0.086					5.62		
182	10/10/2010	Kitchen Sink	Valley	Yes	Calcium-Bicarbonate	100	< .05	< .002	0.059			< .002	37.7	13.2	< .005	283
183	11/4/2010	Kitchen Sink	Valley		Calcium-Bicarbonate	80	< .01	< .001	0.1			< .0001	21	9.8	0.002	145
184	11/4/2010	Kitchen Sink	Upland		Calcium-Sodium-Bicarbonate	135	< .01	< .001	0.15			< .0001	23	4.54	< .002	193
185	3/12/2009	Basement Before Filter	Upland	Yes			< .05		0.45					179		
186	10/10/2010	Outside Spigot	Valley	Yes	Calcium-Bicarbonate	100	< .05	< .002	0.029			< .002	40.4	23.7	< .005	307
187	11/4/2010	Pressure Tank	Valley		Sodium-Bicarbonate	145	< .01	< .001	0.55			< .0001	17	19.7	< .002	
188	11/6/2010	Kitchen Sink	Valley	Yes	Calcium-Bicarbonate	130	< .01	< .001	0.04			< .0001	31	6.75	< .002	210
189	11/6/2010	Outside Spigot	Valley		Calcium-Bicarbonate	95	< .01	< .001	0.12			0.0001	24	17	< .002	180
190	11/7/2010	Kitchen Sink	Valley	Yes	Calcium-Bicarbonate-Chloride	155	< .01	< .001	0.03			< .0001	56	55.5	< .002	488
191	11/7/2010	Pressure Tank	Valley	Yes	Calcium-Bicarbonate-Chloride	100	< .01	< .001	0.043			< .0001	40	51.3	< .002	370
192	11/7/2010	Kitchen Sink	Valley	Yes	Calcium-Sodium-Bicarbonate	190	< .01	< .001	0.0088			< .0001	46	18.3	< .002	437
193	10/13/2010	Kitchen Faucet	Valley	Yes	Calcium-Bicarbonate	85	< .05	< .003	0.032			< .002	43.3	28.3	< .005	325
194	10/13/2010	Pressure Tank	Valley	Yes	Calcium-Bicarbonate	150	< .05	< .003	0.123			< .002	41.2	4.57	< .005	324
195	10/13/2010	Pressure Tank	Valley	Yes	Calcium-Sodium-Bicarbonate-Chloride	55	< .05	< .002	0.062			< .002	36.1	52.8	< .005	378
196	10/13/2010	Kitchen Sink	Valley	Yes	Calcium-Bicarbonate	95	< .05	< .003	0.071			< .002	40	15.3	< .005	288
197	10/11/2010	Kitchen Sink	Valley	Yes	Calcium-Bicarbonate-Chloride	20	< .05	< .002	0.225			< .002	13.9	19.2	< .005	127
198	10/18/2010	Bathroom Sink	Valley		Calcium-Bicarbonate-Chloride	75	<0.05	<0.003	0.099			<0.002	52.9	53	<0.005	
199	10/2/2010	Kitchen Sink Faucet	Upland		Calcium-Sodium-Bicarbonate	100	< .05	0.007	0.371			< .002	27.4	19	< .005	282

Table S.8. Groundwater Quality Data for 1701 "Pre-Drill" Water Well Samples from Susquehanna County, Pennsylvania

Water Well ID	Sample Date	Sampling Port	Topo-graphic Location	Gas Extraction Area (Within 1 km of Active Gas Well)	Groundwater Type (If Determined)	Alkalinity (mg/L)	Aluminum (mg/L)	Arsenic (mg/L)	Barium (mg/L)	Boron (mg/L)	Bromide (mg/L)	Cadmium (mg/L)	Calcium (mg/L)	Chloride (mg/L)	Chromium (mg/L)	Conductivity - Field (µs/cm)
200	10/2/2010	Pressure Tank	Valley		Calcium-Bicarbonate	125	< .05	< .003	0.065			< .002	36.5	3.9	< .005	278
201	10/2/2010	Bathroom Faucet	Valley		Calcium-Bicarbonate	120	< .05	< .003	0.095			< .002	32.8	4.47	< .005	
202	10/15/2010	Kitchen Sink	Valley	Yes	Calcium-Bicarbonate	95	< .05	< .002	0.039			< .002	35.2	18.8	< .005	282
203	4/19/2009		Valley	Yes			<0.05		<0.025					78.6		
204	4/4/2008		Upland				<0.05		<1					4.11		
205	10/19/2010	Kitchen Sink	Valley	Yes	Calcium-Sodium-Bicarbonate	150	< .05	0.004	0.313			< .002	29.1	4.45	< .005	327
206	10/19/2010	Kitchen Sink	Valley	Yes	Calcium-Bicarbonate	75	< .05	< .003	0.046			< .002	48.9	14.6	< .005	352
207	10/19/2010	Hand Dug Well	Valley	Yes	Calcium-Bicarbonate	35	0.174	< .003	0.028			< .002	12.2	2.13	< .005	97
208	10/20/2010	Kitchen Sink	Valley	Yes	Calcium-Bicarbonate	< 90	< .05	< .003	0.036			< .002	39.1	15.1	< .005	230
209	10/26/2010	Kitchen Sink	Valley	Yes	Calcium-Bicarbonate	115	< .05	< .003	< .025			< .002	40.4	19.3	< .005	302
210	10/26/2010	Outside Spigot	Valley		Calcium-Bicarbonate	155	< .05	< .003	< .025			< .002	48	2.26	< .005	337
211	7/14/2011	Kitchen Sink	Upland													105
212	8/1/2011	Kitchen Sink	Valley													191
213	10/26/2010		Valley	Yes	Calcium-Bicarbonate-Chloride	60	< .05	< .003	< .025			< .002	34.4	47.4	< .005	
214	10/28/2010	Kitchen Sink	Valley	Yes	Calcium-Bicarbonate	150	< .05	< .003	< .025			< .002	44.6	23.9	< .005	
215	7/17/2010	Outside Spigot	Valley				< .1	< .003	0.115	< .1				5.96		225
216	10/28/2010	Basement Pressure Tank	Valley		Calcium-Sodium-Bicarbonate	135	< .05	0.004	0.153			< .002	33	4.52	< .005	258
217	10/28/2010	Pressure Tank	Upland		Calcium-Bicarbonate	130	< .05	< .003	0.096			< .002	47	3.88	< .005	300
218	10/28/2010	Bar Sink	Upland		Calcium-Bicarbonate	35	< .05	< .003	0.073			< .002	21.9	6.54	< .005	
219	10/29/2010	Outside Spigot	Valley	Yes	Sodium-Bicarbonate	120	< .05	0.009	0.153			< .002	24.4	9.17	< .005	289
220	10/29/2010		Valley		Calcium-Bicarbonate	115	< .05	< .003	0.16			< .002	37.4	6.56	< .005	
221	10/29/2010	Outside Sink	Valley		Calcium-Sodium-Chloride	25	< .05	< .003	0.085			< .002	27.8	51	< .005	346
222	10/29/2010	Kitchen Faucet	Upland		Calcium-Bicarbonate	125	< .05	< .003	0.091			< .002	41.6	5.16	< .005	323
223	11/10/2010	Kitchen Sink	Upland		Calcium-Bicarbonate	90	< .01	< .001	0.19			< .0001	36	4.96	< .002	223

Table S.8. Groundwater Quality Data for 1701 "Pre-Drill" Water Well Samples from Susquehanna County, Pennsylvania

Water Well ID	Sample Date	Sampling Port	Topo-graphic Location	Gas Extraction Area (Within 1 km of Active Gas Well)	Groundwater Type (If Determined)	Alkalinity (mg/L)	Aluminum (mg/L)	Arsenic (mg/L)	Barium (mg/L)	Boron (mg/L)	Bromide (mg/L)	Cadmium (mg/L)	Calcium (mg/L)	Chloride (mg/L)	Chromium (mg/L)	Conductivity - Field (µs/cm)
224	11/10/2010	Kitchen Sink	Valley		Calcium-Bicarbonate	130	< .01	< .001	0.093			< .0001	24	3.31	< .002	162
225	9/28/2010		Upland		Calcium-Bicarbonate-Chloride	150	< .05	< .002	0.102			< .002	43.2	112	< .005	
226	11/11/2010	Outside Spigot	Valley	Yes	Calcium-Bicarbonate	185	< .01	< .001	0.14			< .0001	49	3.52	< .002	367
227	11/11/2010	Kitchen Faucet	Valley	Yes	Calcium-Bicarbonate	90	< .01	< .001	0.23			< .0001	29	23.9	< .002	280
228	9/28/2010	Kitchen Sink	Valley		Calcium-Bicarbonate	65	< .05	< .002	< .025			< .002	29.1	7.55	< .005	139
229	11/17/2010	Kitchen Sink	Valley	Yes	Calcium-Bicarbonate	155	0.012	0.0013	0.095			< .0001	40	14	< .002	259
230	11/17/2010	Kitchen Sink	Upland		Calcium-Bicarbonate	135	0.18	0.0051	0.3			0.00018	56	15.7	0.02	286
231	11/17/2010	Kitchen Sink	Upland		Calcium-Bicarbonate	25	< .01	< .001	0.043			< .0001	8.5	4.47	< .002	62
232	12/4/2010	Kitchen Sink	Valley	Yes	Calcium-Bicarbonate	160	< .05	< .003	0.13			< .002	47.2	25.1	< .005	323
233	12/4/2010	Kitchen Sink	Valley	Yes	Calcium-Bicarbonate-Chloride	20	0.088	< .003	0.061			< .002	9.6	2.98	< .005	60
234	12/4/2010	Basement At Pressure Tank	Valley	Yes	Calcium-Bicarbonate	115	< .05	< .003	< .025			< .002	42.4	21.8	< .005	223
235	12/4/2010	Kitchen Sink	Valley	Yes	Calcium-Bicarbonate	140	< .05	< .003	0.102			< .002	43.8	4.23	< .005	245
236	12/5/2010	Kitchen Sink	Upland	Yes	Calcium-Bicarbonate-Chloride	20	< .05	< .003	0.109			< .002	12.8	5.1	< .005	110
237	12/5/2010	Kitchen Faucet	Valley	Yes	Calcium-Bicarbonate	155	< .05	< .003	0.131			< .002	46.8	3.87	< .005	336
238	12/5/2010	Barn At Ceiling Spigot	Valley	Yes	Calcium-Sodium-Bicarbonate	100	< .05	0.004	< .025			< .002	29.8	15	< .005	280
239	2/17/2009	Kitchen Sink	Valley	Yes			< .05		0.151					13.4		
240	12/8/2010	Kitchen Sink	Valley	Yes	Calcium-Bicarbonate	160	< .01	0.0011	0.099			< .0001	44	6.75	< .002	267
241	12/8/2010	Kitchen Sink	Upland		Calcium-Sodium-Bicarbonate	35	0.018	< .001	0.02			< .0001	12	9.07	< .002	109
242	12/8/2010	Kitchen Sink	Valley	Yes	Calcium-Bicarbonate	20	0.023	< .001	0.048			< .0001	6.6	3.27	< .002	44
243	12/8/2010	Hand Dug Well	Upland		Calcium-Sodium-Bicarbonate-Chloride	70	0.025	0.0026	0.14			0.00021	31	28	< .002	201
244	2/17/2009	Kitchen Sink	Valley	Yes			< .05		0.093					12.7		
245	4/4/2009	Well Head	Valley	Yes			< .05		0.148					13.5		

Table S.8. Groundwater Quality Data for 1701 "Pre-Drill" Water Well Samples from Susquehanna County, Pennsylvania

Water Well ID	Sample Date	Sampling Port	Topo-graphic Location	Gas Extraction Area (Within 1 km of Active Gas Well)	Groundwater Type (If Determined)	Alkalinity (mg/L)	Aluminum (mg/L)	Arsenic (mg/L)	Barium (mg/L)	Boron (mg/L)	Bromide (mg/L)	Cadmium (mg/L)	Calcium (mg/L)	Chloride (mg/L)	Chromium (mg/L)	Conductivity - Field (µs/cm)
246	12/11/2010	Kitchen Sink	Valley		Calcium-Sodium-Bicarbonate	70	< .01	< .001	0.36			< .0001	17	< 2	< .002	127
247	12/11/2010		Upland		Calcium-Sodium-Bicarbonate-Chloride	35	0.011	< .001	0.096			< .0001	15	15.9	< .002	
248	12/11/2010	Kitchen Sink	Valley		Calcium-Bicarbonate	95	0.018	0.0014	0.22			< .0001	29	< 2	< .002	152
249	3/16/2009	Basement At Pressure Tank	Valley	Yes			< .05		0.169					10.5		
250	12/17/2010	Pressure Tank	Upland		Calcium-Bicarbonate	40	0.017	0.00083	0.076			7.60E-05	16	5.06	0.002	129
251	12/17/2010	Pressure Tank	Upland		Calcium-Bicarbonate	40	0.052	0.0058	0.13			< .0001	17	8.49	< .002	139
252	12/18/2010	Basement Pressure Tank	Valley	Yes	Calcium-Bicarbonate	175	< .01	< .001	0.065			< .0001	44	5.86	< .002	253
253	6/24/2009	Kitchen Sink	Upland	Yes			< .1		0.266					170		
254	12/18/2010	Kitchen Sink	Valley	Yes	Calcium-Sodium-Bicarbonate	120	< .01	0.0087	1.2			< .0001	26	13.7	< .002	193
255	3/19/2009	Kitchen Sink	Valley	Yes			< .05		0.091					58.6		
256	3/19/2009	Kitchen Sink	Upland	Yes			< .05		< .025					5.13		
257	12/14/2010	Kitchen Sink	Valley	Yes	Calcium-Bicarbonate	120	< .01	< .001	0.076			< .0001	39	4.57	< .002	179
258	3/4/2009	Kitchen Sink	Upland				< .05		0.092					6.92		
259	12/14/2010	Hydrant	Valley	Yes	Calcium-Bicarbonate	105	1.2	0.038	0.25			0.0017	38	< 2	0.0031	181
260	12/14/2010	Kitchen Sink	Valley	Yes	Calcium-Bicarbonate	130	< .01	0.0012	0.2			< .0001	34	8.35	< .002	200
261	12/14/2010	Bathtub Faucet	Valley	Yes	Calcium-Bicarbonate-Chloride	105	< .01	< .001	0.059			< .0001	47	36.1	< .002	286
262	2/23/2011		Valley	Yes	Calcium-Bicarbonate	110	0.11	< .001	0.046			< .0001	32	6.72	< .002	
263	12/19/2010		Valley	Yes	Calcium-Bicarbonate	110	< .01	< .001	0.025			< .0001	45	15.7	< .002	
264	12/19/2010	Kitchen Sink	Valley		Calcium-Sodium-Bicarbonate	115	< .01	< .001	1.8			< .0001	16	8.42	< .002	265
265	12/19/2010	Kitchen Faucet	Valley		Calcium-Bicarbonate	40	0.011	< .001	0.048			< .0001	15	11.5	< .002	133

Table S.8. Groundwater Quality Data for 1701 "Pre-Drill" Water Well Samples from Susquehanna County, Pennsylvania

Water Well ID	Sample Date	Sampling Port	Topo-graphic Location	Gas Extraction Area (Within 1 km of Active Gas Well)	Groundwater Type (If Determined)	Alkalinity (mg/L)	Aluminum (mg/L)	Arsenic (mg/L)	Barium (mg/L)	Boron (mg/L)	Bromide (mg/L)	Cadmium (mg/L)	Calcium (mg/L)	Chloride (mg/L)	Chromium (mg/L)	Conductivity - Field (µs/cm)
266	12/19/2010	School Art Studio Bathroom Sink	Valley	Yes	Calcium-Bicarbonate	85	< .01	< .001	0.07			< .0001	42	18.2	< .002	312
267	2/26/2009		Upland				<0.05		0.072					12.4		
268	1/27/2011	Downstairs Kitchen Sink	Valley		Sodium-Bicarbonate	140	< .01	0.0033	0.071			< .0001	4.8	10.5	< .002	256
269	1/27/2011	Kitchen Sink	Valley		Calcium-Bicarbonate	75	< .01	0.00041	0.056			< .0001	30	5.18	< .002	155
270	1/27/2011	Kitchen Sink	Valley		Calcium-Bicarbonate	60	< .01	< .001	0.049			< .0001	27	5.26	< .002	163
271	3/4/2009	Kitchen Sink	Upland				< .05		0.102					2.75		
272	2/11/2011	Kitchen Sink	Upland	Yes	Calcium-Bicarbonate	170	< .01	< .001	0.11			< .0001	54	5.86	< .002	270
273	2/11/2011	Kitchen Sink	Upland		Calcium-Bicarbonate	160	0.065	< .001	0.22			< .0001	76	52.2	< .002	358
274	2/11/2011	Kitchen Sink	Upland		Sodium-Bicarbonate	165	< .01	< .001	0.54			< .0001	11	5.3	< .002	213
275	2/11/2011	Kitchen Sink	Upland	Yes	Calcium-Bicarbonate	195	< .01	< .001	0.2			< .0001	71	48.1	< .002	441
276	2/11/2011	In Milkhouse, At Bottom Of Pressure Tank	Upland		Calcium-Bicarbonate	115	< .01	0.0013	0.14			< .0001	49	11.4	< .002	234
277	2/4/2011	Pressure Tank	Valley	Yes	Calcium-Bicarbonate	160	< .01	0.0015	0.11			< .0001	42	5.21	< .002	335
278	2/8/2011	Bathtub	Valley		Calcium-Bicarbonate	110	< .01	< .001	0.083			< .0001	47	11.1	< .002	
279	3/4/2009	Kitchen Sink	Upland				< .05		0.106					50.3		
280	2/8/2011	Kitchen Sink	Upland		Calcium-Bicarbonate-Chloride	55	< .01	< .001	0.17			< .0001	37	16.3	< .002	196
281	10/19/2010	Kitchen Sink	Valley	Yes	Calcium-Bicarbonate	115	0.05	< .003	0.062			< .002	38.2	6.99	< .005	269
282	2/8/2011	Kitchen Sink, Small Spigot On Right Hand Side Of S	Valley		Calcium-Bicarbonate	110	< .01	< .001	0.065			< .0001	37	7.41	< .002	169
283	2/11/2011	Kitchen Sink	Upland	Yes	Sodium-Bicarbonate	195	< .01	< .001	0.01			< .0001	7.1	26	< .002	
284	2/11/2011	Kitchen Sink	Upland		Calcium-Bicarbonate	110	< .01	< .001	0.19			< .0001	35	5.49	< .002	161

Table S.8. Groundwater Quality Data for 1701 "Pre-Drill" Water Well Samples from Susquehanna County, Pennsylvania

Water Well ID	Sample Date	Sampling Port	Topo-graphic Location	Gas Extraction Area (Within 1 km of Active Gas Well)	Groundwater Type (If Determined)	Alkalinity (mg/L)	Aluminum (mg/L)	Arsenic (mg/L)	Barium (mg/L)	Boron (mg/L)	Bromide (mg/L)	Cadmium (mg/L)	Calcium (mg/L)	Chloride (mg/L)	Chromium (mg/L)	Conductivity - Field (µs/cm)
285	2/8/2011	Rental (Adam Vibbard) Kitchen Sink	Upland		Calcium-Bicarbonate	130	< .01	< .001	0.26			< .0001	45	18.8	< .002	233
286	2/8/2011	Kitchen Sink	Upland		Calcium-Bicarbonate	70	< .01	< .001	0.097			< .0001	25	19.7	< .002	151
287	1/13/2011	Kitchen Sink	Valley	Yes			< .01		0.26					4.05		188
288	2/15/2011	Pressure Tank	Upland		Calcium-Bicarbonate	125	< .01	< .001	0.21			< .0001	32	4.12	< .002	258
289	2/15/2011	Laundry Room Faucet	Upland		Calcium-Sodium-Bicarbonate	140	< .01	0.0028	0.48			< .0001	21	5.36	< .002	278
290	3/4/2009	Kitchen Sink	Valley				< .05		< .025					< 2		
291	2/14/2011	Kitchen Sink	Upland		Calcium-Bicarbonate	160	< .01	< .001	0.27			< .0001	42	4.36	< .002	224
292	2/15/2011	Kitchen Faucet	Valley		Calcium-Sodium-Bicarbonate-Chloride	130	< .01	< .001	0.11			< .0001	50	174	< .002	821
293	2/14/2011	Barn Milkhouse Sink	Valley		Calcium-Bicarbonate	125	< .01	< .001	0.037			< .0001	34	2.6	< .002	183
294	2/15/2011	Milkhouse Faucet	Upland	Yes	Calcium-Sodium-Bicarbonate	110	< .01	0.0049	0.18			< .0001	29	12.5	< .002	283
295	2/15/2011	1St Floor Kitchen Faucet	Upland	Yes	Calcium-Bicarbonate	135	< .01	< .001	0.12			< .0001	41	9.91	< .002	288
296	2/17/2011	Pressure Tank In Basement	Upland	Yes	Calcium-Bicarbonate	215	< .01	< .001	0.049			< .0001	60	7.24	< .002	478
297	3/4/2009	1St Floor Mens Room Sink	Valley				< .05		0.09					5.02		
298	3/21/2011		Valley		Sodium-Chloride	85.8	< .03	0.0024	5.9			< .001	40	688	< .002	
299	3/21/2011		Upland	Yes	Calcium-Bicarbonate-Chloride	76.7	< .03	< .001	0.15			< .001	37.2	60.3	< .002	
300	3/22/2011		Valley	Yes	Calcium-Bicarbonate	119	< .03	0.0072	0.33			< .001	28	6.2	< .002	
301	3/4/2009	Kitchen Sink	Valley				< .05		0.045					2.5		
302	12/10/2010	Garage Faucet	Upland	Yes	Calcium-Bicarbonate	170	< .01	0.001	0.072			< .0001	43	6.34	< .002	373
303	12/10/2010	Kitchen Faucet	Upland	Yes	Calcium-Bicarbonate	120	< .01	< .001	0.11			< .0001	38	2.6	< .002	250

Table S.8. Groundwater Quality Data for 1701 "Pre-Drill" Water Well Samples from Susquehanna County, Pennsylvania

Water Well ID	Sample Date	Sampling Port	Topo-graphic Location	Gas Extraction Area (Within 1 km of Active Gas Well)	Groundwater Type (If Determined)	Alkalinity (mg/L)	Aluminum (mg/L)	Arsenic (mg/L)	Barium (mg/L)	Boron (mg/L)	Bromide (mg/L)	Cadmium (mg/L)	Calcium (mg/L)	Chloride (mg/L)	Chromium (mg/L)	Conductivity - Field (µs/cm)
304	12/10/2010	Kitchen Sink	Valley	Yes	Calcium-Bicarbonate	95	< .01	< .001	0.06			< .0001	39	17.2	< .002	291
305	12/10/2010		Upland	Yes	Calcium-Bicarbonate	175	< .01	0.0012	0.051			< .0001	38	< 2	< .002	326
306	12/10/2010	Pressure Tank	Upland		Calcium-Sodium-Bicarbonate-Chloride	75	0.013	< .001	0.017			< .0001	39	41.5	< .002	364
307	12/10/2010	Kitchen Faucet	Valley		Calcium-Bicarbonate	30	0.018	< .001	0.017			< .0001	13	2.64	< .002	96
308	12/14/2010	Kitchen Sink	Valley	Yes	Calcium-Bicarbonate	110	< .01	0.0011	0.029			< .0001	41	7.26	< .002	195
309	3/12/2009	Kitchen Sink Faucet	Upland				< .05		0.039					< 2		
310	12/20/2010	Kitchen Sink	Upland		Calcium-Bicarbonate	160	0.05	< .001	0.17			< .0001	46	4.77	< .002	243
311	12/20/2010	Pressure Tank	Valley		Calcium-Sodium-Bicarbonate	130	< .01	0.0039	0.2			< .0001	30	2.92	< .002	207
312	12/20/2010	Basement At Pressure Tank	Valley		Calcium-Bicarbonate	155	< .01	< .001	0.15			< .0001	45	3.73	< .002	241
313	12/20/2010	Kitchen Sink	Upland		Calcium-Bicarbonate	90	< .01	< .001	0.24			< .001	28	4.92	< .002	142
314	12/20/2010	Kitchen Sink	Upland		Calcium-Bicarbonate	80	0.011	< .001	0.18			< .0001	27	3.79	< .002	135
315	3/4/2009	Pressure Tank In Basement	Upland				< .05		0.047					2.2		
316	12/20/2010	Bathtub Faucet	Upland		Calcium-Bicarbonate	80	0.011	< .001	0.23			< .0001	26	3.35	< .002	137
317	4/15/2009	Horse Barn Faucet	Valley	Yes			< .1		0.075					4.84		
318	1/6/2011	Kitchen Sink	Upland	Yes	Calcium-Sodium-Bicarbonate	120	0.011	< .001	0.46			< .0001	16	12.9	< .002	210
319	4/19/2009	Kitchen Sink Faucet	Valley	Yes			< .05		0.07					44.3		252
320	4/8/2009	Barn After Filter	Valley				< .05		0.067					63.9		
321	4/12/2011	Cwt Of Kitchen Sink	Valley		Calcium-Bicarbonate	114	< .03	< .001	0.086			< .001	36.5	10.2	< .002	247
322	4/13/2011	Well	Upland	Yes	Calcium-Sodium-Bicarbonate	114	< .03	0.0053	0.35			< .001	27.6	15.2	< .002	
323	4/13/2011	Kitchen Sink	Valley	Yes	Calcium-Bicarbonate	71.3	< .03	< .001	0.021			< .001	26.8	2	< .002	128
324	4/13/2011	Kitchen Sink Cwt	Upland		Calcium-Bicarbonate	134	0.046	< .001	0.095			< .001	39.3	2.9	< .002	225

Table S.8. Groundwater Quality Data for 1701 "Pre-Drill" Water Well Samples from Susquehanna County, Pennsylvania

Water Well ID	Sample Date	Sampling Port	Topo-graphic Location	Gas Extraction Area (Within 1 km of Active Gas Well)	Groundwater Type (If Determined)	Alkalinity (mg/L)	Aluminum (mg/L)	Arsenic (mg/L)	Barium (mg/L)	Boron (mg/L)	Bromide (mg/L)	Cadmium (mg/L)	Calcium (mg/L)	Chloride (mg/L)	Chromium (mg/L)	Conductivity - Field (µs/cm)
325	4/13/2011	Kitchen Sink Cwt	Upland		Calcium-Sodium-Bicarbonate	121	< .03	0.0064	0.076			< .001	28	15.9	< .002	188
326	3/12/2009	Basement Before Filter	Upland	Yes			< .05		0.078					6.1		
327	4/14/2011	Farmhouse Kitchen Sink Cwt	Valley		Calcium-Bicarbonate	117	< .03	< .001	0.11			< .001	45.1	15.1	< .002	273
328	4/15/2011	Kitchen Sink Cwt	Upland		Calcium-Bicarbonate	120	< .03	0.001	0.12			< .001	43.3	10.3	< .002	233
329	4/18/2011	Pressure Tank In Basement	Valley		Calcium-Sodium-Chloride	32.1	< .03	< .001	0.016			< .001	24.1	103	< .002	373
330	4/18/2011	Kitchen Sink	Upland		Calcium-Bicarbonate	170	< .03	< .001	0.042			< .001	63.8	10.3	< .002	
331	4/19/2011	Kitchen Faucet	Upland			163	< .03	< .001	0.071		< .02	< .001	42		< .002	155
332	4/22/2011	Mary Steele Kitchen Sink	Valley	Yes	Calcium-Bicarbonate	63	< .03	< .001	0.06			< .001	27.6	18	< .002	
333	4/26/2011	Kicthen Sink	Valley	Yes	Calcium-Bicarbonate	117	< .03	< .001	0.055			< .001	35.3	12.4	< .002	
334	4/27/2011	Well 2	Upland	Yes	Calcium-Bicarbonate	151	< .03	< .001	0.065		< .2	< .001	40.7	3.6	< .002	
335	4/28/2011	Basement Pressure Tank	Valley	Yes	Calcium-Bicarbonate	133	0.13	< .001	0.028		< .2	< .001	33.9	9.5	< .002	
336	4/28/2011	Pressure Tank In Basement On E Side Of House	Valley	Yes	Calcium-Bicarbonate	118	< .03	< .001	0.031		< .2	< .001	40.7	16.3	< .002	
337	5/2/2011		Upland	Yes		104	<0.03	0.002	0.1		<0.2	<0.001	38.2		<0.002	
338	5/2/2011		Upland	Yes	Calcium-Sodium-Chloride	28.3	0.068	<0.001	0.095		<0.2	<0.001	15	39.9	0.003	
339	5/3/2011	Kitchen Sink	Valley	Yes	Calcium-Bicarbonate	107	< .03	< .001	0.029		< .2	< .001	41.1	22.7	< .002	
340	5/4/2011	Kitchen Cwt	Valley	Yes	Calcium-Bicarbonate	94.9	< .03	< .001	0.048		< .2	< .001	31	3.8	< .002	
341	5/4/2011	Kitchen Sink	Valley	Yes	Calcium-Bicarbonate	93.6	< .03	< .001	0.12		< .2	< .001	32	3.8	< .002	
342	5/4/2011	Outside Hose Bib On The East Side Of Home	Valley	Yes	Calcium-Bicarbonate	155	< .03	< .001	0.074		< .2	< .001	45.1	23.1	< .002	0.347
343	5/4/2011	Outdoor Spigot	Valley	Yes	Calcium-Bicarbonate	112	< .03	< .001	0.04		< .2	< .001	35.8	14.5	< .002	249
344	2/26/2009	Kitchen Sink	Upland				< .05		0.052					9.07		

Table S.8. Groundwater Quality Data for 1701 "Pre-Drill" Water Well Samples from Susquehanna County, Pennsylvania

Water Well ID	Sample Date	Sampling Port	Topo-graphic Location	Gas Extraction Area (Within 1 km of Active Gas Well)	Groundwater Type (If Determined)	Alkalinity (mg/L)	Aluminum (mg/L)	Arsenic (mg/L)	Barium (mg/L)	Boron (mg/L)	Bromide (mg/L)	Cadmium (mg/L)	Calcium (mg/L)	Chloride (mg/L)	Chromium (mg/L)	Conductivity - Field (µs/cm)
345	5/4/2011	School Art Studio Bathroom Sink	Valley	Yes	Calcium-Bicarbonate	102	0.11	< .001	0.071		< .2	< .001	39	28.1	< .002	
346	5/4/2011	Pressure Tank	Valley	Yes	Calcium-Bicarbonate	101	< .03	< .001	0.045		< .2	< .001	37.1	25.6	< .002	
347	5/5/2011	Kitchen Sink Cwt	Upland	Yes	Calcium-Sodium-Bicarbonate	219	< .03	< .001	0.015		< .2	< .001	39.8	< 1	< .002	0.365
348	5/5/2011	Kitchen Sink	Valley	Yes	Calcium-Sodium-Bicarbonate	200	< .03	< .001	0.01		< .2	< .001	40	15.8	< .002	
349	5/5/2011	Pressure Tank	Valley	Yes	Calcium-Bicarbonate-Chloride	109	0.04	< .001	0.023		< .2	< .001	38.5	60.4	< .002	
350	5/5/2011	Kitchen Sink Cwt	Valley	Yes	Calcium-Bicarbonate	123	< .03	< .001	0.019		< .2	< .001	34.4	21.9	< .002	277
351	5/5/2011	Pressure Tank	Valley	Yes	Calcium-Bicarbonate	176	< .03	0.0025	0.12		< .2	< .001	43.5	2.6	< .002	0.314
352	5/6/2011	Pressure Tank	Valley	Yes	Calcium-Bicarbonate	173	< .03	< .001	0.043		0.14	< .001	49	18.5	< .002	364
353	5/6/2011	Kitchen Sink	Valley	Yes	Calcium-Bicarbonate	115	< .03	< .001	0.066		< .2	< .001	35.6	6.3	< .002	
354	5/7/2011	Pressure Tank In Basement	Valley	Yes	Calcium-Bicarbonate	166	< .03	0.0013	0.11		< .2	< .001	34.8	3.4	< .002	271
355	5/7/2011	Bathroom Sink Ctw	Valley	Yes	Calcium-Bicarbonate	178	< .03	< .001	0.1		< .2	< .001	36.7	2.8	< .002	0.298
356	5/7/2011	Backroom Sink	Valley	Yes	Calcium-Bicarbonate	160	< .03	< .001	0.095		< .2	< .001	34.6	2.5	< .002	
357	5/7/2011	Pressure Tank In Basement	Upland	Yes	Calcium-Bicarbonate	181	< .03	< .001	0.077		< .2	< .001	36.5	2.2	< .002	287
358	5/7/2011	Kitchen Sink	Valley	Yes	Calcium-Bicarbonate	118	0.037	< .001	0.067		< .2	< .001	48.4	25.6	< .002	260
359	5/7/2011	Pressure Tank	Valley	Yes	Calcium-Bicarbonate	125	< .03	< .001	0.058		< .2	< .001	42.5	18.6	< .002	
360	5/9/2011	Pressure Tank	Valley	Yes	Calcium-Bicarbonate-Chloride	86.7	< .03	0.0012	0.026		< .2	< .001	34.6	34.4	< .002	0.277
361	5/9/2011	Basement Pressure Tank	Valley	Yes	Calcium-Bicarbonate	123	< .03	0.0012	0.023		< .2	< .001	41.4	21.3	< .002	
362	5/9/2011	Well	Valley	Yes	Calcium-Sodium-Bicarbonate-Chloride	55.3	< .03	< .001	0.04		< .2	< .001	18.6	30.5	< .002	
363	5/9/2011	Kitchen Sink	Valley	Yes	Calcium-Bicarbonate-Chloride	122	< .03	< .001	0.047		< .2	< .001	39.9	43.7	< .002	

Table S.8. Groundwater Quality Data for 1701 "Pre-Drill" Water Well Samples from Susquehanna County, Pennsylvania

Water Well ID	Sample Date	Sampling Port	Topo-graphic Location	Gas Extraction Area (Within 1 km of Active Gas Well)	Groundwater Type (If Determined)	Alkalinity (mg/L)	Aluminum (mg/L)	Arsenic (mg/L)	Barium (mg/L)	Boron (mg/L)	Bromide (mg/L)	Cadmium (mg/L)	Calcium (mg/L)	Chloride (mg/L)	Chromium (mg/L)	Conductivity - Field (µs/cm)
364	5/9/2011	Outdoor Hose Bib On South Side Of Gas Station	Valley	Yes	Calcium-Bicarbonate	180	< .03	< .001	0.061		< .2	< .001	35.6	3.9	< .002	
365	5/9/2011	Outside Spigot	Valley	Yes	Calcium-Bicarbonate	170	< .03	0.0013	0.13		< .2	< .001	40.4	2.4	< .002	285
366	5/9/2011	Pressure Tank	Upland	Yes	Calcium-Bicarbonate-Chloride	68.7	0.073	0.0011	0.09		< .2	< .001	27.4	37.2	0.0049	
367	5/9/2011	Pressure Tank	Valley	Yes	Calcium-Sodium-Bicarbonate-Chloride	62.9	< .03	< .001	0.061		< .2	< .001	28.7	49.5	< .002	
368	5/9/2011	Outside Faucet	Valley	Yes	Calcium-Bicarbonate	179	< .03	< .001	0.06		< .2	< .001	34.7	3.7	< .002	
369	5/10/2011	Kitchen Sink	Upland	Yes	Calcium-Sodium-Chloride	15.8	0.087	< .001	0.055		< .2	< .001	9.1	22.4	< .002	140
370	5/10/2011	Inside Faucet	Valley	Yes	Calcium-Sodium-Bicarbonate-Chloride	62.1	< .03	< .001	0.044		< .2	< .001	26.7	43.6	< .002	
371	5/10/2011	Inside Faucet	Valley	Yes	Calcium-Bicarbonate	159	< .03	< .001	< .01		< .2	< .001	36	10	< .002	339
372	5/11/2011		Valley	Yes	Calcium-Sodium-Bicarbonate-Chloride	32.4	0.1	< .001	0.024		< .2	< .001	11.8	18.8	< .002	
373	5/11/2011	Pressure Tank In Basement	Valley	Yes	Calcium-Bicarbonate	135	< .03	< .001	0.12		< .2	< .001	43.5	27.3	< .002	0.435
374	5/11/2011		Valley	Yes	Calcium-Sodium-Bicarbonate-Chloride	50.9	< .03	< .001	0.022		< .2	< .001	25.4	47.5	< .002	
375	5/11/2011	Kitchen Sink	Valley	Yes	Calcium-Sodium-Bicarbonate-Chloride	35	< .03	< .001	0.092		< .2	< .001	16.4	32.5	< .002	
376	5/11/2011	Outside Bib	Valley	Yes	Calcium-Bicarbonate	133	< .03	< .001	0.1		< .2	< .001	38.2	36.7	< .002	443
377	5/11/2011	Kitchen Sink	Valley	Yes	Calcium-Bicarbonate-Chloride	76.2	< .03	< .001	0.079		< .2	< .001	34	46.4	< .002	276
378	5/11/2011	Pressure Tank In Basement	Valley	Yes	Calcium-Sodium-Bicarbonate-Chloride	74.7	< .03	< .001	0.015		< .2	< .001	32.3	61.3	< .002	
379	5/12/2011		Valley	Yes	Calcium-Bicarbonate	130	< .03	< .001	0.045		< .2	< .001	34.6	19.8	< .002	

Table S.8. Groundwater Quality Data for 1701 "Pre-Drill" Water Well Samples from Susquehanna County, Pennsylvania

Water Well ID	Sample Date	Sampling Port	Topo-graphic Location	Gas Extraction Area (Within 1 km of Active Gas Well)	Groundwater Type (If Determined)	Alkalinity (mg/L)	Aluminum (mg/L)	Arsenic (mg/L)	Barium (mg/L)	Boron (mg/L)	Bromide (mg/L)	Cadmium (mg/L)	Calcium (mg/L)	Chloride (mg/L)	Chromium (mg/L)	Conductivity - Field (µs/cm)
380	5/12/2011	Outside Faucet	Valley	Yes	Calcium-Sodium-Bicarbonate-Chloride	38.9	< .03	< .001	0.11		< .2	< .001	20.1	48.9	< .002	0.299
381	5/12/2011	Basement At Pressure Tank	Valley	Yes	Calcium-Bicarbonate	121	< .03	< .001	0.019		< .2	< .001	32.9	17	< .002	260
382	5/12/2011	Pressure Tank	Valley	Yes	Sodium-Chloride	78.6	< .03	< .001	0.14		0.17	< .001	65	1310	< .002	
383	5/12/2011	Kitchen Sink	Valley	Yes	Calcium-Bicarbonate-Chloride	85.6	< .03	< .001	0.03		< .2	< .001	34.4	62.8	< .002	
384	5/13/2011	Pressure Tank Rental Property	Valley	Yes	Calcium-Sodium-Bicarbonate-Chloride	113	< .03	< .001	0.018		< .2	< .001	37.2	49.3	< .002	334
385	5/13/2011	Pressure Tank	Valley	Yes	Calcium-Bicarbonate	164	< .03	< .001	0.092		< .2	< .001	38.8	1.9	< .002	263
386	5/13/2011	Kitchen Sink	Valley	Yes	Calcium-Sodium-Bicarbonate-Chloride	39.2	< .03	< .001	0.11		< .2	< .001	23.4	41.3	< .002	0.23
387	5/13/2011	Outside Faucet	Valley	Yes	Calcium-Bicarbonate	201	< .03	0.0012	0.24		< .2	< .001	45.2	11.5	< .002	363
388	1/31/2011	Kitchen Sink	Valley		Calcium-Bicarbonate	125	< .01	< .001	0.12			< .0001	39	4.85	< .002	187
389	1/8/2011	Kitchen Sink	Upland	Yes	Calcium-Bicarbonate	110	< .01	0.0011	0.11			< .0001	39	18.5	< .002	196
390	1/8/2011	Kitchen Sink	Valley		Calcium-Bicarbonate-Chloride	15	0.05	< .001	0.1			< .0001	14	6.8	< .002	78
391	1/4/2011	Kitchen Sink	Valley	Yes	Calcium-Bicarbonate-Chloride	45	0.026	< .001	0.09			< .0001	15	16.6	< .002	
392	2/17/2011	Outside Spigot	Upland		Sodium-Bicarbonate	145	< .01	0.013	0.074			< .0001	2.1	8.16	< .002	342
393	2/22/2011	Kitchen Sink	Upland	Yes	Calcium-Bicarbonate	145	< .01	0.0011	0.17			< .0001	46	42.6	< .002	254
394	2/22/2011	Pressure Tank In Barn, Old Milkhouse	Upland		Calcium-Bicarbonate	160	< .01	< .001	0.094			< .0001	50	22	< .002	300
395	2/22/2011	Rental Kitchen Sink	Upland		Calcium-Bicarbonate	165	< .01	< .001	0.094			< .0001	58	34.8	< .002	371
396	1/8/2011	Basement At Pressure Tank	Valley	Yes	Calcium-Bicarbonate	75	0.13	< .001	0.15			0.00094	23	5	< .002	132
397	1/31/2011	Kitchen Sink	Valley		Calcium-Bicarbonate	30	0.079	< .001	0.064			< .0001	10	2.65	< .002	58

Table S.8. Groundwater Quality Data for 1701 "Pre-Drill" Water Well Samples from Susquehanna County, Pennsylvania

Water Well ID	Sample Date	Sampling Port	Topo-graphic Location	Gas Extraction Area (Within 1 km of Active Gas Well)	Groundwater Type (If Determined)	Alkalinity (mg/L)	Aluminum (mg/L)	Arsenic (mg/L)	Barium (mg/L)	Boron (mg/L)	Bromide (mg/L)	Cadmium (mg/L)	Calcium (mg/L)	Chloride (mg/L)	Chromium (mg/L)	Conductivity - Field (µs/cm)
398	1/9/2011	Hand Dug Well West Of House	Upland	Yes	Calcium-Bicarbonate-Chloride	20	0.062	< .001	0.04			< .0001	9	4.04	< .002	76
399	1/4/2011	Pressure Tank	Upland		Calcium-Bicarbonate	125	< .01	< .001	0.042	< .0001		< .002	38	4.5	< .002	190
400	3/12/2009	Kitchen Sink	Valley				< .05		0.31					< 2		
401	1/31/2011	Kitchen Sink	Upland	Yes	Calcium-Bicarbonate	155	0.015	0.024	0.11			< .0001	50	6.91	< .002	223
402	2/22/2011	Upstairs Kitchen Sink	Upland		Calcium-Bicarbonate	85	< .01	< .001	0.12			< .0001	36	< 2	< .002	200
403	1/31/2011	Kitchen Sink	Upland	Yes	Calcium-Bicarbonate	80	< .01	< .001	0.1			< .0001	27	5.68	< .002	150
404	1/31/2011	Kitchen Sink	Upland		Sodium-Bicarbonate	155	< .01	0.0026	0.27			< .0001	9.4	12.7	< .002	228
405	1/4/2011	Hand Dug Well	Upland		Calcium-Bicarbonate-Chloride	15	0.016	< .001	0.01			< .0001	6.8	2.38	< 2	35
406	2/22/2011	Basement Of Rental At Pressure Tank	Upland		Calcium-Bicarbonate	225	< .01	< .001	0.16			< .0001	52	10.4	< .002	340
407	1/8/2011	Basement At Pressure Tank	Upland		Calcium-Bicarbonate	85	< .01	< .001	0.045			< .0001	25	6.17	< .002	115
408	1/8/2011	Kithcen Sink	Upland		Calcium-Bicarbonate	70	< .01	< .001	0.015			< .0001	27	4.23	< .002	125
409	1/14/2011	Laundry Room Sink	Upland		Calcium-Bicarbonate	110	< .01	< .001	0.073			< .0001	37	5.82	< .002	176
410	3/12/2009	Kitchen Sink	Valley				< .05		0.093					< 2		
411	1/31/2011	Kitchen Sink	Valley		Calcium-Sodium-Bicarbonate	115	0.023	< .001	0.18			< .0001	19	7.24	< .002	160
412	2/22/2011	Breakroom Sink- Lower Level	Upland		Calcium-Bicarbonate	185	< .01	0.001	0.2			< .0001	51	32.5	< .002	325
413	3/16/2009	Kitchen Sink	Valley				< .05		0.163					< 2		
414	1/8/2011	Kitchen Sink	Valley	Yes	Calcium-Bicarbonate	155	< .01	< .001	0.017			< .0001	37	3.5	< .002	217
415	1/4/2011	Ktichen Sink	Upland		Calcium-Bicarbonate	45	0.022	< .001	0.12			< .0001	16	3.85	< .002	81
416	1/31/2011	Kitchen Sink	Valley	Yes	Calcium-Bicarbonate	85	< .01	< .001	0.14			< .0001	25	4.16	< .002	135
417	1/10/2011	Hand Dug Well	Upland		Calcium-Bicarbonate	35	0.026	< .001	0.024			0.00011	15	5.4	< .002	70
418	2/22/2011	Kitchen Sink	Valley		Calcium-Bicarbonate	105	0.017	0.0019	0.36			0.00021	27	4.04	< .002	168

Table S.8. Groundwater Quality Data for 1701 "Pre-Drill" Water Well Samples from Susquehanna County, Pennsylvania

Water Well ID	Sample Date	Sampling Port	Topo-graphic Location	Gas Extraction Area (Within 1 km of Active Gas Well)	Groundwater Type (If Determined)	Alkalinity (mg/L)	Aluminum (mg/L)	Arsenic (mg/L)	Barium (mg/L)	Boron (mg/L)	Bromide (mg/L)	Cadmium (mg/L)	Calcium (mg/L)	Chloride (mg/L)	Chromium (mg/L)	Conductivity - Field (µs/cm)
419	1/10/2011	Kitchen Sink	Valley		Calcium-Bicarbonate	225	< .01	< .001	0.16			< .0001	54	3.51	< .002	293
420	1/4/2011		Upland		Calcium-Bicarbonate	60	< .01	< .001	0.11			< .0001	23	7.61	< .002	
421	2/17/2011	Kitchen Faucet On 2Nd Floor	Upland		Calcium-Bicarbonate	80	< .01	< .001	0.11			< .0001	26	6.91	< .002	190
422	1/4/2011		Upland	Yes	Calcium-Bicarbonate	120	< .01	< .001	3.3			< .0001	27	18.3	< .002	
423	1/7/2011	Milkhouse Faucet	Valley	Yes	Calcium-Bicarbonate	115	< .01	< .001	0.19			< .0001	34	23.3	< .002	307
424	1/7/2011	Kitchen Sink Rental	Upland	Yes	Calcium-Bicarbonate	110	0.02	< .001	0.37			< .0001	34	3.46	< .002	227
425	10/8/2009		Upland				1.01		0.696					5.04		
426	3/13/2009	Kitchen Sink	Valley				< .05		0.806					20.4		
427	3/12/2009	Kitchen Sink	Upland				0.051		0.062					2.49		
428	6/24/2009		Upland	Yes			< .1		0.026					2.15		
429	3/4/2009		Upland				<0.05		0.106					50.3		
430	6/13/2011	Barn Spigot	Upland													164
431	6/14/2011	Kitchen Sink Overlooking Road	Upland													180
432	6/14/2011	Pressure Tank	Valley													208
433	6/16/2011	Kitchen Sink On Island In Kitchen	Valley													225
434	6/16/2011	Kitchen Sink	Valley													160
435	6/16/2011	In Barn Milkhouse-At Sink	Valley													292
436	6/16/2011	Outside Spigot- Next To Porch At End Of Driveway	Upland													155
437	6/16/2011	Kitchen Sink On Lake Road Side Of House Under Window	Valley													221
438	6/17/2011	Kitchen Sink	Valley													388

Table S.8. Groundwater Quality Data for 1701 "Pre-Drill" Water Well Samples from Susquehanna County, Pennsylvania

Water Well ID	Sample Date	Sampling Port	Topo-graphic Location	Gas Extraction Area (Within 1 km of Active Gas Well)	Groundwater Type (If Determined)	Alkalinity (mg/L)	Aluminum (mg/L)	Arsenic (mg/L)	Barium (mg/L)	Boron (mg/L)	Bromide (mg/L)	Cadmium (mg/L)	Calcium (mg/L)	Chloride (mg/L)	Chromium (mg/L)	Conductivity - Field (µs/cm)
439	6/23/2011	Kitchen Sink	Valley													
440	6/23/2011	Pressure Tank	Upland													194
441	6/23/2011	Pressure Tank	Valley													194
442	6/24/2011		Valley													
443	6/24/2011	Kitchen Sink	Upland													159
444	6/24/2011	Kitchen Sink	Valley													252
445	6/24/2011	Kitchen Sink	Upland													247
446	6/24/2011	Shed Spigot	Valley													247
447	6/13/2011	Kitchen Faucet	Upland													
448	6/15/2011	Kitchen Sink Near Rear Of House	Upland													125
449	6/15/2011	Kitchen Sink On South Side Of House	Upland													192
450	6/15/2011	Kitchen Sink On Wall Closest To Red Barn Behind House	Upland													149
451	6/15/2011	Kitchen Sink	Valley													159
452	6/15/2011	Outside Spigot- On The North Side Of The Residence	Upland													113
453	6/15/2011		Upland													
454	3/25/2009	Kitchen Sink	Upland				< .05		0.182					3.84		
455	6/27/2011	Greenhouse Sink	Valley													257
456	4/15/2009	Kitchen Sink	Valley				< .1		0.083					4.2		
457	4/19/2009	Basement Pressure Tank	Valley				< .05		0.065					4.87		
458	6/13/2011	Outside Hose Bib	Valley	Yes	Calcium-Bicarbonate	65	< .03	< .001	0.06		< .2	< .001	22	2.9	< .002	0.127

Table S.8. Groundwater Quality Data for 1701 "Pre-Drill" Water Well Samples from Susquehanna County, Pennsylvania

Water Well ID	Sample Date	Sampling Port	Topo-graphic Location	Gas Extraction Area (Within 1 km of Active Gas Well)	Groundwater Type (If Determined)	Alkalinity (mg/L)	Aluminum (mg/L)	Arsenic (mg/L)	Barium (mg/L)	Boron (mg/L)	Bromide (mg/L)	Cadmium (mg/L)	Calcium (mg/L)	Chloride (mg/L)	Chromium (mg/L)	Conductivity - Field (µs/cm)
459	6/13/2011	Outside Cold Water Hose Bib	Upland	Yes	Calcium-Bicarbonate	180	< .03	< .001	0.099		< .2	< .001	43	< 1	< .002	0.263
460	6/16/2011	Bailed Directly From Well	Upland		Calcium-Bicarbonate	38	0.25	0.0014	0.038		< .2	< .001	11	2.4	< .002	0.087
461	2/26/2009	Mary Steele Kitchen Sink	Valley				< .05		0.058					23.4		
462	6/16/2011		Upland			75	<0.03	<0.001	0.066		<0.2	<0.001	21		<0.002	
463	6/20/2011	Kitchen Sink Cwt On Nw Side Of House	Upland		Calcium-Bicarbonate	58	< .03	< .001	0.11		< .2	< .001	21	5	< .002	0.214
464	6/20/2011	Well	Upland		Calcium-Bicarbonate	39	< .03	< .001	0.082		< .2	< .001	15	9.4	< .002	0.193
465	1/25/2009		Valley				< .025		0.036					3.74		
466	6/20/2011		Upland	Yes	Calcium-Sodium-Bicarbonate	150	< .03	0.0015	0.11		< .2	< .001	30	2.4	0.002	
467	6/21/2011	At Pressure Tank In Basement	Upland	Yes	Calcium-Bicarbonate-Chloride	54	< .03	< .001	0.074		< .2	< .001	20	23	< .002	174
468	6/22/2011	Inside Hose Bib Located In The Garage	Valley		Calcium-Bicarbonate	75	<0.03	<0.001	0.1		<0.2	<0.001	24	1.3	<0.002	0.15
469	4/1/2009		Valley				< .05		0.101					30		
470	6/22/2011	Outside Hose	Valley		Calcium-Bicarbonate	110	< .03	< .001	0.1		< .2	< .001	27	3.7	< .002	0.185
471	6/22/2011	Well	Valley	Yes	Calcium-Bicarbonate	180	< .03	< .001	0.044		< .2	< .001	41	< 1	< .002	0.297
472	6/22/2011	Kitchen Sink Cwt	Valley		Calcium-Bicarbonate	91	< .03	< .001	0.078		< .2	< .001	21	< 1	< .002	0.141
473	6/23/2011	Pipe In Basement	Upland		Calcium-Bicarbonate	26	0.034	0.0011	0.027		< .2	< .001	6.9	< 1	< .002	0.163
474	4/1/2009		Valley				< .05		0.183					135		
475	6/23/2011	Outside Spigot	Valley		Calcium-Sodium-Bicarbonate	130	< .03	0.0011	0.14		< .2	< .001	30	4.9	< .002	0.225
476	4/15/2009	Kitchen Sink	Upland				< .1		0.322					134		
477	6/24/2011	Kitchen Sink	Valley		Calcium-Bicarbonate	110	0.6	0.001	0.19		< .2	< .001	31	1.2	< .002	0.135
478	6/24/2011	Outside Hose Bib On The W End Of Home	Valley	Yes	Calcium-Bicarbonate-Chloride	89	< .03	< .001	0.015		< .2	< .001	38	49	< .002	0.287
479	4/15/2009	Kitchen Sink	Upland				< .1		0.355					42.2		

Table S.8. Groundwater Quality Data for 1701 "Pre-Drill" Water Well Samples from Susquehanna County, Pennsylvania

Water Well ID	Sample Date	Sampling Port	Topo-graphic Location	Gas Extraction Area (Within 1 km of Active Gas Well)	Groundwater Type (If Determined)	Alkalinity (mg/L)	Aluminum (mg/L)	Arsenic (mg/L)	Barium (mg/L)	Boron (mg/L)	Bromide (mg/L)	Cadmium (mg/L)	Calcium (mg/L)	Chloride (mg/L)	Chromium (mg/L)	Conductivity - Field (µs/cm)
480	3/16/2009	Pressure Tank	Upland				< .05		0.16					3.5		268
481	6/27/2011	Sample Collected From An Outside Spigot Located On The North Side Of The House.	Valley		Calcium-Sodium-Bicarbonate	100	< .03	0.0063	0.23		< .2	< .001	18	< 1	< .002	0.168
482	6/27/2011	Kitchen Sink	Valley		Calcium-Sodium-Bicarbonate	82	< .03	< .001	0.21		< .2	< .001	20	2.8	< .002	0.106
483	6/28/2011	Dip Sample Directly From Well. Well Is Locatedon The South End Of Property Inside Of Electric Fence. It Is Covered With Metal Sheeting And An Old Car Hood Frame.	Upland		Calcium-Sodium-Bicarbonate-Chloride	120	0.048	0.0014	0.069		0.27	< .001	29	57	< .002	0.403
484	6/28/2011	Kitchen Sink Cwt	Valley		Calcium-Sodium-Bicarbonate	110	< .03	< .001	0.036		< .2	< .001	20	1.4	< .002	0.24
485	6/29/2011	Well	Upland		Calcium-Bicarbonate	40	0.35	0.0021	0.044		< .2	< .001	14	2.3	< .002	0.148
486	6/29/2011	Laundry Room Sink	Valley		Calcium-Bicarbonate	110	< .03	0.004	0.14		< .2	< .001	26	2.5	< .002	0.18
487	6/29/2011	Dug Well	Valley		Calcium-Bicarbonate-Chloride	26	< .03	< .001	0.031		< .2	< .001	11	4.2	< .002	0.072
488	6/29/2011		Valley		Calcium-Bicarbonate	40	< .03	< .001	0.039		< .2	< .001	15	3.1	< .002	
489	6/30/2011	Pressure Tank	Upland		Calcium-Bicarbonate	56	< .03	< .001	0.033		< .2	< .001	18	2	< .002	0.109
490	7/1/2011	Outside Hose Bib	Valley		Calcium-Sodium-Bicarbonate	140	< .03	0.0023	0.16		< .2	< .001	26	1.8	< .002	0.239
491	2/12/2009		Valley				0.048		0.15					2.65		
492	5/7/2009		Valley				0.158		0.073					6.42		
493	4/26/2009		Valley				< .05		0.568					910		

Table S.8. Groundwater Quality Data for 1701 "Pre-Drill" Water Well Samples from Susquehanna County, Pennsylvania

Water Well ID	Sample Date	Sampling Port	Topo-graphic Location	Gas Extraction Area (Within 1 km of Active Gas Well)	Groundwater Type (If Determined)	Alkalinity (mg/L)	Aluminum (mg/L)	Arsenic (mg/L)	Barium (mg/L)	Boron (mg/L)	Bromide (mg/L)	Cadmium (mg/L)	Calcium (mg/L)	Chloride (mg/L)	Chromium (mg/L)	Conductivity - Field (µs/cm)
494	7/5/2011	Kitchen Sink	Valley		Calcium-Bicarbonate	110	< .03	< .001	0.073		< .2	< .001	32	4.3	< .002	0.175
495	7/5/2011	Samples Collected By Dipping 1 Inch Plastic Bailer Down The Well.	Upland		Calcium-Bicarbonate	100	12	0.023	0.2		< .2	< .001	27	1.6	0.016	0.181
496	7/5/2011	Outside Hose Bib On South Of House	Valley		Calcium-Bicarbonate	180	< .03	0.023	0.12		< .2	< .001	52	7.7	< .002	0.278
497	7/6/2011	Pressure Tank In Basement	Upland		Calcium-Bicarbonate	130	< .03	< .001	0.11		< .2	< .001	41	9.6	< .002	0.231
498	7/8/2011	Outside Faucet	Upland		Sodium-Bicarbonate	55	0.045	< .001	< .01		< .2	< .001	< .1	5.8	< .002	0.123
499	5/14/2011	Kitchen Sink	Valley	Yes	Calcium-Bicarbonate	170	< .03	0.0012	0.088		< .2	< .001	39	12	< .002	286
500	5/3/2009	Bathroom Sink	Upland				< .05		0.103					16.1		
501	5/19/2011	Outside Spigot	Valley	Yes	Calcium-Sodium-Bicarbonate-Chloride	58	< .03	< .001	0.047		< .2	< .001	27	32	< .002	0.207
502	5/25/2011	Pressure Tank	Valley	Yes	Calcium-Bicarbonate	180	< .03	< .001	0.07		< .2	< .001	46	18	< .002	0.31
503	5/26/2011	Pressure Tank	Valley	Yes	Calcium-Bicarbonate	170	< .03	< .001	0.016		< .2	< .001	56	28	< .002	0.359
504	10/26/2009		Valley				< .1		0.164					5.89		
505	5/26/2011	Outside Hose Bib Cold	Valley	Yes	Calcium-Bicarbonate	100	< .03	< .001	0.058		< .2	< .001	40	23	< .002	0.246
506	10/26/2009		Valley	Yes			< .1		0.237					9.39		
507	6/1/2011	Outside Hose	Upland		Calcium-Bicarbonate	200	< .03	0.0013	0.047		< .2	< .001	45	< 1	0.0064	0.275
508	6/1/2011	Pressure Tank	Valley		Calcium-Bicarbonate	140	< .03	< .001	0.022		< .2	< .001	37	3.7	< .002	0.195
509	6/2/2011	Pressure Tank In Old Creamery	Upland		Calcium-Bicarbonate	73	< .03	< .001	0.051		< .2	< .001	24	2.5	< .002	0.131
510	10/26/2009	Pressure Tank	Valley	Yes			< .1		0.096					< 2		
511	6/6/2011	Dug Well 20 Feet From Se Corner Of House	Upland		Calcium-Bicarbonate	170	< .03	0.0032	0.039		< .2	< .001	71	37	< .002	333
512	10/26/2009	Pressure Tank	Upland	Yes			< .1		0.101					30.5		

Table S.8. Groundwater Quality Data for 1701 "Pre-Drill" Water Well Samples from Susquehanna County, Pennsylvania

Water Well ID	Sample Date	Sampling Port	Topo-graphic Location	Gas Extraction Area (Within 1 km of Active Gas Well)	Groundwater Type (If Determined)	Alkalinity (mg/L)	Aluminum (mg/L)	Arsenic (mg/L)	Barium (mg/L)	Boron (mg/L)	Bromide (mg/L)	Cadmium (mg/L)	Calcium (mg/L)	Chloride (mg/L)	Chromium (mg/L)	Conductivity - Field (µs/cm)
513	6/13/2011	Kitchen Sink	Valley													245
514	6/13/2011	Hand Dug Well	Upland													151
515	6/13/2011	Main Kitchen Sink	Upland													200
516	6/13/2011	Basement Pressure Tank	Valley													185
517	6/13/2011	Exterior Faucet On South Side Of House Between Two Green Garage Doors	Upland													156
518	6/14/2011	Kitchen Sink	Valley													0.247
519	6/14/2011	Kitchen Sink	Upland													242
520	6/14/2011	Pressure Tank	Upland													197
521	6/14/2011	Kitchen Sink	Valley													146
522	10/26/2009	Basement At Pressure Tank	Upland	Yes			< .1		0.141					2.42		
523	6/14/2011	Kitchen Faucet	Valley													
524	6/14/2011	Kitchen Sink	Valley													216
525	6/14/2011	Kitchen Sink	Upland													124
526	6/14/2011	Kitchen Sink	Valley													192
527	6/14/2011	Pressure Tank	Valley													213
528	6/15/2011		Upland													
529	6/15/2011	Kitchen Sink	Upland													87
530	6/15/2011	Spigot At Well House	Upland													0.34
531	6/15/2011	Kitchen Sink	Upland													253
532	6/15/2011	Kitchen Sink	Upland													51
533	6/15/2011	Kitchen Sink	Upland													325

Table S.8. Groundwater Quality Data for 1701 "Pre-Drill" Water Well Samples from Susquehanna County, Pennsylvania

Water Well ID	Sample Date	Sampling Port	Topo-graphic Location	Gas Extraction Area (Within 1 km of Active Gas Well)	Groundwater Type (If Determined)	Alkalinity (mg/L)	Aluminum (mg/L)	Arsenic (mg/L)	Barium (mg/L)	Boron (mg/L)	Bromide (mg/L)	Cadmium (mg/L)	Calcium (mg/L)	Chloride (mg/L)	Chromium (mg/L)	Conductivity - Field (µs/cm)
534	6/16/2011	Kitchen Sink	Upland													201
535	6/16/2011	Pressure Tank	Valley													233
536	6/16/2011	Kitchen Sink Overlooking Blanding Road	Upland													117
537	6/16/2011	Kitchen Sink On South Side Of House	Upland													473
538	6/16/2011	Kitchen Sink	Upland													143
539	6/16/2011	Kitchen Sink	Upland													200
540	6/16/2011	Wellhead From Hose	Upland													319
541	6/16/2011	Kitchen Sink	Valley													307
542	6/17/2011	Kitchen Sink	Upland													150
543	6/17/2011	Pressure Tank In Basement On Tingley Lake Side Of Road	Valley													289
544	6/20/2011	Pressure Tank	Valley													192
545	6/21/2011	Kitchen Sink	Valley													140
546	6/21/2011	Kitchen Sink	Upland													177
547	6/21/2011	Kitchen Sink	Upland													81
548	4/26/2009	Kitchen Sink	Valley				< .05		0.143					6.07		
549	6/21/2011	Kitchen Sink	Upland													178
550	6/21/2011		Valley													
551	6/22/2011	Kitchen Sink	Upland													127
552	6/22/2011	Basement Pressure Tank	Valley													235
553	6/22/2011	Kitchen Sink	Upland													201
554	6/22/2011	Kitchen Sink	Valley													105

Table S.8. Groundwater Quality Data for 1701 "Pre-Drill" Water Well Samples from Susquehanna County, Pennsylvania

Water Well ID	Sample Date	Sampling Port	Topo-graphic Location	Gas Extraction Area (Within 1 km of Active Gas Well)	Groundwater Type (If Determined)	Alkalinity (mg/L)	Aluminum (mg/L)	Arsenic (mg/L)	Barium (mg/L)	Boron (mg/L)	Bromide (mg/L)	Cadmium (mg/L)	Calcium (mg/L)	Chloride (mg/L)	Chromium (mg/L)	Conductivity - Field (µs/cm)
555	6/22/2011		Upland													
556	6/22/2011	Kitchen Sink	Upland													188
557	6/23/2011	Pressure Tank	Valley													231
558	6/23/2011	Pressure Tank	Valley													235
559	6/23/2011	Kitchen Sink	Valley													156
560	6/23/2011	Kitchen Sink	Valley													198
561	6/23/2011		Valley													
562	6/23/2011		Upland													
563	10/27/2009	Kitchen Sink - Ann'S House	Valley	Yes			< .1		0.065					29.1		
564	6/24/2011	Spigot	Upland													213
565	6/24/2011	Kitchen Sink	Upland													258
566	6/24/2011	Kitchen Sink	Upland													102
567	6/24/2011	Kitchen Sink	Upland													166
568	6/24/2011	Kitchen Sink	Valley													212
569	6/24/2011	Kitchen Sink	Valley													242
570	6/24/2011	Kitchen Sink	Upland													148
571	6/24/2011	Kitchen Sink	Upland													136
572	6/24/2011		Upland													
573	6/27/2011	Kitchen Sink	Valley													239
574	6/27/2011		Valley													
575	6/27/2011	House Kitchen Sink	Valley													242
576	6/27/2011		Valley													81
577	6/27/2011	Kitchen Sink	Upland													203
578	6/27/2011	Kitchen Sink	Upland													145
579	6/27/2011	Outside Spigot	Valley													282
580	6/28/2011	Kitchen Sink	Valley													230
581	6/28/2011		Upland													
582	6/28/2011	Outside Spigot	Upland													216
583	6/28/2011		Valley													

Table S.8. Groundwater Quality Data for 1701 "Pre-Drill" Water Well Samples from Susquehanna County, Pennsylvania

Water Well ID	Sample Date	Sampling Port	Topo-graphic Location	Gas Extraction Area (Within 1 km of Active Gas Well)	Groundwater Type (If Determined)	Alkalinity (mg/L)	Aluminum (mg/L)	Arsenic (mg/L)	Barium (mg/L)	Boron (mg/L)	Bromide (mg/L)	Cadmium (mg/L)	Calcium (mg/L)	Chloride (mg/L)	Chromium (mg/L)	Conductivity - Field (µs/cm)
584	6/28/2011	Kitchen Sink	Upland													253
585	6/28/2011		Valley													
586	10/27/2009	Pressure Tank	Upland				< .1		0.071					19.6		
587	6/28/2011	Kitchen Sink	Valley													230
588	6/28/2011	Basement Pressure Tank	Upland													394
589	6/29/2011	Well Spigot	Upland													342
590	6/29/2011		Valley													
591	6/29/2011		Upland													
592	6/29/2011		Valley													
593	6/29/2011		Valley													
594	6/29/2011		Upland													
595	6/29/2011	House Spigot	Valley													237
596	6/29/2011	Kitchen Sink (Dennis)	Upland													275
597	6/30/2011	Kitchen Sink	Valley													213
598	10/27/2009	Pressure Tank	Valley				< .1		0.165					5.12		
599	6/30/2011	Pressure Tank	Valley													154
600	6/30/2011		Valley													
601	6/30/2011	Kitchen Sink	Upland													94
602	6/30/2011	Pressure Tank	Valley													343
603	6/30/2011		Valley													
604	6/30/2011		Valley													
605	6/30/2011		Valley													
606	6/30/2011	Sink In Bays Of Garage	Upland													347
607	10/27/2009	Pressure Tank	Upland				< .1		0.182					7.01		
608	6/30/2011	Basement Pressure Tank	Valley													160
609	6/30/2011	Outside Spigot	Upland													145
610	6/30/2011		Upland													130
611	6/30/2011	Kitchen Sink	Upland													
612	6/30/2011	Sink In Barn	Valley													285
613	7/1/2011	Kitchen Sink	Upland													87

Table S.8. Groundwater Quality Data for 1701 "Pre-Drill" Water Well Samples from Susquehanna County, Pennsylvania

Water Well ID	Sample Date	Sampling Port	Topo-graphic Location	Gas Extraction Area (Within 1 km of Active Gas Well)	Groundwater Type (If Determined)	Alkalinity (mg/L)	Aluminum (mg/L)	Arsenic (mg/L)	Barium (mg/L)	Boron (mg/L)	Bromide (mg/L)	Cadmium (mg/L)	Calcium (mg/L)	Chloride (mg/L)	Chromium (mg/L)	Conductivity - Field (µs/cm)
614	7/1/2011	Kitchen Sink	Upland													77
615	7/1/2011	Kitchen Sink	Upland													
616	7/1/2011	Kitchen Sink	Upland													87
617	7/1/2011	Kitchen Sink	Valley													193
618	7/1/2011	Outside Spigot	Upland													186
619	7/1/2011		Valley													
620	7/1/2011	Outside Spigot	Valley													
621	7/1/2011	Wellhead Spigot	Valley													
622	7/5/2011	Bathroom Sink	Valley													177
623	7/5/2011	Kitchen Sink	Upland													217
624	7/5/2011	Basement Pressure Tank	Upland													0.143
625	7/5/2011		Valley													
626	7/5/2011	Kitchen Sink	Valley													168
627	10/27/2009	Pressure Tank	Valley				< .1		0.851					19.4		
628	7/5/2011	Kitchen Sink	Upland													224
629	7/5/2011	Outside Spigot	Valley													
630	7/5/2011	Kitchen Sink	Upland													
631	7/5/2011	Kitchen Sink	Valley													
632	7/5/2011	Outside Spigot	Valley													
633	7/5/2011	Kitchen Sink	Valley													
634	7/5/2011	Kitchen Sink	Valley													95
635	7/5/2011	Kitchen Sink Main Camp	Valley													122
636	7/5/2011	Pressure Tank	Valley													0.25
637	7/5/2011	Kitchen Sink	Upland													
638	7/5/2011	Kitchen Faucet	Valley													

Table S.8. Groundwater Quality Data for 1701 "Pre-Drill" Water Well Samples from Susquehanna County, Pennsylvania

Water Well ID	Sample Date	Sampling Port	Topo-graphic Location	Gas Extraction Area (Within 1 km of Active Gas Well)	Groundwater Type (If Determined)	Alkalinity (mg/L)	Aluminum (mg/L)	Arsenic (mg/L)	Barium (mg/L)	Boron (mg/L)	Bromide (mg/L)	Cadmium (mg/L)	Calcium (mg/L)	Chloride (mg/L)	Chromium (mg/L)	Conductivity - Field (µs/cm)
639	7/5/2011	Kitchen Sink	Valley													271
640	7/5/2011	Kitchen Sink	Upland													266
641	7/5/2011	Kitchen Sink	Upland													320
642	7/6/2011	Kitchen Sink	Upland													300
643	7/6/2011	Kitchen Sink	Valley													183
644	7/6/2011	Kitchen Sink	Upland													231
645	7/6/2011		Upland													
646	7/6/2011	Farm Sink	Valley													
647	7/6/2011		Upland													
648	7/6/2011	Kitchen Sink	Valley													249
649	7/6/2011		Valley													
650	7/6/2011	Kitchen Sink	Valley													170
651	7/6/2011	Kitchen Sink	Valley													175
652	7/6/2011	Basement Pressure Tank	Valley													154
653	7/6/2011	House Well	Upland													186
654	7/6/2011	Outside Spigot	Valley													276
655	7/7/2011	Pressure Tank In Basement	Upland													260
656	7/7/2011	Kitchen Sink	Upland													0.181
657	7/7/2011	Outside Faucet	Upland													162
658	7/7/2011	Kitchen Sink	Valley													142
659	7/7/2011	Kitchen Sink	Upland													149
660	7/7/2011	Kitchen Sink	Upland													
661	7/7/2011	Outside Faucet	Valley													
662	7/7/2011	Garage Spigot	Valley													
663	7/7/2011	Kitchen Sink	Valley													97
664	7/7/2011	Pressure Tank	Upland													193
665	7/7/2011	Kitchen Sink	Upland													131

Table S.8. Groundwater Quality Data for 1701 "Pre-Drill" Water Well Samples from Susquehanna County, Pennsylvania

Water Well ID	Sample Date	Sampling Port	Topo-graphic Location	Gas Extraction Area (Within 1 km of Active Gas Well)	Groundwater Type (If Determined)	Alkalinity (mg/L)	Aluminum (mg/L)	Arsenic (mg/L)	Barium (mg/L)	Boron (mg/L)	Bromide (mg/L)	Cadmium (mg/L)	Calcium (mg/L)	Chloride (mg/L)	Chromium (mg/L)	Conductivity - Field (µs/cm)
666	7/7/2011	Kitchen Sink	Valley													
667	7/7/2011	Kitchen Sink	Upland													108
668	7/7/2011	Kitchen Sink	Upland													255
669	7/7/2011	Bunk House Sink	Upland													0.261
670	7/7/2011	Spigot At Well Head	Valley													143
671	7/8/2011	Kitchen Sink	Valley													207
672	7/8/2011	Kitchen Faucet	Upland													184
673	7/8/2011	Pressure Tank	Valley													
674	7/8/2011	Kitchen Faucet	Upland													
675	7/8/2011	Kitchen Faucet	Upland													76
676	7/8/2011	Kitchen Sink	Upland													154
677	7/8/2011	Kitchen Sink	Upland													220
678	7/8/2011	Kitchen Sink	Valley													229
679	7/8/2011	Spigot In Cooling Barn	Upland													211
680	7/8/2011	Kitchen Sink	Upland													293
681	7/8/2011	Shop Sink	Upland													103
682	7/8/2011	Kitchen Sink	Upland													161
683	7/9/2011	Kitchen Sink	Upland													
684	7/11/2011	Spigot On Front Of House	Valley													364
685	7/11/2011	Spigot Near Water Fountain	Valley													265
686	7/11/2011	Outside Spigot	Upland													
687	7/11/2011	Kitchen Sink	Valley													
688	7/11/2011	Kitchen Sink	Valley													
689	7/11/2011	Kitchen Sink	Valley													162

Table S.8. Groundwater Quality Data for 1701 "Pre-Drill" Water Well Samples from Susquehanna County, Pennsylvania

Water Well ID	Sample Date	Sampling Port	Topo-graphic Location	Gas Extraction Area (Within 1 km of Active Gas Well)	Groundwater Type (If Determined)	Alkalinity (mg/L)	Aluminum (mg/L)	Arsenic (mg/L)	Barium (mg/L)	Boron (mg/L)	Bromide (mg/L)	Cadmium (mg/L)	Calcium (mg/L)	Chloride (mg/L)	Chromium (mg/L)	Conductivity - Field (µs/cm)
690	11/5/2009	Pressure Tank	Upland	Yes			< .1		0.084					11		
691	7/11/2011	Kitchen Sink	Upland													224
692	7/11/2011	Kitchen Sink	Upland													149
693	7/11/2011	Kitchen Sink	Upland													162
694	7/11/2011	Hand Dug Well	Upland													151
695	7/11/2011	Kitchen Sink	Valley													637
696	7/12/2011	Bathroom Sink Downstairs	Valley													
697	7/12/2011	Kitchen Sink	Upland													
698	7/12/2011	Kitchen Faucet	Valley													195
699	7/12/2011	Kitchen Sink	Valley													185
700	7/12/2011	Kitchen Sink	Valley													415
701	7/12/2011	Pressure Tank	Upland													250
702	7/12/2011	Kitchen Sink	Upland													
703	7/12/2011	Spigot Before Filter	Valley													
704	7/12/2011	Kitchen Sink	Valley													
705	7/12/2011		Valley													
706	7/12/2011	Pressure Tank	Upland													153
707	7/12/2011	Inside Faucet	Upland													217
708	7/12/2011	Kitchen Sink	Upland													191
709	7/12/2011	Spigot	Upland													240
710	7/12/2011	Spigot	Upland													257
711	7/12/2011	Kitchen Sink	Valley													153
712	7/12/2011	Kitchen Faucet	Upland													163
713	7/12/2011	Kitchen Faucet	Valley													118
714	7/13/2011	Kitchen Sink	Valley													247
715	7/13/2011	Kitchen Sink	Valley													172

Table S.8. Groundwater Quality Data for 1701 "Pre-Drill" Water Well Samples from Susquehanna County, Pennsylvania

Water Well ID	Sample Date	Sampling Port	Topo-graphic Location	Gas Extraction Area (Within 1 km of Active Gas Well)	Groundwater Type (If Determined)	Alkalinity (mg/L)	Aluminum (mg/L)	Arsenic (mg/L)	Barium (mg/L)	Boron (mg/L)	Bromide (mg/L)	Cadmium (mg/L)	Calcium (mg/L)	Chloride (mg/L)	Chromium (mg/L)	Conductivity - Field (µs/cm)
716	7/13/2011	Kitchen Sink	Upland													202
717	7/13/2011	Spigot	Valley													191
718	7/13/2011	Spigot On Well	Valley													442
719	7/13/2011	Barn Spigot	Valley													221
720	7/13/2011	Kitchen Sink	Valley													247
721	7/13/2011	Garage Spigot	Upland													351
722	7/13/2011	Kitchen Sink	Valley													252
723	7/13/2011	Kitchen Sink	Valley													231
724	7/8/2011		Upland													
725	7/8/2011	Kitchen Sink	Upland													
726	4/13/2011	Milkhouse Faucet Barn Well	Valley		Sodium-Bicarbonate	165	< .01	< .001	0.33			< .0001	4	32.7	< .002	326
727	4/13/2011	Tenant'S Pressure Tank	Upland		Calcium-Bicarbonate-Chloride	30	0.023	< .001	0.08			< .0001	12	11.4	< .002	113
728	4/13/2011	Outside Spigot On Garage	Upland		Calcium-Sodium-Bicarbonate	130	0.54	0.0098	0.21			< .0001	27	19.7	< .002	
729	4/26/2011	Pressure Tank	Valley		Calcium-Bicarbonate	160	0.059	0.0029	0.19			0.0001	56	12.1	0.0042	280
730	6/17/2011	Spigot On Front Of House	Upland													183
731	6/17/2011	Kitchen Sink	Valley													195
732	6/17/2011	Spigot Inside Of Shed On Right	Upland													258
733	6/17/2011	Kitchen Sink	Valley													329
734	6/17/2011	Pressure Tank	Valley													280
735	6/20/2011	Kitchen Sink	Upland													235
736	6/20/2011	Pressure Tank	Valley													318
737	6/20/2011	Kitchen Sink	Upland													269
738	6/20/2011	Kitchen Sink	Valley													326
739	6/20/2011	Kitchen Sink	Valley													247

Table S.8. Groundwater Quality Data for 1701 "Pre-Drill" Water Well Samples from Susquehanna County, Pennsylvania

Water Well ID	Sample Date	Sampling Port	Topo-graphic Location	Gas Extraction Area (Within 1 km of Active Gas Well)	Groundwater Type (If Determined)	Alkalinity (mg/L)	Aluminum (mg/L)	Arsenic (mg/L)	Barium (mg/L)	Boron (mg/L)	Bromide (mg/L)	Cadmium (mg/L)	Calcium (mg/L)	Chloride (mg/L)	Chromium (mg/L)	Conductivity - Field (µs/cm)
740	6/20/2011	Kitchen Sink	Valley													314
741	6/21/2011	Kitchen Sink	Upland													179
742	6/21/2011	Kitchen Sink	Valley													232
743	6/21/2011	Kitchen Sink	Valley													223
744	6/21/2011	Kitchen Sink	Valley													203
745	6/21/2011		Valley													
746	6/21/2011	Outside Spigot	Valley													206
747	6/21/2011	Kitchen Sink	Valley													226
748	6/21/2011	Wellhead Spigot	Valley													195
749	6/21/2011	Kitchen Sink	Upland													186
750	6/21/2011		Upland													
751	6/29/2011	Kitchen Sink	Upland													81
752	6/29/2011	Barn Spigot	Valley													119
753	7/8/2011	Well 2 On South Side Of House	Upland													45
754	7/8/2011	Kitchen Sink	Upland													211
755	7/8/2011	Kichen Sink	Valley													
756	7/9/2011	Kitchen Sink	Valley													
757	7/9/2011	Spring	Upland													
758	7/11/2011	Kitchen Sink	Valley													
759	7/11/2011	Kitchen Sink	Valley													44
760	7/11/2011	Kitchen Sink	Valley													101
761	11/5/2009	Outside Faucet	Valley	Yes			< .1		0.305					5.75		
762	7/12/2011	Kitchen Sink	Valley													144
763	7/12/2011	Kitchen Sink	Valley													194
764	7/14/2011	Kitchen Sink	Upland													195
765	7/14/2011	Kitchen Sink	Upland													219
766	7/14/2011	Kitchen Sink	Valley													1344

Table S.8. Groundwater Quality Data for 1701 "Pre-Drill" Water Well Samples from Susquehanna County, Pennsylvania

Water Well ID	Sample Date	Sampling Port	Topo-graphic Location	Gas Extraction Area (Within 1 km of Active Gas Well)	Groundwater Type (If Determined)	Alkalinity (mg/L)	Aluminum (mg/L)	Arsenic (mg/L)	Barium (mg/L)	Boron (mg/L)	Bromide (mg/L)	Cadmium (mg/L)	Calcium (mg/L)	Chloride (mg/L)	Chromium (mg/L)	Conductivity - Field (µs/cm)
767	7/14/2011	Spigot On Side Of House	Valley													461
768	7/14/2011	Kitchen Sink	Valley													943
769	7/14/2011	Kitchen Sink	Valley													497
770	7/14/2011	Kitchen Sink	Valley													285
771	7/14/2011	Kitchen Sink	Upland													254
772	7/14/2011	Kitchen Sink	Valley													434
773	7/14/2011	Spigot	Valley													1291
774	7/15/2011	Kitchen Sink	Valley													191
775	7/15/2011	Kitchen Sink	Valley													259
776	7/15/2011	Kitchen Sink	Upland													190.1
777	7/15/2011	Kitchen Sink	Upland													212
778	7/15/2011	Outside Faucet	Valley													244
779	7/15/2011	Kitchen Sink	Valley													177
780	7/15/2011	Spigot East Of House	Valley													173
781	7/15/2011	Outside Faucet	Upland													131
782	7/15/2011	Outside Faucet	Upland													211
783	7/15/2011	Kitchen Sink	Upland													155
784	7/15/2011	Kitchen Sink	Valley													376
785	7/15/2011	Kitchen Sink	Valley													211
786	7/15/2011	Kitchen Sink	Valley													317
787	7/15/2011	Kitchen Sink	Valley													189
788	7/15/2011	Kitchen Sink	Valley													303
789	7/15/2011	Kitchen Sink	Valley													171.9
790	7/15/2011	Kitchen Sink	Valley													278
791	7/15/2011	Kitchen Sink	Upland													283

Table S.8. Groundwater Quality Data for 1701 "Pre-Drill" Water Well Samples from Susquehanna County, Pennsylvania

Water Well ID	Sample Date	Sampling Port	Topo-graphic Location	Gas Extraction Area (Within 1 km of Active Gas Well)	Groundwater Type (If Determined)	Alkalinity (mg/L)	Aluminum (mg/L)	Arsenic (mg/L)	Barium (mg/L)	Boron (mg/L)	Bromide (mg/L)	Cadmium (mg/L)	Calcium (mg/L)	Chloride (mg/L)	Chromium (mg/L)	Conductivity - Field (µs/cm)
792	7/15/2011	Spigot On Rear Of House	Valley													147
793	7/15/2011	Kitchen Sink	Valley													102.3
794	7/15/2011	Kitchen Sink	Valley													142
795	7/16/2011	Kitchen Faucet	Upland													153
796	7/16/2011	Kitchen Faucet	Valley													141
797	7/16/2011	Kitchen Faucet	Upland													75
798	7/16/2011	Kitchen Faucet	Upland													141
799	7/16/2011	Kitchen Sink	Upland													154
800	7/17/2011	Outside Spigot In Back Of House	Valley													143
801	7/17/2011	Outside Spigot On Front Of House	Valley													141
802	7/17/2011	Well	Upland													64
803	7/17/2011	Blue Well 40Ft From Road	Upland													140
804	7/17/2011	Kitchen Faucet	Upland													108
805	7/17/2011	Spigot From Garage	Valley													102
806	7/18/2011	Outside Spigot	Upland													142
807	7/18/2011	Kitchen Sink	Upland													88
808	7/18/2011	Outside Spigot	Upland													150
809	7/18/2011	Kitchen Sink	Valley													548
810	7/18/2011	Kitchen Sink	Upland													118
811	7/18/2011	Kitchen Sink	Valley													154
812	7/18/2011	Kitchen Sink	Valley													127
813	7/19/2011		Upland													
814	7/19/2011	Garage Sink	Valley													291
815	7/19/2011	Kitchen Sink	Valley													203

Table S.8. Groundwater Quality Data for 1701 "Pre-Drill" Water Well Samples from Susquehanna County, Pennsylvania

Water Well ID	Sample Date	Sampling Port	Topo-graphic Location	Gas Extraction Area (Within 1 km of Active Gas Well)	Groundwater Type (If Determined)	Alkalinity (mg/L)	Aluminum (mg/L)	Arsenic (mg/L)	Barium (mg/L)	Boron (mg/L)	Bromide (mg/L)	Cadmium (mg/L)	Calcium (mg/L)	Chloride (mg/L)	Chromium (mg/L)	Conductivity - Field (µs/cm)
816	7/19/2011	Barn 2 Pressure Tank	Valley													331
817	7/19/2011	Spigot On Side Of Barn	Upland													298
818	7/19/2011	Well Head	Valley													180.7
819	11/23/2009		Upland	Yes			< .1		0.064					10.3		
820	7/19/2011	Kitchen Sink	Valley													98
821	7/19/2011	Kitchen Sink	Valley													119.7
822	7/19/2011	Kitchen Sink	Valley													284
823	7/19/2011	Kitchen Sink	Valley													178.6
824	7/20/2011	Outside Faucet	Upland													281
825	7/20/2011	Inside Spigot	Upland													375
826	7/20/2011	Kitchen Sink	Upland													172
827	7/20/2011	Kitchen Sink	Valley													211
828	7/20/2011	Kitchen Sink	Upland													150
829	7/20/2011	Kitchen Sink	Valley													146
830	7/20/2011	Outside Spigot	Upland													159
831	7/20/2011	Kitchen Sink	Valley													113
832	7/20/2011	Kitchen Sink	Valley													214
833	7/20/2011	Outside Faucet	Valley													285
834	7/20/2011	Kitchen Sink	Upland													130
835	7/20/2011	Kitchen Sink	Upland													234
836	7/21/2011	Outside Faucet	Upland													267
837	7/21/2011	Spigot In Front Of House	Upland													156
838	7/21/2011	Kitchen Sink	Valley													320
839	7/21/2011	Kitchen Sink	Upland													292
840	7/21/2011	Kitchen Sink	Upland													140

Table S.8. Groundwater Quality Data for 1701 "Pre-Drill" Water Well Samples from Susquehanna County, Pennsylvania

Water Well ID	Sample Date	Sampling Port	Topo-graphic Location	Gas Extraction Area (Within 1 km of Active Gas Well)	Groundwater Type (If Determined)	Alkalinity (mg/L)	Aluminum (mg/L)	Arsenic (mg/L)	Barium (mg/L)	Boron (mg/L)	Bromide (mg/L)	Cadmium (mg/L)	Calcium (mg/L)	Chloride (mg/L)	Chromium (mg/L)	Conductivity - Field (µs/cm)
841	7/21/2011	Kitchen Sink	Valley													299
842	7/21/2011	Kitchen Sink	Valley													146
843	7/21/2011	Kitchen Sink	Upland													77
844	7/21/2011	Pressure Tank	Upland													41.7
845	7/21/2011	Basement Sink	Upland													170
846	7/21/2011	Kitchen Sink	Upland													250
847	7/21/2011	Spigot In Rear Of House	Upland													
848	7/21/2011	Kitchen Sink	Valley													494
849	7/21/2011	Kitchen Sink	Valley													306
850	7/22/2011	Kitchen Sink	Valley													147.6
851	7/22/2011	Outside Spigot	Valley													349
852	7/22/2011	Kitchen Sink	Valley													264
853	7/22/2011	Kitchen Sink	Valley													236
854	7/22/2011	Kitchen Sink	Valley													383
855	7/22/2011	Pipe Overflow	Valley													288
856	7/22/2011	Kitchen Sink	Valley													201
857	7/22/2011	Pressure Tank In Garage	Valley													481
858	11/5/2009	Basement At Pressure Tank	Valley	Yes			< .1		0.169					4.53		
859	7/22/2011	Outside Spigot	Valley													139
860	7/22/2011	Kitchen Faucet	Valley													157
861	7/22/2011	Spigot Under Porch	Valley													163
862	7/22/2011	Kitchen Sink	Valley													736
863	7/22/2011	Pressure Tank In Garage	Valley													668

Table S.8. Groundwater Quality Data for 1701 "Pre-Drill" Water Well Samples from Susquehanna County, Pennsylvania

Water Well ID	Sample Date	Sampling Port	Topo-graphic Location	Gas Extraction Area (Within 1 km of Active Gas Well)	Groundwater Type (If Determined)	Alkalinity (mg/L)	Aluminum (mg/L)	Arsenic (mg/L)	Barium (mg/L)	Boron (mg/L)	Bromide (mg/L)	Cadmium (mg/L)	Calcium (mg/L)	Chloride (mg/L)	Chromium (mg/L)	Conductivity - Field (µs/cm)
864	7/22/2011	Kitchen Sink	Upland													130.7
865	7/22/2011	Kitchen Sink	Upland													142.1
866	7/22/2011	Kitchen Sink	Upland													137.9
867	11/5/2009	Kitchen Sink	Upland	Yes			< .1		< .025					12.3		
868	7/22/2011	Kitchen Sink	Valley													1310
869	7/22/2011	Pressure Tank	Valley													598
870	7/25/2011	Kitchen Sink	Valley													146
871	7/25/2011	Kitchen Sink	Valley													170
872	7/25/2011	Kitchen Sink	Upland													153
873	7/25/2011	Kitchen Sink	Upland													171
874	7/25/2011	Kitchen Sink	Upland													328
875	7/25/2011	Kitchen Sink	Upland													152
876	7/25/2011	Warehouse At Quarry	Upland													135
877	7/25/2011	Kitchen Sink	Valley													284
878	7/25/2011	Kitchen Sink	Upland													234
879	7/25/2011	Kitchen Sink	Valley													730
880	1/3/2010	Pressure Tank	Upland	Yes			< .1		0.117					23.3		
881	7/25/2011	Kitchen Sink	Valley													357
882	7/25/2011	Pressure Tank	Valley													756
883	7/26/2011	Kitchen Sink	Valley													275
884	7/26/2011	Kitchen Sink	Valley													137
885	7/26/2011	Kitchen Sink	Valley													155
886	7/26/2011	Kitchen Sink	Valley													166
887	7/26/2011	Kitchen Sink	Valley													142
888	12/22/2009	Pressure Tank	Upland	Yes			< .1		0.053					4.33		128.6

Table S.8. Groundwater Quality Data for 1701 "Pre-Drill" Water Well Samples from Susquehanna County, Pennsylvania

Water Well ID	Sample Date	Sampling Port	Topo-graphic Location	Gas Extraction Area (Within 1 km of Active Gas Well)	Groundwater Type (If Determined)	Alkalinity (mg/L)	Aluminum (mg/L)	Arsenic (mg/L)	Barium (mg/L)	Boron (mg/L)	Bromide (mg/L)	Cadmium (mg/L)	Calcium (mg/L)	Chloride (mg/L)	Chromium (mg/L)	Conductivity - Field (µs/cm)
889	7/26/2011	Kitchen Sink	Upland													153
890	7/26/2011	Pipe From Well	Valley													322
891	7/26/2011	Outside Spigot	Upland													228
892	7/26/2011	Outside Spigot	Valley													220
893	7/26/2011	Outside Spigot	Upland													238
894	7/26/2011	Kitchen Sink	Valley													184.8
895	12/15/2009	Pressure Tank	Upland				0.141		0.22					10.2		
896	7/26/2011	Outside Spigot	Upland													166.9
897	7/26/2011	Kitchen Sink	Valley													147.8
898	7/26/2011	Outside Spigot	Upland													202
899	7/26/2011	Kitchen Sink	Upland													50
900	7/26/2011	Kitchen Sink	Upland													132.8
901	7/27/2011	Kitchen Sink	Upland													91
902	7/27/2011	Outside Faucet	Valley													142
903	7/27/2011	Basement Spigot	Valley													247
904	12/17/2009	Pressure Tank	Valley	Yes			0.127		1.31					5.62		224
905	7/27/2011	Kitchen Sink	Valley													171
906	7/27/2011	Kitchen Sink	Valley													207
907	7/27/2011	Kitchen Sink	Upland													158
908	7/27/2011	Pipe Overflow	Valley													91
909	7/27/2011	Pressure Tank	Upland													93.2
910	7/27/2011	Kitchen Sink	Upland													59
911	7/27/2011	Kitchen Sink	Upland													62.8
912	7/27/2011	Outside Spigot	Valley													182.4
913	7/27/2011	Kitchen Sink	Valley													208

Table S.8. Groundwater Quality Data for 1701 "Pre-Drill" Water Well Samples from Susquehanna County, Pennsylvania

Water Well ID	Sample Date	Sampling Port	Topo-graphic Location	Gas Extraction Area (Within 1 km of Active Gas Well)	Groundwater Type (If Determined)	Alkalinity (mg/L)	Aluminum (mg/L)	Arsenic (mg/L)	Barium (mg/L)	Boron (mg/L)	Bromide (mg/L)	Cadmium (mg/L)	Calcium (mg/L)	Chloride (mg/L)	Chromium (mg/L)	Conductivity - Field (µs/cm)
914	7/27/2011	Kitchen Sink	Upland													230
915	7/27/2011	Kitchen Sink	Upland													166.8
916	7/28/2011	Kitchen Sink	Upland													222
917	7/28/2011	Kitchen Sink	Valley													111
918	7/28/2011	Kitchen Sink	Upland													83
919	7/28/2011	Kitchen Sink	Upland													44
920	7/28/2011	Kitchen Sink	Valley													101
921	7/28/2011	Outside Spigot	Valley	Yes												825
922	7/29/2011	Kitchen Sink	Valley													114
923	12/14/2009	Basement At Pressure Tank	Valley	Yes			< .1		0.062					7.82		
924	12/17/2009	Kitchen Sink	Valley				< .1		0.195					7.04		327
925	12/13/2009	Pressure Tank	Valley				< .1		0.041					11.6		
926	7/29/2011	Kitchen Sink	Valley													147
927	7/29/2011	Bathroom Sink	Valley													169
928	7/29/2011	Kitchen Sink	Upland													138
929	7/29/2011	Spigot On Side Of House	Upland													62
930	7/29/2011	Bathroom Sink	Upland													107
931	12/21/2009	Pressure Tank	Valley	Yes			< .1		1.16					13.3		277
932	7/29/2011	Spigot On Side Of House	Upland													134
933	7/30/2011	Kitchen Sink	Valley													227
934	7/30/2011	Outside Spigot	Valley													243
935	7/30/2011	Kitchen Sink	Upland													247
936	7/30/2011	Basement Sink	Upland													429
937	7/30/2011	Outside Spigot	Valley													222

Table S.8. Groundwater Quality Data for 1701 "Pre-Drill" Water Well Samples from Susquehanna County, Pennsylvania

Water Well ID	Sample Date	Sampling Port	Topo-graphic Location	Gas Extraction Area (Within 1 km of Active Gas Well)	Groundwater Type (If Determined)	Alkalinity (mg/L)	Aluminum (mg/L)	Arsenic (mg/L)	Barium (mg/L)	Boron (mg/L)	Bromide (mg/L)	Cadmium (mg/L)	Calcium (mg/L)	Chloride (mg/L)	Chromium (mg/L)	Conductivity - Field (µs/cm)
938	7/30/2011	Presssure Tank	Valley													1166
939	7/30/2011	Kitchen Sink	Upland													142.3
940	7/30/2011	Kitchen Sink	Upland													130.7
941	8/1/2011	Outside Spigot	Valley													143
942	8/1/2011	Kitchen Sink	Upland													147
943	8/1/2011	Kitchen Sink	Valley													286
944	8/1/2011	Kitchen Sink	Valley													211
945	8/1/2011	Pressure Tank	Upland	Yes												208
946	8/1/2011	Outside Spigot	Valley													264
947	12/21/2009	Pressure Tank-Tenants House	Upland				< .1		0.074					22.1		304
948	8/1/2011	Kitchen Sink	Upland													250
949	8/1/2011	Spigot On Garage	Valley													335
950	8/1/2011	Spigot On South Side Of House	Valley													299
951	8/1/2011	Shop Sink	Upland													275
952	8/1/2011	Pressure Tank	Valley													491
953	8/2/2011	Kitchen Sink	Upland													278
954	8/2/2011	Kitchen Sink	Valley													
955	8/2/2011	Kitchen Sink	Valley													
956	8/2/2011	Kitchen Sink	Valley													275
957	8/2/2011	Kitchen Sink	Upland													241
958	8/2/2011	Pressure Tank	Valley													263
959	8/2/2011	Kitchen Sink	Upland													159
960	8/2/2011	Kitchen Sink	Upland													144

Table S.8. Groundwater Quality Data for 1701 "Pre-Drill" Water Well Samples from Susquehanna County, Pennsylvania

Water Well ID	Sample Date	Sampling Port	Topo-graphic Location	Gas Extraction Area (Within 1 km of Active Gas Well)	Groundwater Type (If Determined)	Alkalinity (mg/L)	Aluminum (mg/L)	Arsenic (mg/L)	Barium (mg/L)	Boron (mg/L)	Bromide (mg/L)	Cadmium (mg/L)	Calcium (mg/L)	Chloride (mg/L)	Chromium (mg/L)	Conductivity - Field (µs/cm)
961	8/2/2011	Spigot Across The Road From Office Building	Valley													
962	8/2/2011	Kitchen Sink	Upland													137
963	8/5/2011	Kitchen Sink	Valley													119
964	8/5/2011	Kitchen Sink	Valley													396
965	8/5/2011	Spigot On Side Of House	Valley													
966	8/5/2011	Spigot In Shed Behind House	Upland													135
967	8/5/2011	Pressure Tank	Valley													354
968	8/5/2011	Kitchen Sink	Upland	Yes												97
969	8/5/2011	Outside Spigot	Valley													507
970	12/15/2009	Pressure Tank	Upland	Yes			0.138		0.106					20.4		
971	8/5/2011	Kitchen Sink	Upland													160
972	8/5/2011	Kitchen Sink	Upland													225
973	8/5/2011	Outside Spigot	Valley													186
974	8/5/2011	Outside Spigot	Valley													564
975	8/5/2011	Spigot On Side Of House	Upland													94
976	8/5/2011	Spigot On Side Of House	Upland													134
977	8/5/2011	Kitchen Sink	Upland													212
978	8/5/2011	Spigot On Side Of House	Upland													236
979	8/5/2011	Well	Upland													67
980	8/5/2011	Pressure Tank	Upland													329
981	8/5/2011	Kitchen Sink	Upland													239

Table S.8. Groundwater Quality Data for 1701 "Pre-Drill" Water Well Samples from Susquehanna County, Pennsylvania

Water Well ID	Sample Date	Sampling Port	Topo-graphic Location	Gas Extraction Area (Within 1 km of Active Gas Well)	Groundwater Type (If Determined)	Alkalinity (mg/L)	Aluminum (mg/L)	Arsenic (mg/L)	Barium (mg/L)	Boron (mg/L)	Bromide (mg/L)	Cadmium (mg/L)	Calcium (mg/L)	Chloride (mg/L)	Chromium (mg/L)	Conductivity - Field (µs/cm)
982	8/5/2011	Kitchen Faucet	Valley													321
983	1/4/2010	Well	Valley	Yes			< .1		2.15					61.4		389
984	8/5/2011	Outside Faucet	Valley													256
985	8/5/2011	Kitchen Sink	Upland													268
986	8/8/2011	Pressure Tank	Valley													329
987	8/8/2011	Kitchen Sink	Valley													319
988	8/8/2011	Kitchen Sink	Valley													215
989	8/8/2011		Upland													
990	8/8/2011	Kitchen Sink	Valley													17.3
991	8/8/2011	Kitchen Sink	Upland													
992	8/8/2011	Kitchen Sink	Upland													224
993	8/8/2011	Outside Spigot	Upland													276
994	8/8/2011	Kitchen Sink	Valley													240
995	8/8/2011	Kitchen Sink	Valley													775
996	8/8/2011	Spigoton Side Of House	Upland													282
997	8/8/2011	Sink In Garage	Upland													364
998	8/8/2011	Kitchen Sink	Upland													274
999	8/8/2011		Valley													
1000	8/8/2011	Outside Spigot	Upland													270
1001	8/9/2011	Basement Sink	Upland													184.1
1002	8/9/2011	Kitchen Sink	Valley													349
1003	8/9/2011	Pressure Tank	Valley													273
1004	8/9/2011	Pressure Tank	Valley													296
1005	8/9/2011	Kitchen Sink	Valley													119
1006	8/9/2011	Outside Spigot	Valley													249
1007	12/14/2009	Basement At Pressure Tank	Valley				0.139		0.354					6.05		

Table S.8. Groundwater Quality Data for 1701 "Pre-Drill" Water Well Samples from Susquehanna County, Pennsylvania

Water Well ID	Sample Date	Sampling Port	Topo-graphic Location	Gas Extraction Area (Within 1 km of Active Gas Well)	Groundwater Type (If Determined)	Alkalinity (mg/L)	Aluminum (mg/L)	Arsenic (mg/L)	Barium (mg/L)	Boron (mg/L)	Bromide (mg/L)	Cadmium (mg/L)	Calcium (mg/L)	Chloride (mg/L)	Chromium (mg/L)	Conductivity - Field (µs/cm)
1008	8/9/2011	Bathroom Sink	Valley													294
1009	8/9/2011	Outside Spigot	Valley													520
1010	8/9/2011	Kitchen Sink	Upland													161.4
1011	8/9/2011	Kitchen Sink	Upland													253
1012	8/9/2011	Kitchen Sink	Upland													277
1013	8/9/2011	Kitchen Sink	Valley													218
1014	8/9/2011	Kitchen Sink	Upland													278
1015	8/9/2011	Kitchen Sink	Upland													244
1016	8/9/2011	Kitchen Sink	Upland													240
1017	7/11/2011	Spigot On Side Of House	Upland													145
1018	7/12/2011	Bathroom Sink	Valley													271
1019	7/12/2011	Sink In Rear Of Far Building	Valley													247
1020	7/21/2011	Basement Sink	Upland													170
1021	7/22/2011	Kitchen Faucet	Upland													46
1022	7/25/2011	Spigot Outside Barn	Valley													346
1023	7/25/2011	Kitchen Sink	Upland													114
1024	7/26/2011	Outside Spigot	Upland													232
1025	7/28/2011		Valley													
1026	8/1/2011	Kitchen Sink	Valley													166
1027	8/1/2011	Kitchen Sink	Valley													207
1028	8/1/2011	Outside Spigot	Upland													151
1029	8/2/2011	Pipe Overflow	Valley													175
1030	8/2/2011	Kitchen Faucet	Valley													

Table S.8. Groundwater Quality Data for 1701 "Pre-Drill" Water Well Samples from Susquehanna County, Pennsylvania

Water Well ID	Sample Date	Sampling Port	Topo-graphic Location	Gas Extraction Area (Within 1 km of Active Gas Well)	Groundwater Type (If Determined)	Alkalinity (mg/L)	Aluminum (mg/L)	Arsenic (mg/L)	Barium (mg/L)	Boron (mg/L)	Bromide (mg/L)	Cadmium (mg/L)	Calcium (mg/L)	Chloride (mg/L)	Chromium (mg/L)	Conductivity - Field (µs/cm)
1031	8/3/2011	Garage Building Next To House	Valley													190
1032	8/3/2011	Kitchen Sink	Upland													240
1033	8/3/2011	Outside Faucet	Upland													259
1034	8/3/2011	Kitchen Faucet	Valley													
1035	12/15/2009	Well House	Valley	Yes			< .1		1.04					136		
1036	8/3/2011	Pressure Tank	Upland													632
1037	8/3/2011	Pressure Tank	Upland													289
1038	8/3/2011	Kitchen Sink	Valley													378
1039	8/3/2011	Outside Faucet	Valley	Yes												285
1040	8/3/2011	Outside Faucet	Valley													334
1041	8/3/2011	Outside Faucet	Upland	Yes												374
1042	8/3/2011	Kitchen Sink	Upland													143
1043	8/3/2011	Pressure Tank	Valley													264
1044	1/3/2010	Pressure Tank	Valley				< .1		0.069					2.26		131.4
1045	8/3/2011	Spigot	Valley													328
1046	8/3/2011	Barn Sink	Valley													307
1047	8/3/2011	Kitchen Sink	Valley													274
1048	8/3/2011	Kitchen Sink	Valley													257
1049	8/3/2011	Outside Spigot	Valley													375
1050	8/3/2011	Kitchen Sink	Upland													218
1051	8/3/2011	Kitchen Sink	Upland													187
1052	8/3/2011	Kitchen Sink	Upland													162
1053	8/3/2011	Kitchen Sink	Valley													129
1054	8/3/2011	Pressure Tank In Basement	Upland													122
1055	8/3/2011	Kitchen Sink	Valley													127

Table S.8. Groundwater Quality Data for 1701 "Pre-Drill" Water Well Samples from Susquehanna County, Pennsylvania

Water Well ID	Sample Date	Sampling Port	Topo-graphic Location	Gas Extraction Area (Within 1 km of Active Gas Well)	Groundwater Type (If Determined)	Alkalinity (mg/L)	Aluminum (mg/L)	Arsenic (mg/L)	Barium (mg/L)	Boron (mg/L)	Bromide (mg/L)	Cadmium (mg/L)	Calcium (mg/L)	Chloride (mg/L)	Chromium (mg/L)	Conductivity - Field (µs/cm)
1056	8/3/2011	Kitchen Sink	Upland													105
1057	8/4/2011	Kitchen Sink	Valley													159.6
1058	8/4/2011	Outside Spigot	Upland													114
1059	8/4/2011	Outside Spigot	Upland													259
1060	12/21/2009	Laundry Sink	Upland				< .1		0.142					36.5		410
1061	8/4/2011	Spigot On Side Of House	Upland													136
1062	8/4/2011	Spigot Behind House	Valley													683
1063	8/4/2011	Kitchen Sink	Valley													219
1064	8/4/2011	Kitchen Sink	Valley													143.3
1065	8/4/2011	Kitchen Sink	Valley													220
1066	8/4/2011	Outside Spigot	Upland													249
1067	8/4/2011	Kitchen Sink	Upland													124
1068	8/4/2011	Spigot In Rear Of Building	Valley	Yes												95
1069	4/4/2009	Bathroom Sink First Floor	Valley				< .05		0.041					< 2		
1070	12/28/2009	Kitchen Sink	Upland				< .1		0.137					16.5		301
1071	8/4/2011	Pressure Tank	Valley	Yes												355
1072	8/4/2011	Kitchen Sink	Valley	Yes												333
1073	8/4/2011	Kitchen Sink	Valley	Yes												579
1074	8/4/2011	Spigot In Warehouse	Upland													147
1075	8/4/2011	Kitchen Sink	Upland													120
1076	8/4/2011	Kitchen Sink	Upland													372
1077	8/4/2011	Kitchen Sink	Valley													377

Table S.8. Groundwater Quality Data for 1701 "Pre-Drill" Water Well Samples from Susquehanna County, Pennsylvania

Water Well ID	Sample Date	Sampling Port	Topo-graphic Location	Gas Extraction Area (Within 1 km of Active Gas Well)	Groundwater Type (If Determined)	Alkalinity (mg/L)	Aluminum (mg/L)	Arsenic (mg/L)	Barium (mg/L)	Boron (mg/L)	Bromide (mg/L)	Cadmium (mg/L)	Calcium (mg/L)	Chloride (mg/L)	Chromium (mg/L)	Conductivity - Field (µs/cm)
1078	1/10/2010	Little Farm House Basement Pressure Tank	Upland				< .1		0.092					71.7		
1079	8/4/2011	Kitchen Sink	Upland													272
1080	8/4/2011	Spigot On Side Of House	Valley													165
1081	8/4/2011	Kitchen Sink	Upland													237
1082	8/4/2011	Kitchen Sink	Valley													229
1083	7/11/2011	Outside Hose Bib	Upland	Yes	Calcium-Sodium-Bicarbonate	96	<0.03	0.0021	0.12		<0.2	<0.001	23	16	<0.002	
1084	7/11/2011	Basement At Pressure Tank	Upland	Yes	Sodium-Bicarbonate	180	< .03	0.0026	0.078		< .2	< .001	8.7	48	< .002	0.354
1085	12/21/2009		Valley	Yes			< .1		0.051					4.43		
1086	7/11/2011	Bathroom Sink First Floor	Valley	Yes	Calcium-Bicarbonate	85	< .03	< .001	0.043		< .2	< .001	27	1.9	< .002	0.157
1087	7/12/2011	Outside Hose Bib	Upland	Yes	Calcium-Bicarbonate	130	< .03	0.0015	0.027		< .2	< .001	27	< 1	< .002	0.173
1088	7/12/2011	Kitchen Sink	Valley	Yes	Calcium-Bicarbonate	140	< .03	< .001	0.092		< .2	< .001	32	13	< .002	0.261
1089	7/12/2011	Pressure Tank	Valley	Yes	Calcium-Bicarbonate	130	0.044	< .001	0.11		< .2	< .001	27	4.7	< .002	0.211
1090	12/21/2009	Pressure Tank	Valley	Yes			< .1		8.06					559		1401
1091	7/13/2011	Pressure Tank In Basement	Upland		Calcium-Bicarbonate	170	< .03	< .001	0.15		< .2	< .001	39	3.6	< .002	0.748
1092	7/13/2011	Well	Valley		Calcium-Bicarbonate	140	< .03	0.001	0.012		< .2	< .001	33	1.6	< .002	
1093	7/13/2011	Outside Hose Bib	Upland	Yes	Calcium-Bicarbonate	84	0.036	0.0011	0.038		< .2	< .001	28	2.9	< .002	0.196
1094	12/14/2009	Pressure Tank In Basement	Valley	Yes			0.176		0.041					2.15		
1095	7/13/2011	Well	Valley	Yes	Calcium-Bicarbonate	120	< .03	< .001	0.064		< .2	< .001	30	< 1	< .002	0.23
1096	7/13/2011	Pressure Tank	Valley	Yes	Calcium-Bicarbonate	52	0.039	< .001	0.047		< .2	< .001	18	1.9	< .002	0.124
1097	1/3/2010	Spigot Under House	Upland	Yes			< .1		0.053					14		
1098	12/17/2009	Pressure Tank	Upland				< .1		0.615					14.6		607

Table S.8. Groundwater Quality Data for 1701 "Pre-Drill" Water Well Samples from Susquehanna County, Pennsylvania

Water Well ID	Sample Date	Sampling Port	Topo-graphic Location	Gas Extraction Area (Within 1 km of Active Gas Well)	Groundwater Type (If Determined)	Alkalinity (mg/L)	Aluminum (mg/L)	Arsenic (mg/L)	Barium (mg/L)	Boron (mg/L)	Bromide (mg/L)	Cadmium (mg/L)	Calcium (mg/L)	Chloride (mg/L)	Chromium (mg/L)	Conductivity - Field (µs/cm)
1099	1/5/2010	Kitchen Sink	Valley	Yes			< .1		7.19					361		964
1100	7/18/2011	Outside Faucet	Valley	Yes	Calcium-Bicarbonate	41	< .03	< .001	0.069		< .02	< .001	19	3.7	< .002	
1101	12/13/2009		Upland				< .1		0.114					6.93		
1102	7/19/2011	Inside Faucet	Valley	Yes	Calcium-Bicarbonate	130	< .03	0.0012	0.065		< .2	< .001	37	1	< .002	0.177
1103	7/20/2011	Kitchen Sink Cold Water Tap	Upland		Calcium-Bicarbonate	52	< .03	< .001	0.076		0.22	< .001	12	3.2	< .002	0.098
1104	7/20/2011	Pressure Tank	Upland		Calcium-Bicarbonate	120	< .03	< .001	0.078		< .2	< .001	38	7.4	< .002	0.202
1105	12/28/2009	Pressure Tank	Upland				< .1		0.193					48.7		428
1106	7/20/2011	Frost Free Hydrant 2 Feet From Well Head	Upland		Calcium-Bicarbonate	100	< .03	0.001	0.13		< .2	< .001	26	1.4	< .002	0.165
1107	7/20/2011	Inside Faucet	Upland		Calcium-Bicarbonate	100	< .03	0.0017	0.11		< .2	< .001	33	8.9	< .002	0.196
1108	12/13/2009	Pressure Tank	Upland				< .1		0.171					5.51		
1109	12/28/2009	Pressure Tank	Upland				< .1		< .025					11.8		427
1110	7/26/2011	Pressure Tank In Gun Shop	Valley		Sodium-Bicarbonate	180	< .03	< .001	0.76		0.34	< .001	15	35	< .002	486
1111	12/16/2009	Kitchen Sink	Valley	Yes			< .1		0.044					21.6		
1112	7/26/2011	Inside Faucet	Valley		Calcium-Sodium-Bicarbonate	140	< .03	0.0018	0.16		< .2	< .001	22	12	< .002	0.247
1113	7/27/2011	Outside Spigot Near Large Rock Slabs	Valley		Sodium-Bicarbonate	140	< .03	< .001	0.77		< .2	< .001	11	2.1	< .002	297
1114	12/13/2009	Pressure Tank	Upland	Yes			< .1		0.075					2.74		
1115	7/27/2011	Outside Faucet	Upland		Calcium-Bicarbonate	95	0.35	< .001	0.12		< .2	< .001	32	2.5	< .002	166
1116	7/27/2011	Kitchen Sink Cold Water Tap	Valley	Yes	Calcium-Bicarbonate	140	< .03	0.0012	0.045		< .2	< .001	44	3.9	< .002	0.196
1117	1/11/2010	Pressure Tank	Upland				< .1		1.48					59.3		
1118	7/27/2011	Kitchen Sink Faucet	Upland		Calcium-Bicarbonate	54	< .03	< .001	0.12		< .2	< .001	21	4.1	< .002	0.093
1119	7/27/2011	Outside Hose Bib	Upland		Calcium-Bicarbonate	120	< .03	0.0011	0.12		< .2	< .001	37	3.6	< .002	0.195
1120	7/28/2011	Kitchen Sink	Valley		Calcium-Bicarbonate	120	< .03	0.0016	0.22		< .2	< .001	28	5.6	< .002	0.232

Table S.8. Groundwater Quality Data for 1701 "Pre-Drill" Water Well Samples from Susquehanna County, Pennsylvania

Water Well ID	Sample Date	Sampling Port	Topo-graphic Location	Gas Extraction Area (Within 1 km of Active Gas Well)	Groundwater Type (If Determined)	Alkalinity (mg/L)	Aluminum (mg/L)	Arsenic (mg/L)	Barium (mg/L)	Boron (mg/L)	Bromide (mg/L)	Cadmium (mg/L)	Calcium (mg/L)	Chloride (mg/L)	Chromium (mg/L)	Conductivity - Field (µs/cm)
1121	7/28/2011	Inside Faucet	Valley		Calcium-Bicarbonate	120	< .03	< .001	0.14		< .2	< .001	21	2.1	< .002	184
1122	7/28/2011	Outside Faucet	Valley		Calcium-Bicarbonate	120	< .03	0.0014	0.28		< .2	< .001	24	2.7	< .002	160
1123	7/29/2011	Pressure Tank	Upland	Yes	Calcium-Bicarbonate	62	< .03	< .001	0.11		< .2	< .001	25	15	< .002	
1124	7/29/2011	Kitchen Sink	Valley		Calcium-Bicarbonate	27	0.067	< .001	0.035		< .2	< .001	6.2	1	< .002	0.093
1125	7/29/2011	Pressure Tank	Valley		Calcium-Bicarbonate	80	< .03	< .001	0.043		< .2	< .001	19	2	< .002	210
1126	8/1/2011	Outside Faucet	Upland		Calcium-Bicarbonate	110	< .03	< .001	0.16		< .2	< .001	22	2	< .002	86
1127	8/1/2011	Outside Faucet	Valley		Calcium-Bicarbonate	30	0.051	< .001	0.062		< .2	< .001	8.7	1.1	< .002	86
1128	8/1/2011	Pressure Tank At Basement	Upland	Yes	Calcium-Bicarbonate	130	0.031	< .001	0.093		< .2	< .001	40	1.6	< .002	0.218
1129	8/2/2011		Upland	Yes		110	<0.03	<0.001	0.074			<0.001	34		<0.002	
1130	12/28/2009	Kitchen Sink	Valley				< .1		0.102					5		
1131	8/3/2011	Kitchen Sink	Upland	Yes	Calcium-Bicarbonate-Chloride	69	<.03	0.0059	0.1		<.2	< .001	39	24	<.002	
1132	8/3/2011	Pressure Tank	Valley		Calcium-Sodium-Bicarbonate	110	< .03	< .001	0.69		< .2	< .001	16	4.5	< .002	0.169
1133	10/22/2009		Upland	Yes			< .1		0.15					8.65		
1134	12/28/2009	Garage Sink	Upland				< .1		0.182					86.1		
1135	8/4/2011	Bathroom Sink Cwt On South End	Upland		Calcium-Bicarbonate	83	< .03	< .001	0.11		< .2	< .001	20	2	< .002	0.16
1136	8/4/2011	Bailed Well	Upland	Yes	Calcium-Bicarbonate-Chloride	56	0.038	0.0039	0.069		< .2	< .001	23	18	< .002	0.21
1137	8/4/2011	Kitchen Sink	Upland	Yes	Calcium-Bicarbonate	120	0.16	0.003	0.15		< .2	< .001	38	1.2	< .002	0.137
1138	8/4/2011	Pressure Tank	Valley	Yes	Calcium-Bicarbonate	170	0.033	< .001	0.086		< .2	< .001	45	2.3	< .002	0.297
1139	5/19/2011		Valley		Calcium-Sodium-Bicarbonate	140	<0.03	<0.001	0.57		0.22	<0.001	15	3.6	<0.002	
1140	7/25/2011	Kitchen Sink	Valley		Calcium-Sodium-Bicarbonate	180	< .03	0.0012	0.17		< .2	< .001	25	4.4	< .002	282
1141	7/27/2011	Outside Spigot Under Back Deck	Valley		Calcium-Bicarbonate	110	0.032	0.002	0.67		< .2	< .001	19	4	< .002	250
1142	8/2/2011	Pressure Tank	Valley		Calcium-Bicarbonate	76	0.11	< .001	0.093		< .2	< .001	18	3.1	< .002	201

Table S.8. Groundwater Quality Data for 1701 "Pre-Drill" Water Well Samples from Susquehanna County, Pennsylvania

Water Well ID	Sample Date	Sampling Port	Topo-graphic Location	Gas Extraction Area (Within 1 km of Active Gas Well)	Groundwater Type (If Determined)	Alkalinity (mg/L)	Aluminum (mg/L)	Arsenic (mg/L)	Barium (mg/L)	Boron (mg/L)	Bromide (mg/L)	Cadmium (mg/L)	Calcium (mg/L)	Chloride (mg/L)	Chromium (mg/L)	Conductivity - Field (µs/cm)
1143	8/2/2011	Outside Faucet	Upland		Calcium-Bicarbonate	72	< .03	< .001	0.078		< .2	< .001	17	2.1	< .002	180
1144	1/3/2010	Kitchen Sink	Upland	Yes			< .1		0.054					21.8		
1145	12/13/2009		Upland	Yes			< .1		0.035					10.2		
1146	1/10/2010	Spring House	Upland				< .1		0.13					119		
1147	1/5/2010	Pressure Tank	Upland				< .1		0.059					5.77		
1148	5/10/2011	Kitchen Sink	Valley		Sodium-Bicarbonate	145	< .05	< .003	0.384		< 1	< .002	9.44	27.4	< .005	259
1149	2/1/2010	Pressure Tank	Upland				< .1		0.04					18.1		
1150	8/20/2009		Valley				0.165		0.369					430		
1151	12/6/2009	Washroom Sink	Valley				< .1		0.068					5.53		
1152	1/17/2010	Kitchen Sink	Valley				< .1		0.23					64.8		
1153	1/14/2010	Basement At Pressure Tank	Upland				< .1		0.046					8.19		361
1154	1/17/2010	Kitchen Sink	Upland				< .1		0.124					21.8		
1155	1/17/2010	Pressure Tank	Valley				< .1		0.084					32.7		115.7
1156	12/11/2010	Kitchen Sink	Valley		Calcium-Sodium-Bicarbonate-Chloride	20	0.027	<0.001	0.033			<0.0001	12	16.6	<0.002	
1157	8/4/2011	Spigot Back Yard	Upland													407
1158	8/4/2011	Kitchen Sink	Valley													390
1159	8/4/2011	Pressure Tank	Valley													138
1160	8/10/2011		Upland		Calcium-Sodium-Chloride	80	< .01	< .001	4.3		5.24	< .0001	40	174	< .002	
1161	8/10/2011	Kitchen Sink	Valley													262
1162	8/10/2011	Kitchen Sink	Upland													236
1163	8/10/2011	Spigot On Side Of House	Upland													
1164	8/10/2011	Outside Spigot	Valley													2.68
1165	8/10/2011	Kitchen Sink	Valley													3.53

Table S.8. Groundwater Quality Data for 1701 "Pre-Drill" Water Well Samples from Susquehanna County, Pennsylvania

Water Well ID	Sample Date	Sampling Port	Topo-graphic Location	Gas Extraction Area (Within 1 km of Active Gas Well)	Groundwater Type (If Determined)	Alkalinity (mg/L)	Aluminum (mg/L)	Arsenic (mg/L)	Barium (mg/L)	Boron (mg/L)	Bromide (mg/L)	Cadmium (mg/L)	Calcium (mg/L)	Chloride (mg/L)	Chromium (mg/L)	Conductivity - Field (µs/cm)
1166	8/10/2011	Spigot On Side Of Shed	Valley													233
1167	8/10/2011	Outside Spigot	Upland													
1168	8/10/2011	Kitchen Sink	Upland													62.9
1169	8/10/2011	Outside Spigot	Upland													306
1170	8/10/2011	Kitchen Sink	Upland													338
1171	8/10/2011	Dug Well	Upland													
1172	8/10/2011	Shop Sink	Upland													360
1173	12/10/2009	Kitchen Sink	Valley	Yes			< .1		0.133					2.51		
1174	8/11/2011	Kitchen Sink	Upland													243
1175	8/11/2011	Kitchen Sink	Upland													253
1176	8/11/2011	Outside Spigot	Valley													683
1177	8/11/2011	Kitchen Sink	Upland													299
1178	8/14/2011	Outside Spigot	Valley													193.1
1179	8/15/2011	Hand Dug Well	Upland													115.2
1180	8/16/2011	Kitchen Sink	Upland													203
1181	1/14/2010	Basement Pressure Tank	Valley				< .1		0.054					3.9		79.3
1182	8/16/2011	Dug Well	Upland													42.3
1183	8/16/2011	Kitchen Sink	Valley													206
1184	2/1/2010	Elementary Boiler Room	Upland	Yes			< .1		0.163					14.1		
1185	8/17/2011	Kitchen Sink	Valley													298
1186	8/19/2011	Kitchen Sink	Upland													176
1187	8/19/2011	Kitchen Sink	Valley													71
1188	8/29/2011	Outside Faucet	Valley		Calcium-Bicarbonate	104	< .03	< .001	0.063		< 1	< .001	39	3.18	< .002	261
1189	1/14/2010	Basement At Pressure Tank	Valley				< .1		0.187					20.8		330
1190	9/1/2011	Outside Faucet	Upland													275
1191	9/1/2011	Hydrant	Upland													85

Table S.8. Groundwater Quality Data for 1701 "Pre-Drill" Water Well Samples from Susquehanna County, Pennsylvania

Water Well ID	Sample Date	Sampling Port	Topo-graphic Location	Gas Extraction Area (Within 1 km of Active Gas Well)	Groundwater Type (If Determined)	Alkalinity (mg/L)	Aluminum (mg/L)	Arsenic (mg/L)	Barium (mg/L)	Boron (mg/L)	Bromide (mg/L)	Cadmium (mg/L)	Calcium (mg/L)	Chloride (mg/L)	Chromium (mg/L)	Conductivity - Field (µs/cm)
1192	8/2/2011	Pressure Tank	Upland	Yes	Sodium-Bicarbonate	190	0.044	0.0043	0.036		0.25	< .001	0.61	6.5	0.0021	0.417
1193	1/17/2010	Garage Sink	Upland				< .1		0.086					23.5		352
1194	8/10/2011	Outside Faucet	Valley	Yes	Calcium-Bicarbonate	120	0.095	0.003	0.47		< .2	< .001	37	< 1	0.006	254
1195	8/10/2011	Inside Faucet	Upland	Yes	Calcium-Sodium-Bicarbonate	130	< .03	< .001	1.3		< .2	< .001	27	1.1	< .002	249
1196	1/13/2010	Kitchen Sink	Upland				< .1		< .025					5.34		
1197	2/3/2010	Sink In Milkhouse Barn	Upland	Yes			< .1		0.06					24.4		
1198	8/15/2011	Outside Faucet	Upland	Yes	Calcium-Bicarbonate	140	< .03	0.0017	0.084		< .2	< .001	39	1.2	< .002	222
1199	1/12/2010	Pressure Tank	Upland				< .1		0.115					5.53		
1200	1/18/2010	Kitchen Sink	Upland				< .1		0.073					32.1		176.4
1201	8/22/2011	Barn Spigot	Upland													178
1202	8/22/2011	Kitchen Sink	Valley													170
1203	8/22/2011	Kitchen Sink	Upland													178
1204	8/22/2011	Kitchen Sink	Valley													224
1205	8/22/2011	Kitchen Sink	Upland													170
1206	8/23/2011	Outside Spigot	Valley													239
1207	1/23/2010	Basement At Pressure Tank	Upland				< .1		0.208					25.9		
1208	1/13/2010		Valley				< .1		0.58					845		
1209	6/23/2011	Kitchen Sink	Valley	Yes	Calcium-Bicarbonate	130	< .03	0.0018	0.13		< .2	< .001	28	2.8	< .002	308
1210	1/13/2010	Kitchen Sink	Upland				< .1		0.12					4.54		
1211	8/5/2011	Inside Faucet	Upland	Yes	Calcium-Bicarbonate	100	0.037	0.0014	0.19		< .2	< .001	27	7.5	< .002	0.184
1212	8/12/2011	Pressure Tank	Valley		Calcium-Bicarbonate	100	<0.03	<0.001	0.13		<0.2	<0.001	23	8.3	<0.002	
1213	12/6/2009	Kitchen Sink	Upland				< .1		0.171					31.9		
1214	8/12/2011	Inside Faucet	Upland		Calcium-Bicarbonate	78	< .03	< .001	0.1		< .2	< .001	25	1.5	< .002	1251
1215	8/21/2011	Outside Faucet	Upland		Calcium-Bicarbonate	120	2	0.012	0.34		< .2	< .001	38	4	0.0032	188
1216	8/23/2011	Inside Faucet	Upland		Calcium-Sodium-Bicarbonate	100	< .03	0.0024	0.097		< .2	< .001	20	2.5	< .002	157

Table S.8. Groundwater Quality Data for 1701 "Pre-Drill" Water Well Samples from Susquehanna County, Pennsylvania

Water Well ID	Sample Date	Sampling Port	Topo-graphic Location	Gas Extraction Area (Within 1 km of Active Gas Well)	Groundwater Type (If Determined)	Alkalinity (mg/L)	Aluminum (mg/L)	Arsenic (mg/L)	Barium (mg/L)	Boron (mg/L)	Bromide (mg/L)	Cadmium (mg/L)	Calcium (mg/L)	Chloride (mg/L)	Chromium (mg/L)	Conductivity - Field (µs/cm)
1217	8/23/2011	Inside Faucet	Upland	Yes	Calcium-Sodium-Bicarbonate	100	< .03	< .001	0.2		< .2	< .001	17	1.4	< .002	142
1218	8/24/2011	Pressure Tank	Upland	Yes	Calcium-Sodium-Bicarbonate	67	0.25	0.0073	0.12		< .2	< .001	13	3.9	< .002	119
1219	8/24/2011	Inside Faucet	Valley		Calcium-Bicarbonate	120	< .03	< .001	0.052		< .2	< .001	33	1.4	< .002	169
1220	1/18/2010	Basement Pressure Tank	Upland				< .1		0.172					89.8		
1221	8/25/2011	Inside Faucet	Upland		Calcium-Bicarbonate	110	< .03	0.0011	0.086		< .2	< .001	30	1.7	< .002	160
1222	8/26/2011	Well	Upland	Yes	Calcium-Bicarbonate	130	0.68	0.007	0.048		< .2	< .001	25	2.7	< .002	
1223	8/29/2011	Kitchen Sink	Upland		Calcium-Bicarbonate	120	< .03	0.0011	0.14		< .2	< .001	39	1.3	< .002	259
1224	1/11/2010	Kitchen Sink	Upland				< .1		0.318					34.8		278
1225	1/18/2010	Pressure Tank In Basement	Valley	Yes			< .1		0.103					35		244
1226	1/24/2010	Pressure Tank	Upland	Yes			< .1		0.162					45.7		
1227	8/30/2011	Pressure Tank	Upland		Calcium-Bicarbonate	130	0.073	< .001	0.19		< .2	< .001	31	1.1	< .002	262
1228	8/30/2011	Outside Hose Bib	Valley		Calcium-Bicarbonate	140	< .03	< .001	0.15		< .2	< .001	34	1.8	< .002	222
1229	8/10/2011	Pressure Tank Before Treatment	Valley		Sodium-Bicarbonate-Chloride	130	< .03	< .001	0.87		0.67	< .001	12	65	< .002	373
1230	1/10/2010	Little House By Pond Spring	Valley				< .1		0.042					19.8		
1231	1/10/2010	Pressure Tank	Valley				< .1		2.8					5.73		
1232	1/16/2010	Basement Pressure Tank	Upland				< .1		0.444					64.4		
1233	7/19/2011	Kitchen Sink	Upland													347
1234	8/24/2011	Kitchen Sink	Valley													205
1235	8/24/2011	Inside Faucet	Valley													102
1236	1/16/2010	Basement At Pressure Tank	Upland				< .1		0.099					27.5		
1237	1/16/2010	Basement Pressure Tank	Upland				< .1		0.133					18.2		261

Table S.8. Groundwater Quality Data for 1701 "Pre-Drill" Water Well Samples from Susquehanna County, Pennsylvania

Water Well ID	Sample Date	Sampling Port	Topo-graphic Location	Gas Extraction Area (Within 1 km of Active Gas Well)	Groundwater Type (If Determined)	Alkalinity (mg/L)	Aluminum (mg/L)	Arsenic (mg/L)	Barium (mg/L)	Boron (mg/L)	Bromide (mg/L)	Cadmium (mg/L)	Calcium (mg/L)	Chloride (mg/L)	Chromium (mg/L)	Conductivity - Field (µs/cm)
1238	8/26/2011	Kitchen Sink	Valley													159
1239	8/27/2011	Kitchen Sink	Upland													206
1240	8/27/2011	Kitchen Sink	Valley													253
1241	1/21/2010	Kitchen Sink	Upland				< .1		0.098					71.2		285
1242	8/27/2011	Kitchen Faucet	Valley													
1243	1/23/2010	Pressre Tank In Barn	Upland	Yes			< .1		0.11					66.9		
1244	9/1/2011	Kitchen Sink	Valley													145
1245	1/23/2010	Basement At Pressure Tank	Upland	Yes			0.1		0.136					115		
1246	9/1/2011	Well In Field	Upland													62
1247	8/10/2011	Kitchen Sink	Upland													255
1248	8/10/2011	Kitchen Sink	Upland													246
1249	8/11/2011	Pressure Tank	Valley													1011
1250	1/14/2010	Basement At Pressure Tank	Upland				< .1		0.09					3.58		342
1251	2/3/2010	Holding Tank	Upland				< .1		0.122					6.42		84.9
1252	8/18/2011	Kitchen Sink	Valley	Yes	Calcium-Bicarbonate	150	< .03	0.0012	0.11		< .2	< .001	37	2.2	< .002	270
1253	1/31/2010	Basement At Pressure Tank	Upland				< .1		0.051					7.99		
1254	1/24/2010	Kitchen Sink	Upland				< .1		0.083					10.8		298
1255	1/21/2010	Pressure Tank	Valley	Yes			< .1		0.832					56		
1256	2/12/2009		Upland	Yes			0.077		0.08					33.2		
1257	4/8/2009		Upland				<0.05		0.099					52.4		
1258	1/16/2010	Basement Pressure Tank	Upland	Yes			< .1		0.064					20.6		
1259	1/24/2010	Pressure Tank - Drilled Well	Valley	Yes			< .1		0.068					9.18		
1260	1/31/2010	Crawl Space At Pressure Tank	Valley				< .1		0.072					7.26		379
1261	1/31/2010	Pressure Tank	Valley				< .1		0.051					3.52		

Table S.8. Groundwater Quality Data for 1701 "Pre-Drill" Water Well Samples from Susquehanna County, Pennsylvania

Water Well ID	Sample Date	Sampling Port	Topo-graphic Location	Gas Extraction Area (Within 1 km of Active Gas Well)	Groundwater Type (If Determined)	Alkalinity (mg/L)	Aluminum (mg/L)	Arsenic (mg/L)	Barium (mg/L)	Boron (mg/L)	Bromide (mg/L)	Cadmium (mg/L)	Calcium (mg/L)	Chloride (mg/L)	Chromium (mg/L)	Conductivity - Field (µs/cm)
1262	1/24/2010	Outside Spigot	Upland				< .1		0.074					5.04		
1263	1/24/2010	Kitchen Sink	Upland				< .1		0.085					5.81		
1264	1/23/2010	Pressure Tank In Basement	Valley	Yes			< .1		< .025					30.3		75.5
1265	1/18/2010	Basement Pressure Tank	Upland				< .1		0.105					84.2		260
1266	1/10/2010	Basement Pressure Tank	Upland				< .1		< .025					4.12		
1267	1/11/2010	Pressure Tank	Upland				< .1		0.118					2.39		138.6
1268	12/9/2009	Basement At Pressure Tank	Upland	Yes			< .1		0.147					8.37		
1269	1/18/2010	Pressure Tank	Upland				< .1		0.093					32.9		359
1270	1/31/2010		Upland	Yes			< .1		0.098					2.27		
1271	8/7/2009	Kitchen Sink	Valley				< .01		0.055					4.32		
1272	1/12/2010	Kitchen Sink At Ashley Sarnosky'S	Upland				< .1		0.088					4.55		78.2
1273	2/21/2010	Kitchen Sink	Upland				< .1		0.179					3.56		205
1274	8/6/2009		Upland	Yes			< .1		0.084					14.7		
1275	2/15/2010	Pressure Tank	Upland				< .1		1.42					14.7		328
1276	2/13/2010	Basement At Pressure Tank	Valley				< .1		0.073					2.47		
1277	2/27/2010	Basement At Spigot	Valley				< .1		0.12					8.69		
1278	2/18/2010	Pressure Tank	Upland	Yes			< .1		0.118					19.2		
1279	2/16/2010	Kitchen Sink	Upland				< .1		0.231					2.7		215
1280	2/18/2010	Kitchen Sink	Upland	Yes			< .1		0.124					19.8		
1281	2/18/2010	Kitchen Sink	Upland				< .1		0.171					13.4		252
1282	2/18/2010	Kitchen Sink	Upland				< .1		0.124					12.3		

Table S.8. Groundwater Quality Data for 1701 "Pre-Drill" Water Well Samples from Susquehanna County, Pennsylvania

Water Well ID	Sample Date	Sampling Port	Topo-graphic Location	Gas Extraction Area (Within 1 km of Active Gas Well)	Groundwater Type (If Determined)	Alkalinity (mg/L)	Aluminum (mg/L)	Arsenic (mg/L)	Barium (mg/L)	Boron (mg/L)	Bromide (mg/L)	Cadmium (mg/L)	Calcium (mg/L)	Chloride (mg/L)	Chromium (mg/L)	Conductivity - Field (µs/cm)
1283	2/22/2010	Rental House Basement At Pressure Tank	Upland				< .1		0.088					24.5		
1284	2/16/2010	Kitchen Sink	Upland				< .1		0.131					30.3		304
1285	2/21/2010	Kitchen Sink	Upland				< .1		1.29					40.7		
1286	2/18/2010	Pressure Tank	Valley				8.91		0.495					11		265
1287	2/6/2010	Basement At Pressure Tank	Valley				< .1		0.055					38.1		271
1288	2/13/2010	Pressure Tank	Upland				< .1		0.263					48.1		
1289	2/1/2010	Basement Sink	Valley	Yes			< .1		0.07					12.6		
1290	2/21/2010	Pressure Tank	Upland				< .1		0.099					25.2		202
1291	2/21/2010	Barn Well	Upland				< .1		0.13					183		625
1292	2/16/2010	Kitchen Sink	Upland				< .1		0.176					5.55		200
1293	2/22/2010	Hand Dug Well	Upland				< .1		0.034					2.11		180.5
1294	2/16/2010	Bathroom Sink	Upland	Yes			< .1		0.117					11.9		
1295	2/16/2010	Pressure Tank	Upland				< .1		0.15					44.1		
1296	2/7/2010	Main House Pressure Tank	Valley				< .1		0.192					31		
1297	2/7/2010	Rental Property Pressure Tank	Upland				< .1		0.252					196		
1298	2/13/2010	Kitchen Sink	Upland				< .1		0.9					15.2		
1299	2/13/2010	Well House	Upland				< .1		0.079					53.8		
1300	8/6/2009		Valley				0.199		0.046					2.16		
1301	2/7/2010	Barn - Pump	Upland				< .1		0.252					16.1		251
1302	2/7/2010	Pressure Tank	Upland				< .1		0.096					6.04		246
1303	2/7/2010	Pressure Tank	Upland				< .1		0.14					13.5		
1304	2/6/2010	Pressure Tank	Valley				< .1		0.162					143		
1305	2/6/2010	Basement At Pressure Tank	Valley				< .1		0.134					18		309

Table S.8. Groundwater Quality Data for 1701 "Pre-Drill" Water Well Samples from Susquehanna County, Pennsylvania

Water Well ID	Sample Date	Sampling Port	Topo-graphic Location	Gas Extraction Area (Within 1 km of Active Gas Well)	Groundwater Type (If Determined)	Alkalinity (mg/L)	Aluminum (mg/L)	Arsenic (mg/L)	Barium (mg/L)	Boron (mg/L)	Bromide (mg/L)	Cadmium (mg/L)	Calcium (mg/L)	Chloride (mg/L)	Chromium (mg/L)	Conductivity - Field (µs/cm)
1306	2/6/2010	Pressure Tank	Valley				< .1		0.1					5.1		204
1307	2/15/2010	Outside Hydrant	Upland	Yes			< .1		0.162					17.2		244
1308	2/27/2010	Hand Dug Well 1 Back Of House	Valley				< .1		0.037					29.9		
1309	2/27/2010	Hand Dug Well 2 Front Of House	Valley				< .1		0.029					73.5		
1310	2/7/2010	Outside Faucet	Valley				< .1		2.57					69.9		38.3
1311	2/6/2010	Basement At Pressure Tank	Valley				< .1		0.184					10.2		
1312	2/21/2010	Kitchen Sink	Valley				< .1		0.314					2.91		
1313	8/6/2009		Valley				< .1		0.089					2.89		
1314	2/22/2010	Milkhouse Sink	Upland				< .1		0.098					3.72		294
1315	2/28/2010	Pressure Tank	Valley	Yes			< .1		0.043					3.77		
1316	2/13/2010	Basement Men'S Room Sink	Upland				< .1		0.511					228		
1317	2/28/2010	Outside Spigot	Valley				< .1		0.564					29.7		276
1318	2/28/2010	Kitchen Sink	Upland				< .1		0.052					5.35		
1319	2/27/2010	Basement Pressure Tank	Valley				< .1		< .025					3.12		
1320	3/16/2010	Kitchen Sink	Valley	Yes			< .1		0.033					< 2		45.3
1321	3/11/2010	Kitchen Sink	Upland	Yes			< .1		0.079					2.73		200
1322	3/18/2010	Kitchen Sink	Upland				< .1	< .003	0.36	0.133				187		718
1323	8/11/2011	Kitchen Sink	Upland	Yes												123.1
1324	8/11/2011	Kitchen Sink	Valley													278
1325	8/11/2011	Kitchen Sink	Upland													95.2
1326	8/11/2011	Kitchen Sink	Upland													168.8
1327	8/11/2011	Kitchen Sink	Upland													203
1328	8/11/2011	Kitchen Sink	Upland													198.4

Table S.8. Groundwater Quality Data for 1701 "Pre-Drill" Water Well Samples from Susquehanna County, Pennsylvania

Water Well ID	Sample Date	Sampling Port	Topo-graphic Location	Gas Extraction Area (Within 1 km of Active Gas Well)	Groundwater Type (If Determined)	Alkalinity (mg/L)	Aluminum (mg/L)	Arsenic (mg/L)	Barium (mg/L)	Boron (mg/L)	Bromide (mg/L)	Cadmium (mg/L)	Calcium (mg/L)	Chloride (mg/L)	Chromium (mg/L)	Conductivity - Field (µs/cm)
1329	8/11/2011	Kitchen Sink	Upland													138.6
1330	8/6/2009		Valley				< .1		0.126					65.2		
1331	8/11/2011	Kitchen Sink	Upland													156.7
1332	8/11/2011	Kitchen Sink	Valley													264
1333	8/11/2011	Kitchen Sink	Upland													461
1334	8/11/2011	Kitchen Sink	Valley													
1335	8/12/2011	Kitchen Sink	Valley													330
1336	8/12/2011	Kitchen Sink	Valley													166.3
1337	8/12/2011	Kitchen Sink	Valley													174.8
1338	8/12/2011	Kitchen Sink	Valley													212
1339	8/12/2011	Kitchen Sink	Valley													187.1
1340	5/13/2010		Upland	Yes										5.47		
1341	8/12/2011	Pressure Tank	Upland													123.9
1342	8/12/2011	Kitchen Sink	Valley													248
1343	8/12/2011	Bathroom Sink	Valley													183.1
1344	8/14/2011	Outside Spigot	Valley													224
1345	8/14/2011	Kitchen Sink	Upland													85.4
1346	5/13/2010		Upland				< .1	< .003	0.423	< .1				2.76		
1347	8/14/2011	Kitchen Sink	Valley													231
1348	8/14/2011	Kitchen Sink	Upland													154.1
1349	8/14/2011	Kitchen Sink	Upland													239
1350	8/14/2011	Pressure Tank	Upland													260
1351	8/14/2011	Kitchen Sink	Upland													302
1352	8/15/2011	Kitchen Sink	Upland													205
1353	8/15/2011	Barn Sink	Upland													232
1354	8/15/2011	Kitchen Sink	Valley													299
1355	8/15/2011	Kitchen Sink	Valley													267
1356	8/15/2011	Sink In Barn	Valley													306

Table S.8. Groundwater Quality Data for 1701 "Pre-Drill" Water Well Samples from Susquehanna County, Pennsylvania

Water Well ID	Sample Date	Sampling Port	Topo-graphic Location	Gas Extraction Area (Within 1 km of Active Gas Well)	Groundwater Type (If Determined)	Alkalinity (mg/L)	Aluminum (mg/L)	Arsenic (mg/L)	Barium (mg/L)	Boron (mg/L)	Bromide (mg/L)	Cadmium (mg/L)	Calcium (mg/L)	Chloride (mg/L)	Chromium (mg/L)	Conductivity - Field (µs/cm)
1357	8/16/2011	Kitchen Sink	Upland													185.1
1358	8/16/2011	Outside Spigot	Upland													212
1359	8/16/2011	Kitchen Sink	Upland													200
1360	8/16/2011	Kitchen Sink	Upland													205
1361	8/16/2011	Kitchen Sink	Valley													182.5
1362	8/16/2011	Spigot On Side Of House	Upland													231
1363	8/17/2011	Kitchen Sink	Upland													227
1364	8/17/2011	Outside Spigot	Valley													168.2
1365	8/17/2011	Kitchen Sink	Upland													212
1366	8/17/2011	Kitchen Sink	Valley													248
1367	8/17/2011	Kitchen Sink	Valley													302
1368	8/17/2011	Kitchen Sink	Valley													258
1369	8/18/2011	Kitchen Sink	Upland													142
1370	2/8/2010	Basement At Pressure Tank	Upland	Yes			< .1		0.134					36.6		
1371	8/18/2011	Kitchen Sink	Valley													170
1372	8/18/2011	Kitchen Sink	Valley													162
1373	8/18/2011	Kitchen Sink	Valley													246
1374	8/18/2011	Barn Sink	Upland													147
1375	8/18/2011	Barn Sink	Valley													150
1376	3/8/2010	Kitchen Sink	Valley	Yes			< .1		0.148					6.34		294
1377	8/18/2011	Kitchen Sink	Upland													155
1378	8/19/2011	Kitchen Sink	Upland													157
1379	8/19/2011	Kitchen Sink	Upland													176
1380	8/19/2011	Kitchen Sink	Upland													
1381	8/17/2009		Valley				< .1		0.269					13.3		
1382	8/19/2011		Valley													125

Table S.8. Groundwater Quality Data for 1701 "Pre-Drill" Water Well Samples from Susquehanna County, Pennsylvania

Water Well ID	Sample Date	Sampling Port	Topo-graphic Location	Gas Extraction Area (Within 1 km of Active Gas Well)	Groundwater Type (If Determined)	Alkalinity (mg/L)	Aluminum (mg/L)	Arsenic (mg/L)	Barium (mg/L)	Boron (mg/L)	Bromide (mg/L)	Cadmium (mg/L)	Calcium (mg/L)	Chloride (mg/L)	Chromium (mg/L)	Conductivity - Field (µs/cm)
1383	8/19/2011	Kitchen Sink	Valley													104
1384	8/22/2011	Kitchen Sink	Upland													158
1385	8/22/2011	Kitchen Sink	Upland													103
1386	8/23/2011	Outside Spigot	Upland													227
1387	8/23/2011	Barn Outside Spigot	Upland													164.9
1388	8/23/2011	Outside Spigot	Upland													213
1389	3/4/2010	Post Office - Bathroom Sink	Upland	Yes			< .1		0.217					67.4		468
1390	8/23/2011	Outside Spigot	Valley													163.4
1391	8/23/2011	Outside Spigot	Upland													310
1392	8/24/2011	Kitchen Sink	Upland													107.1
1393	8/24/2011	Kitchen Sink	Upland													225
1394	8/24/2011	Kitchen Sink	Valley													155
1395	8/24/2011	Kitchen Sink	Upland													107
1396	8/24/2011	Kitchen Sink	Valley													129
1397	8/24/2011	Inside Faucet	Upland													85
1398	8/24/2011	Kitchen Sink	Upland													212
1399	8/24/2011	Kitchen Sink	Valley													175.4
1400	3/8/2010	Basement At Pressure Tank	Upland				< .1		0.033					< 2		
1401	8/24/2011	Kitchen Sink	Upland													
1402	8/24/2011	Kitchen Sink	Upland													277
1403	8/24/2011	Kitchen Sink	Upland													185.5
1404	8/24/2011	Bathroom Sink	Valley													145
1405	8/24/2011	Kitchen Sink	Valley													137
1406	8/25/2011	Kitchen Sink	Valley													121

Table S.8. Groundwater Quality Data for 1701 "Pre-Drill" Water Well Samples from Susquehanna County, Pennsylvania

Water Well ID	Sample Date	Sampling Port	Topo-graphic Location	Gas Extraction Area (Within 1 km of Active Gas Well)	Groundwater Type (If Determined)	Alkalinity (mg/L)	Aluminum (mg/L)	Arsenic (mg/L)	Barium (mg/L)	Boron (mg/L)	Bromide (mg/L)	Cadmium (mg/L)	Calcium (mg/L)	Chloride (mg/L)	Chromium (mg/L)	Conductivity - Field (µs/cm)
1407	8/25/2011	Kitchen Sink	Valley													101
1408	8/25/2011	Kitchen Sink	Valley													155
1409	8/25/2011	Downstairs Sink	Valley													177
1410	8/25/2011	Kitchen Sink	Upland													139
1411	8/25/2011	Kitchen Sink	Valley													253
1412	8/25/2011	Kitchen Sink	Valley													231
1413	8/26/2011	Kitchen Sink	Upland													125
1414	8/26/2011	Outside Spigot	Valley													99
1415	8/26/2011	Kitchen Sink	Upland													133
1416	8/26/2011	Kitchen Sink	Valley													139
1417	8/26/2011	Kitchen Sink	Upland													127
1418	8/26/2011	Kitchen Sink	Upland													138
1419	8/26/2011	Barn Sink	Valley													333
1420	8/27/2011	Kitchen Sink	Upland													219
1421	8/27/2011	Kitchen Sink	Upland													128.4
1422	10/30/2009	Basement At Pressure Tank	Upland	Yes			< .1		0.072					7.64		160.7
1423	8/31/2011	Kitchen Sink	Upland													323
1424	8/31/2011	Kitchen Sink	Valley													
1425	8/31/2011	Spigot On Well	Valley													236
1426	8/31/2011	Kitchen Sink	Upland													115.3
1427	9/1/2011	Kitchen Sink	Valley													126
1428	9/1/2011	Spigot Side Of House	Valley													121
1429	9/2/2011	Kitchen Sink	Upland													148
1430	9/2/2011	Spigot On Well	Valley													157
1431	3/7/2010	Kitchen Sink	Valley				< .1		< .025					< 2		434

Table S.8. Groundwater Quality Data for 1701 "Pre-Drill" Water Well Samples from Susquehanna County, Pennsylvania

Water Well ID	Sample Date	Sampling Port	Topo-graphic Location	Gas Extraction Area (Within 1 km of Active Gas Well)	Groundwater Type (If Determined)	Alkalinity (mg/L)	Aluminum (mg/L)	Arsenic (mg/L)	Barium (mg/L)	Boron (mg/L)	Bromide (mg/L)	Cadmium (mg/L)	Calcium (mg/L)	Chloride (mg/L)	Chromium (mg/L)	Conductivity - Field (µs/cm)
1432	9/2/2011	Kitchen Sink	Upland													116
1433	9/2/2011	Kitchen Sink	Valley													107
1434	9/2/2011	Bathroom Sink	Upland													68
1435	9/2/2011	Kitchen Sink	Upland													93
1436	9/2/2011	Well Spigot	Upland													64
1437	3/8/2010	Basement At Pressure Tank	Upland				< .1		0.025					< 2		
1438	9/2/2011	Kitchen Sink	Valley													153
1439	9/7/2011	Kitchen Sink	Upland													135
1440	9/7/2011	Kitchen Sink	Upland													232
1441	9/7/2011	Kitchen Sink	Valley													484
1442	9/7/2011	Kitchen Sink	Upland													173.3
1443	9/8/2011	Kitchen Faucet	Valley													
1444	9/8/2011	Kitchen Sink	Valley													124
1445	9/8/2011	Outside Spigot	Upland													120
1446	9/8/2011	Kitchen Sink	Valley													142
1447	9/8/2011	Kitchen Sink	Upland													114
1448	9/8/2011	Kitchen Sink	Upland													58
1449	9/8/2011	Kitchen Sink	Upland													189
1450	3/8/2010	Basement Pressure Tank	Upland				< .1		0.201					10.3		286
1451	8/20/2009		Upland	Yes			< .1		0.147					6.77		
1452	3/7/2010	Pressure Tank	Upland				< .1		0.083					< 2		
1453	3/7/2010	Kitchen Sink	Upland				< .1		0.064					4.37		
1454	3/11/2010	Pressure Tank	Valley				< .1		0.168					11.8		268
1455	5/1/2010	Kitchen Sink	Valley				< .1	< .003	0.182	< .1				2.61		
1456	5/1/2010	Kitchen Sink	Valley				< .1	< .003	0.034	< .1				12		93.2

Table S.8. Groundwater Quality Data for 1701 "Pre-Drill" Water Well Samples from Susquehanna County, Pennsylvania

Water Well ID	Sample Date	Sampling Port	Topo-graphic Location	Gas Extraction Area (Within 1 km of Active Gas Well)	Groundwater Type (If Determined)	Alkalinity (mg/L)	Aluminum (mg/L)	Arsenic (mg/L)	Barium (mg/L)	Boron (mg/L)	Bromide (mg/L)	Cadmium (mg/L)	Calcium (mg/L)	Chloride (mg/L)	Chromium (mg/L)	Conductivity - Field (µs/cm)
1457	5/3/2010	Outside Spigot On Garage	Upland	Yes			< .1	< .003	< .025	< .1				5.2		76.5
1458	5/6/2010	Kitchen Sink	Upland				< .1	< .003	0.045	< .1				2.86		195.3
1459	5/6/2010	Kitchen Sink	Valley	Yes			< .1	< .003	1.6	0.137				140		682
1460	5/7/2010	Kitchen Sink	Valley	Yes			< .1	0.004	0.268	0.13				11.8		254
1461	8/24/2011	Kitchen Sink	Valley		Calcium-Bicarbonate	100	< .03	< .001	0.071		< .2	< .001	30	2.3	< .002	180
1462	8/24/2011	Inside Faucet	Valley		Calcium-Bicarbonate	130	< .03	< .001	0.15		< .2	< .001	30	2.9	< .002	191
1463	8/24/2011	Pressure Tank Hose Bib	Upland		Calcium-Sodium-Bicarbonate	78	< .03	< .001	0.073		< .2	< .001	19	3.7	< .002	130
1464	5/7/2010	Outside Spigot	Upland				< .1	< .003	0.137	< .1				5.45		
1465	8/30/2011	Inside Faucet	Valley		Calcium-Bicarbonate	180	0.17	0.0016	0.15		< .2	< .001	43	< 1	< .002	233
1466	5/13/2010	Kitchen Sink	Valley				< .1	< .003	0.066	< .1				2.43		192.4
1467	8/30/2011	Pressure Tank	Valley		Calcium-Bicarbonate	140	< .03	< .001	0.14		< .2	< .001	33	1	< .002	421
1468	5/13/2010	Kitchen Sink	Upland				< .1	< .003	0.18	< .1				3.12		234
1469	5/13/2010	Outside Spigot	Upland				< .1	< .003	0.109	< .1				3.93		269
1470	8/30/2011	Inside Faucet	Upland		Calcium-Bicarbonate	130	< .03	< .001	0.094		< .2	< .001	35	1.8	0.0047	173
1471	7/7/2009	Kitchen Sink	Upland				< .05		0.076					2.6		
1472	5/20/2010	Kitchen Sink	Upland				< .1	< .003	0.143	< .1						213
1473	5/20/2010	Basement At Pressure Tank	Upland				< .1	< .003	0.093	< .1				2.93		136.3
1474	5/20/2010	Pressure Tank- Quarry Top Well	Upland											5.28		
1475	5/20/2010	Pressure Tank At Trailer Park	Upland				< .1	< .003	0.218	< .1				6.27		218
1476	5/20/2010	Quarry Sulfur Well	Upland				< .1	< .003	0.204	< .1				6.51		223
1477	5/20/2010	Spigot In Garage	Upland				< .1	< .003	0.131	< .1				6.17		120.8
1478	3/16/2010	Outside Spigot	Upland				< .1		0.096					< 2		

Table S.8. Groundwater Quality Data for 1701 "Pre-Drill" Water Well Samples from Susquehanna County, Pennsylvania

Water Well ID	Sample Date	Sampling Port	Topo-graphic Location	Gas Extraction Area (Within 1 km of Active Gas Well)	Groundwater Type (If Determined)	Alkalinity (mg/L)	Aluminum (mg/L)	Arsenic (mg/L)	Barium (mg/L)	Boron (mg/L)	Bromide (mg/L)	Cadmium (mg/L)	Calcium (mg/L)	Chloride (mg/L)	Chromium (mg/L)	Conductivity - Field (µs/cm)
1479	4/19/2009	Kitchen Sink	Upland				< .05		0.097					5.93		
1480	3/18/2010	Kitchen Sink	Upland				< .1	< .003	0.044	< .05				3.25		
1481	3/18/2010	Pressure Tank	Upland	Yes			< .1	< .003	0.085	< .05				5.63		81.8
1482	3/22/2010	Kitchen Sink	Valley	Yes			< .1	< .003	0.048	< .05				4.73		123.1
1483	1/13/2011		Valley	Yes	Calcium-Bicarbonate	120	< .01	0.003	0.41			< .0001	29	21.8	< .002	
1484	3/25/2010	Basement At Pressure Tank	Upland				< .1	< .003	0.044	< .05				5.24		254
1485	3/25/2010	Hand Dug Well	Valley				< .1	< .003	0.031	< .05				2.56		55
1486	6/14/2011	Directly From Pressure Tank Cwt In Sw Corner Of Ba	Upland	Yes	Calcium-Bicarbonate	58	0.31	< .001	0.034		< .2	< .001	14	1	< .002	0.108
1487	6/14/2011	Kitchen Sink	Upland		Calcium-Bicarbonate	170	< .03	0.001	0.1		< .2	< .001	40	< 1	< .002	0.246
1488	6/14/2011	Hose Bib	Upland		Calcium-Bicarbonate-Chloride	28	0.034	< .001	0.078		< .2	< .001	18	7.4	< .002	0.174
1489	3/25/2010	Spring At Overflow Pipe	Valley				< .1	< .003	0.048	< .05				5.52		82.4
1490	6/14/2011	Outside Hose Bib On Sw End Of Home	Valley		Calcium-Bicarbonate	110	< .03	0.0017	0.13		< .2	< .001	31	11	< .002	0.209
1491	3/28/2010	Basement At Pressure Tank	Upland				< .1	0.016	0.549	0.075				5.2		371
1492	1/13/2011		Upland	Yes	Calcium-Bicarbonate	85	<0.01	0.0015	0.14			<0.0001	28	5.68	<0.0020	157
1493	6/16/2011	Wash Tub In Garage	Upland		Calcium-Bicarbonate	150	< .03	< .001	0.087		< .2	< .001	34	< 1	< .002	0.061
1494	6/16/2011	Kitchen Sink	Valley		Calcium-Sodium-Bicarbonate-Chloride	57	0.054	< .001	0.071		< .2	< .001	24	44	< .002	456
1495	6/27/2011	Bathroom Sink Body Shop	Valley	Yes	Calcium-Bicarbonate	110	< .03	< .001	0.16		< .2	< .001	22	1.9	< .002	265
1496	6/28/2011	Garage Sink	Valley		Calcium-Bicarbonate	110	< .03	< .001	0.14		< .2	< .001	30	1.6	< .002	268
1497	6/28/2011	Kitchen Sink	Upland		Calcium-Bicarbonate	120	< .03	< .001	0.077		< .2	< .001	35	4.3	< .002	297

Table S.8. Groundwater Quality Data for 1701 "Pre-Drill" Water Well Samples from Susquehanna County, Pennsylvania

Water Well ID	Sample Date	Sampling Port	Topo-graphic Location	Gas Extraction Area (Within 1 km of Active Gas Well)	Groundwater Type (If Determined)	Alkalinity (mg/L)	Aluminum (mg/L)	Arsenic (mg/L)	Barium (mg/L)	Boron (mg/L)	Bromide (mg/L)	Cadmium (mg/L)	Calcium (mg/L)	Chloride (mg/L)	Chromium (mg/L)	Conductivity - Field (µs/cm)
1498	3/18/2010	Kitchen Sink	Upland				< .1	< .003	0.205	< .05				8.04		259
1499	5/21/2010	Kitchen Sink	Upland	Yes			< .1	< .003	0.068	< .1						317
1500	5/21/2010	Hand Dug #1	Upland	Yes			< .1	< .003	< .025	< .1						61.7
1501	7/6/2011	Kitchen Sink	Upland		Calcium-Bicarbonate	120	0.031	< .001	0.15		< .2	< .001	41	4.3	< .002	302
1502	7/6/2011	Kitchen Sink	Upland		Calcium-Bicarbonate	79	< .03	0.0013	0.21		< .2	< .001	23	5.5	< .002	243
1503	5/21/2010	Hand Dug Well #2	Upland	Yes			< .1	< .003	< .025	< .1						76.1
1504	7/6/2011	Kitchen Sink	Valley		Calcium-Bicarbonate	62	< .03	< .001	0.12		< .2	< .001	21	5.9	< .002	194
1505	7/6/2011	Kitchen Sink	Upland		Calcium-Bicarbonate	120	< .03	< .001	0.1		< .2	< .001	30	5	< .002	0.09
1506	7/7/2011	Pressure Tank Tap	Valley		Calcium-Bicarbonate	68	< .03	< .001	0.058		< .2	< .001	22	3	< .002	191
1507	5/21/2010		Valley				< .1	< .003	0.113	< .1						
1508	7/14/2011	Outdoor Spigot	Upland		Calcium-Bicarbonate	130	< .03	< .001	0.13		< .2	< .001	42	3	< .002	251
1509	7/16/2011	Pressure Tank	Valley		Calcium-Sodium-Bicarbonate	110	< .03	0.0042	0.23		< .2	< .001	19	1.4	< .002	0.143
1510	5/22/2010	Spare Well	Upland				< .1	< .003	< .039	< .1						91.1
1511	5/22/2010	Kitchen Sink	Upland				< .1	< .003	0.104	< .1						336
1512	7/18/2011	Outside Hose Bib	Upland		Calcium-Bicarbonate	120	0.31	0.002	0.14		< .2	< .001	36	7	< .002	0.203
1513	7/18/2011	Hydrant Northwest Of Well	Upland		Calcium-Bicarbonate	160	< .03	0.0017	0.27		< .2	< .001	48	40	< .002	0.453
1514	7/18/2011		Valley	Yes	Calcium-Bicarbonate	98	< .03	< .001	0.021		< .2	< .001	31	1.3	< .002	
1515	5/16/2011	Pressure Tank	Upland		Calcium-Bicarbonate	110	<0.03	0.001	0.05		<0.2	<0.001	27	3.9	<0.002	
1516	5/16/2011	Slop Sink In Barn	Upland		Calcium-Bicarbonate	85	< .03	< .001	0.093		< .2	< .001	20	3	< .002	0.202
1517	5/16/2011	Pressure Tank	Upland		Calcium-Bicarbonate	170	< .03	< .001	0.087		< .2	< .001	30	1.5	< .002	297
1518	5/16/2011	Outdoor Spigot	Upland		Calcium-Bicarbonate	41	0.064	< .001	0.035		< .2	< .001	13	1.1	< .002	98
1519	7/25/2011	Kitchen Sink Cold Water Tap	Upland		Calcium-Bicarbonate	100	< .03	0.0018	0.097		< .2	< .001	29	12	< .002	0.954
1520	5/17/2011	Outside Spigot	Upland		Calcium-Bicarbonate	140	< .03	< .001	0.1		< .2	< .001	27	2	< .002	211
1521	5/17/2011	Kitchen Sink	Valley		Calcium-Sodium-Bicarbonate	150	< .03	< .001	0.39		< .2	< .001	17	2.8	< .002	247

Table S.8. Groundwater Quality Data for 1701 "Pre-Drill" Water Well Samples from Susquehanna County, Pennsylvania

Water Well ID	Sample Date	Sampling Port	Topo-graphic Location	Gas Extraction Area (Within 1 km of Active Gas Well)	Groundwater Type (If Determined)	Alkalinity (mg/L)	Aluminum (mg/L)	Arsenic (mg/L)	Barium (mg/L)	Boron (mg/L)	Bromide (mg/L)	Cadmium (mg/L)	Calcium (mg/L)	Chloride (mg/L)	Chromium (mg/L)	Conductivity - Field (µs/cm)
1522	5/17/2011	Kitchen Sink	Valley		Calcium-Sodium-Bicarbonate	130	< .03	< .001	0.36		< .2	< .001	20	1.8	< .002	230
1523	5/17/2011	Outdoor Spigot	Upland		Calcium-Bicarbonate	100	< .03	< .001	0.13		< .2	< .001	22	1.3	< .002	
1524	5/17/2011	Garage Pressure Tank	Upland		Calcium-Bicarbonate	110	< .03	< .001	0.11		< .2	< .001	24	3.9	< .002	201
1525	3/31/2010	Spigot On Porch	Valley				< .1	< .003	0.029	< .05				57.4		341
1526	5/17/2011	Pressure Tank Spigot	Upland		Calcium-Bicarbonate	100	< .03	< .001	0.15		< .2	< .001	22	4	< .002	193
1527	3/31/2010	Hydrant At Well Head	Upland				< .1	< .003	0.144	< .05				6.68		181.1
1528	8/4/2011	Inside Faucet	Valley		Calcium-Bicarbonate	110	< .03	< .001	0.23		< .2	< .001	25	2.5	< .002	276
1529	8/9/2011	Outside Faucet	Upland		Calcium-Bicarbonate	88	<0.03	<0.001	0.11		<0.2	<0.001	22	2.3	<0.002	
1530	3/28/2010	Prssure Tank At Fitzsimon'S House	Upland				< .1	< .003	0.026	< .05				20.2		155.9
1531	5/18/2011	Kitchen Sink	Upland		Calcium-Bicarbonate	100	<0.03	<0.001	0.11		<0.2	<0.001	22	4.1	<0.002	211
1532	5/18/2011	Kitchen Sink	Upland		Sodium-Bicarbonate	140	< .03	< .001	0.3		< .2	< .001	12	5.5	< .002	251
1533	5/18/2011	Kitchen Sink	Upland		Calcium-Bicarbonate-Chloride	24	< .03	< .001	0.047		< .2	< .001	10	8.2	< .002	108
1534	5/18/2011	Kitchen Sink	Upland		Calcium-Sodium-Bicarbonate	110	<0.03	<0.001	0.14		<0.2	<0.001	17	3.2	<0.002	212
1535	3/28/2010	Pressure Tank In Furnace Room	Upland				< .1	< .003	0.114	< .05				7.14		122.9
1536	5/19/2011	Bathroom Sink Body Shop	Valley	Yes	Calcium-Bicarbonate	110	< .03	< .001	0.13		< .2	< .001	22	1.4	< .002	194
1537	4/1/2010	Pressure Tank	Upland				< .1	< .003	0.037	< .1				3.93		
1538	5/24/2011	Basement Pressure Tank	Valley		Sodium-Bicarbonate	200	< .03	< .001	0.66		0.37	< .001	4.1	24	< .002	400
1539	5/24/2011	Kitchen Sink	Upland		Calcium-Bicarbonate	130	< .03	< .001	0.25		< .2	< .001	24	< 1	< .002	285
1540	4/5/2010	Kitchen Sink	Upland				0.19	< .003	0.077	< .1				9.46		
1541	4/5/2010	Kitchen Sink Father'S	Upland				< .1	< .003	0.468	< .1				6.51		

Table S.8. Groundwater Quality Data for 1701 "Pre-Drill" Water Well Samples from Susquehanna County, Pennsylvania

Water Well ID	Sample Date	Sampling Port	Topo-graphic Location	Gas Extraction Area (Within 1 km of Active Gas Well)	Groundwater Type (If Determined)	Alkalinity (mg/L)	Aluminum (mg/L)	Arsenic (mg/L)	Barium (mg/L)	Boron (mg/L)	Bromide (mg/L)	Cadmium (mg/L)	Calcium (mg/L)	Chloride (mg/L)	Chromium (mg/L)	Conductivity - Field (µs/cm)
1542	5/24/2011	Pressure Tank	Valley	Yes	Calcium-Bicarbonate	100	< .03	0.0018	0.092		< .2	< .001	44	21	< .002	0.25
1543	4/1/2010	Kitchen Sink	Upland				< .1	< .003	0.07	< .1				15.4		
1544	4/1/2010	Kitchen Sink	Upland				< .1	< .003	0.082	< .1				12		35.3
1545	6/1/2011	Kitchen Sink	Upland		Calcium-Bicarbonate	170	< .03	0.0013	0.16		< .2	< .001	42	2.1	< .002	302
1546	4/1/2010	Kitchen Sink	Upland				< .1	< .003	0.145	< .1				13.5		223
1547	6/2/2011	Township Bldg Garage Slop Sink	Valley		Calcium-Sodium-Bicarbonate	170	< .03	0.0029	0.15		< .2	< .001	31	3	< .002	278
1548	6/3/2011	Outside Spigot	Valley		Sodium-Bicarbonate	180	< .03	0.025	0.27		< .2	< .001	14	2.9	< .002	303
1549	6/3/2011	Pressure Tank	Valley	Yes	Calcium-Bicarbonate	140	<0.03	0.0011	0.12		<0.2	<0.001	40	14	<0.002	
1550	6/9/2011	Outside Spigot	Valley	Yes	Calcium-Bicarbonate	91	< .03	< .001	0.037		< .2	< .001	34	17	< .002	238
1551	6/9/2011	Kitchen Sink	Upland		Calcium-Bicarbonate	170	< .03	< .001	0.2		< .2	< .001	49	3.4	< .002	322
1552	6/10/2011	Pressure Tank (Basement Back Left Closet)	Valley	Yes	Calcium-Bicarbonate	110	< .03	< .001	0.095		< .2	< .001	33	5.1	< .002	243
1553	6/10/2011		Valley		Calcium-Sodium-Bicarbonate	190	<0.03	0.0013	0.06		<0.2	<0.001	40	1.2	<0.002	
1554	3/28/2010	Outside Spigot	Upland				< .1	< .003	< .025	< .05				3.38		363
1555	4/9/2010	Basement Pressure Tank	Upland				< .1	< .003	0.1	< .1				3.05		110.4
1556	4/9/2010	Kitchen Sink	Valley	Yes			< .1	< .003	0.154	< .1				10.4		116
1557	4/9/2010	Basement Pressure Tank	Valley				0.1	< .003	0.855	0.186				6.36		285
1558	5/27/2010	Garage Sink	Valley				< .1	< .003	0.072	< .1						163.3
1559	5/27/2010		Upland				< .1	< .003	0.116	< .1						
1560	5/27/2010	Outside Spigot	Upland				< .1	< .003	0.062	< .1						67.5
1561	6/3/2010	Main House Kitchen Sink	Upland													
1562	6/4/2010	Outside Hydrant	Upland													276
1563	6/4/2010	Kitchen Sink	Upland	Yes												402

Table S.8. Groundwater Quality Data for 1701 "Pre-Drill" Water Well Samples from Susquehanna County, Pennsylvania

Water Well ID	Sample Date	Sampling Port	Topo-graphic Location	Gas Extraction Area (Within 1 km of Active Gas Well)	Groundwater Type (If Determined)	Alkalinity (mg/L)	Aluminum (mg/L)	Arsenic (mg/L)	Barium (mg/L)	Boron (mg/L)	Bromide (mg/L)	Cadmium (mg/L)	Calcium (mg/L)	Chloride (mg/L)	Chromium (mg/L)	Conductivity - Field (µs/cm)
1564	6/4/2010	Kitchen Sink	Upland													
1565	6/4/2010	Kitchen Sink	Valley													333
1566	6/13/2010	Kitchen Sink	Valley													250
1567	6/13/2010	Hydrant On Well	Valley													266
1568	6/13/2010	Kitchen Sink	Valley													301
1569	6/13/2010	Kitchen Sink	Upland													197
1570	6/13/2010		Valley													
1571	6/13/2010	Kitchen Sink	Valley													84.7
1572	6/12/2010	Pressure Tank	Upland													182.9
1573	6/7/2010	Outside Spigot	Upland													846
1574	6/7/2010	Spring Pipe	Upland													77.7
1575	6/7/2010	Kitchen Sink	Upland													438
1576	6/10/2010	Pressure Tank	Upland				< .1	< .003	0.116	< .1				7.07		156
1577	6/10/2010	Kitchen Sink	Upland													175.5
1578	6/10/2010	Pressure Tank	Upland													168.3
1579	6/10/2010	Kitchen Sink	Upland													336
1580	6/10/2010	Kitchen Sink	Valley													387
1581	6/10/2010		Upland													
1582	6/10/2010	Kitchen Sink	Upland													238
1583	3/25/2009		Valley	Yes			<0.05		<0.025					<2		
1584	6/10/2010	Kitchen Sink	Upland													124.1
1585	6/12/2010	Pressure Tank	Upland													130.1
1586	6/12/2010	Kitchen Faucet	Upland													200
1587	6/12/2010	Bathroom Faucet	Valley													250
1588	6/14/2010	At Pressure Tank	Valley													
1589	6/14/2010	Kitchen Sink	Upland													197.7
1590	4/4/2009		Valley				< .05		0.056					3.7		
1591	6/2/2011	Dug Well	Upland	Yes	Calcium-Bicarbonate	24	0.036	<0.001	0.049		<0.2	<0.001	16	2.3	<0.002	0.102

Table S.8. Groundwater Quality Data for 1701 "Pre-Drill" Water Well Samples from Susquehanna County, Pennsylvania

Water Well ID	Sample Date	Sampling Port	Topo-graphic Location	Gas Extraction Area (Within 1 km of Active Gas Well)	Groundwater Type (If Determined)	Alkalinity (mg/L)	Aluminum (mg/L)	Arsenic (mg/L)	Barium (mg/L)	Boron (mg/L)	Bromide (mg/L)	Cadmium (mg/L)	Calcium (mg/L)	Chloride (mg/L)	Chromium (mg/L)	Conductivity - Field (µs/cm)
1592	3/4/2009		Valley				< .05		0.051					2.95		
1593	4/19/2009		Upland				< .05		0.082					5.3		
1594	9/1/2009		Upland	Yes			< .1		0.107					26.2		
1595	7/19/2011		Upland													
1596	2/20/2009	Barn Sink	Valley				<0.05		0.412					18.4		
1597	2/26/2009	Barn Sink	Upland				<0.05		0.063					<1		
1598	11/9/2009	Pressure Tank	Valley	Yes			< .1		0.523					2.14		
1599	12/6/2009	Kitchen Faucet	Upland				< .1		0.077					2.37		
1600	12/6/2009	Kitchen Faucet	Valley	Yes			< .1		0.091					7.26		
1601	12/6/2009	Pressure Tank	Valley	Yes			< .1		0.098					7.12		
1602	12/6/2009	Kitchen Faucet	Upland				< .1		0.141					27		
1603	12/15/2009	Kitchen Sink	Upland				<0.1		0.102					6.22		
1604	12/17/2009	Pressure Tank	Upland				< .1		0.029					< 2		
1605	1/11/2010	Pressure Tank	Upland				< .1		0.108					9.25		
1606	1/11/2010	Kitchen Faucet	Upland				< .1		0.187					5.38		
1607	1/11/2010	Kitchen Sink	Upland				<0.1		0.089					4.66		
1608	1/11/2010	Pressure Tank	Upland				< .1		0.075					5.02		
1609	1/11/2010	Pressure Tank	Upland				< .1		0.674					3.52		
1610	1/11/2010	Pressure Tank	Upland				< .1		0.286					2.55		
1611	1/12/2010	Spigot Into House	Upland	Yes			0.973		0.076					21.4		
1612	1/16/2010	Pressure Tank	Upland				< .1		0.445					26.1		
1613	1/21/2010	Kitchen Sink	Upland	Yes			<0.1		0.052					43.1		
1614	2/4/2010	Pressure Tank	Valley				<0.1		0.114					18.9		
1615	2/4/2010	Kitchen Sink	Upland				<0.1		0.161					6.08		
1616	2/6/2010	Kitchen Sink	Valley				<.1		0.166					32		
1617	2/15/2010	Kitchen Sink	Valley				<0.1		0.25					10.8		
1618	2/15/2010	Kitchen Sink	Valley				<.1		0.242					11.4		
1619	2/18/2010	Bathroom Sink	Valley	Yes			0.452		0.147					21.5		

Table S.8. Groundwater Quality Data for 1701 "Pre-Drill" Water Well Samples from Susquehanna County, Pennsylvania

Water Well ID	Sample Date	Sampling Port	Topo-graphic Location	Gas Extraction Area (Within 1 km of Active Gas Well)	Groundwater Type (If Determined)	Alkalinity (mg/L)	Aluminum (mg/L)	Arsenic (mg/L)	Barium (mg/L)	Boron (mg/L)	Bromide (mg/L)	Cadmium (mg/L)	Calcium (mg/L)	Chloride (mg/L)	Chromium (mg/L)	Conductivity - Field (µs/cm)
1620	2/22/2010	Kitchen Faucet	Upland				< .1		0.189					12.7		
1621	2/27/2010	Kitchen Sink	Valley				<0.1		0.068					7.07		
1622	3/4/2010	Bathroom Sink	Upland	Yes			0.232		0.061					24.8		
1623	3/7/2010	Pressure Tank	Upland				< .1		0.048					< 2		
1624	3/28/2010	Kitchen Faucet	Upland				< .1		0.896					17		
1625	4/8/2010	Outside Faucet	Upland				< .1	< .003	0.559	< .1				12		
1626	4/9/2010	Kitchen Sink	Valley				<0.1		0.085					5.49		
1627	4/10/2010	Kitchen Sink	Valley				<0.1	<0.003	0.165	<0.1				7.41		
1628	4/10/2010	Pressure Tank	Valley				<0.1	<0.003	1.03	0.196				55.5		
1629	4/19/2010	Kitchen Sink	Valley				<0.10	<0.003	0.066	<0.1				26.9		
1630	4/22/2010	Kitchen Sink	Valley				<0.1	<0.003	0.194					55.5		
1631	4/22/2010	Kitchen Sink	Valley				<0.1	<0.003	0.247	<0.100				40.7		
1632	4/22/2010	Stepmother' S House	Valley				<0.10	<0.003	0.191	<0.1				43.8		
1633	4/24/2010	Kitchen Faucet	Upland	Yes			< .1	< .003	0.07	< .1				4.63		
1634	4/22/2010	Outside Faucet	Valley				< .1	< .003	0.338	< .1				58		
1635	5/1/2010	Kitchen Faucet	Valley	Yes			< .1	0.009	0.126	< .1				2.24		
1636	5/1/2010	Kitchen Sink	Upland				<0.10	<0.003	0.081	<0.10				3.15		
1637	5/21/2010	Pressure Tank	Valley				< .1	0.006	0.05	< .1				4.72		
1638	6/3/2010	Kitchen Faucet	Upland				< .1	< .003	0.043	< .1						
1639	6/7/2010	Outside Spigot	Upland				<0.1	0.006	0.197	<0.1				27.8		
1640	6/12/2010	Spigot In Garage	Valley				<0.1	<0.003	0.08	<0.1				50.8		
1641	6/13/2010	Kitchen Sink	Valley				<0.100	<0.003	0.25	<0.1				22.7		
1642	6/13/2010	Kitchen Sink	Valley											15.3		
1643	6/23/2010	Kitchen Faucet	Upland				< .1	< .003	0.124	< .1				18.8		
1644	7/1/2010	Kitchen Faucet	Valley				< 0.1	< .003	0.156	< .1				3.63		

Table S.8. Groundwater Quality Data for 1701 "Pre-Drill" Water Well Samples from Susquehanna County, Pennsylvania

Water Well ID	Sample Date	Sampling Port	Topo-graphic Location	Gas Extraction Area (Within 1 km of Active Gas Well)	Groundwater Type (If Determined)	Alkalinity (mg/L)	Aluminum (mg/L)	Arsenic (mg/L)	Barium (mg/L)	Boron (mg/L)	Bromide (mg/L)	Cadmium (mg/L)	Calcium (mg/L)	Chloride (mg/L)	Chromium (mg/L)	Conductivity - Field (μs/cm)
1645	7/2/2010	Kitchen Sink	Valley													167
1646	7/6/2010	Kitchen Faucet	Valley				< .1	< .003	0.136	< .1				2.76		
1647	7/6/2010	Kitchen Sink	Upland				<0.1	<0.003	0.259	<0.100				5.73		
1648	7/6/2010	Pressure Tank	Upland				<0.1	<0.003	0.078	<0.1				15.8		
1649	7/7/2010	Kitchen Sink	Upland				<0.1	<0.003	0.157	<0.10				8.69		
1650	7/7/2010	Kitchen Faucet	Valley				< .122	< .003	0.139	< .1				22.3		
1651	7/7/2010	Pressure Tank	Valley											10		
1652	7/8/2010	Kitchen Sink	Upland				<0.1	<0.003	0.176	<0.1				2.46		
1653	7/9/2010	Pressure Tank	Valley				< .1	< .003	0.431	< .1				3.96		
1654	7/2/2010	Kitchen Faucet	Valley				< .1	< .003	0.333					8.35		
1655	7/9/2010	Outside Spigot	Valley				<0.1	<0.003	0.141	<0.1				4.13		
1656	7/9/2010	Kitchen Sink	Valley				<0.100	<0.003	0.137	<0.100				3.22		
1657	7/7/2010	Kitchen Faucet	Upland	Yes			< .1	< .003	0.16	< .1				25.6		
1658	7/7/2010	Kitchen Sink	Upland				<0.1	<0.003	0.172	<0.100				35.8		
1659	7/8/2010	Kitchen Faucet	Valley				< .1	0.003	0.132	< .1				2.57		
1660	7/8/2010	Kitchen Sink	Upland				<0.100	<0.003	0.045	<0.100				26.3		
1661	7/10/2010	Pressure Tank	Upland				< .1	< .003	0.078	< .1				7.43		
1662	7/10/2010	Basement Spigot Pre-Filter	Valley				<0.100	0.005		<0.1				3.43		
1663	7/10/2010	Kitchen Faucet	Upland				< .1	0.004	0.028	< .1				4.7		
1664	7/10/2010	Pressure Tank	Upland				<0.1	<0.003	0.088	<0.1				3.55		
1665	7/11/2010	Kitchen Faucet	Upland				< .1	< .003	0.183	< .1				5.3		
1666	7/12/2010	Kitchen Faucet	Valley				< .1	< .003	1.13	< .1				3.4		
1667	7/13/2010	Basement (Pre-Filter)	Valley	Yes			< .1	0.003	0.113	< .1				3.56		
1668	7/13/2010	Kitchen Sink	Valley				<0.1	<0.003	0.07	<0.1				2.25		
1669	7/13/2010	Deep Well	Valley				< .1	< .003	0.71	0.122				28.8		

Table S.8. Groundwater Quality Data for 1701 "Pre-Drill" Water Well Samples from Susquehanna County, Pennsylvania

Water Well ID	Sample Date	Sampling Port	Topo-graphic Location	Gas Extraction Area (Within 1 km of Active Gas Well)	Groundwater Type (If Determined)	Alkalinity (mg/L)	Aluminum (mg/L)	Arsenic (mg/L)	Barium (mg/L)	Boron (mg/L)	Bromide (mg/L)	Cadmium (mg/L)	Calcium (mg/L)	Chloride (mg/L)	Chromium (mg/L)	Conductivity - Field (µs/cm)
1670	7/28/2010	Kitchen Faucet	Valley				< .1	< .003	0.177	< .1				3.29		
1671	7/28/2010	Milkhouse Sink	Upland	Yes			<0.1	<0.003	0.0969					3.65		
1672	8/7/2010	Kitchen Sink	Upland				<0.10	<0.003	0.128	<0.1				9.24		
1673	8/8/2010	Kitchen Sink	Upland				<0.1	<0.003	<0.025	<0.1				3.11		
1674	8/13/2010	Outside Hydrant	Valley				< .1	< .003	< .025	< .1				13.9		
1675	8/19/2010	Kitchen Faucet	Valley	Yes		25								21.8		
1676	9/23/2010	Outside Spigot	Upland		Calcium-Sodium-Bicarbonate	125	<0.1	<0.003	1.68			<0.002	25.2	4.88	<0.005	
1677	10/10/2010		Valley	Yes			<0.05	<0.002	0.107			<0.002	21.9	37.8	<0.005	
1678	10/18/2010	Kitchen Sink	Valley	Yes		75	<0.05	<0.003	0.086			<0.002	41.4		<0.005	
1679	10/18/2010	Kitchen Faucet	Upland	Yes		145								20		
1680	10/19/2010	Kitchen Faucet	Valley	Yes		65								31.5		
1681	10/26/2010	Spring House	Valley	Yes		40	<0.05	<0.003	0.059			<0.002	19.6	4.7	<0.005	
1682	10/29/2010	Kitchen Faucet	Upland		Calcium-Bicarbonate	125	<0.05	<0.003	0.092			<0.002	41.2	4.41	<0.005	
1683	10/29/2010	Kitchen Faucet	Upland		Calcium-Bicarbonate	145	<0.05	<0.003	0.094			<0.002	54.7	12	<0.005	
1684	11/11/2010	Kitchen Faucet	Valley	Yes		190								19.4		
1685	11/11/2010	Kitchen Faucet	Upland			130								6.59		
1686	11/11/2010	Kitchen Faucet	Upland	Yes	Calcium-Bicarbonate	155	<0.01	<0.001	0.1			<0.00010	41	4.48	<0.002	
1687	12/2/2010	Kitchen Sink	Valley		Calcium-Bicarbonate	110	<0.05	<0.003	<0.025			<0.002	43.4	17.8	<0.005	
1688	12/4/2010	Kitchen Sink	Valley			30	<0.05	<0.003	0.036			<0.002	11.1		<0.005	
1689	12/5/2010	Kitchen Faucet	Valley	Yes		140								3.58		
1690	12/9/2010	Kitchen Faucet	Upland			125								2.08		
1691	12/11/2010	Kitchen Sink	Valley			60								<2		
1692	12/14/2010	Kitchen Sink	Upland	Yes	Calcium-Bicarbonate-Chloride	30	0.017	<0.001	0.069			0	14	7.1	<0.002	
1693	12/14/2010	Kitchen Faucet	Upland	Yes		115	<0.01	0.0014	0.093			0	40		0	
1694	12/18/2010	Kitchen Faucet	Valley	Yes		195								2.87		

Table S.8. Groundwater Quality Data for 1701 "Pre-Drill" Water Well Samples from Susquehanna County, Pennsylvania

Water Well ID	Sample Date	Sampling Port	Topo-graphic Location	Gas Extraction Area (Within 1 km of Active Gas Well)	Groundwater Type (If Determined)	Alkalinity (mg/L)	Aluminum (mg/L)	Arsenic (mg/L)	Barium (mg/L)	Boron (mg/L)	Bromide (mg/L)	Cadmium (mg/L)	Calcium (mg/L)	Chloride (mg/L)	Chromium (mg/L)	Conductivity - Field (μs/cm)
1695	10/29/2009	Pressure Tank	Upland	Yes			< .1		0.255					100		
1696	11/6/2010	Kitchen Faucet	Upland		Sodium-Bicarbonate	110	< .01	< .001	< .002			< .0001	< .5	7.55	< .002	
1697	3/19/2009		Upland	Yes			< .05		0.187					8.03		
1698	3/25/2009		Upland				<0.05		0.093					4.99		
1699	3/12/2009		Upland				<0.05		0.079					9.19		
1700	4/5/2010	Kitchen Sink, Main House	Upland				<0.100	<0.003	0.131	<0.100				5.4		
1701	6/2/2011		Valley		Calcium-Sodium-Bicarbonate	150	< .03	0.0023	0.16		< .02	< .001	36	2	< .002	

Table S.8. Groundwater Quality Data for 1701 "Pre-Drill" Water Well Samples from Susquehanna County, Pennsylvania

Water Well ID	Sample Date	Sampling Port	Topo-graphic Location	Gas Extraction Area (Within 1 km of Active Gas Well)	Dissolved Oxygen - Field (mg/L)	Ethane ug/L	Hardness (mg/L)	Iron (mg/L)	Lead (mg/L)	Magnesium (mg/L)	Manganese (mg/L)	Mercury (mg/L)	Methane ug/L	Nitrate as N (mg/L)	Oil Grease (mg/L)	pH - Field (SU)	Potassium (mg/L)
1	6/3/2011		Valley			<26	47	0.05	<0.001	2.3	<0.0005	<0.0002	<26	1.2	<4.7		0.93
2	5/27/2010	Pressure Tank	Valley		10.42	5.7							17000			7.67	
3	6/29/2011		Upland		3.21	<26	93	0.12	<0.001	5.1	0.0092	<0.0002	<26	0.88		8.16	0.96
4	6/29/2011		Upland			< 26	23	2.9	0.0018	1.9	0.049	< .0002	< 26	< .05			0.93
5	6/17/2010	Outside Spigot	Upland	Yes	5.53								0.11			6.33	
6	6/17/2010	Kitchen Sink	Valley		8.07								0.11			6.3	
7	6/17/2010	Kitchen Sink	Upland		10.55								0.23			6.9	
8	4/10/2010	Pressure Tank	Upland		12.18			< .05		1.86	< .025		1.8			5.98	
9	4/10/2010	Basement At Pressure Tank	Upland		5.14			< .05		7.92	< .025		0.91			7.31	
10	4/10/2010	Pressure Tank	Valley		8.05			< .05		4.87	< .025		0.39			6.85	
11	4/11/2010	Outside Hydrant Directly From Well	Upland		5.12			< .05		4.65	< .025		1.7			6.55	
12	4/11/2010		Valley					< .05		5.06	< .025		0.3				
13	4/11/2010	Kitchen Sink	Valley		3.48			< .05		1.18	0.042		1400			7.49	
14	4/11/2010	Kitchen Sink	Upland	Yes	4.2			< .05		6.52	< .025		< .1			7.51	
15	4/11/2010	Pressures Tank	Upland		8.6			< .05		9.37	< .025		0.71			6.81	
16	4/11/2010	Kitchen Sink	Valley		3.4			< .05		1.86	< .025		1300			8.74	
17	4/11/2010	Kitchen Sink	Upland					< .05		8.58	0.086		28				
18	4/11/2010	Outside Hydrant Direct From Well	Upland					< .05		7.92	0.095		230				
19	4/15/2010	Outside Hydrant	Upland			< .025		< .05		6.54	< .025		1.4				
20	4/15/2010	Kitchen Sink	Upland			< .025		< .05		5.73	< .025		3.2				
21	4/15/2010	Outside Hydrant	Valley					< .05		5.81	< .025		0.79				
22	4/15/2010	Pressure Tank	Valley					< .05		5.3	< .025		0.16				
23	4/19/2010	Laundry Room Sink	Upland					< .05		5.05	< .025		< .1				

Table S.8. Groundwater Quality Data for 1701 "Pre-Drill" Water Well Samples from Susquehanna County, Pennsylvania

Water Well ID	Sample Date	Sampling Port	Topo-graphic Location	Gas Extraction Area (Within 1 km of Active Gas Well)	Dissolved Oxygen - Field (mg/L)	Ethane ug/L	Hardness (mg/L)	Iron (mg/L)	Lead (mg/L)	Magnesium (mg/L)	Manganese (mg/L)	Mercury (mg/L)	Methane ug/L	Nitrate as N (mg/L)	Oil Grease (mg/L)	pH - Field (SU)	Potassium (mg/L)
24	4/19/2010	Hydrant At Well Head	Valley					< .05		10	< .025		14				
25	4/19/2010	Diversion Ditch	Upland	Yes				0.353		2.63	0.086		1.2				
26	4/22/2010	Kitchen Sink	Valley					< .05		12.4	< .025		2.9				
27	4/22/2010	Kitchen Sink	Upland	Yes				< .05		7.52	< .025		57				
28	4/22/2010	Basement Sink	Upland					< .05		6.54	< .025		0.32				
29	6/24/2010	Pond	Upland		8.9								68			7.06	
30	6/24/2010	Pressure Tank	Upland		8.76								0.14			7.16	
31	5/14/2011		Upland		0.55	< 26	130	0.5	0.0012	7.2	4.1	< .0002	< 26	0.29		6.77	0.98
32	6/24/2010	Kitchen Sink	Valley		1.71								18000			7.45	
33	6/24/2010	Outside Spigot	Upland		9.52	< .025		< .05		3.57	< .025		0.12			6.89	
34	4/25/2010	Bathroom Faucet	Upland	Yes	10.04			< .05		2.96	< .025		0.62			6.76	
35	4/25/2010		Upland	Yes				0.098		5.27	0.059		0.37				
36	4/24/2010	Outside Spigot	Valley					< .05		15.5	< .025		0.77				
37	4/24/2010	Kitchen Sink	Upland	Yes	10.14			< .05		2.01	< .025		0.79			6.37	
38	4/24/2010		Upland					< .05		6.26	< .025		0.24				
39	4/24/2010	Kitchen Bar Sink	Upland					< .05		12.3	< .025		0.31				
40	4/24/2010	Outside Spigot	Upland					< .05		5.28	< .025		1				
41	4/24/2010	Outside Spigot	Upland					< .05		6.14	< .025		0.3				
42	4/29/2010	Outside Spigot	Upland					< .05		6.31	< .025		< .1				
43	4/29/2010	Spring Culvert	Upland					< .05		1.23	< .025		0.74				
44	4/29/2010	Basement At Pressure Tank	Valley					< .05		5.79	< .025		0.2				
45	7/2/2010	Kitchen Sink	Valley		2.59	< .025							16			7.61	
46	7/2/2010	Bathroom Sink	Valley		5.31	< .025							< .1			7.15	
47	7/2/2010	Kitchen Sink	Valley		25.6	< .025							0.28			7.19	
48	7/2/2010	Kitchen Sink	Valley		4.88	< .025							0.22			7.37	
49	7/2/2010	Kitchen Sink	Upland		5.53	< .025							0.19			6.95	

Table S.8. Groundwater Quality Data for 1701 "Pre-Drill" Water Well Samples from Susquehanna County, Pennsylvania

Water Well ID	Sample Date	Sampling Port	Topo-graphic Location	Gas Extraction Area (Within 1 km of Active Gas Well)	Dissolved Oxygen - Field (mg/L)	Ethane ug/L	Hardness (mg/L)	Iron (mg/L)	Lead (mg/L)	Magnesium (mg/L)	Manganese (mg/L)	Mercury (mg/L)	Methane ug/L	Nitrate as N (mg/L)	Oil Grease (mg/L)	pH - Field (SU)	Potassium (mg/L)
50	7/2/2010	Kitchen Sink	Upland		6.93	< .025							< .1			7.15	
51	7/2/2010	Kitchen Sink	Valley		6.46	< .025							0.36			7.04	
52	7/6/2010	Kitchen Sink	Upland		6.55	< .025		< .05		4.33	< .025		< .1			7.61	
53	7/6/2010	Garage	Valley		8.21	< .025		< .05		6.91	0.294		38			7.8	
54	7/6/2010	Kitchen Sink	Valley		8.9	< .025		< .05		2.71	< .025		< .1			7.55	
55	7/6/2010	Kitchen Sink	Upland		6.38	< .025		< .05		< 1	< .025		2.9			7.77	
56	7/6/2010	Kitchen Sink	Valley		1.76	1.8		< .05		4.53	0.025		9500			7.01	
57	7/6/2010		Valley			< .025		< .05		3.67	< .025		3.2				
58	7/6/2010	Kitchen Sink	Valley		5.41	< .025		< .05		3.97	< .025		0.63			8.06	
59	7/6/2010	Basement At Pressure Tank	Valley		6.08	< .025		< .05		2.58	< .025		39			5.58	
60	7/6/2010		Upland			< .025		< .05		2.54	< .025		0.11				
61	7/7/2010	Kitchen Sink	Valley		5.64	< .025		< .05		6.01	< .025		0.74			6.73	
62	7/7/2010	Spring Head	Valley		6.08	< .025		< .05		2.21	< .025		1.1			5.95	
63	7/7/2010	Kitchen Sink	Valley		7.89	0.071		0.072		2.66	0.052		140			6.35	
64	7/7/2010	Kitchen Sink	Valley		5.14	< .025		< .05		4.21	< .025		0.18			7.55	
65	7/7/2010	Kitchen Sink	Valley		7.89	< .025		< .05		3.68	< .025		0.11			6.49	
66	7/7/2010	Basement Pressure Tank	Valley		4.13	< .025		< .05		4.74	< .025		19			5.92	
67	7/7/2010	Kitchen Sink	Valley		1.77	0.075		< .05		6.13	< .025		610			6.51	
68	7/8/2010	Basement Sink	Valley		3.14	< .025		< .05		6.59	< .025		1.1			8.15	
69	7/8/2010	Kitchen Sink	Upland		4.15	< .025		< .05		5.73	< .025		0.12			8.01	
70	7/8/2010	Kitchen Sink	Valley		2.98	< .025		< .05		6.39	< .025		3.4			7.73	
71	7/9/2010	Kitchen Sink	Valley		2.13	< .025		< .05		7.11	< .025		0.16			7.04	
72	7/9/2010	Kitchen Sink - Debonis Property	Valley		8.22	< .025		< .05		3.76	< .025		0.23			6.37	
73	7/7/2010	Kitchen Sink	Valley		2.54	< .025		< .05		4.91	< .025		0.81			8.09	
74	7/7/2010	Outside Garage Spigot	Valley		8.48	< .025		< .05		3.15	< .025		0.15			8.04	

Table S.8. Groundwater Quality Data for 1701 "Pre-Drill" Water Well Samples from Susquehanna County, Pennsylvania

Water Well ID	Sample Date	Sampling Port	Topo-graphic Location	Gas Extraction Area (Within 1 km of Active Gas Well)	Dissolved Oxygen - Field (mg/L)	Ethane ug/L	Hardness (mg/L)	Iron (mg/L)	Lead (mg/L)	Magnesium (mg/L)	Manganese (mg/L)	Mercury (mg/L)	Methane ug/L	Nitrate as N (mg/L)	Oil Grease (mg/L)	pH - Field (SU)	Potassium (mg/L)
75	7/8/2010	Kitchen Sink	Valley			< .025		< .05		2.37	< .025		0.12				
76	7/7/2010	Kitchen Sink	Valley		2.93	< .025		< .05		6.48	< .025		0.7			7.7	
77	7/7/2010	Kitchen Sink	Valley		6.8	< .025		< .05		2.02	< .025		< .1			7.55	
78	7/8/2010	Kitchen Sink	Valley		6.25	0.06		< .05		5.02	0.054		440			6.83	
79	7/8/2010	Kitchen Sink	Upland		8.28	< .025		< .05		6.1	< .025		0.6			7.3	
80	7/8/2010	Kitchen Sink	Valley		6.46	0.029		< .05		6.04	< .025		1.4			7.3	
81	7/8/2010	Kitchen Sink	Valley		11.35	< .025		< .05		5.88	< .025		0.26			7.31	
82	7/9/2010		Valley	Yes		< .025		< .05		3.76	< .025		0.12				
83	7/9/2010	Kitchen Sink	Valley		2.49	< .025		< .05		6.26	< .025		0.33			6.49	
84	7/9/2010	Kitchen Sink	Valley		2.87	< .025		< .05		6.88	< .025		0.44			8.25	
85	7/9/2010	Kitchen Sink	Upland		2.63	< .025		< .05		4.87	< .025		0.12			7.64	
86	7/9/2010	Kitchen Sink	Valley		0.52	< .025		< .05		3.57	0.026		4.5			8.08	
87	7/9/2010		Valley			< .025		< .05		2.34	< .025		0.34				
88	7/10/2010		Valley			7.2		< .05		10.6	0.09		17000				
89	7/8/2010	Pressure Tank	Upland		7.12			< .05		4.22	< .025		< .1			7.07	
90	5/20/2010	Kitchen Sink	Valley		3.07			< .05		10.9	0.096		7.3			7.2	
91	7/11/2010	Kitchen Sink	Valley		1.35	< .025		< .05		6.27	< .025		1.3			7.6	
92	7/11/2010	Barn Hose	Valley			<0.025		0.106		3.24	<0.025		12				
93	7/8/2010	Kitchen Sink	Upland	Yes	6.38	< .025		< .05		6.89	< .025		< .1			7.42	
94	4/8/2009		Upland			<0.025		<0.05		8.3	<0.025		1.9		<5	7.61	
95	7/9/2010		Valley			< .025		< .05		4.04	< .025		0.34				
96	7/10/2010	Kitchen Sink	Valley		2.33	< .025		< .05		6.99	< .025		0.17			7.74	
97	7/10/2010	Kitchen Sink	Upland		1	< .025		< .05		1.21	0.757		610			6.2	
98	7/12/2010	Artesian Well	Valley			< .025		< .05		3.19	< .025		3				
99	7/12/2010		Valley			4.9		< .05		2.96	< .025		13000				
100	7/12/2010	Kitchen Sink	Valley		0.27	17		< .05		4.81	0.279		25000			7.73	
101	7/12/2010		Valley			15		< .05		7.37	0.04		27000				
102	7/12/2010		Valley			< .025		< .05		6.29	< .025		< .1				
103	7/11/2010	Kitchen Sink	Valley	Yes	0.87	13		< .05		6.15	0.066		22000			8.46	

Table S.8. Groundwater Quality Data for 1701 "Pre-Drill" Water Well Samples from Susquehanna County, Pennsylvania

Water Well ID	Sample Date	Sampling Port	Topo-graphic Location	Gas Extraction Area (Within 1 km of Active Gas Well)	Dissolved Oxygen - Field (mg/L)	Ethane ug/L	Hardness (mg/L)	Iron (mg/L)	Lead (mg/L)	Magnesium (mg/L)	Manganese (mg/L)	Mercury (mg/L)	Methane ug/L	Nitrate as N (mg/L)	Oil Grease (mg/L)	pH - Field (SU)	Potassium (mg/L)
104	7/12/2010	Kitchen Sink	Valley			< .025		< .05		2.92	< .025		0.29				
105	7/13/2010		Upland			< .025		< .05		7.73	< .025		0.75				
106	7/12/2010	Kitchen Sink	Valley			11		< .05		1.31	< .025		24000				
107	7/17/2010	Kitchen Sink	Valley		2.84	< .025		< .05		6.27	0.075		11			7.73	
108	7/17/2010	Kitchen Sink	Valley		5.37	< .025		< .05		6.55	< .025		0.86			7.5	
109	7/17/2010	Kitchen Sink	Valley		6.8	< .025		< .05		3.68	< .025		0.28			7.09	
110	7/11/2010		Upland			< .025		< .05		5.53	0.132		3.6				
111	7/13/2010	Kitchen Sink	Valley			< .025		< .05		4.66	< .025		< .1				
112	7/13/2010	Kitchen Sink	Valley			0.028		< .05		5.25	0.042		85				
113	7/12/2010		Valley			< .025		< .05		4.43	0.087		0.63				
114	7/25/2011	Kitchen Sink	Upland		1.46	1.3							4300			7.89	
115	8/17/2011	Bathroom Sink	Valley		2.29	0.99							6600			7.97	
116	7/5/2011		Valley		3.06	<0.025							0.83			7.77	
117	3/12/2009	Barn	Valley		4.72	5.9		< .05		5.04	0.06		4200		< 5	8.23	
118	2/20/2009	Basement	Upland		10.64			0.108		2.27	< .025		0.12			6.06	
119	7/1/2010		Valley			< .025		< .05		2.65	< .025		0.35				
120	7/1/2010		Upland			< .025		< .05		3.1	< .025		0.92				
121	2/20/2009		Upland					0.212		5.79	1.9		0.99		< 5		
122	8/3/2010	Hand Dug Well	Upland		7.46	< .025		< .05		5.82	< .025		0.22			5.69	
123	2/20/2009	Basement	Valley		5			0.064		7.9	0.513		1.5			7.74	
124	8/7/2010	Kitchen Sink	Upland		2.44	0.12		< .05		9.82	0.067		430			7.48	
125	8/7/2010	Hand Dug Well	Upland		4.61	< .025		< .05		3.58	< .025		0.8			8.24	
126	8/8/2010	Pressure Tank	Valley			0.006		0.066		9.33	0.048		4.1				
127	8/8/2010	Kitchen Sink	Valley		3.19	0.03		< .05		< 1	< .025		140			8.07	
128	8/8/2010	Bathroom Sink	Valley		3.72	< .025		< .05		< 1	< .025		2			7.8	
129	8/8/2010	Kitchen Sink	Upland		6.24	< .025		< .05		6.08	< .025		0.12			6.62	
130	7/30/2010	Bathroom Sink	Valley	Yes	2.99	11		< .05		6.21	0.094		16000			7.74	
131	8/13/2010	Outside Spigot	Upland		6.09	< .025		< .05		3.62	< .025		0.3			7.17	
132	7/20/2010	Kitchen Sink	Valley		5.69	< .025		< .05		8.59	< .025		0.21			7.18	
133	7/20/2010	Heefer Barn	Upland		5.04	< .025		< .05		12.7	< .025		0.4			7.19	
134	8/11/2010	House #2	Valley		2.08	< .025		< .05		1.54	0.039		38			7.43	
135	4/15/2009		Upland			< .025		< .05		3.76	< .025		0.35		< 5		

Table S.8. Groundwater Quality Data for 1701 "Pre-Drill" Water Well Samples from Susquehanna County, Pennsylvania

Water Well ID	Sample Date	Sampling Port	Topo-graphic Location	Gas Extraction Area (Within 1 km of Active Gas Well)	Dissolved Oxygen - Field (mg/L)	Ethane ug/L	Hardness (mg/L)	Iron (mg/L)	Lead (mg/L)	Magnesium (mg/L)	Manganese (mg/L)	Mercury (mg/L)	Methane ug/L	Nitrate as N (mg/L)	Oil Grease (mg/L)	pH - Field (SU)	Potassium (mg/L)
136	8/11/2010	Main House Kitchen Sink	Valley		8.12	< .025		< .05		9.72	< .025		< .1			7.98	
137	8/19/2010		Upland	Yes		< .025	106.4333	< .05	0.005	4.32	< .025	< .0002	0.14	< 1			1.01
138	8/19/2010	Barn Well Milkhouse Sink	Upland	Yes	4.37	< .025	110.3356	< .05	< .001	4.54	< .025	< .0002	0.31	< 1		6.77	0.851
139	6/24/2009	Basement Pressure Tank	Valley		10.71	< .025		0.154		2.39	< .025		2.2		< 5	7.19	
140	8/19/2010	Pressure Tank	Upland		4.62	< .025	95.24654	0.052	0.02	3.18	< .025	< .0002	0.38	< 1		7.49	1.76
141	8/19/2010	Kitchen Sink	Upland	Yes	3.92	< .025	154.771	< .05	< .001	5.75	< .025	< .0002	< .1	< 1		7.31	1.49
142	8/19/2010	Spigot Under Mobile Home	Upland	Yes	4.19	< .025	119.3432	< .05	< .001	4.12	< .025	< .0002	0.15	< 1		7.25	1.43
143	8/20/2010	Laundry Room Sink	Upland		2.57	10	73.81654	< .05	< .001	5.98	< .025	< .0002	16000	< 1		7.87	1.05
144	6/24/2009	Well Head	Valley		2.54	< .025		0.122		3.07	< .025		85		< 5	8.86	
145	6/24/2009	Kitchen Sink	Valley		3.85	< .025		< .025		3.88	< .025		0.13		< 5	7.72	
146	8/26/2010	Outside Spigot	Upland		4.61	< .025	137.718	< .05	< .001	7.43	0.064	< .0002	1.6	< 1		6.88	1.14
147	7/29/2010		Valley			< .025		< .05		15.4	< .025		0.087				
148	7/29/2010	Kitchen Sink	Upland	Yes	4.18	0.034		0.069		3.86	0.04		120			7.23	
149	8/28/2010	Pressure Tank	Valley		5.48	< .025	154.1148	< .05	0.001	< 12.2	< .025	< .0002	0.22	< 1		7.11	1.4
150	8/28/2010	Outside Spigot	Valley		4.34	< .025	131.8529	0.542	< .001	5.46	< .025	< .0002	3.4	< 1	< 5	6.98	0.9
151	9/2/2010	Kitchen Sink	Upland		3.15	< .025	162.0584	< .05	< .002	9.46	< .025	< .0002	1.9	< 1		6.97	1.4
152	6/13/2011	Kitchen Sink	Valley		6.02	0.03							120			7.14	
153	9/10/2010	Kitchen Faucet	Valley		9.5	< .025	52.2	< .05	< .09	3.27	< .025	< .0002	7.6	< 1		7.66	1.95
154	7/20/2010	Tenant House Kitchen Sink	Upland		3.18	< .025		< .05		9.16	< .025		0.28			7.28	
155	2/27/2010	Kitchen Sink	Upland		7.06	< .025		< .05		5.48	< .025		0.2			6.97	
156	2/22/2010	Basement At Pressure Tank	Valley		3.39	< .025		0.113		7.22	0.071		19			7.97	

Table S.8. Groundwater Quality Data for 1701 "Pre-Drill" Water Well Samples from Susquehanna County, Pennsylvania

Water Well ID	Sample Date	Sampling Port	Topo-graphic Location	Gas Extraction Area (Within 1 km of Active Gas Well)	Dissolved Oxygen - Field (mg/L)	Ethane ug/L	Hardness (mg/L)	Iron (mg/L)	Lead (mg/L)	Magnesium (mg/L)	Manganese (mg/L)	Mercury (mg/L)	Methane ug/L	Nitrate as N (mg/L)	Oil Grease (mg/L)	pH - Field (SU)	Potassium (mg/L)
157	9/17/2010	Kitchen Sink	Valley		3.7	0.71	48.87348	< .05	< .001	4.41	< .025	< .0002	5100	< 1		7.59	1.76
158	1/10/2010	Basement Pressure Tank	Upland		49	< .025		< .05		6.49	< .025		0.71			7.55	
159	10/3/2010	Kitchen Sink	Valley	Yes	2.24	< .025	169.3858	< .05	< .001	11.3	< .025	< .0002	2	< 1	< 5	6.6	1.5
160	1/31/2011	Kitchen Sink	Upland		9.64	< .025	74.341	< .05	< .0003	3.5	< .0025	< .0002	0.34	< 1	< 5	6.99	1.5
161	4/8/2009	Laundry Sink	Upland		1.82	< .025		< .025		13.3	< .025		1.3		< 5	7.59	
162	10/7/2010	Kitchen Sink	Upland		3.12	< .025	123.8789	0.087	< .001	4.13	< .025	< .0002	0.68		< 5	6.93	1.6
163	10/7/2010	Kitchen Sink	Valley	Yes	4.14	< .025	127.8824	0.37	< .001	6.8	0.081	< .0002	0.2		< 5	6.56	1.1
164	10/7/2010		Valley	Yes		< .025	140.145	1.31	0.034	8.08	0.136	< .0002	4.7	< 1	< 5		1.1
165	4/8/2009	Boiler Room	Upland		5.3	< .025		< .025		8.4	< .025		8.1			8.99	
166	7/14/2011	Kitchen Sink	Upland		10.41	< .025							0.31			7.24	
167	7/14/2011	Kitchen Sink	Upland		10.41	< .025							0.14			7.76	
168	10/8/2010	Kitchen Faucet	Valley	Yes	3.92	< .025	96.3981	< .05	< .001	5.4	< .025	< .0002	0.48	< 1	< 5	6.99	1.2
169	10/8/2010	Outside Spigot	Valley	Yes	6.21	< .025	140.6954	0.477	< .005	8.82	< .025	< .0002	1.5	< 1	< 5	7.27	1.2
170	7/22/2010	Pressure Tank	Upland	Yes	5.32	0.037		< .05		3.55	0.105		210			7.69	
171	4/8/2009	Career & Tech Bldg Pressure Tank	Upland		5.97	0.062		< .025		1.23	< .025		59		< 5	8.37	
172	4/8/2009	Boiler Room	Upland		2.47	< .025		< .025		5.72	< .025		17		< 5	7.93	
173	10/1/2010	Kitchen Sink	Valley	Yes	4.23	7.7	43.24492	0.051	< .001	2.74	< .025	< .0002	22000	< 1	< 5	7.81	2
174	11/5/2009	Pressure Tank	Upland	Yes		< .025		< .05		6.1	< .025		0.32			7.36	
175	7/14/2011	Spigot On Side Of House	Valley		4.42	< .025							1.6			7.63	
176	7/14/2011	Kitchen Sink	Valley		7.34	< .025							0.3			7.87	
177	7/14/2011	Kitchen Sink	Valley		3.82	< .025							1.9			6.82	
178	7/14/2011	Kitchen Sink	Upland		2.54	< .025							0.36			8.13	
179	7/14/2011	Kitchen Sink	Upland		8.78	< .025							< .1			6.62	

Table S.8. Groundwater Quality Data for 1701 "Pre-Drill" Water Well Samples from Susquehanna County, Pennsylvania

Water Well ID	Sample Date	Sampling Port	Topo-graphic Location	Gas Extraction Area (Within 1 km of Active Gas Well)	Dissolved Oxygen - Field (mg/L)	Ethane ug/L	Hardness (mg/L)	Iron (mg/L)	Lead (mg/L)	Magnesium (mg/L)	Manganese (mg/L)	Mercury (mg/L)	Methane ug/L	Nitrate as N (mg/L)	Oil Grease (mg/L)	pH - Field (SU)	Potassium (mg/L)
180	10/10/2010	Kitchen Sink Rental Property	Valley	Yes	2.74	< .025	197.4357	0.077	0.001	13.2	< .025	< .0002	1.1	< 1	< 5	5.95	1.4
181	4/15/2009	Pressure Tank	Valley	Yes	8.77	< .025		< .05		4.69	< .025		0.23		< 5	7.65	
182	10/10/2010	Kitchen Sink	Valley	Yes	3.07	< .025	121.851	0.232	0.002	6.73	< .025	< .0002	0.44	< 1	< 5	6.91	1.2
183	11/4/2010	Kitchen Sink	Valley		5.63	< .025	83.7338	< .05	0.0006	7.6	< .0025	< .0002	0.18	< 1	< 5	6.59	1.06
184	11/4/2010	Kitchen Sink	Upland		5.98	< .025	96.1402	0.074	0.00084	9.4	< .0025	< .0002	0.17	< 1	< 5	6.84	1.35
185	3/12/2009	Basement Before Filter	Upland	Yes	8.66	0.25		< .05		2.13	0.027		3000		< 5	6.34	
186	10/10/2010	Outside Spigot	Valley	Yes	4.94	< .025	129.1695	0.07	< .001	6.87	< .025	< .0002	0.12	< 1	< 5	6.86	1.3
187	11/4/2010	Pressure Tank	Valley		5.28	0.23	67.9806	< .05	0.001	6.2	0.0095	< .0002	1100	< 1	< 5	7.41	1.3
188	11/6/2010	Kitchen Sink	Valley	Yes	6.14	< .025	104.5858	< .05	< .0003	6.6	0.03	< .0002	0.21	< 1	< 5	6.64	1.16
189	11/6/2010	Outside Spigot	Valley		3.9	< .025	95.7546	< .05	0.0045	8.7	< .0025	< .0002	< .1	< 1	< 5	6.88	1.27
190	11/7/2010	Kitchen Sink	Valley	Yes	4.16	< .025	189.248	0.14	0.00062	12	0.025	< .0002	< .1	< 1	< 5	6.85	1.28
191	11/7/2010	Pressure Tank	Valley	Yes	7.01	< .025	137.3538	0.13	0.008	9.1	< .0025	< .0002	0.11	< 1	< 5	6.27	1.42
192	11/7/2010	Kitchen Sink	Valley	Yes	5.2	< .025	160.16	0.087	< .00013	11	0.013	< .0002	12	< 1	< 5	6.71	1.22
193	10/13/2010	Kitchen Faucet	Valley	Yes	4.35	< .025	137.2755	0.109	< .001	7.08	< .025	< .0002	0.32	< 1	< 5	6.46	1.38
194	10/13/2010	Pressure Tank	Valley	Yes	6.07	< .025	141.0503	< .05	< .001	9.27	< .025	< .0002	0.18	< 1	< 5	7.13	1.5
195	10/13/2010	Pressure Tank	Valley	Yes	3.84	< .025	115.6733	< .05	0.003	6.2	< .025	< .0002	0.16	< 1	< 5	5.87	1.59
196	10/13/2010	Kitchen Sink	Valley	Yes	3.51	< .025	124.0527	< .05	< .001	5.87	< .025	< .0002	< .1	< 1	< 5	6.38	1.5
197	10/11/2010	Kitchen Sink	Valley	Yes	7.94	< .025	49.3272	< .05	0.001	3.55	< .025	< .0002	0.16	< 1	< 5	5.45	1.3
198	10/18/2010	Bathroom Sink	Valley			<0.025		0.064	<0.001	8.96	4.79	<0.0002	50	<1	<5		1.29
199	10/2/2010	Kitchen Sink Faucet	Upland		2.79	< .025	88.43128	< .05	0.001	4.86	0.183	< .0002	180	< 1	< 5	7.19	1.7

Table S.8. Groundwater Quality Data for 1701 "Pre-Drill" Water Well Samples from Susquehanna County, Pennsylvania

Water Well ID	Sample Date	Sampling Port	Topo-graphic Location	Gas Extraction Area (Within 1 km of Active Gas Well)	Dissolved Oxygen - Field (mg/L)	Ethane ug/L	Hardness (mg/L)	Iron (mg/L)	Lead (mg/L)	Magnesium (mg/L)	Manganese (mg/L)	Mercury (mg/L)	Methane ug/L	Nitrate as N (mg/L)	Oil Grease (mg/L)	pH - Field (SU)	Potassium (mg/L)
200	10/2/2010	Pressure Tank	Valley		4.39	< .025	107.4811	< .05	0.002	4.15	< .025	< .0002	0.49	< 1	< 5	6.95	1.3
201	10/2/2010	Bathroom Faucet	Valley		1.85	< .025	97.92062	0.072	0.005	3.89	< .025	< .0002	0.64	< 1	< 5	6.96	1.4
202	10/15/2010	Kitchen Sink	Valley	Yes	4.23	< .025	110.5022	< .05	< .001	5.49	< .025	< .0002	0.078	< 1	< 5	6.61	1.17
203	4/19/2009		Valley	Yes		<0.025		<0.025		2.56	<0.025		1.1				
204	4/4/2008		Upland			<0.025		<0.025		1.66	<0.025		0.28				
205	10/19/2010	Kitchen Sink	Valley	Yes	2.25	< .025	97.7825	0.501	< .001	6.1	0.048		6.4	< 1	< 5	7.02	1.9
206	10/19/2010	Kitchen Sink	Valley	Yes	3.71	< .025	148.2114	< .05	0.002	6.34	< .025	< .0002	0.15	< 1	< 5	6.47	3.16
207	10/19/2010	Hand Dug Well	Valley	Yes	5.27	< .025	37.95816	0.223	< .001	1.82	< .025	< .0002	0.5	< 1	< 5	6.13	1.7
208	10/20/2010	Kitchen Sink	Valley	Yes	5.68	< .025	124.7291	< .05	< .001	6.58	< .025	< .0002	0.12	< 1	< 5	7.14	1.13
209	10/26/2010	Kitchen Sink	Valley	Yes	3.92	< .025	127.8105	0.244	< .001	6.54	< .025	< .0002	0.74	< 1	< 5	6.59	1.12
210	10/26/2010	Outside Spigot	Valley		4.68	< .025	146.9524	0.66	< .001	6.58	0.038	< .0002	2.8	< 1	< 5	6.98	1.3
211	7/14/2011	Kitchen Sink	Upland		8.9	< .025							0.11			7.86	
212	8/1/2011	Kitchen Sink	Valley		3.72	< .025							0.7			7.66	
213	10/26/2010		Valley	Yes		< .025	114.8875	0.128	0.004	7.04	< .025	< .0002	0.24	< 1	< 5		1.23
214	10/28/2010	Kitchen Sink	Valley	Yes	4.75	< .025	148.9224	< .05	0.001	9.12	< .025	< .0002	1.8	< 1	< 5	7.16	1.26
215	7/17/2010	Outside Spigot	Valley		3.76	< .025		< .05		5.45	0.25		0.76			7.47	
216	10/28/2010	Basement Pressure Tank	Valley		3.84	< .025	101.5909	< .05	0.01	4.66	0.092	< .0002	9.3	< 1	< 5	7.05	2.09
217	10/28/2010	Pressure Tank	Upland		4.58	< .025	144.4554	< .05	0.002	6.58	< .025	< .0002	0.13	< 1	< 5	7.26	1.45
218	10/28/2010	Bar Sink	Upland		8.57	< .025	68.52078	< .05	0.002	3.36	< .025	< .0002	0.15	< 1	< 5	6.01	1.11
219	10/29/2010	Outside Spigot	Valley	Yes	3.33	< .025	73.23962	< .05	< .001	2.99	< .025	< .0002	12	< 1	< 5	6.95	2.82
220	10/29/2010		Valley			< .021	113.0718	< .05	< .001	4.78	< .025	< .0002	1.5	< 1	< 5		1.56
221	10/29/2010	Outside Sink	Valley		7.74	< .025	84.77674	< .05	0.003	3.73	< .025	< .0002	0.11	< 1	< 5	5.97	1.28
222	10/29/2010	Kitchen Faucet	Upland		3.15	< .025	134.1013	< .05	< .001	7.34	< .025	< .0002	0.12	< 1	< 5	6.67	1.04
223	11/10/2010	Kitchen Sink	Upland		5.21	< .025	108.8348	0.55	0.0094	4.6	< .0025	< .0002	< .1	< 1	< 5	7.01	1.9

Table S.8. Groundwater Quality Data for 1701 "Pre-Drill" Water Well Samples from Susquehanna County, Pennsylvania

Water Well ID	Sample Date	Sampling Port	Topo-graphic Location	Gas Extraction Area (Within 1 km of Active Gas Well)	Dissolved Oxygen - Field (mg/L)	Ethane ug/L	Hardness (mg/L)	Iron (mg/L)	Lead (mg/L)	Magnesium (mg/L)	Manganese (mg/L)	Mercury (mg/L)	Methane ug/L	Nitrate as N (mg/L)	Oil Grease (mg/L)	pH - Field (SU)	Potassium (mg/L)
224	11/10/2010	Kitchen Sink	Valley		4.51	< .025	96.1664	0.17	0.0047	8.8	< .0025	< .0002	< .1	< 1	< 5	6.81	1.1
225	9/28/2010		Upland			0.012	162.6398	1	< .005	13.3	< .025	< .0002	1	< 1			1.6
226	11/11/2010	Outside Spigot	Valley	Yes	5.16	< .025	167.651	0.13	< .0003	11	< .0025	< .0002	0.58	< 1	< 5	7.08	1.51
227	11/11/2010	Kitchen Faucet	Valley	Yes	4.01	0.026	113.593	< .05	< .0003	10	0.012	< .0002	170	< 1	< 5	6.59	1.25
228	9/28/2010	Kitchen Sink	Valley		7.64	< .025	81.3105	< .05	< .005	2.1	< .025	< .0002	< .1	< 1	< 5	7.27	< .001
229	11/17/2010	Kitchen Sink	Valley	Yes	3.7	< .025	140.6482	< .05	0.00037	9.9	0.0026	< .0002	0.19	< 1	< 5	7.31	1.4
230	11/17/2010	Kitchen Sink	Upland		6.98	< .025	174.4232	1.6	0.028	8.4	8.1	< .0002	0.31	< 1	< 5	7.09	1.55
231	11/17/2010	Kitchen Sink	Upland		10.76	< .025	29.8723	< .05	0.0011	2.1	< .0025	< .0005	0.31	< 1	< 5	7.32	0.829
232	12/4/2010	Kitchen Sink	Valley	Yes	4.39	< .025	158.6678	< .05	< .001	9.91	< .025	< .0002	0.13	< 1	< 5	6.72	1.35
233	12/4/2010	Kitchen Sink	Valley	Yes	9.17	< .025	30.7659	0.116	0.001	1.65	< .025	< .0002	0.26	< 1	< 5	6.19	0.969
234	12/4/2010	Basement At Pressure Tank	Valley	Yes	5.8	< .025	132.7633	0.084	0.042	6.53	< .025	< .0002	0.15	< 1	< 5	6.66	1.24
235	12/4/2010	Kitchen Sink	Valley	Yes	4.15	< .025	148.4896	0.226	0.013	9.5	< .025	< .0002	0.21	< 1	< 5	6.95	1.37
236	12/5/2010	Kitchen Sink	Upland	Yes	8.18	< .025	44.48032	< .05	< .001	3.04	< .025	< .0002	0.18	< 1	< 5	5.46	1.68
237	12/5/2010	Kitchen Faucet	Valley	Yes	3.46	< .025	157.669	< .05	< .001	9.91	< .025	< .0002	0.67	< 1	< 5	6.63	1.61
238	12/5/2010	Barn At Ceiling Spigot	Valley	Yes	5.82	8	97.10078	< .05	< .001	5.51	0.074	< .0002	5200	< 1	< 5	7.43	1.93
239	2/17/2009	Kitchen Sink	Valley	Yes	3.78	< .025		< .05		6.37	< .025		8.2		< 5	8.05	
240	12/8/2010	Kitchen Sink	Valley	Yes	4.07	< .025	155.166	< .05	0.00064	11	0.012	< .0002	0.78	< 1	< 5	7.19	1.6
241	12/8/2010	Kitchen Sink	Upland		8.17	< .025	34.4938	< .05	0.0011	1.1	< .0025	< .0002	0.3	< 1	< 5	5.69	< 1
242	12/8/2010	Kitchen Sink	Valley	Yes	9.94	< .025	21.8336	0.063	0.00064	1.3	0.014	< .0002	0.32	< 1	< 5	5.53	< 1
243	12/8/2010	Hand Dug Well	Upland		8.72	< .025	95.5262	< .05	< .0003	4.4	0.0036	< .0002	0.12	< 1	< 5	6.25	11
244	2/17/2009	Kitchen Sink	Valley	Yes	3.14	< .025		0.047		5.56	< .025		1.7		< 5	6.7	
245	4/4/2009	Well Head	Valley	Yes	3.79	< .025		2.46		7.61	0.229		1.5		< 5	7.36	

Table S.8. Groundwater Quality Data for 1701 "Pre-Drill" Water Well Samples from Susquehanna County, Pennsylvania

Water Well ID	Sample Date	Sampling Port	Topo-graphic Location	Gas Extraction Area (Within 1 km of Active Gas Well)	Dissolved Oxygen - Field (mg/L)	Ethane ug/L	Hardness (mg/L)	Iron (mg/L)	Lead (mg/L)	Magnesium (mg/L)	Manganese (mg/L)	Mercury (mg/L)	Methane ug/L	Nitrate as N (mg/L)	Oil Grease (mg/L)	pH - Field (SU)	Potassium (mg/L)
246	12/11/2010	Kitchen Sink	Valley		2.6	< .025	48.626	< .05	< .0003	1.5	0.024	< .0002	0.23	< 1	< 5	6.81	2
247	12/11/2010		Upland			< .025	43.2202	< .05	0.0037	1.4	< .0025	< .0002	0.65	< 1	< 5		< 1
248	12/11/2010	Kitchen Sink	Valley		7.08	< .025	90.5322	< .05	< .0003	4.4	0.0034	< .0002	< .1	< 1	< 5	6.51	1.7
249	3/16/2009	Basement At Pressure Tank	Valley	Yes	8.23	< .025		< .05		3.45	< .025		0.5		< 5	6.73	
250	12/17/2010	Pressure Tank	Upland		7.33	< .025	51.4824	0.27	0.013	2.8	0.0065	0.0002	0.11	< 1	< 5	6.2	0.84
251	12/17/2010	Pressure Tank	Upland		8.48	< .025	55.2148	1.5	0.015	3.1	0.054	< .0002	< .58	< 1	< 5	6.91	1.3
252	12/18/2010	Basement Pressure Tank	Valley	Yes	5.19	0.016	148.1654	0.29	0.0052	9.3	0.19	< .0002	13	< 1	< 5	6.79	1.3
253	6/24/2009	Kitchen Sink	Upland	Yes	1.14	< .025		0.101		8.19	< .025		27		< 5	7.95	
254	12/18/2010	Kitchen Sink	Valley	Yes	1.89	3.2	99.1014	1.1	0.00043	8.3	0.18	< .0002	19000	< 1	< 5	7.14	1.4
255	3/19/2009	Kitchen Sink	Valley	Yes	3.1	< .025		< .05		7.23	< .025		1.1		< 5	7.57	
256	3/19/2009	Kitchen Sink	Upland	Yes	2.63	< .025		< .05		8.04	< .025		7.6		< 5	7.37	
257	12/14/2010	Kitchen Sink	Valley	Yes	4.06	< .025	119.2084	< .05	< .0003	5.3	< .0025	< .0002	0.12	< 1	< 5	6.71	1.3
258	3/4/2009	Kitchen Sink	Upland		7.62	0.026		< .025		6.28	< .025		0.92		< 5	7.5	
259	12/14/2010	Hydrant	Valley	Yes	12.12	< .025	119.594	14	0.15	6	6.4	< .0002	1.3	< 1	< 5	6.85	1.9
260	12/14/2010	Kitchen Sink	Valley	Yes	3.16	0.18	104.6644	< .05	0.00031	4.8	0.0036	< .0002	770	< 1	< 5	7.03	1.5
261	12/14/2010	Bathtub Faucet	Valley	Yes	5.3	< .025	156.48	< .05	< .0003	9.5	0.0087	< .0002	0.2	< 1	< 5	6.99	1.5
262	2/23/2011		Valley	Yes		< .025	102.1412	0.23	0.002	5.4	0.004	< .0002	8.4	< 1	< 5		1.2
263	12/19/2010		Valley	Yes		< .025	149.8388	0.14	< .0003	9.1	0.0025	< .0002	1.5	< 1	< 5		1.5
264	12/19/2010	Kitchen Sink	Valley		4.03	0.11	64.2482	< .05	< .0003	5.9	0.029	< .0002	290	< 1	< 5	7.77	1.8
265	12/19/2010	Kitchen Faucet	Valley		9	< .025	54.3388	< .05	0.00039	4.1	< .0025	< .0002	2.7	< 1	< 5	7.01	1.5

Table S.8. Groundwater Quality Data for 1701 "Pre-Drill" Water Well Samples from Susquehanna County, Pennsylvania

Water Well ID	Sample Date	Sampling Port	Topo-graphic Location	Gas Extraction Area (Within 1 km of Active Gas Well)	Dissolved Oxygen - Field (mg/L)	Ethane ug/L	Hardness (mg/L)	Iron (mg/L)	Lead (mg/L)	Magnesium (mg/L)	Manganese (mg/L)	Mercury (mg/L)	Methane ug/L	Nitrate as N (mg/L)	Oil Grease (mg/L)	pH - Field (SU)	Potassium (mg/L)
266	12/19/2010	School Art Studio Bathroom Sink	Valley	Yes	4.64	0.025	131.641	< .05	0.00036	6.5	0.003	< .0002	2.7	< 1	< 5	6.77	1.3
267	2/26/2009		Upland			<0.025		<0.025		4.68	<0.025		2.8				
268	1/27/2011	Downstairs Kitchen Sink	Valley		4.38	0.14	14.00342	< .05	0.00068	< .5	0.019	< .0002	1100	< 1	< 5	8.74	1.5
269	1/27/2011	Kitchen Sink	Valley		8.23	< .025	88.4994	0.13	0.00027	3.3	< .0025	< .0002	0.34	< 1	< 5	7.14	1.1
270	1/27/2011	Kitchen Sink	Valley		5.98	0.008	80.1848	< .05	0.00012	3.1	< .0025	< .0002	0.23	< 1	< 5	7.54	1
271	3/4/2009	Kitchen Sink	Upland		5.34	< .025		< .025		8.79	< .025		0.46		< 5	7.8	
272	2/11/2011	Kitchen Sink	Upland	Yes	5.52	< .025	180.136	< .05	< .0003	11	< .0025	< .0002	< .1	< 1	< 5	7.14	2.4
273	2/11/2011	Kitchen Sink	Upland		4.37	< .025	239.188	< .05	0.00031	12	< .0025	< .0002	1.1	< 1	< 5	6.83	2.8
274	2/11/2011	Kitchen Sink	Upland		4.62	0.14	34.4676	< .05	0.00033	1.7	0.041	< .0002	440	< 1	< 5	7.95	2.9
275	2/11/2011	Kitchen Sink	Upland	Yes	5.15	< .025	243.175	< .05	0.00088	16	0.08	< .0002	0.82	< 1	< 5	7.15	3.9
276	2/11/2011	In Milkhouse, At Bottom Of Pressure Tank	Upland		10.49	< .025	154.4734	< .05	0.0079	7.8	0.0058	< .0002	0.52	< 1	< 5	7.24	2.3
277	2/4/2011	Pressure Tank	Valley	Yes	8.79	0.068	143.995	1.5	0.0041	9.5	0.021	< .0002	17	< 1	< 5	7.08	1.3
278	2/8/2011	Bathtub	Valley			0.036	146.5968	0.23	0.0012	7.1	0.0031	< .0002	1	< 1	< 5		1.8
279	3/4/2009	Kitchen Sink	Upland		7.05			0.046		5.94	< .025		1.7			7.01	
280	2/8/2011	Kitchen Sink	Upland		9.53	< .025	116.6852	< .05	0.00063	5.9	< .0025	< .0002	1.7	< 1	< 5	6.26	3.2
281	10/19/2010	Kitchen Sink	Valley	Yes	2.72	< .025	120.3405	< .05	< .001	6.06	< .025	< .0002	0.31	< 1	< 5	6.73	1.48
282	2/8/2011	Kitchen Sink, Small Spigot On Right Hand Side Of S	Valley		4.53	< .025	111.7436	< .05	0.0006	4.7	0.0062	< .0002	1.3	< 1	< 5	7.1	1
283	2/11/2011	Kitchen Sink	Upland	Yes		< .025	27.6119	< .05	< .0003	2.4	< .0025	< .0002	0.12	< 1	< 5		1
284	2/11/2011	Kitchen Sink	Upland		7.59	< .025	108.8086	< .05	0.00075	5.2	< .0025	< .0002	0.14	< 1	< 5	7.15	1.7

Table S.8. Groundwater Quality Data for 1701 "Pre-Drill" Water Well Samples from Susquehanna County, Pennsylvania

Water Well ID	Sample Date	Sampling Port	Topo-graphic Location	Gas Extraction Area (Within 1 km of Active Gas Well)	Dissolved Oxygen - Field (mg/L)	Ethane ug/L	Hardness (mg/L)	Iron (mg/L)	Lead (mg/L)	Magnesium (mg/L)	Manganese (mg/L)	Mercury (mg/L)	Methane ug/L	Nitrate as N (mg/L)	Oil Grease (mg/L)	pH - Field (SU)	Potassium (mg/L)
285	2/8/2011	Rental (Adam Vibbard) Kitchen Sink	Upland		7.46	< .025	134.1904	< .05	< .0003	5.3	< .0025	< .0002	0.23	< 1	< 5	6.62	2
286	2/8/2011	Kitchen Sink	Upland		6.77	< .025	78.4852	< .05	0.00077	3.9	< .0025	< .0002	< .1	< 1	< 5	6.85	1.1
287	1/13/2011	Kitchen Sink	Valley	Yes	4.84	0.78		< .05		7.4	0.005		28		< 5	7	
288	2/15/2011	Pressure Tank	Upland		5.78	< .025	100.494	< .05	0.0022	5	0.019	< .0002	0.17	< 1	< 5	7.45	2
289	2/15/2011	Laundry Room Faucet	Upland		5.38	< .025	61.4966	< .05	0.00041	2.2	0.035	< .0002	2.8	< 1	< 5	7.72	2.7
290	3/4/2009	Kitchen Sink	Valley		2.38	< .025		0.05		9.29	0.519		1.8		< 5	7.27	
291	2/14/2011	Kitchen Sink	Upland		6.6	< .025	135.3472	< .05	< .0003	7.4	< .0025	< .0002	0.62	< 1	< 5	7.39	2.3
292	2/15/2011	Kitchen Faucet	Valley		5.89	< .025	159.0294	< .05	< .0003	8.3	0.0076	< .0002	0.15	< 1	< 5	7.09	1.8
293	2/14/2011	Barn Milkhouse Sink	Valley		4.23	< .025	119.901	< .05	< .0003	8.5	0.003	< .0002	0.32	< 1	< 5	7.12	< 1
294	2/15/2011	Milkhouse Faucet	Upland	Yes	9.19	< .025	88.4732	< .05	< .0003	3.9	< .0025	< .0002	0.41	< 1	< 5	7.02	2.7
295	2/15/2011	1St Floor Kitchen Faucet	Upland	Yes	4.68	< .025	134.9092	< .05	< .0003	7.9	0.0042	< .0002	0.22	< 1	< 5	7.26	1.6
296	2/17/2011	Pressure Tank In Basement	Upland	Yes	8.98	< .025	199.236	0.086	< .0003	12	0.003	< .0002	0.24	< 1	< 5	7.04	1.9
297	3/4/2009	1St Floor Mens Room Sink	Valley		4.02	< .025		0.081		5.74	< .025		0.34		< 5	7.68	
298	3/21/2011		Valley			< 26	130	0.061	< .001	7.4	0.052	< .0002	20900	< .05	< 4.7		6.7
299	3/21/2011		Upland	Yes		< 26	134	0.087	< .001	9.9	0.0011	< .0002	< 26	1.4	< 4.7		1.2
300	3/22/2011		Valley	Yes		< 26	104	0.16	< .001	8.2	0.25	< .0002	185	< .05	< 4.9		0.92
301	3/4/2009	Kitchen Sink	Valley		3.34			0.079		5.39	< .025		1.1			7.63	
302	12/10/2010	Garage Faucet	Upland	Yes	3.11	< .025	148.551	< .22	< .0003	10	< .0025	< .0002	3.7	< 1	< 5	6.98	1.2
303	12/10/2010	Kitchen Faucet	Upland	Yes	4.07	< .025	116.7114	< .05	< .0003	5.3	< .0025	< .0002	< .1	< 1	< 5	7.3	1.2

Table S.8. Groundwater Quality Data for 1701 "Pre-Drill" Water Well Samples from Susquehanna County, Pennsylvania

Water Well ID	Sample Date	Sampling Port	Topo-graphic Location	Gas Extraction Area (Within 1 km of Active Gas Well)	Dissolved Oxygen - Field (mg/L)	Ethane ug/L	Hardness (mg/L)	Iron (mg/L)	Lead (mg/L)	Magnesium (mg/L)	Manganese (mg/L)	Mercury (mg/L)	Methane ug/L	Nitrate as N (mg/L)	Oil Grease (mg/L)	pH - Field (SU)	Potassium (mg/L)
304	12/10/2010	Kitchen Sink	Valley	Yes	5.37	< .025	124.15	< .05	< .0003	6.5	< .0025	< .0002	< .1	< 1	< 5	6.87	1.2
305	12/10/2010		Upland	Yes	4.25	< .025	128.2418	0.45	0.031	8.1	0.54	< .0002	3.2	< 1	< 5	7	< 1
306	12/10/2010	Pressure Tank	Upland		6.82	< .025	127.0326	0.07	0.0017	7.2	0.027	< .0002	2.3	< 1	< 5	6.77	1.3
307	12/10/2010	Kitchen Faucet	Valley		7.57	< .025	37.4026	< .05	< .0003	1.2	< .0025	< .0002	< .1	< 1	< 5	6.36	1.8
308	12/14/2010	Kitchen Sink	Valley	Yes	4.3	< .025	126.6732	< .05	< .0003	5.9	< .0025	< .0002	0.58	< 1	< 5	7.09	< 1.2
309	3/12/2009	Kitchen Sink Faucet	Upland		5.95	< .025		< .05		3.84	< .025		< .1		< 5	7.41	
310	12/20/2010	Kitchen Sink	Upland		4.29	< .025	146.1588	< .05	< .0003	7.6	0.013	< .0002	< .1	< 1	< 5	6.99	2
311	12/20/2010	Pressure Tank	Valley		7.35	< .025	94.6764	1.8	0.0013	4.8	0.13	< .0002	7.3	< 1	< 5	7.49	1.9
312	12/20/2010	Basement At Pressure Tank	Valley		7.95	< .025	143.6618	< .05	0.026	7.6	0.11	< .0002	1.3	< 1	< 5	7.02	1.3
313	12/20/2010	Kitchen Sink	Upland		9.37	< .025	85.5644	0.14	0.0011	3.8	0.0061	< .0002	0.11	< 1	< 5	7.14	1.2
314	12/20/2010	Kitchen Sink	Upland		6.09	< .025	81.832	< .05	0.00037	3.5	< .0025	< .0002	0.84	< 1	< 5	6.77	1.2
315	3/4/2009	Pressure Tank In Basement	Upland		6.38	< .025		0.058		8.94	< .025		0.98		< 5	7.58	
316	12/20/2010	Bathtub Faucet	Upland		6.18	< .025	77.6878	< .05	< .0003	3.1	< .0025	< .0002	0.14	< 1	< 5	6.75	1.5
317	4/15/2009	Horse Barn Faucet	Valley	Yes	3.31	< .025		< .05		5.17	< .025		0.55		< 5	7.39	
318	1/6/2011	Kitchen Sink	Upland	Yes	9.5	0.76		< .05	0.0016	4.4	0.042	< .0002	2000	< 1	< 5	7.14	1.6
319	4/19/2009	Kitchen Sink Faucet	Valley	Yes	6.6	0.036		0.051		6.85	< .025		5.5		< 5	7.13	
320	4/8/2009	Barn After Filter	Valley		8.21	< .025		0.545		5.88	< .025		0.16		< 5	6.66	
321	4/12/2011	Cwt Of Kitchen Sink	Valley		8.54	< 26	112	0.17	< .001	5.1	0.0013	< .0002	< 26	2.8	< 4.7	7.57	0.9
322	4/13/2011	Well	Upland	Yes		< 26	89	1	0.0014	4.9	0.23	< .0002	< 26	< .05	< 5.2		1.4
323	4/13/2011	Kitchen Sink	Valley	Yes	5.98	< 26	75.6	< .05	< .001	2.1	< .0005	< .0002	< 26	0.34	< 4.6	7.39	0.75
324	4/13/2011	Kitchen Sink Cwt	Upland		2.67	< 26	118	0.33	< .001	4.9	0.54	< .0002	< 26	0.33	< 4.6	7.71	1.2

Table S.8. Groundwater Quality Data for 1701 "Pre-Drill" Water Well Samples from Susquehanna County, Pennsylvania

Water Well ID	Sample Date	Sampling Port	Topo-graphic Location	Gas Extraction Area (Within 1 km of Active Gas Well)	Dissolved Oxygen - Field (mg/L)	Ethane ug/L	Hardness (mg/L)	Iron (mg/L)	Lead (mg/L)	Magnesium (mg/L)	Manganese (mg/L)	Mercury (mg/L)	Methane ug/L	Nitrate as N (mg/L)	Oil Grease (mg/L)	pH - Field (SU)	Potassium (mg/L)
325	4/13/2011	Kitchen Sink Cwt	Upland		5.28	< 26	82.5	< .05	< .001	3.1	0.0047	< .0002	< 26	0.87	< 4.7	6.97	1.6
326	3/12/2009	Basement Before Filter	Upland	Yes	5.83	< .025		< .05		5.55	< .025		< .1		< 5	7.78	
327	4/14/2011	Farmhouse Kitchen Sink Cwt	Valley		6.55	< 26	135	0.1	< .001	5.4	0.025	< .0002	< 26	2.2	< 4.6	7.75	1.7
328	4/15/2011	Kitchen Sink Cwt	Upland		9.55	< 26	129	0.83	< .001	5	0.0071	< .0002	< 26	1.4	< 4.7	7.26	1.3
329	4/18/2011	Pressure Tank In Basement	Valley		8.2	< 26	72.3	< .05	0.0014	2.9	0.0012	< .0002	< 26	3.8	< 4.7	6.26	5
330	4/18/2011	Kitchen Sink	Upland		5.48	< 26	191	< .05	< .001	7.7	0.0028	< .0002	< 26	3.4	< 4.6	7.44	1.2
331	4/19/2011	Kitchen Faucet	Upland		5.33	< 26	128	< .05	< .001	5.6	0.013	< .0002	< 26	0.13	< 4.6	7.65	1
332	4/22/2011	Mary Steele Kitchen Sink	Valley	Yes	10.37	< 26	84.6	0.079	< .001	3.8	0.0011	< .0002	< 26	4.1	< 4.6	6.99	0.79
333	4/26/2011	Kicthen Sink	Valley	Yes	4.59	< 26	116	< .05	< .001	6.8	0.00072	< .0002	< 26	0.99	< 4.6	7.26	1.1
334	4/27/2011	Well 2	Upland	Yes	4.08	< 26	130	< .05	< .001	6.8	0.002	< .0002	< 26	0.7	< 4.6	7.71	1.2
335	4/28/2011	Basement Pressure Tank	Valley	Yes	0.52	< 26	116	1.6	< .001	7.7	0.95	< .0002	< 26	0.4	< 4.7	7.42	1
336	4/28/2011	Pressure Tank In Basement On E Side Of House	Valley	Yes	3	< 26	134	0.16	< .001	7.8	0.031	< .0002	< 26	0.78	< 4.6	7.58	1
337	5/2/2011		Upland	Yes		<26	111	<0.05	<0.001	3.7	0.045	<0.0002	<26	0.25	<4.6		1.7
338	5/2/2011		Upland	Yes	6.27	<26	46.6	<0.05	0.0019	2.2	0.0031	<0.0002	<26		<4.7	5.91	1.6
339	5/3/2011	Kitchen Sink	Valley	Yes	5.39	< 26	137	0.14	< .001	8.3	0.0007	< .0002	< 26	1.1	< 4.6	7.37	1.3
340	5/4/2011	Kitchen Cwt	Valley	Yes	3.84	< 26	98.6	0.055	< .001	5.1	0.0007	< .0002	< 26	2.9	< 4.6	7.53	0.95
341	5/4/2011	Kitchen Sink	Valley	Yes	3.18	< 26	101	< .05	< .001	5.1	< .0005	< .0002	< 26	2.3	< 4.6	7.38	0.99
342	5/4/2011	Outside Hose Bib On The East Side Of Home	Valley	Yes	0.23	< 26	160	0.64	< .001	11.4	0.042	< .0002	< 26	0.45	< 4.6	7.22	1.1
343	5/4/2011	Outdoor Spigot	Valley	Yes	1.57	< 26	117	0.088	< .001	6.7	0.00076	< .0002	< 26	0.89	< 4.6	7.22	1.1
344	2/26/2009	Kitchen Sink	Upland		3.62	< .025		< .05		5.52	< .025		0.44		< 5	7.16	

Table S.8. Groundwater Quality Data for 1701 "Pre-Drill" Water Well Samples from Susquehanna County, Pennsylvania

Water Well ID	Sample Date	Sampling Port	Topo-graphic Location	Gas Extraction Area (Within 1 km of Active Gas Well)	Dissolved Oxygen - Field (mg/L)	Ethane ug/L	Hardness (mg/L)	Iron (mg/L)	Lead (mg/L)	Magnesium (mg/L)	Manganese (mg/L)	Mercury (mg/L)	Methane ug/L	Nitrate as N (mg/L)	Oil Grease (mg/L)	pH - Field (SU)	Potassium (mg/L)
345	5/4/2011	School Art Studio Bathroom Sink	Valley	Yes	4.78	< 26	125	0.33	< .001	6.7	0.0046	< .0002	< 26	1.2	< 4.6	7.31	1.2
346	5/4/2011	Pressure Tank	Valley	Yes	5.17	< 26	119	3.1	0.001	6.4	0.013	< .0002	< 26	1.1	< 4.6	7.64	1.1
347	5/5/2011	Kitchen Sink Cwt	Upland	Yes	7.55	< 26	145	0.13	< .001	11.2	0.51	< .0002	< 26	< .05	< 4.6	7.37	1
348	5/5/2011	Kitchen Sink	Valley	Yes	71.3	< 26	140	0.27	< .001	9.6	0.005	< .0002	< 26	0.083	< 4.7	7.59	0.94
349	5/5/2011	Pressure Tank	Valley	Yes	1.45	< 26	128	0.73	0.0017	7.8	0.37	< .0002	< 26	0.65	< 5	7.05	0.93
350	5/5/2011	Kitchen Sink Cwt	Valley	Yes	6.2	< 26	110	< .05	< .001	6	0.049	< .0002	< 26	0.94	< 4.7	7.27	0.75
351	5/5/2011	Pressure Tank	Valley	Yes	0.52	< 26	155	1	0.005	11.3	0.0058	< .0002	< 26	0.062	< 4.6	7.75	1.2
352	5/6/2011	Pressure Tank	Valley	Yes	0.74	< 26	170	0.14	< .001	11.6	0.46	< .0002	< 26	0.081	< 4.7	7.42	1.1
353	5/6/2011	Kitchen Sink	Valley	Yes	2.86	< 26	115	< .05	< .001	6.3	< .0005	< .0002	< 26	0.99	< 4.6	7.76	1.5
354	5/7/2011	Pressure Tank In Basement	Valley	Yes	0.07	< 26	120	2.3	< .001	8.1	0.01	< .0002	< 26	0.099	< 4.8	7.65	1.1
355	5/7/2011	Bathroom Sink Ctw	Valley	Yes	0.77	< 26	127	< .05	< .001	8.6	0.0015	< .0002	< 26	0.063	< 5	7.69	0.99
356	5/7/2011	Backroom Sink	Valley	Yes	4.54	< 26	116	0.27	< .001	7.1	0.009	< .0002	< 26	0.077	< 4.7	7.58	0.91
357	5/7/2011	Pressure Tank In Basement	Upland	Yes	0.37	< 26	125	0.11	0.001	8.3	0.01	< .0002	< 26	0.052	< 4.6	7.67	0.94
358	5/7/2011	Kitchen Sink	Valley	Yes	3.69	< 26	138	0.25	0.001	4	0.028	< .0002	< 26	9.6	< 4.7	6.41	3.1
359	5/7/2011	Pressure Tank	Valley	Yes	6.6	< 26	137	< .05	< .001	7.4	< .0005	< .0002	< 26	4.7	< 4.7	6.62	1.2
360	5/9/2011	Pressure Tank	Valley	Yes	2.25	< 26	115	0.45	0.0014	7	0.028	< .0002	< 26	0.85	< 4.6	7.1	1.1
361	5/9/2011	Basement Pressure Tank	Valley	Yes	1.04	< 26	133	2.8	< .001	7.3	0.1	< .0002	< 26	0.81	< 4.6	7.21	1.2
362	5/9/2011	Well	Valley	Yes		< 26	60.8	< .05	0.011	3.5	0.0018	< .0002	< 26	2.4	< 4.7		1.5
363	5/9/2011	Kitchen Sink	Valley	Yes	4.8	< 26	135	0.059	< .001	8.5	0.014	< .0002	< 26	0.42	< 4.8	7.57	1.1

Table S.8. Groundwater Quality Data for 1701 "Pre-Drill" Water Well Samples from Susquehanna County, Pennsylvania

Water Well ID	Sample Date	Sampling Port	Topo-graphic Location	Gas Extraction Area (Within 1 km of Active Gas Well)	Dissolved Oxygen - Field (mg/L)	Ethane ug/L	Hardness (mg/L)	Iron (mg/L)	Lead (mg/L)	Magnesium (mg/L)	Manganese (mg/L)	Mercury (mg/L)	Methane ug/L	Nitrate as N (mg/L)	Oil Grease (mg/L)	pH - Field (SU)	Potassium (mg/L)
364	5/9/2011	Outdoor Hose Bib On South Side Of Gas Station	Valley	Yes	157	< 26	122	0.34	< .001	8.2	0.18	< .0002	< 26	< .05	< 4.7	7.43	0.98
365	5/9/2011	Outside Spigot	Valley	Yes	3.34	< 26	136	0.1	< .001	8.6	< .0005	< .0002	< 26	0.43	< 4.6	7.52	1.2
366	5/9/2011	Pressure Tank	Upland	Yes		< 26	90.2	2.3	0.034	5.3	0.022	< .0002	< 26	1.4	< 4.7		1
367	5/9/2011	Pressure Tank	Valley	Yes	7.31	< 26	93.4	< .05	0.0016	5.3	0.013	< .0002	< 26	3.6	< 4.7	6.2	1.6
368	5/9/2011	Outside Faucet	Valley	Yes	0.57	< 26	119	0.58	< .001	7.8	0.17	< .0002	< 26	< .05	< 4.8	7.43	0.94
369	5/10/2011	Kitchen Sink	Upland	Yes	5.35	< 26	27	0.19	0.0036	1.1	0.017	< .0002	< 26	0.18	< 4.6	6.6	0.65
370	5/10/2011	Inside Faucet	Valley	Yes	3.9	< 26	84.9	0.25	0.0013	4.4	0.0029	< .0002	< 26	1.5	< 4.6	6.47	1
371	5/10/2011	Inside Faucet	Valley	Yes	2.55	< 26	124	0.2	< .001	8.3	0.018	< .0002	< 26	0.35	< 4.6	7.56	0.9
372	5/11/2011		Valley	Yes		< 26	38.3	0.3	< .001	2.1	0.069	< .0002	< 26	0.36	< 4.7		1.9
373	5/11/2011	Pressure Tank In Basement	Valley	Yes	1.99	< 26	152	0.58	< .001	10.6	0.0098	< .0002	< 26	0.84	< 4.8	7.25	1.4
374	5/11/2011		Valley	Yes		< 26	84.4	0.21	0.0023	5.1	0.0062	< .0002	< 26	2.6	< 4.6		1.6
375	5/11/2011	Kitchen Sink	Valley	Yes	16.8	< 26	55.3	0.097	0.0038	3.5	0.0026	< .0002	< 26	2.5	< 4.7	6.09	1
376	5/11/2011	Outside Bib	Valley	Yes	4.71	< 26	134	0.077	< .001	9.5	0.0026	< .0002	< 26	1.1	< 4.8	7.59	1.2
377	5/11/2011	Kitchen Sink	Valley	Yes	4.75	< 26	107	0.14	0.0082	5.3	0.0013	< .0002	< 26	1.5	< 4.6	6.85	1.1
378	5/11/2011	Pressure Tank In Basement	Valley	Yes	17.2	< 26	106	0.22	< .001	6.1	0.016	< .0002	< 26	2.2	< 4.8	6.58	1.1
379	5/12/2011		Valley	Yes		< 26	117	0.16	< .001	7.4	0.0017	< .0002	< 26	0.97	< 4.7		1.1

Table S.8. Groundwater Quality Data for 1701 "Pre-Drill" Water Well Samples from Susquehanna County, Pennsylvania

Water Well ID	Sample Date	Sampling Port	Topo-graphic Location	Gas Extraction Area (Within 1 km of Active Gas Well)	Dissolved Oxygen - Field (mg/L)	Ethane ug/L	Hardness (mg/L)	Iron (mg/L)	Lead (mg/L)	Magnesium (mg/L)	Manganese (mg/L)	Mercury (mg/L)	Methane ug/L	Nitrate as N (mg/L)	Oil Grease (mg/L)	pH - Field (SU)	Potassium (mg/L)
380	5/12/2011	Outside Faucet	Valley	Yes	4.1	< 26	67.4	< .05	0.0021	4.2	0.0047	< .0002	< 26	3.4	< 4.6	5.87	1.1
381	5/12/2011	Basement At Pressure Tank	Valley	Yes	3.33	< 26	105	< .05	0.0025	5.7	0.0015	< .0002	< 26	0.81	< 4.8	7.18	0.86
382	5/12/2011	Pressure Tank	Valley	Yes	2.19	< 26	209	1.3	0.0039	11.4	0.46	< .0002	< 26	0.45	< 4.7	6.16	1.4
383	5/12/2011	Kitchen Sink	Valley	Yes	52.8	< 26	116	0.37	< .001	7.3	0.002	< .0002	< 26	2	< 4.6	6.72	1.1
384	5/13/2011	Pressure Tank Rental Property	Valley	Yes	1.42	< 26	132	< .05	0.0016	9.6	< .0005	< .0002	< 26	2.5	< 4.7	6.57	0.88
385	5/13/2011	Pressure Tank	Valley	Yes	0.011	< 26	139	< .05	< .001	10.1	0.0081	< .0002	< 26	0.081	< 4.7	7.75	1.2
386	5/13/2011	Kitchen Sink	Valley	Yes	5.6	< 26	78.9	0.093	0.0031	5	0.0063	< .0002	< 26	2.9	< 4.7	6.22	1.3
387	5/13/2011	Outside Faucet	Valley	Yes	0.08	< 26	159	0.45	< .001	11.2	0.052	< .0002	< 26	< .05	< 4.7	7.51	1.3
388	1/31/2011	Kitchen Sink	Valley		4.3	< .025	121.6792	< .05	< .0003	5.9	< .0025	< .0002	0.3	< 1	< 5	7.02	1
389	1/8/2011	Kitchen Sink	Upland	Yes	5.72	< .025	113.4432	0.1	< .0003	3.9	< .0025	< .0002	< .1	< 1	< 5	7.16	1.4
390	1/8/2011	Kitchen Sink	Valley		8.24	< .025	41.9586	< .05	0.00068	1.7	< .0025	< .0002	0.13	< 1	< 5	6.73	< 1
391	1/4/2011	Kitchen Sink	Valley	Yes		< .025	60.5158	< .05	0.00071	5.6	< .0025	< .0002	< .1	< 1	< 5		1.1
392	2/17/2011	Outside Spigot	Upland		11.77	< .025	9.3617	< .05	0.00075	< .5	< .0025	< .0002	40	< 1	< 5	8.26	1.6
393	2/22/2011	Kitchen Sink	Upland	Yes	13.56	< .025	149.4532	< .05	0.00041	8.4	0.0025	< .0002	< .1	< 1	< 5	6.87	2.8
394	2/22/2011	Pressure Tank In Barn, Old Milkhouse	Upland		9.23	< .025	178.384	0.053	0.0013	13	0.0025	< .0002	< .1	< 1	< 5	6.67	1.6
395	2/22/2011	Rental Kitchen Sink	Upland		7.31	< .025	194.242	0.11	0.00041	12	0.0025	< .0002	0.16	< 1	< 5	6.91	1.8
396	1/8/2011	Basement At Pressure Tank	Valley	Yes	8.59	< .025	84.6098	0.21	0.013	6.6	0.0048	< .0002	< .1	< 1	< 5	6.6	1.3
397	1/31/2011	Kitchen Sink	Valley		9.31	< .025	31.9706	0.72	0.0019	1.7	0.011	< .0002	1.2	< 1	< 5	6.36	< 1

Table S.8. Groundwater Quality Data for 1701 "Pre-Drill" Water Well Samples from Susquehanna County, Pennsylvania

Water Well ID	Sample Date	Sampling Port	Topo-graphic Location	Gas Extraction Area (Within 1 km of Active Gas Well)	Dissolved Oxygen - Field (mg/L)	Ethane ug/L	Hardness (mg/L)	Iron (mg/L)	Lead (mg/L)	Magnesium (mg/L)	Manganese (mg/L)	Mercury (mg/L)	Methane ug/L	Nitrate as N (mg/L)	Oil Grease (mg/L)	pH - Field (SU)	Potassium (mg/L)
398	1/9/2011	Hand Dug Well West Of House	Upland	Yes	9.77	< .025	28.65	< .05	< .0003	1.5	0.0056	< .0002	1.1	< 1	< 5	5.42	< 1
399	1/4/2011	Pressure Tank	Upland		8.76	< .025	117.9468	< .05	0.0014	5.6	< .0025	< .0002	< .05	< 1	< 5	7.8	1.2
400	3/12/2009	Kitchen Sink	Valley		2.86	< .025		< .05		7.86	0.03		1.1		< 5	7.93	
401	1/31/2011	Kitchen Sink	Upland	Yes	2.64	< .025	150.3816	< .05	0.00059	6.2	0.93	< .0002	220	< 1	< 5	6.61	1.1
402	2/22/2011	Upstairs Kitchen Sink	Upland		4.4	< .025	119.9534	0.078	< .0003	7.3	0.0079	< .0002	2.2	< 1	< 5	6.53	< 1
403	1/31/2011	Kitchen Sink	Upland	Yes	9.45	< .025	97.8922	< .05	< .0003	7.4	< .0025	< .0002	0.46	< 1	< 5	7.11	1.2
404	1/31/2011	Kitchen Sink	Upland		4.35	< .025	27.5898	< .05	< .0003	1	0.0093	< .0002	170	< 1	< 5	8.18	2.2
405	1/4/2011	Hand Dug Well	Upland		8.13	< .025	20.85052	< .05	0.00034	0.94	0.0037	< .0002	0.68	< 1	< 5	6.57	0.14
406	2/22/2011	Basement Of Rental At Pressure Tank	Upland		9.26	< .025	179.26	0.57	0.0039	12	0.25	< .0002	0.58	< 1	< 5	7.03	1.3
407	1/8/2011	Basement At Pressure Tank	Upland		8.35	< .025	76.4262	< .05	0.0006	3.4	< .0025	< .0002	0.46	< 1	< 5	6.66	< 1
408	1/8/2011	Kithcen Sink	Upland		6.13	< .025	82.6556	< .05	< .0003	3.7	< .0025	< .0002	< .1	< 1	< 5	6.96	< 1
409	1/14/2011	Laundry Room Sink	Upland		7.18	< .025	114.6262	< .05	< .0003	5.4	< .0025	< .0002	0.16	< 1	< 5	7.36	1
410	3/12/2009	Kitchen Sink	Valley		3.36	< .025		< .05		8.49	0.026		0.6		5	7.9	
411	1/31/2011	Kitchen Sink	Valley		7.6	< .025	57.3262	< .05	0.00059	2.4	0.0033	< .0002	16	< 1	< 5	6.68	1.4
412	2/22/2011	Breakroom Sink- Lower Level	Upland		2.73	0.048	172.645	< .05	0.0003	11	0.0087	< .0002	1.5	< 1	< 5	6.96	3.2
413	3/16/2009	Kitchen Sink	Valley		2.59	< .025		< .05		9.93	< .025		2.3		< 5	7.62	
414	1/8/2011	Kitchen Sink	Valley	Yes	3.44	< .025	121.215	0.66	< .0003	7	0.052	< .0002	2.7	< 1	< 5	6.63	< 1
415	1/4/2011	Ktichen Sink	Upland		8.7	< .025	48.5998	0.052	0.0054	2.1	< .0025	< .0002	0.83	< 1	< 5	6.5	1.2
416	1/31/2011	Kitchen Sink	Valley	Yes	10.01	< .025	86.3094	< .05	< .0003	5.8	0.011	< .0002	2.5	< 1	< 5	6.61	1.3
417	1/10/2011	Hand Dug Well	Upland		6.39	< .025	42.8084	0.1	< .0003	1.3	0.0053	< .0002	0.84	< 1	< 5	5.96	< 1
418	2/22/2011	Kitchen Sink	Valley		6.23	< .025	82.2438	0.15	0.00079	3.6	0.013	< .0002	1.8	< 1	< 5	6.74	2

Table S.8. Groundwater Quality Data for 1701 "Pre-Drill" Water Well Samples from Susquehanna County, Pennsylvania

Water Well ID	Sample Date	Sampling Port	Topo-graphic Location	Gas Extraction Area (Within 1 km of Active Gas Well)	Dissolved Oxygen - Field (mg/L)	Ethane ug/L	Hardness (mg/L)	Iron (mg/L)	Lead (mg/L)	Magnesium (mg/L)	Manganese (mg/L)	Mercury (mg/L)	Methane ug/L	Nitrate as N (mg/L)	Oil Grease (mg/L)	pH - Field (SU)	Potassium (mg/L)
419	1/10/2011	Kitchen Sink	Valley		3.64	< .025	172.3118	< .05	0.0041	9.1	0.038	< .0002	1.2	< 1	< 5	6.93	2
420	1/4/2011		Upland			< .025	71.0204	< .05	< .0003	3.3	< .0025	< .0002	0.12	< 1	< 5		1.4
421	2/17/2011	Kitchen Faucet On 2Nd Floor	Upland		8.911	< .025	80.5704	< .05	< .0003	3.8	< .0025	< .0002	< .1	< 1	< 5	6.99	1.1
422	1/4/2011		Upland	Yes		0.056	105.3046	0.13	< .0003	9.2	< .0025	< .0002	49	< 1	< 5		2.1
423	1/7/2011	Milkhouse Faucet	Valley	Yes	5.43	< .025	134.314	< .05	< .0003	12	< .0025	< .0002	0.13	< 1	< 5	7.07	2
424	1/7/2011	Kitchen Sink Rental	Upland	Yes	3.72	< .025	119.901	0.27	0.002	8.5	0.13	< .0002	0.61	< 1	< 5	7.45	1.3
425	10/8/2009		Upland					0.548		5.59	< .025		< 1				
426	3/13/2009	Kitchen Sink	Valley		1.71	6.1		< .05		3.92	0.059		15000		< 5	8.26	
427	3/12/2009	Kitchen Sink	Upland		2.55	< .025		< .05		2.03	< .025		2.4			8.71	
428	6/24/2009		Upland	Yes		< .025		< .025		1.16	< .025		0.24				
429	3/4/2009		Upland			<0.025		0.046		5.94	<0.025		1.7				
430	6/13/2011	Barn Spigot	Upland		10.11	< .025							0.14			6.52	
431	6/14/2011	Kitchen Sink Overlooking Road	Upland		4.34	< .025							0.12			7.12	
432	6/14/2011	Pressure Tank	Valley		4.61	0.093							0.45			6.41	
433	6/16/2011	Kitchen Sink On Island In Kitchen	Valley		3.54	< .025							0.16			7.25	
434	6/16/2011	Kitchen Sink	Valley		17.95	< .025							0.13			7.24	
435	6/16/2011	In Barn Milkhouse- At Sink	Valley		7.82	< .025							6.1			7.4	
436	6/16/2011	Outside Spigot- Next To Porch At End Of Driveway	Upland		14.22	< .025							0.16			7.3	
437	6/16/2011	Kitchen Sink On Lake Road Side Of House Under Window	Valley		4.94	< .025							0.28			7.12	
438	6/17/2011	Kitchen Sink	Valley		3.87	0.29							1200			6.8	

Table S.8. Groundwater Quality Data for 1701 "Pre-Drill" Water Well Samples from Susquehanna County, Pennsylvania

Water Well ID	Sample Date	Sampling Port	Topo-graphic Location	Gas Extraction Area (Within 1 km of Active Gas Well)	Dissolved Oxygen - Field (mg/L)	Ethane ug/L	Hardness (mg/L)	Iron (mg/L)	Lead (mg/L)	Magnesium (mg/L)	Manganese (mg/L)	Mercury (mg/L)	Methane ug/L	Nitrate as N (mg/L)	Oil Grease (mg/L)	pH - Field (SU)	Potassium (mg/L)
439	6/23/2011	Kitchen Sink	Valley			<0.025							0.34				
440	6/23/2011	Pressure Tank	Upland		4.47	< .025							0.16			6.69	
441	6/23/2011	Pressure Tank	Valley		3.3	< .025							0.1			6.58	
442	6/24/2011		Valley			< .025							24				
443	6/24/2011	Kitchen Sink	Upland		3.98	< .025							0.48			7.27	
444	6/24/2011	Kitchen Sink	Valley		3.15	< .025							12			7.65	
445	6/24/2011	Kitchen Sink	Upland		2.71	0.08							290			7.76	
446	6/24/2011	Shed Spigot	Valley		6.56	< .025							36			7.37	
447	6/13/2011	Kitchen Faucet	Upland			< .025							0.81				
448	6/15/2011	Kitchen Sink Near Rear Of House	Upland		8.83	< .025							1.1			6.3	
449	6/15/2011	Kitchen Sink On South Side Of House	Upland		8.18	< .025							0.33			6.58	
450	6/15/2011	Kitchen Sink On Wall Closest To Red Barn Behind House	Upland		6.32	< .025							0.14			6.85	
451	6/15/2011	Kitchen Sink	Valley		9.33	< .025							< .1			6.29	
452	6/15/2011	Outside Spigot- On The North Side Of The Residence	Upland		16.21	< .025							0.15			6.52	
453	6/15/2011		Upland			< .025							0.23				
454	3/25/2009	Kitchen Sink	Upland		8.66	< .025		< .05		7.58	< .025		0.2		< 5	7.91	
455	6/27/2011	Greenhouse Sink	Valley		7.5	< .025							0.13			7.2	
456	4/15/2009	Kitchen Sink	Valley		7.23	< .025		0.119		3.74	< .025		0.43		< 5	6.64	
457	4/19/2009	Basement Pressure Tank	Valley		7.55	< .025		< .025		4.18	< .025		4.1		< 5	7.13	
458	6/13/2011	Outside Hose Bib	Valley	Yes	7.81	< 26	70	0.054	< .001	3.5	0.0012	< .0002	< 26	0.83		6.38	1.1

Table S.8. Groundwater Quality Data for 1701 "Pre-Drill" Water Well Samples from Susquehanna County, Pennsylvania

Water Well ID	Sample Date	Sampling Port	Topo-graphic Location	Gas Extraction Area (Within 1 km of Active Gas Well)	Dissolved Oxygen - Field (mg/L)	Ethane ug/L	Hardness (mg/L)	Iron (mg/L)	Lead (mg/L)	Magnesium (mg/L)	Manganese (mg/L)	Mercury (mg/L)	Methane ug/L	Nitrate as N (mg/L)	Oil Grease (mg/L)	pH - Field (SU)	Potassium (mg/L)
459	6/13/2011	Outside Cold Water Hose Bib	Upland	Yes	0.14	< 26	150	0.059	< .001	9.4	0.015	< .0002	46.7	< .05		7.56	1.1
460	6/16/2011	Bailed Directly From Well	Upland		1.85	< 26	34	0.39	< .001	1.7	0.0079	< .0002	< 26	1.4	< 4.8	5.89	1
461	2/26/2009	Mary Steele Kitchen Sink	Valley		9.81	< .025		< .025		3.62	< .025		1.6		< 5	6.85	
462	6/16/2011		Upland			<26	77	<0.05	<0.001	5.7	0.00092	<0.0002	<26	2.1	<4.7		1.2
463	6/20/2011	Kitchen Sink Cwt On Nw Side Of House	Upland		13.3	< 26	71	< .05	0.0012	4.5	0.001	< .0002	< 26	3.7		7.54	0.82
464	6/20/2011	Well	Upland		9.3	< 26	51	0.18	0.0012	3.4	0.0074	< .0002	< 26	2.6		6.85	0.92
465	1/25/2009		Valley					< .025		3.5	< .01		9				
466	6/20/2011		Upland	Yes		< 26	110	< .05	< .001	7.1	0.0094	< .0002	< 26	< .05			1.9
467	6/21/2011	At Pressure Tank In Basement	Upland	Yes	3.64	< 26	61	< .05	0.0013	2.6	< .0005	< .0002	< 26	0.7		6.22	0.67
468	6/22/2011	Inside Hose Bib Located In The Garage	Valley		5.76	<26	72	<4.8	0.0017	2.8	0.0055	<0.0002	<26	1.1		7.35	0.66
469	4/1/2009		Valley			< .025		< .025		5.3	< .025		< .1		< 5		
470	6/22/2011	Outside Hose	Valley		0.81	< 26	90	0.13	< .001	5.4	0.01	< .0002	< 26	0.13		7.82	1.3
471	6/22/2011	Well	Valley	Yes	0.12	< 26	150	0.17	< .001	10	0.12	< .0002	< 26	< .05		8.55	1.4
472	6/22/2011	Kitchen Sink Cwt	Valley		2.43	< 26	75	0.2	< .001	5.7	0.0024	< .0002	< 26	< .05		7.87	0.97
473	6/23/2011	Pipe In Basement	Upland		3.82	< 26	21	1.1	< .001	0.98	0.46	< .0002	< 26	< .05		8.7	2.6
474	4/1/2009		Valley			< .025		< .025		6.08	< .025		0.25		< 5		
475	6/23/2011	Outside Spigot	Valley		0.19	< 26	94	< .05	< .001	4.8	< .0005	< .0002	< 26	0.67		8.01	1.8
476	4/15/2009	Kitchen Sink	Upland		3.16	< .025		< .05		12.5	< .025		0.6		< 5	7.7	
477	6/24/2011	Kitchen Sink	Valley		2.23	< 26	95	1.7	0.0013	4.4	0.055	< .0002	< 26	0.09		8.34	1.5
478	6/24/2011	Outside Hose Bib On The W End Of Home	Valley	Yes	3.09	< 26	130	< .05	< .001	8.1	0.0006	< .0002	< 26	1.9	< 4.7	7.51	1.2
479	4/15/2009	Kitchen Sink	Upland		3.98	< .025		< .05		10.3	< .025		0.47		< 5	7.93	

Table S.8. Groundwater Quality Data for 1701 "Pre-Drill" Water Well Samples from Susquehanna County, Pennsylvania

Water Well ID	Sample Date	Sampling Port	Topo-graphic Location	Gas Extraction Area (Within 1 km of Active Gas Well)	Dissolved Oxygen - Field (mg/L)	Ethane ug/L	Hardness (mg/L)	Iron (mg/L)	Lead (mg/L)	Magnesium (mg/L)	Manganese (mg/L)	Mercury (mg/L)	Methane ug/L	Nitrate as N (mg/L)	Oil Grease (mg/L)	pH - Field (SU)	Potassium (mg/L)
480	3/16/2009	Pressure Tank	Upland		7.68	< .025		< .05		6.27	< .025		0.12		< 5	7.92	
481	6/27/2011	Sample Collected From An Outside Spigot Located On The North Side Of The House.	Valley		0.07	< 26	68	< .05	< .001	5.5	0.082	< .0002	< 26	< .05		8.5	1.1
482	6/27/2011	Kitchen Sink	Valley		3.92	< 26	58	< .05	< .001	2.4	< .0005	< .0002	< 26	0.34		6.94	1.7
483	6/28/2011	Dip Sample Directly From Well. Well Is Locatedon The South End Of Property Inside Of Electric Fence. It Is Covered With Metal Sheeting And An Old Car Hood Frame.	Upland		0.84	< 26	92	0.48	< .001	4.7	3.7	< .0002	< 26	0.055		6.74	5.1
484	6/28/2011	Kitchen Sink Cwt	Valley		2.46	< 26	72	< .05	< .001	5.3	0.0011	< .0002	< 26	0.12		9.04	1.3
485	6/29/2011	Well	Upland		6.94	< 26	44	1.2	0.0011	2.2	0.79	< .0002	< 26	1.2		6.8	1
486	6/29/2011	Laundry Room Sink	Valley		1.4	< 26	84	0.052	< .001	4.6	0.022	< .0002	< 26	0.34		8.38	1.7
487	6/29/2011	Dug Well	Valley		8.43	< 26	35	< .05	< .001	1.7	< .0005	< .0002	< 26	1.2		6.32	0.83
488	6/29/2011		Valley			< 26	48	< .05	< .001	2.4	0.00059	< .0002	< 26	1.9			0.63
489	6/30/2011	Pressure Tank	Upland		7.77	< 26	58	0.66	0.002	2.9	0.0061	< .0002	< 26	2.3		6.59	0.57
490	7/1/2011	Outside Hose Bib	Valley		0.23	< 26	85	0.55	< .001	5	0.2	< .0002	< 26	0.072		7.63	1.1
491	2/12/2009		Valley					0.238		17.4	0.171		9.4		< 5		
492	5/7/2009		Valley			<0.025		6.96		6.14	0.156		0.16				
493	4/26/2009		Valley			49		< .025		7.13	< .025		39000		< 5		

Table S.8. Groundwater Quality Data for 1701 "Pre-Drill" Water Well Samples from Susquehanna County, Pennsylvania

Water Well ID	Sample Date	Sampling Port	Topo-graphic Location	Gas Extraction Area (Within 1 km of Active Gas Well)	Dissolved Oxygen - Field (mg/L)	Ethane ug/L	Hardness (mg/L)	Iron (mg/L)	Lead (mg/L)	Magnesium (mg/L)	Manganese (mg/L)	Mercury (mg/L)	Methane ug/L	Nitrate as N (mg/L)	Oil Grease (mg/L)	pH - Field (SU)	Potassium (mg/L)
494	7/5/2011	Kitchen Sink	Valley		6.03	< 26	96	0.29	< .001	4.1	0.011	< .0002	< 26	1.1		7.63	0.94
495	7/5/2011	Samples Collected By Dipping 1 Inch Plastic Bailer Down The Well.	Upland		6.95	< 26	95	24	0.076	6.8	1.3	< .0002	< 26	0.64		7.62	3.1
496	7/5/2011	Outside Hose Bib On South Of House	Valley		0.09	< 26	160	0.59	0.0019	6.8	2.3	< .0002	< 26	< .05		7.24	1.1
497	7/6/2011	Pressure Tank In Basement	Upland		4.77	< 26	120	< .05	< .001	5.5	0.011	< .0002	< 26	0.57		7.1	1.3
498	7/8/2011	Outside Faucet	Upland		6.06	< 26	< .67	< .05	< .001	< .1	0.0036	< .0002	< 26	0.99		6.44	0.13
499	5/14/2011	Kitchen Sink	Valley	Yes	2.78	< 26	140	< .05	< .001	9.6	0.016	< .0002	< 26	0.073		7.53	1.3
500	5/3/2009	Bathroom Sink	Upland		9.7	< .025		< .05		8.48	< .025		< .1		< 5	7.6	
501	5/19/2011	Outside Spigot	Valley	Yes	7.77	< 26	90	< .05	0.0015	5.3	0.0019	< .0002	< 26	2.4		6.24	2
502	5/25/2011	Pressure Tank	Valley	Yes	0.16	< 26	160	0.59	< .001	12	0.023	< .0002	< 26	0.1		7.42	1.5
503	5/26/2011	Pressure Tank	Valley	Yes	0.23	< 26	180	< .05	< .001	10	0.009	< .0002	< 26	0.42		7.43	1.1
504	10/26/2009		Valley					0.716		4.26	0.139		37				
505	5/26/2011	Outside Hose Bib Cold	Valley	Yes	2.6	< 26	120	< .05	< .001	6.1	< .0005	< .0002	< 26	1.1		7.23	1.2
506	10/26/2009		Valley	Yes				0.13		6.23	< .025		2.5				
507	6/1/2011	Outside Hose	Upland		0.006	< 26	160	6.6	< .001	11	0.36	< .0002	< 26	< .05	< 4.8	7.21	0.69
508	6/1/2011	Pressure Tank	Valley		2.69	< 26	120	0.069	0.0015	6.3	0.06	< .0002	< 26	0.91		7.23	0.95
509	6/2/2011	Pressure Tank In Old Creamery	Upland		5.5	< 26	76	< .05	< .001	3.8	0.0024	< .0002	< 26	1.3		6.42	0.81
510	10/26/2009	Pressure Tank	Valley	Yes				< .05		6.01	< .025		1.6				
511	6/6/2011	Dug Well 20 Feet From Se Corner Of House	Upland		0.15	< 26	200	0.56	< .001	6.7	2.6	< .0002	213	< .05		7.06	2
512	10/26/2009	Pressure Tank	Upland	Yes				< .05		6.79	< .025		0.65				

Table S.8. Groundwater Quality Data for 1701 "Pre-Drill" Water Well Samples from Susquehanna County, Pennsylvania

Water Well ID	Sample Date	Sampling Port	Topo-graphic Location	Gas Extraction Area (Within 1 km of Active Gas Well)	Dissolved Oxygen - Field (mg/L)	Ethane ug/L	Hardness (mg/L)	Iron (mg/L)	Lead (mg/L)	Magnesium (mg/L)	Manganese (mg/L)	Mercury (mg/L)	Methane ug/L	Nitrate as N (mg/L)	Oil Grease (mg/L)	pH - Field (SU)	Potassium (mg/L)
513	6/13/2011	Kitchen Sink	Valley		7.74	< .025							0.16			8.21	
514	6/13/2011	Hand Dug Well	Upland		6.31	< .025							0.52			6.37	
515	6/13/2011	Main Kitchen Sink	Upland		6.67	< .025							0.13			6.76	
516	6/13/2011	Basement Pressure Tank	Valley		6.02	< .025							2.2			7.18	
517	6/13/2011	Exterior Faucet On South Side Of House Between Two Green Garage Doors	Upland		11.55	< .025							0.14			7.14	
518	6/14/2011	Kitchen Sink	Valley		3.82	< .025							120			8.15	
519	6/14/2011	Kitchen Sink	Upland		3.96	< .025							< .1			7.12	
520	6/14/2011	Pressure Tank	Upland		6.02	< .025							0.12			6.74	
521	6/14/2011	Kitchen Sink	Valley		9.23	< .025							0.15			7.22	
522	10/26/2009	Basement At Pressure Tank	Upland	Yes				< .05		5.2	< .025		0.71				
523	6/14/2011	Kitchen Faucet	Valley			2.4							11000				
524	6/14/2011	Kitchen Sink	Valley		2.45	< .025							6.3			7.24	
525	6/14/2011	Kitchen Sink	Upland		5.64	0.093							0.26			6.71	
526	6/14/2011	Kitchen Sink	Valley		5.63	< .025							0.18			7.05	
527	6/14/2011	Pressure Tank	Valley		2.74	< .025							1.2			7.11	
528	6/15/2011		Upland			< .025							< .1				
529	6/15/2011	Kitchen Sink	Upland		9.46	< .025							0.95			6.57	
530	6/15/2011	Spigot At Well House	Upland		8.48	< .025							1.4			7.09	
531	6/15/2011	Kitchen Sink	Upland		6.93	< .025							0.18			6.43	
532	6/15/2011	Kitchen Sink	Upland		17.82	< .025							0.2			5.73	
533	6/15/2011	Kitchen Sink	Upland		7.86	< .025							0.13			6.72	

Table S.8. Groundwater Quality Data for 1701 "Pre-Drill" Water Well Samples from Susquehanna County, Pennsylvania

Water Well ID	Sample Date	Sampling Port	Topo-graphic Location	Gas Extraction Area (Within 1 km of Active Gas Well)	Dissolved Oxygen - Field (mg/L)	Ethane ug/L	Hardness (mg/L)	Iron (mg/L)	Lead (mg/L)	Magnesium (mg/L)	Manganese (mg/L)	Mercury (mg/L)	Methane ug/L	Nitrate as N (mg/L)	Oil Grease (mg/L)	pH - Field (SU)	Potassium (mg/L)
534	6/16/2011	Kitchen Sink	Upland		4.9	< .025							0.12			7.19	
535	6/16/2011	Pressure Tank	Valley		5.83	< .025							0.13			6.69	
536	6/16/2011	Kitchen Sink Overlooking Blanding Road	Upland		9.98	< .025							0.2			6.97	
537	6/16/2011	Kitchen Sink On South Side Of House	Upland		5.2	< .025							< .1			7.5	
538	6/16/2011	Kitchen Sink	Upland		21.92	< .025							0.2			7.45	
539	6/16/2011	Kitchen Sink	Upland		25.54	< .025							0.56			6.55	
540	6/16/2011	Wellhead From Hose	Upland		2.45	0.21							160			8.83	
541	6/16/2011	Kitchen Sink	Valley		9.25	< .025							0.55			7	
542	6/17/2011	Kitchen Sink	Upland		7.09	< .025							< .1			7.13	
543	6/17/2011	Pressure Tank In Basement On Tingley Lake Side Of Road	Valley		8.68	< .025							1.7			6.54	
544	6/20/2011	Pressure Tank	Valley		6.7	< .025							1.4			7.41	
545	6/21/2011	Kitchen Sink	Valley		16.71	< .025							0.25			6.85	
546	6/21/2011	Kitchen Sink	Upland		2.66	< .025							0.14			6.9	
547	6/21/2011	Kitchen Sink	Upland		21.07	< .025							1.2			7.13	
548	4/26/2009	Kitchen Sink	Valley		4.39	< .025		< .025		4.88	< .025		2		< 5	7.4	
549	6/21/2011	Kitchen Sink	Upland		3.16	< .025							0.29			6.42	
550	6/21/2011		Valley			< .025							0.47				
551	6/22/2011	Kitchen Sink	Upland		7.33	< .025							0.13			6.67	
552	6/22/2011	Basement Pressure Tank	Valley		7.48	< .025							0.19			7.24	
553	6/22/2011	Kitchen Sink	Upland		5.92	<0.025							0.33			6.97	
554	6/22/2011	Kitchen Sink	Valley		10.12	0.037							0.34			6.69	

Table S.8. Groundwater Quality Data for 1701 "Pre-Drill" Water Well Samples from Susquehanna County, Pennsylvania

Water Well ID	Sample Date	Sampling Port	Topo-graphic Location	Gas Extraction Area (Within 1 km of Active Gas Well)	Dissolved Oxygen - Field (mg/L)	Ethane ug/L	Hardness (mg/L)	Iron (mg/L)	Lead (mg/L)	Magnesium (mg/L)	Manganese (mg/L)	Mercury (mg/L)	Methane ug/L	Nitrate as N (mg/L)	Oil Grease (mg/L)	pH - Field (SU)	Potassium (mg/L)
555	6/22/2011		Upland			1.1							5300				
556	6/22/2011	Kitchen Sink	Upland		5.29	<0.025							0.22			6.68	
557	6/23/2011	Pressure Tank	Valley		3.19	0.036							95			6.74	
558	6/23/2011	Pressure Tank	Valley		2.84	0.059							330			6.55	
559	6/23/2011	Kitchen Sink	Valley		4.33	< .025							0.17			6.92	
560	6/23/2011	Kitchen Sink	Valley		15.27	< .025							3.4			7.06	
561	6/23/2011		Valley			< .025							0.44				
562	6/23/2011		Upland			< .025							0.14				
563	10/27/2009	Kitchen Sink - Ann'S House	Valley	Yes	6.61	< .025		< .05		7.43	< .025		0.27			7.39	
564	6/24/2011	Spigot	Upland		7.39	< .025							0.16			7.12	
565	6/24/2011	Kitchen Sink	Upland		4.11	< .025							0.16			7.47	
566	6/24/2011	Kitchen Sink	Upland		3.41	< .025							0.27			6.75	
567	6/24/2011	Kitchen Sink	Upland		3.82	< .025							0.19			7.37	
568	6/24/2011	Kitchen Sink	Valley		1.51	0.48							1800			7.91	
569	6/24/2011	Kitchen Sink	Valley		1.41	0.36							5800			7.77	
570	6/24/2011	Kitchen Sink	Upland		4.65	< .025							0.2			7.46	
571	6/24/2011	Kitchen Sink	Upland		4.12	< .025							0.34			7	
572	6/24/2011		Upland			< .025							0.1				
573	6/27/2011	Kitchen Sink	Valley		3.02	0.054							160			7.22	
574	6/27/2011		Valley			< .025							0.13				
575	6/27/2011	House Kitchen Sink	Valley		1.68	0.36							2800			7.14	
576	6/27/2011		Valley		9.75	<0.025							0.15			6.87	
577	6/27/2011	Kitchen Sink	Upland		4.01	< .025							0.4			6.88	
578	6/27/2011	Kitchen Sink	Upland		6.78	< .025							0.16			6.84	
579	6/27/2011	Outside Spigot	Valley		1.33	0.4							3300			7.1	
580	6/28/2011	Kitchen Sink	Valley		2.04	< .025							670			6.69	
581	6/28/2011		Upland			< .025							0.32				
582	6/28/2011	Outside Spigot	Upland		3.98	< .025							0.17			6.62	
583	6/28/2011		Valley			< .025							0.5				

Table S.8. Groundwater Quality Data for 1701 "Pre-Drill" Water Well Samples from Susquehanna County, Pennsylvania

Water Well ID	Sample Date	Sampling Port	Topo-graphic Location	Gas Extraction Area (Within 1 km of Active Gas Well)	Dissolved Oxygen - Field (mg/L)	Ethane ug/L	Hardness (mg/L)	Iron (mg/L)	Lead (mg/L)	Magnesium (mg/L)	Manganese (mg/L)	Mercury (mg/L)	Methane ug/L	Nitrate as N (mg/L)	Oil Grease (mg/L)	pH - Field (SU)	Potassium (mg/L)
584	6/28/2011	Kitchen Sink	Upland		18.34	< .025							0.18			7.82	
585	6/28/2011		Valley			< .025							0.33				
586	10/27/2009	Pressure Tank	Upland					0.134		4.64	< .025		0.29				
587	6/28/2011	Kitchen Sink	Valley		4.71	< .025							0.27			6.89	
588	6/28/2011	Basement Pressure Tank	Upland		5.1	< .025							0.41			7.24	
589	6/29/2011	Well Spigot	Upland		3.59	< .025							13			6.5	
590	6/29/2011		Valley			< .025							40				
591	6/29/2011		Upland			<0.025							0.76				
592	6/29/2011		Valley			< .025							< .1				
593	6/29/2011		Valley			< .025							1.5				
594	6/29/2011		Upland			< .025							0.44				
595	6/29/2011	House Spigot	Valley		2.26	< .025							13			6.85	
596	6/29/2011	Kitchen Sink (Dennis)	Upland		5.03	0.37							1900			8.49	
597	6/30/2011	Kitchen Sink	Valley		2.63	< .025							0.31			7.43	
598	10/27/2009	Pressure Tank	Valley					< .05		3.97	< .025		0.12				
599	6/30/2011	Pressure Tank	Valley		7.8	< .025							0.18			6.74	
600	6/30/2011		Valley			< .025							0.5				
601	6/30/2011	Kitchen Sink	Upland		11.94	< .025							0.16			6.91	
602	6/30/2011	Pressure Tank	Valley		5.09	< .025							0.18			7.21	
603	6/30/2011		Valley			< .025							0.31				
604	6/30/2011		Valley			< .025							0.15				
605	6/30/2011		Valley			< .025							0.47				
606	6/30/2011	Sink In Bays Of Garage	Upland		3.21	< .025							0.3			7.16	
607	10/27/2009	Pressure Tank	Upland					0.272		5.22	< .025		0.22				
608	6/30/2011	Basement Pressure Tank	Valley		8.87	< .025							< .1			6.23	
609	6/30/2011	Outside Spigot	Upland		8.66	< .025							0.4			6.99	
610	6/30/2011		Upland		6.18	<0.025							0.17			7.04	
611	6/30/2011	Kitchen Sink	Upland			< .025							2.4				
612	6/30/2011	Sink In Barn	Valley		3.04	< .025							0.99			6.61	
613	7/1/2011	Kitchen Sink	Upland		15.51	< .025							0.18			7.32	

Table S.8. Groundwater Quality Data for 1701 "Pre-Drill" Water Well Samples from Susquehanna County, Pennsylvania

Water Well ID	Sample Date	Sampling Port	Topo-graphic Location	Gas Extraction Area (Within 1 km of Active Gas Well)	Dissolved Oxygen - Field (mg/L)	Ethane ug/L	Hardness (mg/L)	Iron (mg/L)	Lead (mg/L)	Magnesium (mg/L)	Manganese (mg/L)	Mercury (mg/L)	Methane ug/L	Nitrate as N (mg/L)	Oil Grease (mg/L)	pH - Field (SU)	Potassium (mg/L)
614	7/1/2011	Kitchen Sink	Upland		18.8	< .025							520			6.29	
615	7/1/2011	Kitchen Sink	Upland			< .025							0.14				
616	7/1/2011	Kitchen Sink	Upland		19.35	< .025							0.11			7.56	
617	7/1/2011	Kitchen Sink	Valley		2.05	0.032							420			7.32	
618	7/1/2011	Outside Spigot	Upland		6.1	< .025							0.18			7.3	
619	7/1/2011		Valley			< .025							0.42				
620	7/1/2011	Outside Spigot	Valley			0.025							0.54				
621	7/1/2011	Wellhead Spigot	Valley			< .025							3.2				
622	7/5/2011	Bathroom Sink	Valley		7.01	0.16							1200			7.6	
623	7/5/2011	Kitchen Sink	Upland		6.93	< .025							0.14			6.97	
624	7/5/2011	Basement Pressure Tank	Upland		4.89	< .025							0.39			6.92	
625	7/5/2011		Valley			0.27							2300				
626	7/5/2011	Kitchen Sink	Valley		5.24	< .025							0.13			7.3	
627	10/27/2009	Pressure Tank	Valley					< .05		1.38	< .025		260				
628	7/5/2011	Kitchen Sink	Upland		6.65	< .025							0.25			7.32	
629	7/5/2011	Outside Spigot	Valley			< .025							0.18				
630	7/5/2011	Kitchen Sink	Upland			< .025							0.34				
631	7/5/2011	Kitchen Sink	Valley			< .025							0.23				
632	7/5/2011	Outside Spigot	Valley			< .025							0.18				
633	7/5/2011	Kitchen Sink	Valley			< .025							0.14				
634	7/5/2011	Kitchen Sink	Valley		8.7	< .025							1.5			6.78	
635	7/5/2011	Kitchen Sink Main Camp	Valley		34.8	< .025							0.12			6.22	
636	7/5/2011	Pressure Tank	Valley		5.61	< .025							0.36			7.56	
637	7/5/2011	Kitchen Sink	Upland			< .025							0.32				
638	7/5/2011	Kitchen Faucet	Valley		3.97	< .025							54			7.55	

Table S.8. Groundwater Quality Data for 1701 "Pre-Drill" Water Well Samples from Susquehanna County, Pennsylvania

Water Well ID	Sample Date	Sampling Port	Topo-graphic Location	Gas Extraction Area (Within 1 km of Active Gas Well)	Dissolved Oxygen - Field (mg/L)	Ethane ug/L	Hardness (mg/L)	Iron (mg/L)	Lead (mg/L)	Magnesium (mg/L)	Manganese (mg/L)	Mercury (mg/L)	Methane ug/L	Nitrate as N (mg/L)	Oil Grease (mg/L)	pH - Field (SU)	Potassium (mg/L)
639	7/5/2011	Kitchen Sink	Valley		2.96	< .025							6.6			7.88	
640	7/5/2011	Kitchen Sink	Upland		3.84	< .025							0.24			7.56	
641	7/5/2011	Kitchen Sink	Upland		2.43	0.058							760			8.37	
642	7/6/2011	Kitchen Sink	Upland		1.67	0.82							2600			8.86	
643	7/6/2011	Kitchen Sink	Valley		2.88	< .025							0.16			7.75	
644	7/6/2011	Kitchen Sink	Upland		7.47	< .025							0.24			7.29	
645	7/6/2011		Upland			< .025							0.1				
646	7/6/2011	Farm Sink	Valley			< .025							7.2				
647	7/6/2011		Upland			< .025							0.13				
648	7/6/2011	Kitchen Sink	Valley		3.03	0.15							600			7.28	
649	7/6/2011		Valley			< .025							0.12				
650	7/6/2011	Kitchen Sink	Valley		5.22	< .025							0.57			6.98	
651	7/6/2011	Kitchen Sink	Valley		10.16	< .025							< .1			7.72	
652	7/6/2011	Basement Pressure Tank	Valley		2.84	< .025							0.28			7.38	
653	7/6/2011	House Well	Upland		10.48	< .025							0.23			6.39	
654	7/6/2011	Outside Spigot	Valley		4.24	< .025							1.2			7.22	
655	7/7/2011	Pressure Tank In Basement	Upland		5.56	< .025							0.14			7.28	
656	7/7/2011	Kitchen Sink	Upland		3.39	< .025							0.27			7.53	
657	7/7/2011	Outside Faucet	Upland		2.46	< .025							0.15			7.24	
658	7/7/2011	Kitchen Sink	Valley		13.65	< .025							< .1			6.39	
659	7/7/2011	Kitchen Sink	Upland		7.21	0.36							2300			5.51	
660	7/7/2011	Kitchen Sink	Upland			< .025							0.24				
661	7/7/2011	Outside Faucet	Valley			< .025							22				
662	7/7/2011	Garage Spigot	Valley			< .025							< .1				
663	7/7/2011	Kitchen Sink	Valley		10.42	<0.025							<0.1			6.68	
664	7/7/2011	Pressure Tank	Upland		14.9	< .025							1.2			6.39	
665	7/7/2011	Kitchen Sink	Upland		5.87	< .025							0.63			6.17	

Table S.8. Groundwater Quality Data for 1701 "Pre-Drill" Water Well Samples from Susquehanna County, Pennsylvania

Water Well ID	Sample Date	Sampling Port	Topo-graphic Location	Gas Extraction Area (Within 1 km of Active Gas Well)	Dissolved Oxygen - Field (mg/L)	Ethane ug/L	Hardness (mg/L)	Iron (mg/L)	Lead (mg/L)	Magnesium (mg/L)	Manganese (mg/L)	Mercury (mg/L)	Methane ug/L	Nitrate as N (mg/L)	Oil Grease (mg/L)	pH - Field (SU)	Potassium (mg/L)
666	7/7/2011	Kitchen Sink	Valley			< .025							< .1				
667	7/7/2011	Kitchen Sink	Upland		12.07	< .025							0.66			5.75	
668	7/7/2011	Kitchen Sink	Upland		3.13	< .025							0.51			7.47	
669	7/7/2011	Bunk House Sink	Upland		2.17	0.029							5.3			7.26	
670	7/7/2011	Spigot At Well Head	Valley		9.82	< .025							< .1			6.76	
671	7/8/2011	Kitchen Sink	Valley		2.73	< .025							3			7.03	
672	7/8/2011	Kitchen Faucet	Upland		18.23	< .025							0.75			6.4	
673	7/8/2011	Pressure Tank	Valley			< .025							< .1				
674	7/8/2011	Kitchen Faucet	Upland			< .025							< .1				
675	7/8/2011	Kitchen Faucet	Upland		22.94	< .025							0.27			6.23	
676	7/8/2011	Kitchen Sink	Upland		7.22	< .025							< .1			7.05	
677	7/8/2011	Kitchen Sink	Upland		4.78	< .025							0.11			7.28	
678	7/8/2011	Kitchen Sink	Valley		5.15	< .025							0.38			6.87	
679	7/8/2011	Spigot In Cooling Barn	Upland		9.74	< .025							0.89			6.09	
680	7/8/2011	Kitchen Sink	Upland		8.83	< .025							0.98			6.61	
681	7/8/2011	Shop Sink	Upland		13.49	< .025							0.1			6.05	
682	7/8/2011	Kitchen Sink	Upland		33.31	< .025							0.5			6.68	
683	7/9/2011	Kitchen Sink	Upland			< .025							0.18				
684	7/11/2011	Spigot On Front Of House	Valley		1.87	0.05							1.6			7.44	
685	7/11/2011	Spigot Near Water Fountain	Valley		7.74	0.088							150			6.83	
686	7/11/2011	Outside Spigot	Upland			0.3							1700				
687	7/11/2011	Kitchen Sink	Valley			< .025							2.3				
688	7/11/2011	Kitchen Sink	Valley			< .025							29				
689	7/11/2011	Kitchen Sink	Valley		3.87	< .025							2.5			7.36	

Table S.8. Groundwater Quality Data for 1701 "Pre-Drill" Water Well Samples from Susquehanna County, Pennsylvania

Water Well ID	Sample Date	Sampling Port	Topo-graphic Location	Gas Extraction Area (Within 1 km of Active Gas Well)	Dissolved Oxygen - Field (mg/L)	Ethane ug/L	Hardness (mg/L)	Iron (mg/L)	Lead (mg/L)	Magnesium (mg/L)	Manganese (mg/L)	Mercury (mg/L)	Methane ug/L	Nitrate as N (mg/L)	Oil Grease (mg/L)	pH - Field (SU)	Potassium (mg/L)
690	11/5/2009	Pressure Tank	Upland	Yes	4.14	< .025		< .05		7.14	< .025		0.51			8.1	
691	7/11/2011	Kitchen Sink	Upland		5.9	< .025							0.42			7.3	
692	7/11/2011	Kitchen Sink	Upland		4.85	< .025							0.61			6.62	
693	7/11/2011	Kitchen Sink	Upland		16.61	< .025							0.39			5.87	
694	7/11/2011	Hand Dug Well	Upland		9.68	< .025							0.17			6.8	
695	7/11/2011	Kitchen Sink	Valley		2.3	4.2							11000			7.84	
696	7/12/2011	Bathroom Sink Downstairs	Valley			< .025							0.46				
697	7/12/2011	Kitchen Sink	Upland			0.033							260				
698	7/12/2011	Kitchen Faucet	Valley		4.55	0.031							210			7.59	
699	7/12/2011	Kitchen Sink	Valley		15.91	< .025							0.22			5.95	
700	7/12/2011	Kitchen Sink	Valley		6.71	0.92							3300			8.48	
701	7/12/2011	Pressure Tank	Upland		3.26	< .025							0.13			7.78	
702	7/12/2011	Kitchen Sink	Upland			< .025							< .1				
703	7/12/2011	Spigot Before Filter	Valley			< .025							4.2				
704	7/12/2011	Kitchen Sink	Valley			< .025							0.34				
705	7/12/2011		Valley			< .025							2.2				
706	7/12/2011	Pressure Tank	Upland		5.25	< .025							0.45			7.72	
707	7/12/2011	Inside Faucet	Upland		2.1	< .025							0.37			7.74	
708	7/12/2011	Kitchen Sink	Upland		3.45	< .025							0.71			7.93	
709	7/12/2011	Spigot	Upland		6.01	< .025							4.9			8.12	
710	7/12/2011	Spigot	Upland		3.26	< .025							0.21			7.93	
711	7/12/2011	Kitchen Sink	Valley		2.54	0.055							140			7.96	
712	7/12/2011	Kitchen Faucet	Upland		8.77	< .025							7.6			6.91	
713	7/12/2011	Kitchen Faucet	Valley		10.42	< .025							0.11			6.72	
714	7/13/2011	Kitchen Sink	Valley		9.77	< .017							73			8.26	
715	7/13/2011	Kitchen Sink	Valley		10.08	< .025							0.26			7.76	

Table S.8. Groundwater Quality Data for 1701 "Pre-Drill" Water Well Samples from Susquehanna County, Pennsylvania

Water Well ID	Sample Date	Sampling Port	Topo-graphic Location	Gas Extraction Area (Within 1 km of Active Gas Well)	Dissolved Oxygen - Field (mg/L)	Ethane ug/L	Hardness (mg/L)	Iron (mg/L)	Lead (mg/L)	Magnesium (mg/L)	Manganese (mg/L)	Mercury (mg/L)	Methane ug/L	Nitrate as N (mg/L)	Oil Grease (mg/L)	pH - Field (SU)	Potassium (mg/L)
716	7/13/2011	Kitchen Sink	Upland		9.01	< .025							0.11			6.62	
717	7/13/2011	Spigot	Valley		10.54	< .025							0.2			6.59	
718	7/13/2011	Spigot On Well	Valley		3.85	< .025							0.51			6.12	
719	7/13/2011	Barn Spigot	Valley		4.3	< .025							< .1			7.1	
720	7/13/2011	Kitchen Sink	Valley		9.78	< .025							0.46			7.63	
721	7/13/2011	Garage Spigot	Upland		10.75	< .025							0.26			8.09	
722	7/13/2011	Kitchen Sink	Valley		11.28	0.026							0.65			7.92	
723	7/13/2011	Kitchen Sink	Valley		10.78	0.52							2200			7.85	
724	7/8/2011		Upland			< .025							1.3				
725	7/8/2011	Kitchen Sink	Upland			< .025							0.12				
726	4/13/2011	Milkhouse Faucet Barn Well	Valley		6.73	0.27	12.33526	< .05	< .0003	0.57	0.011	< .0002	2400	< 1	< 5	8.67	2.6
727	4/13/2011	Tenant'S Pressure Tank	Upland		11.84	< .025	39.8472	< .05	0.0019	2.4	< .0025	< .0002	0.072	< 1	< 5	5.92	3.3
728	4/13/2011	Outside Spigot On Garage	Upland			< .025	83.891	1.2	0.0014	4	0.12	< .0002	5.8	< 1	< 5		2.2
729	4/26/2011	Pressure Tank	Valley		10.8	< .025	174.835	1.4	0.023	8.5	3	< .0002	0.23	< 1	< 5	7.03	1.7
730	6/17/2011	Spigot On Front Of House	Upland		3.5	< .025							0.13			7.18	
731	6/17/2011	Kitchen Sink	Valley		7.68	< .025							< .1			7.21	
732	6/17/2011	Spigot Inside Of Shed On Right	Upland		5.15	< .025							14			6.92	
733	6/17/2011	Kitchen Sink	Valley		3.02	< .025							5.7			7.02	
734	6/17/2011	Pressure Tank	Valley		4.79	< .025							9			6.27	
735	6/20/2011	Kitchen Sink	Upland		1.38	< .025							15			7.72	
736	6/20/2011	Pressure Tank	Valley		1.96	< .025							0.34			7.22	
737	6/20/2011	Kitchen Sink	Upland		3.18	< .025							0.2			7.2	
738	6/20/2011	Kitchen Sink	Valley		1.45	< .025							94			6.83	
739	6/20/2011	Kitchen Sink	Valley		1.54	< .025							48			7.39	

Table S.8. Groundwater Quality Data for 1701 "Pre-Drill" Water Well Samples from Susquehanna County, Pennsylvania

Water Well ID	Sample Date	Sampling Port	Topo-graphic Location	Gas Extraction Area (Within 1 km of Active Gas Well)	Dissolved Oxygen - Field (mg/L)	Ethane ug/L	Hardness (mg/L)	Iron (mg/L)	Lead (mg/L)	Magnesium (mg/L)	Manganese (mg/L)	Mercury (mg/L)	Methane ug/L	Nitrate as N (mg/L)	Oil Grease (mg/L)	pH - Field (SU)	Potassium (mg/L)
740	6/20/2011	Kitchen Sink	Valley		1.3	0.13							700			7.68	
741	6/21/2011	Kitchen Sink	Upland		26.55	< .025							0.1			6.63	
742	6/21/2011	Kitchen Sink	Valley		14.39	< .025							1.4			7.22	
743	6/21/2011	Kitchen Sink	Valley		1.64	< .025							23			7.64	
744	6/21/2011	Kitchen Sink	Valley		1.39	< .025							0.13			7.35	
745	6/21/2011		Valley			< .025							< .1				
746	6/21/2011	Outside Spigot	Valley		12.98	0.11							1300			7.17	
747	6/21/2011	Kitchen Sink	Valley		1.95	< .025							210			7.35	
748	6/21/2011	Wellhead Spigot	Valley		12.36	< .025							0.92			7.3	
749	6/21/2011	Kitchen Sink	Upland		2.42	< .025							16			7.38	
750	6/21/2011		Upland			0.15							500				
751	6/29/2011	Kitchen Sink	Upland		5.85	< .025							0.15			6.45	
752	6/29/2011	Barn Spigot	Valley		69.01	< .025							0.13			6.61	
753	7/8/2011	Well 2 On South Side Of House	Upland		12.86	< .025							1.1				
754	7/8/2011	Kitchen Sink	Upland		15.54	< .025							0.13				
755	7/8/2011	Kichen Sink	Valley			< .025							0.11				
756	7/9/2011	Kitchen Sink	Valley			< .025							2				
757	7/9/2011	Spring	Upland			< .025							0.73				
758	7/11/2011	Kitchen Sink	Valley			< .025							0.22				
759	7/11/2011	Kitchen Sink	Valley		8.76	< .025							0.18			7.09	
760	7/11/2011	Kitchen Sink	Valley		17.76	< .025							< .1			7.5	
761	11/5/2009	Outside Faucet	Valley	Yes	6.33	< .025		0.927		5.98	0.025		94			8.26	
762	7/12/2011	Kitchen Sink	Valley		10.17	< .025							0.14			5.53	
763	7/12/2011	Kitchen Sink	Valley		9.34	< .025							0.16			4.45	
764	7/14/2011	Kitchen Sink	Upland		14.48	< .025							0.66			7.81	
765	7/14/2011	Kitchen Sink	Upland		10.6	< .025							0.37			7.23	
766	7/14/2011	Kitchen Sink	Valley		11.04	2.1							12000			7.91	

Table S.8. Groundwater Quality Data for 1701 "Pre-Drill" Water Well Samples from Susquehanna County, Pennsylvania

Water Well ID	Sample Date	Sampling Port	Topo-graphic Location	Gas Extraction Area (Within 1 km of Active Gas Well)	Dissolved Oxygen - Field (mg/L)	Ethane ug/L	Hardness (mg/L)	Iron (mg/L)	Lead (mg/L)	Magnesium (mg/L)	Manganese (mg/L)	Mercury (mg/L)	Methane ug/L	Nitrate as N (mg/L)	Oil Grease (mg/L)	pH - Field (SU)	Potassium (mg/L)
767	7/14/2011	Spigot On Side Of House	Valley		3.07	1.4							6100			7.49	
768	7/14/2011	Kitchen Sink	Valley		10.65	2.9							11000			7.81	
769	7/14/2011	Kitchen Sink	Valley		1.7	1.4							7700			8.5	
770	7/14/2011	Kitchen Sink	Valley		4.69	< .025							0.59			7.67	
771	7/14/2011	Kitchen Sink	Upland		7.77	< .1							0.28			7.75	
772	7/14/2011	Kitchen Sink	Valley		1.11	0.1							1100			8.13	
773	7/14/2011	Spigot	Valley		5.62	3.5							13000			7.9	
774	7/15/2011	Kitchen Sink	Valley		3.55	< .025							< .1			7.51	
775	7/15/2011	Kitchen Sink	Valley		2.54	0.054							6.3			7.64	
776	7/15/2011	Kitchen Sink	Upland		9.2	< .025							0.26			7.32	
777	7/15/2011	Kitchen Sink	Upland		7.86	< .025							0.92			7.5	
778	7/15/2011	Outside Faucet	Valley		8.06	0.073							640			7.37	
779	7/15/2011	Kitchen Sink	Valley		5.29	< .025							0.13			7.17	
780	7/15/2011	Spigot East Of House	Valley		7.34	< .025							0.15			6.9	
781	7/15/2011	Outside Faucet	Upland		5.13	< .025							0.45			7.17	
782	7/15/2011	Outside Faucet	Upland		10.87	< .025							0.36			7.63	
783	7/15/2011	Kitchen Sink	Upland		5.58	< .025							1.1			7.34	
784	7/15/2011	Kitchen Sink	Valley		9.93	0.21							1500			7.83	
785	7/15/2011	Kitchen Sink	Valley		3.09	< .025							0.13			7.75	
786	7/15/2011	Kitchen Sink	Valley		4.01	< .025							1.2			7.52	
787	7/15/2011	Kitchen Sink	Valley		3.71	< .025							0.1			7.65	
788	7/15/2011	Kitchen Sink	Valley		9.67	< .025							0.62			7.64	
789	7/15/2011	Kitchen Sink	Valley		9.4	< .025							0.6			6.95	
790	7/15/2011	Kitchen Sink	Valley		8.95	< .025							0.67			7.7	
791	7/15/2011	Kitchen Sink	Upland		3.36	< .025							2.5			7.7	

Table S.8. Groundwater Quality Data for 1701 "Pre-Drill" Water Well Samples from Susquehanna County, Pennsylvania

Water Well ID	Sample Date	Sampling Port	Topo-graphic Location	Gas Extraction Area (Within 1 km of Active Gas Well)	Dissolved Oxygen - Field (mg/L)	Ethane ug/L	Hardness (mg/L)	Iron (mg/L)	Lead (mg/L)	Magnesium (mg/L)	Manganese (mg/L)	Mercury (mg/L)	Methane ug/L	Nitrate as N (mg/L)	Oil Grease (mg/L)	pH - Field (SU)	Potassium (mg/L)
792	7/15/2011	Spigot On Rear Of House	Valley		4.88	< .025							0.19			7.42	
793	7/15/2011	Kitchen Sink	Valley		9.11	< .025							0.11			6.4	
794	7/15/2011	Kitchen Sink	Valley		9.94	< .025							0.2			7.73	
795	7/16/2011	Kitchen Faucet	Upland		10.8	< .025							1.1			6.98	
796	7/16/2011	Kitchen Faucet	Valley		5.64	< .025							0.62			6.91	
797	7/16/2011	Kitchen Faucet	Upland		14.08	< .025							0.57			7.48	
798	7/16/2011	Kitchen Faucet	Upland		18.35	< .025							1.2			6.81	
799	7/16/2011	Kitchen Sink	Upland		12.87	0.17							1300			6.59	
800	7/17/2011	Outside Spigot In Back Of House	Valley		9.61	0.076							200			7.86	
801	7/17/2011	Outside Spigot On Front Of House	Valley		5.62	< .025							64			7.74	
802	7/17/2011	Well	Upland		17.32	< .025							0.21			6.15	
803	7/17/2011	Blue Well 40Ft From Road	Upland		9.13	0.053							0.7			6.73	
804	7/17/2011	Kitchen Faucet	Upland		13.45	0.053							0.12			7.34	
805	7/17/2011	Spigot From Garage	Valley		12.66	< .025							0.14			6.08	
806	7/18/2011	Outside Spigot	Upland		8.95	< .025							0.4			7.88	
807	7/18/2011	Kitchen Sink	Upland		9.17	< .025							0.23			6.35	
808	7/18/2011	Outside Spigot	Upland		6	< .025							0.14			6.79	
809	7/18/2011	Kitchen Sink	Valley		5.36	3							14000			7.41	
810	7/18/2011	Kitchen Sink	Upland		9.93	< .025							0.12			6.53	
811	7/18/2011	Kitchen Sink	Valley		9.84	< .025							0.22			6.29	
812	7/18/2011	Kitchen Sink	Valley		5.94	0.15							600			6.18	
813	7/19/2011		Upland			< .025							< .1				
814	7/19/2011	Garage Sink	Valley		3.92	<0.025							0.15			7.4	
815	7/19/2011	Kitchen Sink	Valley		1.83	< .025							2.7			7.25	

Table S.8. Groundwater Quality Data for 1701 "Pre-Drill" Water Well Samples from Susquehanna County, Pennsylvania

Water Well ID	Sample Date	Sampling Port	Topo-graphic Location	Gas Extraction Area (Within 1 km of Active Gas Well)	Dissolved Oxygen - Field (mg/L)	Ethane ug/L	Hardness (mg/L)	Iron (mg/L)	Lead (mg/L)	Magnesium (mg/L)	Manganese (mg/L)	Mercury (mg/L)	Methane ug/L	Nitrate as N (mg/L)	Oil Grease (mg/L)	pH - Field (SU)	Potassium (mg/L)
816	7/19/2011	Barn 2 Pressure Tank	Valley		2.8	< .025							1200			7.55	
817	7/19/2011	Spigot On Side Of Barn	Upland		4.96	< .025							0.11			7.04	
818	7/19/2011	Well Head	Valley		9.77	< .025							5.6			7.12	
819	11/23/2009		Upland	Yes		110		< .05		6.48	< .025		8300				
820	7/19/2011	Kitchen Sink	Valley		8.79	< .025							0.25			6.32	
821	7/19/2011	Kitchen Sink	Valley		10.07	< .025							0.12			7.41	
822	7/19/2011	Kitchen Sink	Valley		2.26	< .025							0.15			7.53	
823	7/19/2011	Kitchen Sink	Valley		6.06	< .025							1.9			7.28	
824	7/20/2011	Outside Faucet	Upland		5.29	< .025							0.34			6.94	
825	7/20/2011	Inside Spigot	Upland		5.04	< .025							0.15			7.61	
826	7/20/2011	Kitchen Sink	Upland		7.72	< .025							< .1			6.25	
827	7/20/2011	Kitchen Sink	Valley		8.53	< .025							0.24			6.22	
828	7/20/2011	Kitchen Sink	Upland		4.64	< .025							0.19			7.47	
829	7/20/2011	Kitchen Sink	Valley		9.48	< .025							0.16			7.75	
830	7/20/2011	Outside Spigot	Upland		14.32	< .025							0.16			6.82	
831	7/20/2011	Kitchen Sink	Valley		8.51	< .025							0.16			6.55	
832	7/20/2011	Kitchen Sink	Valley		4.24	< .025							< .1			7.18	
833	7/20/2011	Outside Faucet	Valley		5.24	< .025							0.54			7.86	
834	7/20/2011	Kitchen Sink	Upland		7.05	< .025							0.41			6.86	
835	7/20/2011	Kitchen Sink	Upland		5.25	< .025							13			7.97	
836	7/21/2011	Outside Faucet	Upland		4.68	< .025							0.66			6.98	
837	7/21/2011	Spigot In Front Of House	Upland		8.15	< .025							< .1			7.58	
838	7/21/2011	Kitchen Sink	Valley		1.43	< .025							1.5			7.62	
839	7/21/2011	Kitchen Sink	Upland		2.46	< .025							0.14			7.5	
840	7/21/2011	Kitchen Sink	Upland		5.06	< .025							0.16			7.52	

Table S.8. Groundwater Quality Data for 1701 "Pre-Drill" Water Well Samples from Susquehanna County, Pennsylvania

Water Well ID	Sample Date	Sampling Port	Topo-graphic Location	Gas Extraction Area (Within 1 km of Active Gas Well)	Dissolved Oxygen - Field (mg/L)	Ethane ug/L	Hardness (mg/L)	Iron (mg/L)	Lead (mg/L)	Magnesium (mg/L)	Manganese (mg/L)	Mercury (mg/L)	Methane ug/L	Nitrate as N (mg/L)	Oil Grease (mg/L)	pH - Field (SU)	Potassium (mg/L)
841	7/21/2011	Kitchen Sink	Valley		3.25	<0.025							1.8			8.07	
842	7/21/2011	Kitchen Sink	Valley		3.67	0.061							620			6.37	
843	7/21/2011	Kitchen Sink	Upland		7.95	< .025							0.12			6.82	
844	7/21/2011	Pressure Tank	Upland		4.23	< .025							4.7			6.4	
845	7/21/2011	Basement Sink	Upland		9.56	< .025							< .1			7.88	
846	7/21/2011	Kitchen Sink	Upland		7.28	< .025							< .1			7.03	
847	7/21/2011	Spigot In Rear Of House	Upland		3.45	< .025							3.2			7.63	
848	7/21/2011	Kitchen Sink	Valley		2.42	< .025							2.1			7.62	
849	7/21/2011	Kitchen Sink	Valley		4.7	< .025							0.18			7.94	
850	7/22/2011	Kitchen Sink	Valley		6.74	< .025							0.38			6.5	
851	7/22/2011	Outside Spigot	Valley		2.91	0.35							2300			8.41	
852	7/22/2011	Kitchen Sink	Valley		2.41	< .025							2.2			7.83	
853	7/22/2011	Kitchen Sink	Valley		4.62	< .025							0.56			7.29	
854	7/22/2011	Kitchen Sink	Valley		6.3	< .025							0.26			7.17	
855	7/22/2011	Pipe Overflow	Valley		3.6	1.7							7900			6.12	
856	7/22/2011	Kitchen Sink	Valley		4.25	0.045							9.4			7.49	
857	7/22/2011	Pressure Tank In Garage	Valley		2.29	6.7							17000			7.29	
858	11/5/2009	Basement At Pressure Tank	Valley	Yes	6.14	< .025		0.16		6.45	0.066		2.7			8.09	
859	7/22/2011	Outside Spigot	Valley		4.95	< .025							< .1			7.88	
860	7/22/2011	Kitchen Faucet	Valley		4.27	< .025							3.2			6.68	
861	7/22/2011	Spigot Under Porch	Valley		4.34	< .025							< .1			6.08	
862	7/22/2011	Kitchen Sink	Valley		1.36	3.4							12000			8.33	
863	7/22/2011	Pressure Tank In Garage	Valley		3.84	4.6							18000			6.85	

Table S.8. Groundwater Quality Data for 1701 "Pre-Drill" Water Well Samples from Susquehanna County, Pennsylvania

Water Well ID	Sample Date	Sampling Port	Topo-graphic Location	Gas Extraction Area (Within 1 km of Active Gas Well)	Dissolved Oxygen - Field (mg/L)	Ethane ug/L	Hardness (mg/L)	Iron (mg/L)	Lead (mg/L)	Magnesium (mg/L)	Manganese (mg/L)	Mercury (mg/L)	Methane ug/L	Nitrate as N (mg/L)	Oil Grease (mg/L)	pH - Field (SU)	Potassium (mg/L)
864	7/22/2011	Kitchen Sink	Upland		5.84	< .025							0.23			7.49	
865	7/22/2011	Kitchen Sink	Upland		5.82	< .025							0.17			7.72	
866	7/22/2011	Kitchen Sink	Upland		5.61	0.025							1.2			7.9	
867	11/5/2009	Kitchen Sink	Upland	Yes	3.34	< .025		< .05		< 1	< .025		0.72			8.19	
868	7/22/2011	Kitchen Sink	Valley		4.23	120							12000			7.64	
869	7/22/2011	Pressure Tank	Valley		5.12	2.6							8900			8.68	
870	7/25/2011	Kitchen Sink	Valley		5.31	0.046							360			6.11	
871	7/25/2011	Kitchen Sink	Valley		2.27	0.21							1600			6.99	
872	7/25/2011	Kitchen Sink	Upland		5.79	< .025							0.22			6.68	
873	7/25/2011	Kitchen Sink	Upland			< .025							110			6.5	
874	7/25/2011	Kitchen Sink	Upland		2.39	0.16							870			6.56	
875	7/25/2011	Kitchen Sink	Upland		4.84	< .025							3.6			6.89	
876	7/25/2011	Warehouse At Quarry	Upland		10.48	0.026							0.44			6.54	
877	7/25/2011	Kitchen Sink	Valley		1.73	0.1							1800			8.51	
878	7/25/2011	Kitchen Sink	Upland		4.73	< .025							0.39			7.84	
879	7/25/2011	Kitchen Sink	Valley		1.53	1.9							8800			8.26	
880	1/3/2010	Pressure Tank	Upland	Yes	6.54	< .025		< .05		6.86	< .025		5.6			7.58	
881	7/25/2011	Kitchen Sink	Valley		1.75	< .025							0.34			7.83	
882	7/25/2011	Pressure Tank	Valley		5.67	2.8							12000			8.11	
883	7/26/2011	Kitchen Sink	Valley		2.1	< .025							0.43			7.86	
884	7/26/2011	Kitchen Sink	Valley		7.79	< .025							0.19			6.11	
885	7/26/2011	Kitchen Sink	Valley		4.06	0.067							520			6.59	
886	7/26/2011	Kitchen Sink	Valley		8.27	0.063							1900			6.87	
887	7/26/2011	Kitchen Sink	Valley		7.84	< .025							0.41			6.35	
888	12/22/2009	Pressure Tank	Upland	Yes	9.47	< .025		< .05		1.82	< .025		1.2			7.21	

Table S.8. Groundwater Quality Data for 1701 "Pre-Drill" Water Well Samples from Susquehanna County, Pennsylvania

Water Well ID	Sample Date	Sampling Port	Topo-graphic Location	Gas Extraction Area (Within 1 km of Active Gas Well)	Dissolved Oxygen - Field (mg/L)	Ethane ug/L	Hardness (mg/L)	Iron (mg/L)	Lead (mg/L)	Magnesium (mg/L)	Manganese (mg/L)	Mercury (mg/L)	Methane ug/L	Nitrate as N (mg/L)	Oil Grease (mg/L)	pH - Field (SU)	Potassium (mg/L)
889	7/26/2011	Kitchen Sink	Upland		8.86	< .025							0.22			7.26	
890	7/26/2011	Pipe From Well	Valley		3.47	< .025							26			7.85	
891	7/26/2011	Outside Spigot	Upland		5.28	< .025							1.6			6.22	
892	7/26/2011	Outside Spigot	Valley		5.87	< .025							2			6.52	
893	7/26/2011	Outside Spigot	Upland		6.43	< .025							< .1			6.75	
894	7/26/2011	Kitchen Sink	Valley		6.92	< .025							0.15			6.7	
895	12/15/2009	Pressure Tank	Upland					0.109		8.61	< .025		< .1				
896	7/26/2011	Outside Spigot	Upland		6.24	< .025							0.14			6.67	
897	7/26/2011	Kitchen Sink	Valley		5.99	< .025							0.1			7.05	
898	7/26/2011	Outside Spigot	Upland		6.68	< .025							0.19			6.92	
899	7/26/2011	Kitchen Sink	Upland		10.33	< .025							0.18			6.93	
900	7/26/2011	Kitchen Sink	Upland		8.47	< .025							0.19			6.94	
901	7/27/2011	Kitchen Sink	Upland		6.21	< .025							< .1			7.26	
902	7/27/2011	Outside Faucet	Valley		3.9	< .025							1.1			7.58	
903	7/27/2011	Basement Spigot	Valley		9.33	< .025							0.26			6.89	
904	12/17/2009	Pressure Tank	Valley	Yes	2.22	0.48		0.076		4.49	0.09		6500			7.87	
905	7/27/2011	Kitchen Sink	Valley		5.16	< .025							2.2			7.72	
906	7/27/2011	Kitchen Sink	Valley		7.14	< .025							0.12			6.46	
907	7/27/2011	Kitchen Sink	Upland		9.13	< .025							0.22			7.75	
908	7/27/2011	Pipe Overflow	Valley		8.08	0.028							1.2			6.55	
909	7/27/2011	Pressure Tank	Upland		8.34	< .025							0.17			6.94	
910	7/27/2011	Kitchen Sink	Upland		9.68	< .025							0.2			6.61	
911	7/27/2011	Kitchen Sink	Upland		8.28	< .025							0.28			6.63	
912	7/27/2011	Outside Spigot	Valley		6.45	< .025							1			7.68	
913	7/27/2011	Kitchen Sink	Valley		9.43	< .025							0.1			8.2	

Table S.8. Groundwater Quality Data for 1701 "Pre-Drill" Water Well Samples from Susquehanna County, Pennsylvania

Water Well ID	Sample Date	Sampling Port	Topo-graphic Location	Gas Extraction Area (Within 1 km of Active Gas Well)	Dissolved Oxygen - Field (mg/L)	Ethane ug/L	Hardness (mg/L)	Iron (mg/L)	Lead (mg/L)	Magnesium (mg/L)	Manganese (mg/L)	Mercury (mg/L)	Methane ug/L	Nitrate as N (mg/L)	Oil Grease (mg/L)	pH - Field (SU)	Potassium (mg/L)
914	7/27/2011	Kitchen Sink	Upland		8.42	< .025							0.38			7.82	
915	7/27/2011	Kitchen Sink	Upland		4.22	< .025							1.1			7.74	
916	7/28/2011	Kitchen Sink	Upland		4.65	< .025							0.15			7.66	
917	7/28/2011	Kitchen Sink	Valley		13.86	< .025							0.26			7.26	
918	7/28/2011	Kitchen Sink	Upland		12.15	< .025							0.35			6.85	
919	7/28/2011	Kitchen Sink	Upland		11.11	< .025							0.21			6.54	
920	7/28/2011	Kitchen Sink	Valley		17.7	< .025							0.31			7.2	
921	7/28/2011	Outside Spigot	Valley	Yes	5.96	2.2							11000			6.74	
922	7/29/2011	Kitchen Sink	Valley		6.86	< .025							0.33			7.76	
923	12/14/2009	Basement At Pressure Tank	Valley	Yes				< .05		8.42	< .025		0.13				
924	12/17/2009	Kitchen Sink	Valley		4.65	< .025		< .05		9.18	< .025		0.5			7.83	
925	12/13/2009	Pressure Tank	Valley					0.128		2.17	< .025		0.93				
926	7/29/2011	Kitchen Sink	Valley		5.32	< .025							0.52			7.63	
927	7/29/2011	Bathroom Sink	Valley		5.77	< .025							0.16			7.63	
928	7/29/2011	Kitchen Sink	Upland		6.77	< .025							75			8	
929	7/29/2011	Spigot On Side Of House	Upland		15.93	< .025							0.14			7.06	
930	7/29/2011	Bathroom Sink	Upland		6.86	< .025							0.44			7.5	
931	12/21/2009	Pressure Tank	Valley	Yes	3.29	37		0.375		4.8	0.101		43000			8.02	
932	7/29/2011	Spigot On Side Of House	Upland		4.7	< .025							0.18			7.85	
933	7/30/2011	Kitchen Sink	Valley		2.55	0.34							390			8.19	
934	7/30/2011	Outside Spigot	Valley		7.66	< .025							0.11			6.99	
935	7/30/2011	Kitchen Sink	Upland		3.31	< .025							0.22			7.23	
936	7/30/2011	Basement Sink	Upland		8.23	0.026							74			6.49	
937	7/30/2011	Outside Spigot	Valley		5.21	< .025							2.8			6.9	

Table S.8. Groundwater Quality Data for 1701 "Pre-Drill" Water Well Samples from Susquehanna County, Pennsylvania

Water Well ID	Sample Date	Sampling Port	Topo-graphic Location	Gas Extraction Area (Within 1 km of Active Gas Well)	Dissolved Oxygen - Field (mg/L)	Ethane ug/L	Hardness (mg/L)	Iron (mg/L)	Lead (mg/L)	Magnesium (mg/L)	Manganese (mg/L)	Mercury (mg/L)	Methane ug/L	Nitrate as N (mg/L)	Oil Grease (mg/L)	pH - Field (SU)	Potassium (mg/L)
938	7/30/2011	Presssure Tank	Valley		2.21	2.8							11000			8.31	
939	7/30/2011	Kitchen Sink	Upland		4.95	< .025							2.8			7.39	
940	7/30/2011	Kitchen Sink	Upland		6.92	< .025							0.12			6.5	
941	8/1/2011	Outside Spigot	Valley		3.46	< .025							0.1			6.09	
942	8/1/2011	Kitchen Sink	Upland		4.37	< .025							1.6			7.06	
943	8/1/2011	Kitchen Sink	Valley		2.68	0.62							1800			7.31	
944	8/1/2011	Kitchen Sink	Valley		8.47	< .025							0.11			6.7	
945	8/1/2011	Pressure Tank	Upland	Yes	7.68	< .025							0.59			6.5	
946	8/1/2011	Outside Spigot	Valley		11.13	< .025							1.1			6.72	
947	12/21/2009	Pressure Tank-Tenants House	Upland		6.2	< .025		< .05		4.37	< .025		2.9			7.52	
948	8/1/2011	Kitchen Sink	Upland		8.8	< .025							0.32			7.03	
949	8/1/2011	Spigot On Garage	Valley		11.11	< .025							0.45			6.28	
950	8/1/2011	Spigot On South Side Of House	Valley		8.3	< .025							0.17			6.58	
951	8/1/2011	Shop Sink	Upland		3.62	< .025							1.9			6.94	
952	8/1/2011	Pressure Tank	Valley		11.14	< .025							0.16			6.84	
953	8/2/2011	Kitchen Sink	Upland		11.02	< .025							< .1			6.53	
954	8/2/2011	Kitchen Sink	Valley			< .025							0.1				
955	8/2/2011	Kitchen Sink	Valley			< .025							0.14				
956	8/2/2011	Kitchen Sink	Valley		5.22	< .025							< .1			6.77	
957	8/2/2011	Kitchen Sink	Upland		8.08	< .025							0.69			6.97	
958	8/2/2011	Pressure Tank	Valley		11.96	< .025							0.1			6.37	
959	8/2/2011	Kitchen Sink	Upland		3.82	< .025							0.18			6.97	
960	8/2/2011	Kitchen Sink	Upland		3.25	< .025							0.68			7.09	

Table S.8. Groundwater Quality Data for 1701 "Pre-Drill" Water Well Samples from Susquehanna County, Pennsylvania

Water Well ID	Sample Date	Sampling Port	Topo-graphic Location	Gas Extraction Area (Within 1 km of Active Gas Well)	Dissolved Oxygen - Field (mg/L)	Ethane ug/L	Hardness (mg/L)	Iron (mg/L)	Lead (mg/L)	Magnesium (mg/L)	Manganese (mg/L)	Mercury (mg/L)	Methane ug/L	Nitrate as N (mg/L)	Oil Grease (mg/L)	pH - Field (SU)	Potassium (mg/L)
961	8/2/2011	Spigot Across The Road From Office Building	Valley			2.3							12000				
962	8/2/2011	Kitchen Sink	Upland		6.87	< .025							6.8			7.22	
963	8/5/2011	Kitchen Sink	Valley		6.73	< .025							0.25			7.73	
964	8/5/2011	Kitchen Sink	Valley		2.93	5.4							16000			7.77	
965	8/5/2011	Spigot On Side Of House	Valley			<0.025							0.2				
966	8/5/2011	Spigot In Shed Behind House	Upland		6.13	< .025							1.4			7.42	
967	8/5/2011	Pressure Tank	Valley		2.36	0.22							900			8.02	
968	8/5/2011	Kitchen Sink	Upland	Yes	5.6	< .025							0.13			6.99	
969	8/5/2011	Outside Spigot	Valley		4.82	3.6							13000			8.33	
970	12/15/2009	Pressure Tank	Upland	Yes	8.16	< .025		0.478		4.09	< .025		0.54			7.01	
971	8/5/2011	Kitchen Sink	Upland		6.06	< .025							0.46			7.06	
972	8/5/2011	Kitchen Sink	Upland		6.35	< .025							< .1			7	
973	8/5/2011	Outside Spigot	Valley		2.48	< .025							27			7.78	
974	8/5/2011	Outside Spigot	Valley		5.61	0.4							4600			7.05	
975	8/5/2011	Spigot On Side Of House	Upland		6.75	< .025							0.24			6.53	
976	8/5/2011	Spigot On Side Of House	Upland		5.08	< .025							0.14			7.86	
977	8/5/2011	Kitchen Sink	Upland		5.02	< .025							0.1			8.03	
978	8/5/2011	Spigot On Side Of House	Upland		7.21	0.1							26			7.92	
979	8/5/2011	Well	Upland		12.64	< .025							0.23			6.76	
980	8/5/2011	Pressure Tank	Upland		10.34	< .025							0.4			6.69	
981	8/5/2011	Kitchen Sink	Upland		2.75	< .025							1.5			6.79	

Table S.8. Groundwater Quality Data for 1701 "Pre-Drill" Water Well Samples from Susquehanna County, Pennsylvania

Water Well ID	Sample Date	Sampling Port	Topo-graphic Location	Gas Extraction Area (Within 1 km of Active Gas Well)	Dissolved Oxygen - Field (mg/L)	Ethane ug/L	Hardness (mg/L)	Iron (mg/L)	Lead (mg/L)	Magnesium (mg/L)	Manganese (mg/L)	Mercury (mg/L)	Methane ug/L	Nitrate as N (mg/L)	Oil Grease (mg/L)	pH - Field (SU)	Potassium (mg/L)
982	8/5/2011	Kitchen Faucet	Valley		5.57	0.62							3400			7.86	
983	1/4/2010	Well	Valley	Yes	1.69	9.4		< .05		4.3	< .025		24000			8.36	
984	8/5/2011	Outside Faucet	Valley		5.28	< .025							570			6.23	
985	8/5/2011	Kitchen Sink	Upland		3.46	< .025							0.1			7.34	
986	8/8/2011	Pressure Tank	Valley		4.9	< .025							9.8			7.62	
987	8/8/2011	Kitchen Sink	Valley		4.48	< .025							29			7.45	
988	8/8/2011	Kitchen Sink	Valley		4.2	< .025							0.1			7.9	
989	8/8/2011		Upland			< .025							< .1				
990	8/8/2011	Kitchen Sink	Valley		4.81	1.4							4000			7.83	
991	8/8/2011	Kitchen Sink	Upland			<0.025							<0.1				
992	8/8/2011	Kitchen Sink	Upland		4.35	< .025							0.96			7.7	
993	8/8/2011	Outside Spigot	Upland		5.72	0.036							240			7.96	
994	8/8/2011	Kitchen Sink	Valley		8.01	< .025							< .1			7.8	
995	8/8/2011	Kitchen Sink	Valley		1.9	0.7							6300			7.34	
996	8/8/2011	Spigoton Side Of House	Upland		4.57	< .025							0.13			7.6	
997	8/8/2011	Sink In Garage	Upland		5.9	< .025							< .1			6.98	
998	8/8/2011	Kitchen Sink	Upland		7.73	< .025							0.16			7.75	
999	8/8/2011		Valley			< .025							0.44				
1000	8/8/2011	Outside Spigot	Upland		4.96	< .025							1.5			7.59	
1001	8/9/2011	Basement Sink	Upland		8.04	< .025							< .1			7.28	
1002	8/9/2011	Kitchen Sink	Valley		4.78	1							4200			7.68	
1003	8/9/2011	Pressure Tank	Valley		5.12	< .025							0.12			7.89	
1004	8/9/2011	Pressure Tank	Valley		4.12	< .025							0.16			6.82	
1005	8/9/2011	Kitchen Sink	Valley		8.59	< .025							0.16			7.1	
1006	8/9/2011	Outside Spigot	Valley		4.41	< .025							0.22			7.87	
1007	12/14/2009	Basement At Pressure Tank	Valley		3.46	0.041		0.338		8.59	0.268		440			8.1	

Table S.8. Groundwater Quality Data for 1701 "Pre-Drill" Water Well Samples from Susquehanna County, Pennsylvania

Water Well ID	Sample Date	Sampling Port	Topo-graphic Location	Gas Extraction Area (Within 1 km of Active Gas Well)	Dissolved Oxygen - Field (mg/L)	Ethane ug/L	Hardness (mg/L)	Iron (mg/L)	Lead (mg/L)	Magnesium (mg/L)	Manganese (mg/L)	Mercury (mg/L)	Methane ug/L	Nitrate as N (mg/L)	Oil Grease (mg/L)	pH - Field (SU)	Potassium (mg/L)
1008	8/9/2011	Bathroom Sink	Valley		8.3	< .025							0.14			6.4	
1009	8/9/2011	Outside Spigot	Valley		7.52	1							8000			7.92	
1010	8/9/2011	Kitchen Sink	Upland		8.64	< .025							0.14			6.68	
1011	8/9/2011	Kitchen Sink	Upland		7.24	< .025							< .1			6.71	
1012	8/9/2011	Kitchen Sink	Upland		1.54	< .025							88			7.48	
1013	8/9/2011	Kitchen Sink	Valley		6.58	< .025							< .1			7.25	
1014	8/9/2011	Kitchen Sink	Upland		2.02	< .025							0.25			7.75	
1015	8/9/2011	Kitchen Sink	Upland		5.44	< .025							< .1			7.54	
1016	8/9/2011	Kitchen Sink	Upland		3.04	< .025							0.1			7.84	
1017	7/11/2011	Spigot On Side Of House	Upland		6.95	< .025							0.4			4.77	
1018	7/12/2011	Bathroom Sink	Valley		2.12	0.33							2100			7.81	
1019	7/12/2011	Sink In Rear Of Far Building	Valley		5.35	< .025							4.1			7.43	
1020	7/21/2011	Basement Sink	Upland		9.56	< .025							0.17			7.88	
1021	7/22/2011	Kitchen Faucet	Upland		14.01	0.025							2			7.6	
1022	7/25/2011	Spigot Outside Barn	Valley		3.14	1.4							4900			8.64	
1023	7/25/2011	Kitchen Sink	Upland		7.82	< .025							0.7			7.58	
1024	7/26/2011	Outside Spigot	Upland		3.72	< .025							< .1			6.53	
1025	7/28/2011		Valley			<0.025							64				
1026	8/1/2011	Kitchen Sink	Valley		6.14	< .025							< .1			7.71	
1027	8/1/2011	Kitchen Sink	Valley		5.76	< .025							0.14			7.83	
1028	8/1/2011	Outside Spigot	Upland		2.53	< .025							0.88			7.67	
1029	8/2/2011	Pipe Overflow	Valley		12.55	< .025							< .1			6.47	
1030	8/2/2011	Kitchen Faucet	Valley			< .025							0.82				

Table S.8. Groundwater Quality Data for 1701 "Pre-Drill" Water Well Samples from Susquehanna County, Pennsylvania

Water Well ID	Sample Date	Sampling Port	Topo-graphic Location	Gas Extraction Area (Within 1 km of Active Gas Well)	Dissolved Oxygen - Field (mg/L)	Ethane ug/L	Hardness (mg/L)	Iron (mg/L)	Lead (mg/L)	Magnesium (mg/L)	Manganese (mg/L)	Mercury (mg/L)	Methane ug/L	Nitrate as N (mg/L)	Oil Grease (mg/L)	pH - Field (SU)	Potassium (mg/L)
1031	8/3/2011	Garage Building Next To House	Valley		2.48	0.033							140			6.99	
1032	8/3/2011	Kitchen Sink	Upland		3.91	< .025							27			7.75	
1033	8/3/2011	Outside Faucet	Upland		3.78	< .025							13			7.92	
1034	8/3/2011	Kitchen Faucet	Valley			0.036							1.2				
1035	12/15/2009	Well House	Valley	Yes				< .05		2.37	< .025		15000				
1036	8/3/2011	Pressure Tank	Upland		6.14	< .025							0.63			7.46	
1037	8/3/2011	Pressure Tank	Upland		7.09	< .025							0.5			7.89	
1038	8/3/2011	Kitchen Sink	Valley		5.31	< .025							0.12			6.61	
1039	8/3/2011	Outside Faucet	Valley	Yes	4.87	< .025							< .1			7.68	
1040	8/3/2011	Outside Faucet	Valley		3.78	< .025							1			7.57	
1041	8/3/2011	Outside Faucet	Upland	Yes	6.65	< .025							0.12			7.51	
1042	8/3/2011	Kitchen Sink	Upland		11.93	< .025							0.28			6.46	
1043	8/3/2011	Pressure Tank	Valley		7.05	< .025							3.3			6.77	
1044	1/3/2010	Pressure Tank	Valley		7.02	< .025		0.14		3.77	< .025		3.2			6.87	
1045	8/3/2011	Spigot	Valley		4.51	0.042							1.6			6.77	
1046	8/3/2011	Barn Sink	Valley		4.04	< .025							0.22			6.77	
1047	8/3/2011	Kitchen Sink	Valley		1.76	0.097							930			7.19	
1048	8/3/2011	Kitchen Sink	Valley		2.32	< .025							2.5			7.9	
1049	8/3/2011	Outside Spigot	Valley		6.59	< .025							0.31			7.75	
1050	8/3/2011	Kitchen Sink	Upland		5.54	< .025							< .1			6.8	
1051	8/3/2011	Kitchen Sink	Upland		9.31	< .025							0.27			6.38	
1052	8/3/2011	Kitchen Sink	Upland		5.24	< .025							0.3			7.34	
1053	8/3/2011	Kitchen Sink	Valley		5.69	< .025							< .1			7.11	
1054	8/3/2011	Pressure Tank In Basement	Upland		5.46	< .025							0.14			7.07	
1055	8/3/2011	Kitchen Sink	Valley		7.74	<0.025							0.2			7.1	

Table S.8. Groundwater Quality Data for 1701 "Pre-Drill" Water Well Samples from Susquehanna County, Pennsylvania

Water Well ID	Sample Date	Sampling Port	Topo-graphic Location	Gas Extraction Area (Within 1 km of Active Gas Well)	Dissolved Oxygen - Field (mg/L)	Ethane ug/L	Hardness (mg/L)	Iron (mg/L)	Lead (mg/L)	Magnesium (mg/L)	Manganese (mg/L)	Mercury (mg/L)	Methane ug/L	Nitrate as N (mg/L)	Oil Grease (mg/L)	pH - Field (SU)	Potassium (mg/L)
1056	8/3/2011	Kitchen Sink	Upland		5.37	< .025							0.17			7.03	
1057	8/4/2011	Kitchen Sink	Valley		4.87	< .025							0.15			7.17	
1058	8/4/2011	Outside Spigot	Upland		6.27	< .025							< .1			6.84	
1059	8/4/2011	Outside Spigot	Upland		6.56	< .025							< .1			7.9	
1060	12/21/2009	Laundry Sink	Upland		4.13	0.056		< .05		< 1	< .025		5			8.53	
1061	8/4/2011	Spigot On Side Of House	Upland		4.8	< .025							0.86			7.86	
1062	8/4/2011	Spigot Behind House	Valley		3.89	3.4							15000			7.93	
1063	8/4/2011	Kitchen Sink	Valley		3.05	< .025							81			7.96	
1064	8/4/2011	Kitchen Sink	Valley		8.39	< .025							< .1			6.69	
1065	8/4/2011	Kitchen Sink	Valley		5.68	< .025							0.56			7.79	
1066	8/4/2011	Outside Spigot	Upland		5.05	< .025							< .1			7.17	
1067	8/4/2011	Kitchen Sink	Upland		5	< .025							< .1			6.74	
1068	8/4/2011	Spigot In Rear Of Building	Valley	Yes	7.79	< .025							9.3			6.53	
1069	4/4/2009	Bathroom Sink First Floor	Valley		6.9	< .025		< .025		2.21	< .025		2.7		< 5	6.95	
1070	12/28/2009	Kitchen Sink	Upland		4.87	< .025		< .05		4.92	< .025		0.28			7.79	
1071	8/4/2011	Pressure Tank	Valley	Yes	4.23	0.46							1200			8.07	
1072	8/4/2011	Kitchen Sink	Valley	Yes	6.01	< .025							0.23			7.14	
1073	8/4/2011	Kitchen Sink	Valley	Yes	3.13	1.6							4600			8.14	
1074	8/4/2011	Spigot In Warehouse	Upland		3.82	< .025							0.12			7.62	
1075	8/4/2011	Kitchen Sink	Upland		14.32	< .025							0.18			6.01	
1076	8/4/2011	Kitchen Sink	Upland		8.45	< .025							0.12			6.69	
1077	8/4/2011	Kitchen Sink	Valley		6.53	< .025							0.1			7.1	

Table S.8. Groundwater Quality Data for 1701 "Pre-Drill" Water Well Samples from Susquehanna County, Pennsylvania

Water Well ID	Sample Date	Sampling Port	Topo-graphic Location	Gas Extraction Area (Within 1 km of Active Gas Well)	Dissolved Oxygen - Field (mg/L)	Ethane ug/L	Hardness (mg/L)	Iron (mg/L)	Lead (mg/L)	Magnesium (mg/L)	Manganese (mg/L)	Mercury (mg/L)	Methane ug/L	Nitrate as N (mg/L)	Oil Grease (mg/L)	pH - Field (SU)	Potassium (mg/L)
1078	1/10/2010	Little Farm House Basement Pressure Tank	Upland		9.24	< .025		< .05		6.27	< .025		0.45			6.44	
1079	8/4/2011	Kitchen Sink	Upland		14.86	< .025							0.34			7.37	
1080	8/4/2011	Spigot On Side Of House	Valley		4.73	< .025							0.21			7.77	
1081	8/4/2011	Kitchen Sink	Upland		6.41	< .025							0.17			7.82	
1082	8/4/2011	Kitchen Sink	Valley		7.69	< .025							0.28			7.5	
1083	7/11/2011	Outside Hose Bib	Upland	Yes		<26	68	0.11	<0.001	2.6	0.0022	<0.0002	<26	3.3	<4.7		1.4
1084	7/11/2011	Basement At Pressure Tank	Upland	Yes	4.84	< 26	26	< .05	< .001	1.1	0.0076	< .0002	< 26	0.9		8.93	1.2
1085	12/21/2009		Valley	Yes		< .025		< .05		1.91	< .025		20				
1086	7/11/2011	Bathroom Sink First Floor	Valley	Yes	6.32	< 26	82	< .05	0.0029	3.4	0.0026	< .0002	< 26	0.84	< 4.7	7.62	0.72
1087	7/12/2011	Outside Hose Bib	Upland	Yes	0.17	< 26	84	< .05	< .001	4.3	0.0081	< .0002	< 26	< .05	< 4.7	7.51	0.86
1088	7/12/2011	Kitchen Sink	Valley	Yes	1.74	< 26	110	< .05	< .001	7.3	0.0014	< .0002	< 26	0.36	< 4.7	7.67	1.1
1089	7/12/2011	Pressure Tank	Valley	Yes	0.21	< 26	92	0.78	< .001	6.1	0.035	< .0002	< 26	< .05	< 4.7	7.98	1
1090	12/21/2009	Pressure Tank	Valley	Yes	1.5	5.2		0.164		16.4	0.314		22000			7.83	
1091	7/13/2011	Pressure Tank In Basement	Upland		7.42	< 26	120	0.099	0.0011	5.6	0.046	< .0002	< 26	< .05		7.96	2
1092	7/13/2011	Well	Valley		6.04	< 26	100	< .05	< .001	4.7	0.0014	< .0002	< 26	< .05		7.89	0.97
1093	7/13/2011	Outside Hose Bib	Upland	Yes	4.41	< 26	84	0.16	0.0014	3.4	0.013	< .0002	< 26	1.5	< 4.7	7.1	0.84
1094	12/14/2009	Pressure Tank In Basement	Valley	Yes				0.688		7.58	0.118		4.3				
1095	7/13/2011	Well	Valley	Yes	2.86	< 26	96	0.058	< .001	4.9	0.0011	< .0002	< 26	< .05	< 4.7	6.82	0.88
1096	7/13/2011	Pressure Tank	Valley	Yes	7.7	< 26	54	0.2	0.0042	2.1	0.0082	< .0002	< 26	1.6		6.96	0.61
1097	1/3/2010	Spigot Under House	Upland	Yes	7.16	< .025		< .05		3.99	< .025		6.5			6.95	
1098	12/17/2009	Pressure Tank	Upland		10.09			< .05		9.07	< .025		7			7.43	

Table S.8. Groundwater Quality Data for 1701 "Pre-Drill" Water Well Samples from Susquehanna County, Pennsylvania

Water Well ID	Sample Date	Sampling Port	Topo-graphic Location	Gas Extraction Area (Within 1 km of Active Gas Well)	Dissolved Oxygen - Field (mg/L)	Ethane ug/L	Hardness (mg/L)	Iron (mg/L)	Lead (mg/L)	Magnesium (mg/L)	Manganese (mg/L)	Mercury (mg/L)	Methane ug/L	Nitrate as N (mg/L)	Oil Grease (mg/L)	pH - Field (SU)	Potassium (mg/L)
1099	1/5/2010	Kitchen Sink	Valley	Yes	6.75	4.6		0.094		13.5	0.068		23000			7.51	
1100	7/18/2011	Outside Faucet	Valley	Yes		< 26	57	< .05	< .001	2.3	0.002	< .0002	< 26	5.7	< 4.7		1.7
1101	12/13/2009		Upland					0.082		4.66	< .025		0.13				
1102	7/19/2011	Inside Faucet	Valley	Yes	1.64	< 26	110	0.22	0.0012	5.1	0.0076	< .0002	< 26	< .05	< 4.7	7.62	1.1
1103	7/20/2011	Kitchen Sink Cold Water Tap	Upland		8.19	< 26	40	< .05	< .001	2.1	< .0005	< .0002	< 26	0.33		6.71	0.69
1104	7/20/2011	Pressure Tank	Upland		3.8	< 26	120	< .05	< .001	5.6	< .0005	< .0002	< 26	0.55		7.08	1.4
1105	12/28/2009	Pressure Tank	Upland		2.27			< .05		5.55	0.063		3.8			7.57	
1106	7/20/2011	Frost Free Hydrant 2 Feet From Well Head	Upland		5.5	< 26	83	< .05	< .001	4.1	0.0012	< .0002	< 26	0.082		7.76	1.4
1107	7/20/2011	Inside Faucet	Upland		3.19	< 26	100	< .05	< .001	4.7	0.0031	< .0002	< 26	0.4		7.85	1.1
1108	12/13/2009	Pressure Tank	Upland					0.071		4.04	< .025		7.5				
1109	12/28/2009	Pressure Tank	Upland		11.98	4.9		< .05		< 1	< .025		2000			9.83	
1110	7/26/2011	Pressure Tank In Gun Shop	Valley		1.85	< 26	58	< .05	< .001	5.2	0.059	< .0002	1370	< .05	< 5.2	8.03	1.5
1111	12/16/2009	Kitchen Sink	Valley	Yes	8.82	< .025		< .05		2.1	< .025		< .1			6.47	
1112	7/26/2011	Inside Faucet	Valley		2.21	< 26	72	< .05	< .001	4.2	0.012	< .0002	< 26	0.12		8.02	1.8
1113	7/27/2011	Outside Spigot Near Large Rock Slabs	Valley		1.84	< 26	43	< .05	< .001	3.9	0.019	< .0002	213	< .05	< 5.6	8.35	1.8
1114	12/13/2009	Pressure Tank	Upland	Yes	13.14	< .025		< .05		5.26	< .025		0.31			7.75	
1115	7/27/2011	Outside Faucet	Upland		8.15	< 26	96	0.076	< .001	4.2	0.0019	< .0002	< 26	1.1		7.41	1
1116	7/27/2011	Kitchen Sink Cold Water Tap	Valley	Yes	4.56	< 26	140	0.19	< .001	7.4	0.016	< .0002	< 26	0.068		7.63	1.1
1117	1/11/2010	Pressure Tank	Upland		5			< .05		3.43	0.034		140			8.03	
1118	7/27/2011	Kitchen Sink Faucet	Upland		8.23	< 26	62	< .05	< .001	2.5	0.001	< .0002	< 26	1.6		7.33	0.81
1119	7/27/2011	Outside Hose Bib	Upland		2.73	< 26	110	< .05	< .001	4.8	0.0011	< .0002	< 26	0.78		7.59	1.1
1120	7/28/2011	Kitchen Sink	Valley		0.77	< 26	97	0.085	< .001	6.7	0.26	< .0002	< 26	0.36		7.61	1.1

Table S.8. Groundwater Quality Data for 1701 "Pre-Drill" Water Well Samples from Susquehanna County, Pennsylvania

Water Well ID	Sample Date	Sampling Port	Topo-graphic Location	Gas Extraction Area (Within 1 km of Active Gas Well)	Dissolved Oxygen - Field (mg/L)	Ethane ug/L	Hardness (mg/L)	Iron (mg/L)	Lead (mg/L)	Magnesium (mg/L)	Manganese (mg/L)	Mercury (mg/L)	Methane ug/L	Nitrate as N (mg/L)	Oil Grease (mg/L)	pH - Field (SU)	Potassium (mg/L)
1121	7/28/2011	Inside Faucet	Valley		5.6	< 26	89	0.27	< .001	8.7	0.0048	< .0002	< 26	0.55	< 4.9	7.67	1.5
1122	7/28/2011	Outside Faucet	Valley		2.8	< 26	99	0.13	0.0011	9.2	0.063	< .0002	< 26	< .05	< 5	6.77	1.2
1123	7/29/2011	Pressure Tank	Upland	Yes	5.1	< 26	85	< .05	0.0016	5.7	0.0012	< .0002	< 26	2.7		6.17	1.3
1124	7/29/2011	Kitchen Sink	Valley		8.1	< 26	26	0.087	< .001	2.5	0.0012	< .0002	< 26	0.22		6.32	0.61
1125	7/29/2011	Pressure Tank	Valley		3.79	< 26	78	3.1	0.0025	7.5	0.02	< .0002	< 26	0.11	< 4.8	7.68	0.78
1126	8/1/2011	Outside Faucet	Upland		7.74	< 26	91	< .05	< .001	8.8	0.0038	< .0002	< 26	0.56	< 4.8	5.9	1.5
1127	8/1/2011	Outside Faucet	Valley		7.74	< 26	30	< .05	< .001	2	0.0059	< .0002	< 26	0.2	< 4.7	5.9	0.77
1128	8/1/2011	Pressure Tank At Basement	Upland	Yes	9.03	< 26	120	0.093	0.001	5.2	0.0017	< .0002	< 26	0.33		7.44	1.3
1129	8/2/2011		Upland	Yes		<26	110	1.3	<0.001	5.3	0.013	<0.0002	<26	1.7	<4.7		0.77
1130	12/28/2009	Kitchen Sink	Valley		7.62	< .025		< .05		2.96	< .025		0.51			7.01	
1131	8/3/2011	Kitchen Sink	Upland	Yes		<26	120	0.15	<.001	4.6	0.0068	<.0002	30.9	9.4			1.6
1132	8/3/2011	Pressure Tank	Valley		0.11	48.2	60	0.82	< .001	5	0.14	< .0002	20500	< .05		8.06	0.82
1133	10/22/2009		Upland	Yes				< .05		7.99	< .025		7.1				
1134	12/28/2009	Garage Sink	Upland		94.5	< .025		< .05		6.74	< .025		0.3			7.1	
1135	8/4/2011	Bathroom Sink Cwt On South End	Upland		2.95	< 26	71	0.14	< .001	5.1	0.0085	< .0002	< 26	0.52		7.66	0.82
1136	8/4/2011	Bailed Well	Upland	Yes	2.46	< 26	76	0.16	< .001	4.3	1.4	< .0002	< 26	< .05		6.55	6.6
1137	8/4/2011	Kitchen Sink	Upland	Yes	6.76	< 26	110	0.24	0.0049	4.1	0.032	< .0002	< 26	0.51	< 4.7	7.64	1.7
1138	8/4/2011	Pressure Tank	Valley	Yes	2.02	< 26	160	0.14	0.0011	12	0.004	< .0002	< 26	0.38		7.48	1.1
1139	5/19/2011		Valley			<26	67	<0.05	<0.001	7.2	0.097	<0.0002	110	<0.05			1.1
1140	7/25/2011	Kitchen Sink	Valley		1.99	< 26	100	< .05	0.0027	9.2	0.13	< .0002	< 26	< .05	< 5.1	7.88	1.3
1141	7/27/2011	Outside Spigot Under Back Deck	Valley		1.54	< 26	79	0.78	< .001	7.6	0.071	< .0002	160	< .05	< 4.7	7.84	0.89
1142	8/2/2011	Pressure Tank	Valley		7.75	< 26	77	0.084	< .001	7.6	0.0028	< .0002	< 26	0.34	< 4.8	7.29	0.94

Table S.8. Groundwater Quality Data for 1701 "Pre-Drill" Water Well Samples from Susquehanna County, Pennsylvania

Water Well ID	Sample Date	Sampling Port	Topo-graphic Location	Gas Extraction Area (Within 1 km of Active Gas Well)	Dissolved Oxygen - Field (mg/L)	Ethane ug/L	Hardness (mg/L)	Iron (mg/L)	Lead (mg/L)	Magnesium (mg/L)	Manganese (mg/L)	Mercury (mg/L)	Methane ug/L	Nitrate as N (mg/L)	Oil Grease (mg/L)	pH - Field (SU)	Potassium (mg/L)
1143	8/2/2011	Outside Faucet	Upland		5.7	< 26	69	< .05	< .001	6.3	< .0005	< .0002	< 26	0.83		6.62	0.89
1144	1/3/2010	Kitchen Sink	Upland	Yes	9.27	< .025		< .05		3.43	< .025		3.2			7.02	
1145	12/13/2009		Upland	Yes				0.082		6.76	< .025		2.7				
1146	1/10/2010	Spring House	Upland		11.66	< .025		< .05		6.97	< .025		0.7			7.93	
1147	1/5/2010	Pressure Tank	Upland		12.1	< .025		0.084		1.5	< .025		1.1			8.43	
1148	5/10/2011	Kitchen Sink	Valley		9.05	0.028	23.57168	< .05	0.013	< 1	< .0025	< .0002	21	< 1	< 5	8.24	2.95
1149	2/1/2010	Pressure Tank	Upland		9.08	< .025		< .05		2.48	< .025		0.11			6.57	
1150	8/20/2009		Valley					0.463		< 1	0.03		23000		< 5		
1151	12/6/2009	Washroom Sink	Valley		166.9	< .025		< .05		3.14	< .025		0.87			6.27	
1152	1/17/2010	Kitchen Sink	Valley		4.62			< .05		14	< .025		0.43			7.79	
1153	1/14/2010	Basement At Pressure Tank	Upland		9.62	< .025		< .05		8.28	< .025		2			7.95	
1154	1/17/2010	Kitchen Sink	Upland		5.5			< .05		8.58	< .025		0.35			7.56	
1155	1/17/2010	Pressure Tank	Valley		9.75	< .025		< .05		2.87	< .025		0.21			6.52	
1156	12/11/2010	Kitchen Sink	Valley		9.85	0.038	35.3174	<0.05	0.032	1.3	<0.0025	<0.0002	0.28	<1	<5	6.46	<1
1157	8/4/2011	Spigot Back Yard	Upland		6.5	2.4							11000			7.29	
1158	8/4/2011	Kitchen Sink	Valley		4.25	2.6							12000			6.54	
1159	8/4/2011	Pressure Tank	Valley		5.74	<0.025							22			6.51	
1160	8/10/2011		Upland			3.3	134.0594	< .05	0.0063	8.3	0.046	< .0002	5100	< 1	< 5		3.2
1161	8/10/2011	Kitchen Sink	Valley		7.29	< .025							1.5			7.93	
1162	8/10/2011	Kitchen Sink	Upland		7.92	< .025							< .1			7.34	
1163	8/10/2011	Spigot On Side Of House	Upland			<0.025							0.14				
1164	8/10/2011	Outside Spigot	Valley		1.37	< .025							5.2			7.85	
1165	8/10/2011	Kitchen Sink	Valley		1.28	7.3							22000			8.05	

Table S.8. Groundwater Quality Data for 1701 "Pre-Drill" Water Well Samples from Susquehanna County, Pennsylvania

Water Well ID	Sample Date	Sampling Port	Topo-graphic Location	Gas Extraction Area (Within 1 km of Active Gas Well)	Dissolved Oxygen - Field (mg/L)	Ethane ug/L	Hardness (mg/L)	Iron (mg/L)	Lead (mg/L)	Magnesium (mg/L)	Manganese (mg/L)	Mercury (mg/L)	Methane ug/L	Nitrate as N (mg/L)	Oil Grease (mg/L)	pH - Field (SU)	Potassium (mg/L)
1166	8/10/2011	Spigot On Side Of Shed	Valley		0.71	< .025							0.12			7.85	
1167	8/10/2011	Outside Spigot	Upland		5.61	<0.025							0.34			6.18	
1168	8/10/2011	Kitchen Sink	Upland		5.38	< .025							0.19			6.05	
1169	8/10/2011	Outside Spigot	Upland		8.61	< .025							8			7.84	
1170	8/10/2011	Kitchen Sink	Upland		4.57	< .025							< .1			7.92	
1171	8/10/2011	Dug Well	Upland			< .025							3.4				
1172	8/10/2011	Shop Sink	Upland		8.71	< .025							0.14			7.23	
1173	12/10/2009	Kitchen Sink	Valley	Yes				0.451		7.13	0.052		15				
1174	8/11/2011	Kitchen Sink	Upland		7.82	< .025							0.29			7.55	
1175	8/11/2011	Kitchen Sink	Upland		7.91	< .025							0.18			7.51	
1176	8/11/2011	Outside Spigot	Valley		8.21	0.046							300			8.39	
1177	8/11/2011	Kitchen Sink	Upland		7.12	< .025							0.32			7.14	
1178	8/14/2011	Outside Spigot	Valley		6.95	< .025							0.15			8.05	
1179	8/15/2011	Hand Dug Well	Upland		8.71	< .025							2			6.49	
1180	8/16/2011	Kitchen Sink	Upland		4.32	< .025							0.41			6.5	
1181	1/14/2010	Basement Pressure Tank	Valley		9.19	< .025		< .05		1.75	< .025		< .1			6.66	
1182	8/16/2011	Dug Well	Upland		5.55	< .025							6.6			6.34	
1183	8/16/2011	Kitchen Sink	Valley		7.88	< .025							0.11			7.67	
1184	2/1/2010	Elementary Boiler Room	Upland	Yes	7.42	< .025		< .05		9.4	< .025		8.8			7.92	
1185	8/17/2011	Kitchen Sink	Valley		7.8	< .025							0.15			7.71	
1186	8/19/2011	Kitchen Sink	Upland		5	< .025							0.72			7.45	
1187	8/19/2011	Kitchen Sink	Valley		5.88	< .025							0.24			6.07	
1188	8/29/2011	Outside Faucet	Valley		2.39	< 26	110	< .05	< .001	4.5	< .0005	< .0002	< 26	0.269	< 4.81	7.83	1.4
1189	1/14/2010	Basement At Pressure Tank	Valley		5.08	< .025		0.189		7.88	< .025		0.29			7.71	
1190	9/1/2011	Outside Faucet	Upland		0.64	< 5							< 5			7.96	
1191	9/1/2011	Hydrant	Upland		7.31	< 5							< 5			6.75	

Table S.8. Groundwater Quality Data for 1701 "Pre-Drill" Water Well Samples from Susquehanna County, Pennsylvania

Water Well ID	Sample Date	Sampling Port	Topo-graphic Location	Gas Extraction Area (Within 1 km of Active Gas Well)	Dissolved Oxygen - Field (mg/L)	Ethane ug/L	Hardness (mg/L)	Iron (mg/L)	Lead (mg/L)	Magnesium (mg/L)	Manganese (mg/L)	Mercury (mg/L)	Methane ug/L	Nitrate as N (mg/L)	Oil Grease (mg/L)	pH - Field (SU)	Potassium (mg/L)
1192	8/2/2011	Pressure Tank	Upland	Yes	0.13	< 26	1.5	0.24	0.0012	< .1	0.0086	< .0002	52.1	< .05		9.85	0.68
1193	1/17/2010	Garage Sink	Upland		5.95	< .025		< .05		8.4	< .025		0.19			7.71	
1194	8/10/2011	Outside Faucet	Valley	Yes	5.77	< 26	110	1	< .001	4.9	4.2	< .0002	< 26	< .05		7.48	1.3
1195	8/10/2011	Inside Faucet	Upland	Yes	1.69	< 26	82	0.092	< .001	3.7	0.058	< .0002	372	< .05		7.76	2.2
1196	1/13/2010	Kitchen Sink	Upland					< .05		9.14	< .025		0.84				
1197	2/3/2010	Sink In Milkhouse Barn	Upland	Yes	3.47	< .025		0.113		7.86	< .025		0.26			7.34	
1198	8/15/2011	Outside Faucet	Upland	Yes	4.11	< 26	120	< .05	< .001	6.9	0.00062	< .0002	< 26	0.28		7.39	1.3
1199	1/12/2010	Pressure Tank	Upland					0.098		3.74	< .025		0.43				
1200	1/18/2010	Kitchen Sink	Upland		10.24	< .025		< .05		2.64	< .025		0.12			6.57	
1201	8/22/2011	Barn Spigot	Upland		5.17	< .025							0.18			6.58	
1202	8/22/2011	Kitchen Sink	Valley		4.75	< .025							0.13			7.36	
1203	8/22/2011	Kitchen Sink	Upland		5.31	< .025							0.31			7.44	
1204	8/22/2011	Kitchen Sink	Valley		7.59	< .025							1.4			7.09	
1205	8/22/2011	Kitchen Sink	Upland		5.36	< .025							< .1			7.82	
1206	8/23/2011	Outside Spigot	Valley		5.73	< .025							2			7.21	
1207	1/23/2010	Basement At Pressure Tank	Upland		6.97			< .05		7.54	< .025		0.28			7.8	
1208	1/13/2010		Valley					< .05		3.57	< .025		18000				
1209	6/23/2011	Kitchen Sink	Valley	Yes	7.29	< 26	97	< .05	< .001	6.5	0.017	< .0002	< 26	0.13		7.25	1.3
1210	1/13/2010	Kitchen Sink	Upland					< .05		5.7	< .025		< .1				
1211	8/5/2011	Inside Faucet	Upland	Yes	3.25	< 26	94	< .05	< .001	6.3	0.056	< .0002	< 26	0.69		7.95	1.1
1212	8/12/2011	Pressure Tank	Valley			<26	92	<0.05	0.0037	8.5	0.0033	<0.0002	<26	<0.05	<4.8		1.1
1213	12/6/2009	Kitchen Sink	Upland		4.97	< .025		< .05		10.6	< .025		3.4			7.02	
1214	8/12/2011	Inside Faucet	Upland		4.74	< 26	77	0.077	0.0015	3.1	0.028	< .0002	< 26	0.65		7.47	1.1
1215	8/21/2011	Outside Faucet	Upland		0.41	< 26	130	8.5	0.0038	7.4	2.8	< .0002	< 26	0.38		7.79	2.2
1216	8/23/2011	Inside Faucet	Upland		6.6	< 26	62	< .05	< .001	2.8	< .0005	< .0002	< 26	0.16		7.85	1.3

Table S.8. Groundwater Quality Data for 1701 "Pre-Drill" Water Well Samples from Susquehanna County, Pennsylvania

Water Well ID	Sample Date	Sampling Port	Topo-graphic Location	Gas Extraction Area (Within 1 km of Active Gas Well)	Dissolved Oxygen - Field (mg/L)	Ethane ug/L	Hardness (mg/L)	Iron (mg/L)	Lead (mg/L)	Magnesium (mg/L)	Manganese (mg/L)	Mercury (mg/L)	Methane ug/L	Nitrate as N (mg/L)	Oil Grease (mg/L)	pH - Field (SU)	Potassium (mg/L)
1217	8/23/2011	Inside Faucet	Upland	Yes	4.12	< 26	53	< .05	< .001	2.2	< .0005	< .0002	< 26	0.12		7.45	2.2
1218	8/24/2011	Pressure Tank	Upland	Yes	9.9	< 26	43	2	0.011	2.5	0.043	< .0002	< 26	0.59		7.8	1.4
1219	8/24/2011	Inside Faucet	Valley		1.62	< 26	100	0.32	< .001	5.2	0.0058	< .0002	< 26	0.19		7.65	0.84
1220	1/18/2010	Basement Pressure Tank	Upland		9.86	< .025		0.301		6.74	< .025		0.31			7.19	
1221	8/25/2011	Inside Faucet	Upland		3.37	< 5	96	0.12	< .001	5.2	0.0056	< .0002	< 5	0.22		6.96	1.1
1222	8/26/2011	Well	Upland	Yes		< 5	80	7.1	0.0042	4.4	4.2	< .0002	227	< .05			3.5
1223	8/29/2011	Kitchen Sink	Upland		3.98	< 5	130	0.07	< .001	7.5	0.00084	< .0002	< 5	0.14		7.36	1.9
1224	1/11/2010	Kitchen Sink	Upland		6.22	< .025		0.411		7.97	< .025		2.7			7.58	
1225	1/18/2010	Pressure Tank In Basement	Valley	Yes	3.21	< .025		< .05		3.55	< .025		0.45			7.68	
1226	1/24/2010	Pressure Tank	Upland	Yes	2	< .025		0.171		7.56	0.109		1.5			7.7	
1227	8/30/2011	Pressure Tank	Upland		5.39	< 5	99	0.21	0.0015	5.4	0.041	< .0002	< 5	0.31		7.64	1.3
1228	8/30/2011	Outside Hose Bib	Valley		0.05	< 5	110	< .05	< .001	5.2	0.015	< .0002	6.94	0.2		7.78	1.6
1229	8/10/2011	Pressure Tank Before Treatment	Valley		2.39	< 26	38	0.074	< .001	2.1	0.012	< .0002	13000	< .05		8.77	2
1230	1/10/2010	Little House By Pond Spring	Valley		7.69	< .025		< .05		6.48	< .025		0.28			6.67	
1231	1/10/2010	Pressure Tank	Valley		6.39	0.48		0.152		7.23	0.085		2600			8.18	
1232	1/16/2010	Basement Pressure Tank	Upland		8.58	< .025		< .05		8.93	< .025		1.2			7.58	
1233	7/19/2011	Kitchen Sink	Upland		6.83	< .025							0.65			7.54	
1234	8/24/2011	Kitchen Sink	Valley		6.73	< .025							< .1			7.59	
1235	8/24/2011	Inside Faucet	Valley		8.62	< .025							< .1			7.93	
1236	1/16/2010	Basement At Pressure Tank	Upland		9.15	< .025		< .05		5.79	< .025		1			7.39	
1237	1/16/2010	Basement Pressure Tank	Upland		6.57	< .025		< .05		9.03	0.025		< .1			7.88	

Table S.8. Groundwater Quality Data for 1701 "Pre-Drill" Water Well Samples from Susquehanna County, Pennsylvania

Water Well ID	Sample Date	Sampling Port	Topo-graphic Location	Gas Extraction Area (Within 1 km of Active Gas Well)	Dissolved Oxygen - Field (mg/L)	Ethane ug/L	Hardness (mg/L)	Iron (mg/L)	Lead (mg/L)	Magnesium (mg/L)	Manganese (mg/L)	Mercury (mg/L)	Methane ug/L	Nitrate as N (mg/L)	Oil Grease (mg/L)	pH - Field (SU)	Potassium (mg/L)
1238	8/26/2011	Kitchen Sink	Valley		5.72	< .025							46			8.89	
1239	8/27/2011	Kitchen Sink	Upland		5.56	< .025							0.12			7.42	
1240	8/27/2011	Kitchen Sink	Valley		5.34	0.23							2000			7.92	
1241	1/21/2010	Kitchen Sink	Upland		7.16	< .25		< .05		6.09	< .025		0.2			7.68	
1242	8/27/2011	Kitchen Faucet	Valley			0.26							2100				
1243	1/23/2010	Pressre Tank In Barn	Upland	Yes	4.59			< .05		< 1	< .025		130			8.81	
1244	9/1/2011	Kitchen Sink	Valley		4.8	< .025							1.3			7.57	
1245	1/23/2010	Basement At Pressure Tank	Upland	Yes	5.52			< .05		1.45	< .025		50			7.85	
1246	9/1/2011	Well In Field	Upland		3.32	< .025							2			6.5	
1247	8/10/2011	Kitchen Sink	Upland		4.79	0.41							1700			7.89	
1248	8/10/2011	Kitchen Sink	Upland		4.46	0.15							390			9.28	
1249	8/11/2011	Pressure Tank	Valley		8.6	9.7							16000			8.15	
1250	1/14/2010	Basement At Pressure Tank	Upland		7.69	< .025		< .05		8.78	< .025		0.29			7.6	
1251	2/3/2010	Holding Tank	Upland		9.88	< .025		< .05		2.6	< .025		< .1			6.06	
1252	8/18/2011	Kitchen Sink	Valley	Yes	1.7	< 26	130	< .05	< .001	8.5	0.00065	< .0002	< 26	0.18		7.78	1.2
1253	1/31/2010	Basement At Pressure Tank	Upland		9.09	< .025		< .05		2.8	< .025		0.11			6.85	
1254	1/24/2010	Kitchen Sink	Upland		5.53	< .025		< .05		5.61	< .025		0.28			7.41	
1255	1/21/2010	Pressure Tank	Valley	Yes	6.29	0.053		0.245		3.19	0.075		250			8.36	
1256	2/12/2009		Upland	Yes		0.19		0.072		5.16	<0.01		0.24				
1257	4/8/2009		Upland			<0.025		<0.025		6.58	<0.025		0.55				
1258	1/16/2010	Basement Pressure Tank	Upland	Yes	5.57	< .025		< .05		6.48	< .025		0.29			7.78	
1259	1/24/2010	Pressure Tank - Drilled Well	Valley	Yes	6.77			< .05		2.88	< .025		6.2			7.03	
1260	1/31/2010	Crawl Space At Pressure Tank	Valley		7.99	< .025		< .05		9	0.028		4.7			7.68	
1261	1/31/2010	Pressure Tank	Valley		9.66	< .025		0.433		2.8	< .025		0.31			7.52	

Table S.8. Groundwater Quality Data for 1701 "Pre-Drill" Water Well Samples from Susquehanna County, Pennsylvania

Water Well ID	Sample Date	Sampling Port	Topo-graphic Location	Gas Extraction Area (Within 1 km of Active Gas Well)	Dissolved Oxygen - Field (mg/L)	Ethane ug/L	Hardness (mg/L)	Iron (mg/L)	Lead (mg/L)	Magnesium (mg/L)	Manganese (mg/L)	Mercury (mg/L)	Methane ug/L	Nitrate as N (mg/L)	Oil Grease (mg/L)	pH - Field (SU)	Potassium (mg/L)
1262	1/24/2010	Outside Spigot	Upland			< .025		< .05		5.51	< .025		< 1.7				
1263	1/24/2010	Kitchen Sink	Upland			< .025		< .05		5.51	< .025		1.7				
1264	1/23/2010	Pressure Tank In Basement	Valley	Yes	9.59	< .025		< .05		1.2	< .025		0.27			6.48	
1265	1/18/2010	Basement Pressure Tank	Upland		8.34	< .025		< .05		3.76	< .025		0.38			7.35	
1266	1/10/2010	Basement Pressure Tank	Upland		2.62	0.089		0.068		< 1	< .025		700			8.21	
1267	1/11/2010	Pressure Tank	Upland		9.32	< .025		< .05		3.64	< .025		0.6			7.37	
1268	12/9/2009	Basement At Pressure Tank	Upland	Yes		< .025		< .05		5.67	< .025		0.13				
1269	1/18/2010	Pressure Tank	Upland		4.71	< .025		< .05		8.08	< .025		1.5			7.61	
1270	1/31/2010		Upland	Yes		< .025		< .05		6.32	< .025		1.2				
1271	8/7/2009	Kitchen Sink	Valley					< .025		2.99	< .025		6.1		< 5		
1272	1/12/2010	Kitchen Sink At Ashley Sarnosky'S	Upland		9.01	< .025		< .05		1.67	< .025		0.18			6.73	
1273	2/21/2010	Kitchen Sink	Upland		7.14	< .025		< .05		7.01	< .025		1.4			7.47	
1274	8/6/2009		Upland	Yes				< .025		8.06	< .025		0.8		< 5		
1275	2/15/2010	Pressure Tank	Upland		3.65	< .025		0.205		11.9	0.174		30			7.62	
1276	2/13/2010	Basement At Pressure Tank	Valley		5.61			0.053		11.3	0.115		3			7.64	
1277	2/27/2010	Basement At Spigot	Valley		8.03	< .025		< .05		4.48	< .025		0.55			6.64	
1278	2/18/2010	Pressure Tank	Upland	Yes				< .05		6.9	< .025		0.15				
1279	2/16/2010	Kitchen Sink	Upland		3.36	< .025		< .05		7.4	< .025		3.2			7.74	
1280	2/18/2010	Kitchen Sink	Upland	Yes	8.49	< .025		< .05		6.98	< .025		0.13			7.02	
1281	2/18/2010	Kitchen Sink	Upland		7.27	< .025		< .05		7.88	< .025		0.68			7.35	
1282	2/18/2010	Kitchen Sink	Upland		6.67	< .025		< .05		8.53	< .025		0.12			7.21	

Table S.8. Groundwater Quality Data for 1701 "Pre-Drill" Water Well Samples from Susquehanna County, Pennsylvania

Water Well ID	Sample Date	Sampling Port	Topo-graphic Location	Gas Extraction Area (Within 1 km of Active Gas Well)	Dissolved Oxygen - Field (mg/L)	Ethane ug/L	Hardness (mg/L)	Iron (mg/L)	Lead (mg/L)	Magnesium (mg/L)	Manganese (mg/L)	Mercury (mg/L)	Methane ug/L	Nitrate as N (mg/L)	Oil Grease (mg/L)	pH - Field (SU)	Potassium (mg/L)
1283	2/22/2010	Rental House Basement At Pressure Tank	Upland		7.98	< .025		< .05		6.98	< .025		1.3			7.11	
1284	2/16/2010	Kitchen Sink	Upland		2.86	< .025		< .05		9.89	< .025		0.2			7.03	
1285	2/21/2010	Kitchen Sink	Upland		3.01	< .043		< .05		9.1	0.072		280			7.72	
1286	2/18/2010	Pressure Tank	Valley		5.8	< .025		14.7		11.6	0.43		1.3			6.63	
1287	2/6/2010	Basement At Pressure Tank	Valley		8.24			< .05		7.19	< .025		0.2			7.07	
1288	2/13/2010	Pressure Tank	Upland		10.01	< .025		0.055		9.06	< .025		0.14			7.15	
1289	2/1/2010	Basement Sink	Valley	Yes	10.13	< .025		< .05		6.99	< .025		3.5			7.89	
1290	2/21/2010	Pressure Tank	Upland		10.9	< .025		0.07		3.4	< .025		0.32			7.24	
1291	2/21/2010	Barn Well	Upland		9.82	< .025		< .05		4.08	< .025		0.58			6.09	
1292	2/16/2010	Kitchen Sink	Upland		5.86	< .025		< .05		7.62	< .025		0.24			7.16	
1293	2/22/2010	Hand Dug Well	Upland		7.77	< .025		< .05		5.06	< .025		1.4			6.81	
1294	2/16/2010	Bathroom Sink	Upland	Yes	3.12	< .025		< .05		8.14	< .025		0.17			7.24	
1295	2/16/2010	Pressure Tank	Upland		6.37			< .05		8.27	< .025		0.12			6.54	
1296	2/7/2010	Main House Pressure Tank	Valley		5.16	< .025		0.23		10.4	0.338		9.4			7.32	
1297	2/7/2010	Rental Property Pressure Tank	Upland		8.07	< .025		< .05		6.55	< .025		0.38			6.22	
1298	2/13/2010	Kitchen Sink	Upland		4.61	< .025		< .05		5.69	< .025		7.9			7.59	
1299	2/13/2010	Well House	Upland		5.51	< .025		0.081		7.19	< .025		0.14			6.66	
1300	8/6/2009		Valley					0.345		1.76	< .025		1.4		< 5		
1301	2/7/2010	Barn - Pump	Upland		8.07	< .025		0.097		5.61	< .025		0.46			6.34	
1302	2/7/2010	Pressure Tank	Upland		8.38	< .025		< .05		8.12	< .025		0.47			7.2	
1303	2/7/2010	Pressure Tank	Upland		10.49	< .025		< .05		4.61	< .025		0.27			7.61	
1304	2/6/2010	Pressure Tank	Valley		5.51	< .025		2.53		15.6	0.033		60			7.29	
1305	2/6/2010	Basement At Pressure Tank	Valley		5.66			< .05		8.45	< .025		< .1			7.4	

Table S.8. Groundwater Quality Data for 1701 "Pre-Drill" Water Well Samples from Susquehanna County, Pennsylvania

Water Well ID	Sample Date	Sampling Port	Topo-graphic Location	Gas Extraction Area (Within 1 km of Active Gas Well)	Dissolved Oxygen - Field (mg/L)	Ethane ug/L	Hardness (mg/L)	Iron (mg/L)	Lead (mg/L)	Magnesium (mg/L)	Manganese (mg/L)	Mercury (mg/L)	Methane ug/L	Nitrate as N (mg/L)	Oil Grease (mg/L)	pH - Field (SU)	Potassium (mg/L)
1306	2/6/2010	Pressure Tank	Valley		8.65	< .025		< .05		8.18	< .025		< .1			7.53	
1307	2/15/2010	Outside Hydrant	Upland	Yes	7.76	< .025		< .05		6.82	< .025		0.28			6.97	
1308	2/27/2010	Hand Dug Well 1 Back Of House	Valley		4.4	< .025		< .05		2.01	< .025		0.27			6.7	
1309	2/27/2010	Hand Dug Well 2 Front Of House	Valley		10.87	< .025		< .05		2.43	< .025		0.4			6.4	
1310	2/7/2010	Outside Faucet	Valley		8.3	0.03		2.41		11.5	0.298		34			7.72	
1311	2/6/2010	Basement At Pressure Tank	Valley		5.36			0.136		9.98	0.324		0.12			7.43	
1312	2/21/2010	Kitchen Sink	Valley		3.05	< .025		< .05		8.46	0.105		5.9			7.69	
1313	8/6/2009		Valley					< .025		7.38	< .025		5		< 5		
1314	2/22/2010	Milkhouse Sink	Upland		7.2	< .025		< .05		10.6	< .025		2.8			7.3	
1315	2/28/2010	Pressure Tank	Valley	Yes	5.68	< .025		0.074		7.97	< .025		1.4			7.38	
1316	2/13/2010	Basement Men'S Room Sink	Upland		7.92	0.03		0.386		16.4	< .025		0.26			6.9	
1317	2/28/2010	Outside Spigot	Valley		3.57	0.06		0.417		8.65	0.273		1600			7.21	
1318	2/28/2010	Kitchen Sink	Upland		8.97	< .025		< .05		6.69	< .025		0.79			7.46	
1319	2/27/2010	Basement Pressure Tank	Valley		5.03	< .025		1.58		8.68	0.047		57			7.21	
1320	3/16/2010	Kitchen Sink	Valley	Yes	11.7	< .025		0.238		1.07	< .025		0.11			6.12	
1321	3/11/2010	Kitchen Sink	Upland	Yes	7.4			< .05		< 4.78	< .025		0.32			6.87	
1322	3/18/2010	Kitchen Sink	Upland		1.89			< .05		4.54	< .025		19000			7.36	
1323	8/11/2011	Kitchen Sink	Upland	Yes	7.95	< .025							0.13			6.06	
1324	8/11/2011	Kitchen Sink	Valley		5.56	< .025							0.39			7.89	
1325	8/11/2011	Kitchen Sink	Upland		8.45	< .025							0.11			6.82	
1326	8/11/2011	Kitchen Sink	Upland		8.54	< .025							0.4			7.55	
1327	8/11/2011	Kitchen Sink	Upland		6.09	< .025							0.2			7.89	
1328	8/11/2011	Kitchen Sink	Upland		67.5	< .025							0.17			8.02	

Table S.8. Groundwater Quality Data for 1701 "Pre-Drill" Water Well Samples from Susquehanna County, Pennsylvania

Water Well ID	Sample Date	Sampling Port	Topo-graphic Location	Gas Extraction Area (Within 1 km of Active Gas Well)	Dissolved Oxygen - Field (mg/L)	Ethane ug/L	Hardness (mg/L)	Iron (mg/L)	Lead (mg/L)	Magnesium (mg/L)	Manganese (mg/L)	Mercury (mg/L)	Methane ug/L	Nitrate as N (mg/L)	Oil Grease (mg/L)	pH - Field (SU)	Potassium (mg/L)
1329	8/11/2011	Kitchen Sink	Upland		6.32	< .025							0.2			7.32	
1330	8/6/2009		Valley					< .025		3.95	0.1		10		< 5		
1331	8/11/2011	Kitchen Sink	Upland		68.1	< .025							1.9			8.05	
1332	8/11/2011	Kitchen Sink	Valley		6.21	< .025							2.2			7.85	
1333	8/11/2011	Kitchen Sink	Upland		5.56	0.27							2500			7.98	
1334	8/11/2011	Kitchen Sink	Valley			<0.025							0.37				
1335	8/12/2011	Kitchen Sink	Valley		6.49	< .025							< .1			7.26	
1336	8/12/2011	Kitchen Sink	Valley		6.11	< .025							0.24			6.99	
1337	8/12/2011	Kitchen Sink	Valley		10.6	< .025							0.1			8.1	
1338	8/12/2011	Kitchen Sink	Valley		3.18	< .025							0.11			7.75	
1339	8/12/2011	Kitchen Sink	Valley		7	< .025							0.15			7.66	
1340	5/13/2010		Upland	Yes									50				
1341	8/12/2011	Pressure Tank	Upland		4.15	< .025							0.15			7.3	
1342	8/12/2011	Kitchen Sink	Valley		3.18	< .025							< .1			7.7	
1343	8/12/2011	Bathroom Sink	Valley		4.11	< .025							0.64			7.26	
1344	8/14/2011	Outside Spigot	Valley		7.32	0.06							340			8.13	
1345	8/14/2011	Kitchen Sink	Upland		6.72	< .025							56			6.76	
1346	5/13/2010		Upland					< .05		6.02	< .025		24				
1347	8/14/2011	Kitchen Sink	Valley		6.71	< .025							0.13			7.18	
1348	8/14/2011	Kitchen Sink	Upland		9.55	< .025							0.2			8.06	
1349	8/14/2011	Kitchen Sink	Upland		5.59	< .025							0.6			7.87	
1350	8/14/2011	Pressure Tank	Upland		4.36	< .025							0.19			7.85	
1351	8/14/2011	Kitchen Sink	Upland		7.18	< .025							< .1			7.04	
1352	8/15/2011	Kitchen Sink	Upland		5.72	< .025							1.5			7.98	
1353	8/15/2011	Barn Sink	Upland		8.36	< .025							0.68			7.25	
1354	8/15/2011	Kitchen Sink	Valley		6.09	< .025							0.16			7.41	
1355	8/15/2011	Kitchen Sink	Valley		5.48	< .025							0.14			7.84	
1356	8/15/2011	Sink In Barn	Valley		2.31	< .025							14			7.22	

Table S.8. Groundwater Quality Data for 1701 "Pre-Drill" Water Well Samples from Susquehanna County, Pennsylvania

Water Well ID	Sample Date	Sampling Port	Topo-graphic Location	Gas Extraction Area (Within 1 km of Active Gas Well)	Dissolved Oxygen - Field (mg/L)	Ethane ug/L	Hardness (mg/L)	Iron (mg/L)	Lead (mg/L)	Magnesium (mg/L)	Manganese (mg/L)	Mercury (mg/L)	Methane ug/L	Nitrate as N (mg/L)	Oil Grease (mg/L)	pH - Field (SU)	Potassium (mg/L)
1357	8/16/2011	Kitchen Sink	Upland		4.85	< .025							0.1			7.46	
1358	8/16/2011	Outside Spigot	Upland		3.52	< .025							0.73			7.77	
1359	8/16/2011	Kitchen Sink	Upland		2.48	< .025							0.22			7.83	
1360	8/16/2011	Kitchen Sink	Upland		2.43	< .025							0.4			7.82	
1361	8/16/2011	Kitchen Sink	Valley		7.03	< .025							0.24			6.84	
1362	8/16/2011	Spigot On Side Of House	Upland		7.9	< .025							3			7.6	
1363	8/17/2011	Kitchen Sink	Upland		5.64	< .025							0.16			7.87	
1364	8/17/2011	Outside Spigot	Valley		6.53	< .025							0.18			6.96	
1365	8/17/2011	Kitchen Sink	Upland		4.26	< .025							< .1			7.7	
1366	8/17/2011	Kitchen Sink	Valley		2.31	0.051							620			8.15	
1367	8/17/2011	Kitchen Sink	Valley		5.45	< .025							2			7.87	
1368	8/17/2011	Kitchen Sink	Valley		5.05	< .025							0.12			7.51	
1369	8/18/2011	Kitchen Sink	Upland		5.38	< .025							0.48			7.77	
1370	2/8/2010	Basement At Pressure Tank	Upland	Yes	5.21	< .025		< .05		3.24	< .025		0.52			7.86	
1371	8/18/2011	Kitchen Sink	Valley		4.09	< .025							0.27			7.81	
1372	8/18/2011	Kitchen Sink	Valley		5.53	< .025							17			7.34	
1373	8/18/2011	Kitchen Sink	Valley		6.49	0.025							0.28			6.88	
1374	8/18/2011	Barn Sink	Upland		3.14	< .025							0.51			7.75	
1375	8/18/2011	Barn Sink	Valley		4.29	< .025							< .1			7.13	
1376	3/8/2010	Kitchen Sink	Valley	Yes	3.16	< .025		< .05		< 1	< .025		0.18			8.32	
1377	8/18/2011	Kitchen Sink	Upland		6.05	< .025							0.33			7.87	
1378	8/19/2011	Kitchen Sink	Upland		5.55	< .025							360			7.32	
1379	8/19/2011	Kitchen Sink	Upland		5	<0.025							<0.1			7.45	
1380	8/19/2011	Kitchen Sink	Upland			0.22							340				
1381	8/17/2009		Valley					< .025		3.42	< .025		7.4		< 5		
1382	8/19/2011		Valley		4.55	< .025							2.2			7.95	

Table S.8. Groundwater Quality Data for 1701 "Pre-Drill" Water Well Samples from Susquehanna County, Pennsylvania

Water Well ID	Sample Date	Sampling Port	Topo-graphic Location	Gas Extraction Area (Within 1 km of Active Gas Well)	Dissolved Oxygen - Field (mg/L)	Ethane ug/L	Hardness (mg/L)	Iron (mg/L)	Lead (mg/L)	Magnesium (mg/L)	Manganese (mg/L)	Mercury (mg/L)	Methane ug/L	Nitrate as N (mg/L)	Oil Grease (mg/L)	pH - Field (SU)	Potassium (mg/L)
1383	8/19/2011	Kitchen Sink	Valley		5.94	< .025							3.1			7.83	
1384	8/22/2011	Kitchen Sink	Upland		5.57	< .025							1.7			6.86	
1385	8/22/2011	Kitchen Sink	Upland		6.49	< .025							< .1			7.49	
1386	8/23/2011	Outside Spigot	Upland		7.39	< .025							< .1			7.09	
1387	8/23/2011	Barn Outside Spigot	Upland		7.73	< .025							2.9			8.41	
1388	8/23/2011	Outside Spigot	Upland		5.61	< .025							0.7			7.81	
1389	3/4/2010	Post Office - Bathroom Sink	Upland	Yes	4.38	< .025		< .05		9.68	< .025		0.2			7.45	
1390	8/23/2011	Outside Spigot	Valley		7.27	< .025							31			7.05	
1391	8/23/2011	Outside Spigot	Upland		4.85	< .025							0.15			7.72	
1392	8/24/2011	Kitchen Sink	Upland		5.83	< .025							< .1			6.56	
1393	8/24/2011	Kitchen Sink	Upland		5.96	< .025							1.7			6.96	
1394	8/24/2011	Kitchen Sink	Valley		6.59	0.066							2			7.45	
1395	8/24/2011	Kitchen Sink	Upland		6.85	< .025							0.2			6.66	
1396	8/24/2011	Kitchen Sink	Valley		5.67	< .025							0.21			6.84	
1397	8/24/2011	Inside Faucet	Upland		7.22	< .025							0.15			7.78	
1398	8/24/2011	Kitchen Sink	Upland		6.72	< .025							< .1			6.75	
1399	8/24/2011	Kitchen Sink	Valley		7.92	< .025							2.4			6.55	
1400	3/8/2010	Basement At Pressure Tank	Upland		6.27	< .025		0.444		4.06	0.04		1.2			7.47	
1401	8/24/2011	Kitchen Sink	Upland			<0.025							<0.1				
1402	8/24/2011	Kitchen Sink	Upland		4.89	< .025							43			7.89	
1403	8/24/2011	Kitchen Sink	Upland		5.84	< .025							< .1			7	
1404	8/24/2011	Bathroom Sink	Valley		6.33	0.044							0.82			7.64	
1405	8/24/2011	Kitchen Sink	Valley		5.3	< .025							0.28			7.34	
1406	8/25/2011	Kitchen Sink	Valley		5.88	< .025							0.22			7.84	

Table S.8. Groundwater Quality Data for 1701 "Pre-Drill" Water Well Samples from Susquehanna County, Pennsylvania

Water Well ID	Sample Date	Sampling Port	Topo-graphic Location	Gas Extraction Area (Within 1 km of Active Gas Well)	Dissolved Oxygen - Field (mg/L)	Ethane ug/L	Hardness (mg/L)	Iron (mg/L)	Lead (mg/L)	Magnesium (mg/L)	Manganese (mg/L)	Mercury (mg/L)	Methane ug/L	Nitrate as N (mg/L)	Oil Grease (mg/L)	pH - Field (SU)	Potassium (mg/L)
1407	8/25/2011	Kitchen Sink	Valley		5.33	< .025							4.1			7.21	
1408	8/25/2011	Kitchen Sink	Valley		3.1	0.065							940			8.2	
1409	8/25/2011	Downstairs Sink	Valley		5.41	<0.025							0.16			7.54	
1410	8/25/2011	Kitchen Sink	Upland		6.39	< .025							0.15			7.68	
1411	8/25/2011	Kitchen Sink	Valley		4.09	1.2							5600			7.67	
1412	8/25/2011	Kitchen Sink	Valley		4.86	0.86							2800			8.37	
1413	8/26/2011	Kitchen Sink	Upland		5.24	< .025							0.27			7.27	
1414	8/26/2011	Outside Spigot	Valley		3.62	< .025							14			7.07	
1415	8/26/2011	Kitchen Sink	Upland		6.38	< .025							0.18			7.9	
1416	8/26/2011	Kitchen Sink	Valley		4.69	< .025							0.55			7.71	
1417	8/26/2011	Kitchen Sink	Upland		5.73	< .025							0.11			7.26	
1418	8/26/2011	Kitchen Sink	Upland		4.4	< .025							1.9			8	
1419	8/26/2011	Barn Sink	Valley		4.23	0.96							4700			8.24	
1420	8/27/2011	Kitchen Sink	Upland		6.56	< .025							0.3			7.48	
1421	8/27/2011	Kitchen Sink	Upland		7.76	< .025							1.4			7.09	
1422	10/30/2009	Basement At Pressure Tank	Upland	Yes	7.79	< .025		< .05		4.58	< .025		1.4			7.09	
1423	8/31/2011	Kitchen Sink	Upland		5.75	< .025							0.23			7.35	
1424	8/31/2011	Kitchen Sink	Valley			<0.025							0.14				
1425	8/31/2011	Spigot On Well	Valley		4.1	< .025							0.36			7.57	
1426	8/31/2011	Kitchen Sink	Upland		6.04	0.15							1			6.88	
1427	9/1/2011	Kitchen Sink	Valley		4.82	< .025							< .1			7.59	
1428	9/1/2011	Spigot Side Of House	Valley		5.68	< .025							0.19			7.54	
1429	9/2/2011	Kitchen Sink	Upland		5.07	< .025							0.66			7.38	
1430	9/2/2011	Spigot On Well	Valley		5.55	< .025							< .1			7.27	
1431	3/7/2010	Kitchen Sink	Valley		2.46	0.19		< .05		< 1	< .025		9			7.67	

Table S.8. Groundwater Quality Data for 1701 "Pre-Drill" Water Well Samples from Susquehanna County, Pennsylvania

Water Well ID	Sample Date	Sampling Port	Topo-graphic Location	Gas Extraction Area (Within 1 km of Active Gas Well)	Dissolved Oxygen - Field (mg/L)	Ethane ug/L	Hardness (mg/L)	Iron (mg/L)	Lead (mg/L)	Magnesium (mg/L)	Manganese (mg/L)	Mercury (mg/L)	Methane ug/L	Nitrate as N (mg/L)	Oil Grease (mg/L)	pH - Field (SU)	Potassium (mg/L)
1432	9/2/2011	Kitchen Sink	Upland		4.29	< .025							46			7.69	
1433	9/2/2011	Kitchen Sink	Valley		5.85	< .025							< .1			7.51	
1434	9/2/2011	Bathroom Sink	Upland		6.73	< .025							< .1			7.2	
1435	9/2/2011	Kitchen Sink	Upland		5.42	< .025							0.39			7.6	
1436	9/2/2011	Well Spigot	Upland		5.91	< .025							< .1			6.78	
1437	3/8/2010	Basement At Pressure Tank	Upland		6.81	< .025		< .05		5.03	< .025		0.56			7.43	
1438	9/2/2011	Kitchen Sink	Valley		4.48	< .025							< .1			7.91	
1439	9/7/2011	Kitchen Sink	Upland		7.9	< .025							1.3			7	
1440	9/7/2011	Kitchen Sink	Upland		4.37	< .025							0.17			7.8	
1441	9/7/2011	Kitchen Sink	Valley		8.56	< .025							0.24			7.35	
1442	9/7/2011	Kitchen Sink	Upland		7.25	< .025							0.54			7.99	
1443	9/8/2011	Kitchen Faucet	Valley			< .025							17				
1444	9/8/2011	Kitchen Sink	Valley		4.58	< .025							1.9			7.58	
1445	9/8/2011	Outside Spigot	Upland		6.6	< .025							0.19			7.32	
1446	9/8/2011	Kitchen Sink	Valley		5.94	< .025							0.57			7.54	
1447	9/8/2011	Kitchen Sink	Upland		6.2	< .025							0.16			7.94	
1448	9/8/2011	Kitchen Sink	Upland		5.84	< .025							0.19			7	
1449	9/8/2011	Kitchen Sink	Upland		203	< .025							0.17			7.72	
1450	3/8/2010	Basement Pressure Tank	Upland		5.73	< .025		< .05		4.8	< .025		0.25			7.62	
1451	8/20/2009		Upland	Yes				< .025		5.98	< .025		0.53		< 5		
1452	3/7/2010	Pressure Tank	Upland		5.53	< .025		< .05		3.16	< .025		0.35			7.47	
1453	3/7/2010	Kitchen Sink	Upland		5.55			< .05		5.25	< .025		0.25			7.51	
1454	3/11/2010	Pressure Tank	Valley		5.03			< .05		7.95	< .025		2.4			7.49	
1455	5/1/2010	Kitchen Sink	Valley					< .05		7.35	< .025		6.1				
1456	5/1/2010	Kitchen Sink	Valley		13.63			< .05		1.74	< .025		1.4			6.5	

Table S.8. Groundwater Quality Data for 1701 "Pre-Drill" Water Well Samples from Susquehanna County, Pennsylvania

Water Well ID	Sample Date	Sampling Port	Topo-graphic Location	Gas Extraction Area (Within 1 km of Active Gas Well)	Dissolved Oxygen - Field (mg/L)	Ethane ug/L	Hardness (mg/L)	Iron (mg/L)	Lead (mg/L)	Magnesium (mg/L)	Manganese (mg/L)	Mercury (mg/L)	Methane ug/L	Nitrate as N (mg/L)	Oil Grease (mg/L)	pH - Field (SU)	Potassium (mg/L)
1457	5/3/2010	Outside Spigot On Garage	Upland	Yes	10.82			< .05		1.31	< .025		3.6			6.29	
1458	5/6/2010	Kitchen Sink	Upland		6.5			< .05		6.19	< .025		0.2			7.3	
1459	5/6/2010	Kitchen Sink	Valley	Yes	25.4			< .05		2.28	< .025		18000			8.39	
1460	5/7/2010	Kitchen Sink	Valley	Yes	4.71			< .05		3.55	< .025		0.52			7.69	
1461	8/24/2011	Kitchen Sink	Valley		3.73	< 26	98	< .05	< .001	5.3	0.001	< .0002	< 26	0.66		7.7	1
1462	8/24/2011	Inside Faucet	Valley		0.95	< 26	100	0.056	< .001	6.5	0.0093	< .0002	< 26	0.39		7.95	1.3
1463	8/24/2011	Pressure Tank Hose Bib	Upland		4.93	< 26	58	< .05	< .001	2.5	0.0014	< .0002	43.5	0.38		7.21	0.99
1464	5/7/2010	Outside Spigot	Upland					< .05		6.16	< .025		< .1				
1465	8/30/2011	Inside Faucet	Valley		4.3	< 5	180	0.17	< .001	19	0.0097	< .0002	< 5	0.077		7.06	1.5
1466	5/13/2010	Kitchen Sink	Valley		4.62	< .025		< .05		6.69	< .025		0.29			7.48	
1467	8/30/2011	Pressure Tank	Valley		4.13	< 5	140	0.34	0.0026	15	0.025	< .0002	< 5	0.18		6.79	1.4
1468	5/13/2010	Kitchen Sink	Upland		3.79			< .05		7.85	< .025		0.12			7.58	
1469	5/13/2010	Outside Spigot	Upland		6.23			< .05		7.47	< .025		0.11			7.52	
1470	8/30/2011	Inside Faucet	Upland		4.47	< 5	140	0.14	< .001	14	0.00076	< .0002	< 5	0.54		7.58	1.8
1471	7/7/2009	Kitchen Sink	Upland		4.95	< .025		< .025		4	< .025		< .1		< 5	7.61	
1472	5/20/2010	Kitchen Sink	Upland		3.73			< .05		6.93	< .025		1.1			7.44	
1473	5/20/2010	Basement At Pressure Tank	Upland		8.83			< .05		4.88	< .025		< .1			7.06	
1474	5/20/2010	Pressure Tank- Quarry Top Well	Upland										4.6				
1475	5/20/2010	Pressure Tank At Trailer Park	Upland		6.81			< .05		7.43	0.104		1.7			7.59	
1476	5/20/2010	Quarry Sulfur Well	Upland		5.17			< .05		7.44	< .025		0.98			7.45	
1477	5/20/2010	Spigot In Garage	Upland		10.01			< .05		3.38	< .025		0.26			7.43	
1478	3/16/2010	Outside Spigot	Upland		10.59	< .025		0.103		1.93	< .025		0.87			6.52	

Table S.8. Groundwater Quality Data for 1701 "Pre-Drill" Water Well Samples from Susquehanna County, Pennsylvania

Water Well ID	Sample Date	Sampling Port	Topo-graphic Location	Gas Extraction Area (Within 1 km of Active Gas Well)	Dissolved Oxygen - Field (mg/L)	Ethane ug/L	Hardness (mg/L)	Iron (mg/L)	Lead (mg/L)	Magnesium (mg/L)	Manganese (mg/L)	Mercury (mg/L)	Methane ug/L	Nitrate as N (mg/L)	Oil Grease (mg/L)	pH - Field (SU)	Potassium (mg/L)
1479	4/19/2009	Kitchen Sink	Upland		9.88	< .025		< .025		5.36	< .025		0.77		< 5	6.6	
1480	3/18/2010	Kitchen Sink	Upland		10.62	< .025		< .05		1.82	< .025		1.8			5.82	
1481	3/18/2010	Pressure Tank	Upland	Yes	9.5	< .025		< .05		1.99	< .025		35			5.84	
1482	3/22/2010	Kitchen Sink	Valley	Yes	9.56	< .025		< .05		3.32	< .025		2.1			7.02	
1483	1/13/2011		Valley	Yes		1.3	107.416	< .05	0.0016	8.5	0.023	< .0002	39	< 1	< 5		1.3
1484	3/25/2010	Basement At Pressure Tank	Upland		6.43			< .05		5.7	< .025		1.4			7.64	
1485	3/25/2010	Hand Dug Well	Valley		11.96	< .025		< .05		1.44	< .025		1.4			6.15	
1486	6/14/2011	Directly From Pressure Tank Cwt In Sw Corner Of Ba	Upland	Yes	10.03	< 26	50	0.54	< .001	3.7	0.0099	< .0002	< 26	0.4		7.19	1
1487	6/14/2011	Kitchen Sink	Upland		2.91	< 26	140	0.1	0.0025	9	0.08	< .0002	< 26	< .05		7.6	1.3
1488	6/14/2011	Hose Bib	Upland		3.6	< 26	63	0.18	< .001	4.4	0.0019	< .0002	< 26	7.8	< 4.9	6.29	13
1489	3/25/2010	Spring At Overflow Pipe	Valley		11.68	< .025		< .05		1.98	< .025		0.13			6.07	
1490	6/14/2011	Outside Hose Bib On Sw End Of Home	Valley		2.5	< 26	99	0.12	< .001	5	0.22	< .0002	< 26	0.35		7.24	1.4
1491	3/28/2010	Basement At Pressure Tank	Upland		5.89	0.25		< .05		4.75	0.938		8100			6.93	
1492	1/13/2011		Upland	Yes	9.24	<0.025	92.565	<0.05	<0.0003	5.5	<0.0025	<0.0002	0.16	<1	<5	6.96	1.3
1493	6/16/2011	Wash Tub In Garage	Upland		6.43	< 26	120	< .05	< .001	7.8	0.017	< .0002	< 26	0.053	< 4.7	7.43	1.3
1494	6/16/2011	Kitchen Sink	Valley		19.86	< 26	74	0.073	0.0029	3.3	0.0016	< .0002	< 26	1.5		6.02	1
1495	6/27/2011	Bathroom Sink Body Shop	Valley	Yes	6.55	< 26	96	0.059	< .001	9.8	0.0018	< .0002	< 26	0.2		7.31	1.2
1496	6/28/2011	Garage Sink	Valley		8.94	< 26	98	< .05	< .001	5.7	< .0005	< .0002	< 26	0.58		7.55	1.1
1497	6/28/2011	Kitchen Sink	Upland		13.03	< 26	110	< .05	< .001	5.5	< .0005	< .0002	< 26	0.4		7.26	0.88

Table S.8. Groundwater Quality Data for 1701 "Pre-Drill" Water Well Samples from Susquehanna County, Pennsylvania

Water Well ID	Sample Date	Sampling Port	Topo-graphic Location	Gas Extraction Area (Within 1 km of Active Gas Well)	Dissolved Oxygen - Field (mg/L)	Ethane ug/L	Hardness (mg/L)	Iron (mg/L)	Lead (mg/L)	Magnesium (mg/L)	Manganese (mg/L)	Mercury (mg/L)	Methane ug/L	Nitrate as N (mg/L)	Oil Grease (mg/L)	pH - Field (SU)	Potassium (mg/L)
1498	3/18/2010	Kitchen Sink	Upland		5.19			< .05		4.8	< .025		4.4			7.01	
1499	5/21/2010	Kitchen Sink	Upland	Yes	2.68			< .05		5.37	< .025		0.54			7.27	
1500	5/21/2010	Hand Dug #1	Upland	Yes	7.8			< .05		1.38	< .025		3.6			5.81	
1501	7/6/2011	Kitchen Sink	Upland		220	< 26	130	< .05	< .001	7.8	< .0005	< .0002	< 26	1.2		6.23	1.3
1502	7/6/2011	Kitchen Sink	Upland		404	< 26	82	< .05	< .001	5.8	< .0005	< .0002	< 26	4.5		6.24	1.3
1503	5/21/2010	Hand Dug Well #2	Upland	Yes	10.52			< .05		1.61	< .025		0.55			5.84	
1504	7/6/2011	Kitchen Sink	Valley		598	< 26	72	< .05	< .001	4.9	< .0005	< .0002	< 26	6.3		6.59	0.97
1505	7/6/2011	Kitchen Sink	Upland		6.43	< 26	94	0.82	< .001	4.4	0.0049	< .0002	< 26	0.98		7.86	1.1
1506	7/7/2011	Pressure Tank Tap	Valley		515	< 26	73	< .05	< .001	4.2	0.014	< .0002	< 26	3	< 5.1	6.02	2.9
1507	5/21/2010		Valley					< .05		7.3	< .025		0.19				
1508	7/14/2011	Outdoor Spigot	Upland		4.87	< 26	140	< .05	< .001	7.9	< .0005	< .0002	< 26	0.85		7.28	1.3
1509	7/16/2011	Pressure Tank	Valley		1.47	< 26	73	1.8	0.0086	6.2	0.13	< .0002	60.1	< .05		8.43	1.4
1510	5/22/2010	Spare Well	Upland		4.02			< .05		2.56	< .025		0.47			7.63	
1511	5/22/2010	Kitchen Sink	Upland		4.97			< .05		5.91	< .025		< .1			7.29	
1512	7/18/2011	Outside Hose Bib	Upland		4.31	< 26	110	0.62	0.0061	5	0.91	< .0002	< 26	1.1		7.37	1.1
1513	7/18/2011	Hydrant Northwest Of Well	Upland		2.3	< 26	150	< .05	< .001	6.4	0.018	< .0002	< 26	0.49		7.47	2.8
1514	7/18/2011		Valley	Yes		< 26	93	< .05	< .001	3.6	0.0015	< .0002	< 26	0.38	< 4.7		0.75
1515	5/16/2011	Pressure Tank	Upland			<26	97	<0.05	<0.001	7.1	0.01	<0.0002	<26	0.14	<5.1		0.84
1516	5/16/2011	Slop Sink In Barn	Upland		1.99	< 26	80	< .05	< .001	7.4	< .0005	< .0002	< 26	1.5		7.21	0.93
1517	5/16/2011	Pressure Tank	Upland		1.87	< 26	130	0.29	< .001	13	0.023	< .0002	< 26	< .05		7.43	1.1
1518	5/16/2011	Outdoor Spigot	Upland		5.75	< 26	44	0.084	< .001	2.8	0.0043	< .0002	< 26	0.3		6.38	0.75
1519	7/25/2011	Kitchen Sink Cold Water Tap	Upland		9.51	< 26	88	0.12	0.0019	4	0.055	< .0002	< 26	1		7.1	1.2
1520	5/17/2011	Outside Spigot	Upland		2.94	< 26	120	< .05	0.001	13	0.00087	< .0002	< 26	0.75		7.39	1.1
1521	5/17/2011	Kitchen Sink	Valley		2.4	< 26	67	0.11	< .001	6	0.058	< .0002	439	< .05		8.18	1.1

Table S.8. Groundwater Quality Data for 1701 "Pre-Drill" Water Well Samples from Susquehanna County, Pennsylvania

Water Well ID	Sample Date	Sampling Port	Topo-graphic Location	Gas Extraction Area (Within 1 km of Active Gas Well)	Dissolved Oxygen - Field (mg/L)	Ethane ug/L	Hardness (mg/L)	Iron (mg/L)	Lead (mg/L)	Magnesium (mg/L)	Manganese (mg/L)	Mercury (mg/L)	Methane ug/L	Nitrate as N (mg/L)	Oil Grease (mg/L)	pH - Field (SU)	Potassium (mg/L)
1522	5/17/2011	Kitchen Sink	Valley		0.64	< 26	80	0.065	< .001	7.2	0.044	< .0002	32.9	< .05		7.95	1
1523	5/17/2011	Outdoor Spigot	Upland		3.86	< 26	89	< .05	< .001	8.1	< .0005	< .0002	< 26	0.22		7.3	1.1
1524	5/17/2011	Garage Pressure Tank	Upland		2.97	< 26	93	0.09	0.0035	7.9	0.0022	< .0002	< 26	0.33	< 4.8	7.27	0.91
1525	3/31/2010	Spigot On Porch	Valley		8.38			< .05		1.8	< .025		0.13			6.43	
1526	5/17/2011	Pressure Tank Spigot	Upland		5.66	< 26	90	< .05	0.0011	8.8	< .0005	< .0002	< 26	0.37		7.32	1.1
1527	3/31/2010	Hydrant At Well Head	Upland		7.9	< .025		< .05		2.66	< .025		0.36			7.15	
1528	8/4/2011	Inside Faucet	Valley		10.04	< 26	110	< .05	< .001	10	< .0005	< .0002	< 26	0.76		7.44	1.6
1529	8/9/2011	Outside Faucet	Upland			<26	89	<0.05	<0.001	8.3	<0.0005	<0.0002	<26	1.1	<4.7		1.2
1530	3/28/2010	Prssure Tank At Fitzsimon'S House	Upland		8.26			< .05		3.26	< .025		1.6			7.75	
1531	5/18/2011	Kitchen Sink	Upland		3.62	<26	95	<0.05	<0.001	9.5	<0.005	<0.0002	<26	0.95		7.4	1.1
1532	5/18/2011	Kitchen Sink	Upland		1.52	< 26	43	< .05	< .001	3.3	0.0022	< .0002	< 26	0.42		8.45	1
1533	5/18/2011	Kitchen Sink	Upland		4.01	< 26	35	0.11	< .001	2.2	0.065	< .0002	< 26	1.5		5.7	0.75
1534	5/18/2011	Kitchen Sink	Upland		3.63	<26	68	0.12	<0.001	6.2	0.0026	<0.0002	<26	0.78		7.4	1.2
1535	3/28/2010	Pressure Tank In Furnace Room	Upland		10.95			< .05		4.87	< .025		0.15			7.01	
1536	5/19/2011	Bathroom Sink Body Shop	Valley	Yes	3.04	< 26	95	< .05	< .001	9.9	0.0016	< .0002	< 26	0.17		7.49	1.3
1537	4/1/2010	Pressure Tank	Upland					< .05		3.36	< .025		0.33				
1538	5/24/2011	Basement Pressure Tank	Valley		1.73	< 26	16	< .05	< .001	1.4	0.01	< .0002	2200	< .05		9.7	1.2
1539	5/24/2011	Kitchen Sink	Upland		3.59	< 26	97	< .05	< .001	9.4	0.0017	< .0002	< 26	0.2		7.5	1.4
1540	4/5/2010	Kitchen Sink	Upland					0.292		4.73	< .025		0.73				
1541	4/5/2010	Kitchen Sink Father'S	Upland					0.137		3.84	0.084		30				

Table S.8. Groundwater Quality Data for 1701 "Pre-Drill" Water Well Samples from Susquehanna County, Pennsylvania

Water Well ID	Sample Date	Sampling Port	Topo-graphic Location	Gas Extraction Area (Within 1 km of Active Gas Well)	Dissolved Oxygen - Field (mg/L)	Ethane ug/L	Hardness (mg/L)	Iron (mg/L)	Lead (mg/L)	Magnesium (mg/L)	Manganese (mg/L)	Mercury (mg/L)	Methane ug/L	Nitrate as N (mg/L)	Oil Grease (mg/L)	pH - Field (SU)	Potassium (mg/L)
1542	5/24/2011	Pressure Tank	Valley	Yes	0.18	< 26	140	1.4	0.0012	7.1	0.0043	< .0002	< 26	1.1		7.73	0.97
1543	4/1/2010	Kitchen Sink	Upland					< .05		2.32	< .025		3.1				
1544	4/1/2010	Kitchen Sink	Upland		13.24			< .05		3.08	< .025		0.16			6.35	
1545	6/1/2011	Kitchen Sink	Upland		1.42	< 26	130	< .05	< .001	6	0.025	< .0002	< 26	0.13		6.3	1.8
1546	4/1/2010	Kitchen Sink	Upland		5.45	< .025		< .05		4.67	< .025		1.1			6.81	
1547	6/2/2011	Township Bldg Garage Slop Sink	Valley		1.73	< 26	98	< .05	0.0031	5	0.083	< .0002	< 26	< .05		6.77	1.9
1548	6/3/2011	Outside Spigot	Valley		0.46	< 26	40	< .05	< .001	1.4	0.092	< .0002	151	< .05		7.61	2.7
1549	6/3/2011	Pressure Tank	Valley	Yes		<26	130	0.25	<0.001	8.4	0.0027	<0.0002	<26	0.57	<5		1.3
1550	6/9/2011	Outside Spigot	Valley	Yes	2.69	< 26	110	< .05	< .001	5.6	0.00053	< .0002	< 26	1.3		7.35	1.2
1551	6/9/2011	Kitchen Sink	Upland		5.5	< 26	160	< .05	< .001	8.3	0.012	< .0002	< 26	0.62		7.78	1.8
1552	6/10/2011	Pressure Tank (Basement Back Left Closet)	Valley	Yes	4.72	< 26	100	< .05	< .001	4.8	< .0005	< .0002	< 26	1.1	< 5.3	7.82	1.3
1553	6/10/2011		Valley			<26	120	0.06	<0.001	5	0.043	<0.0002	<26	<0.05	<4.8		1.2
1554	3/28/2010	Outside Spigot	Upland		2.66	0.21		< .05		< 1	0.126		16000			7.02	
1555	4/9/2010	Basement Pressure Tank	Upland		10.32	< .025		< .05		3.28	< .025		0.75			6.86	
1556	4/9/2010	Kitchen Sink	Valley	Yes	10.87			< .05		2.45	< .025		0.52			6.06	
1557	4/9/2010	Basement Pressure Tank	Valley		3.21			< .05		1.26	< .025		1400			8.73	
1558	5/27/2010	Garage Sink	Valley		8.25			< .05		4.85	< .025		0.26			7.64	
1559	5/27/2010		Upland					< .05		5.8	< .025		1.1				
1560	5/27/2010	Outside Spigot	Upland		10.93			< .05		1.21	< .025		2.4			6.7	
1561	6/3/2010	Main House Kitchen Sink	Upland										< .1				
1562	6/4/2010	Outside Hydrant	Upland		7.04								< .1			7.05	
1563	6/4/2010	Kitchen Sink	Upland	Yes	2.91								540			9.13	

Table S.8. Groundwater Quality Data for 1701 "Pre-Drill" Water Well Samples from Susquehanna County, Pennsylvania

Water Well ID	Sample Date	Sampling Port	Topo-graphic Location	Gas Extraction Area (Within 1 km of Active Gas Well)	Dissolved Oxygen - Field (mg/L)	Ethane ug/L	Hardness (mg/L)	Iron (mg/L)	Lead (mg/L)	Magnesium (mg/L)	Manganese (mg/L)	Mercury (mg/L)	Methane ug/L	Nitrate as N (mg/L)	Oil Grease (mg/L)	pH - Field (SU)	Potassium (mg/L)
1564	6/4/2010	Kitchen Sink	Upland										< .1				
1565	6/4/2010	Kitchen Sink	Valley		3.83								0.11			7.13	
1566	6/13/2010	Kitchen Sink	Valley		2.03								0.71			7.25	
1567	6/13/2010	Hydrant On Well	Valley		3.57								5600			7.45	
1568	6/13/2010	Kitchen Sink	Valley		8.04								0.21			7.24	
1569	6/13/2010	Kitchen Sink	Upland		5.86								0.18			7.1	
1570	6/13/2010		Valley										< .1				
1571	6/13/2010	Kitchen Sink	Valley		1.77	< .025							99			7.88	
1572	6/12/2010	Pressure Tank	Upland		6.81								<0.1			6.8	
1573	6/7/2010	Outside Spigot	Upland		9.25								1.1			6.81	
1574	6/7/2010	Spring Pipe	Upland		15.03								0.13			7.62	
1575	6/7/2010	Kitchen Sink	Upland		5.79								0.11			6.27	
1576	6/10/2010	Pressure Tank	Upland		9.86	< .025		< .05		3.42	< .025		< .1			7.47	
1577	6/10/2010	Kitchen Sink	Upland		5.36								< .1			7.2	
1578	6/10/2010	Pressure Tank	Upland		6.28								0.12			6.98	
1579	6/10/2010	Kitchen Sink	Upland		2.74								220			8.47	
1580	6/10/2010	Kitchen Sink	Valley		4.55								0.64			7.51	
1581	6/10/2010		Upland										< .1				
1582	6/10/2010	Kitchen Sink	Upland		6.26								< .1			6.92	
1583	3/25/2009		Valley	Yes		<0.025		<0.025		1.27	<0.025		0.16				
1584	6/10/2010	Kitchen Sink	Upland		7.99								< .1			6.44	
1585	6/12/2010	Pressure Tank	Upland		11.17								0.26			6.33	
1586	6/12/2010	Kitchen Faucet	Upland		8.14								< .1			6.75	
1587	6/12/2010	Bathroom Faucet	Valley		2.93								0.52			7.23	
1588	6/14/2010	At Pressure Tank	Valley										0.11				
1589	6/14/2010	Kitchen Sink	Upland		4								0.11			7.24	
1590	4/4/2009		Valley			< .025		< .025		1.72	< .025		0.43				
1591	6/2/2011	Dug Well	Upland	Yes	8.7	<26	49	<0.05	<0.001	2.2	0.0036	<0.0002	<26	4.4		6.01	0.76

Table S.8. Groundwater Quality Data for 1701 "Pre-Drill" Water Well Samples from Susquehanna County, Pennsylvania

Water Well ID	Sample Date	Sampling Port	Topo-graphic Location	Gas Extraction Area (Within 1 km of Active Gas Well)	Dissolved Oxygen - Field (mg/L)	Ethane ug/L	Hardness (mg/L)	Iron (mg/L)	Lead (mg/L)	Magnesium (mg/L)	Manganese (mg/L)	Mercury (mg/L)	Methane ug/L	Nitrate as N (mg/L)	Oil Grease (mg/L)	pH - Field (SU)	Potassium (mg/L)
1592	3/4/2009		Valley			0.034		< .025		4.6	< .025		0.21				
1593	4/19/2009		Upland			< .025		< .025		7.38	< .025		2.7				
1594	9/1/2009		Upland	Yes		0.1		< .025		7.14	< .025		2.3				
1595	7/19/2011		Upland										0.19				
1596	2/20/2009	Barn Sink	Valley		5.54	2.2		0.074		5.63	0.081		1900		<5.0	7.88	
1597	2/26/2009	Barn Sink	Upland		4.69	<0.025		<0.025		6.14	<0.025		1.1		<5	7.59	
1598	11/9/2009	Pressure Tank	Valley	Yes	5.93	< .025		0.081		3.25	0.04		4.2			8.16	
1599	12/6/2009	Kitchen Faucet	Upland		6.07	< .025		< .05		3.66	< .025		1.3			6.38	
1600	12/6/2009	Kitchen Faucet	Valley	Yes	8.23	< .025		< .05		2.21	< .025		0.63			6.4	
1601	12/6/2009	Pressure Tank	Valley	Yes	11.43	< .025		< .05		2.35	< .025		0.85			6.39	
1602	12/6/2009	Kitchen Faucet	Upland		4.53	< .025		< .05		10.1	< .025		0.76			6.88	
1603	12/15/2009	Kitchen Sink	Upland		9.43	<0.025		<0.05		2.33	<0.025		0.34			6.57	
1604	12/17/2009	Pressure Tank	Upland		4.33	< .025		< .05		5.06	< .025		0.67			7.48	
1605	1/11/2010	Pressure Tank	Upland		8.85	< .025		< .05		3.19	< .025		2			6.92	
1606	1/11/2010	Kitchen Faucet	Upland		6.72	0.04		< .05		5.83	< .025		0.9			7.46	
1607	1/11/2010	Kitchen Sink	Upland		8.12	<0.025		0.653		2.8	<0.025		0.29			7.02	
1608	1/11/2010	Pressure Tank	Upland		8.69	0.075		< .05		2.74	< .025		2.3			6.74	
1609	1/11/2010	Pressure Tank	Upland		2.76	< .025		< .05		7.66	0.154		3.3			7.62	
1610	1/11/2010	Pressure Tank	Upland		5.81	< .025		0.102		7.06	0.43		34			7.7	
1611	1/12/2010	Spigot Into House	Upland	Yes	9.7	<0.025		1.5		3.72	0.609		0.17			7.09	
1612	1/16/2010	Pressure Tank	Upland		4.9	< .025		< .05		5.26	< .025		5.7			7.91	
1613	1/21/2010	Kitchen Sink	Upland	Yes	3.97	<0.025		<0.05		4.76	<0.025		0.52			7.69	
1614	2/4/2010	Pressure Tank	Valley		5.93	<0.025		0.158		5.62	<0.025		0.17			7.01	
1615	2/4/2010	Kitchen Sink	Upland		9.92	<0.025		<0.05		2.26	<0.025		<0.1			7.31	
1616	2/6/2010	Kitchen Sink	Valley		7.25	<0.025		<0.05		7.39	<0.025		0.18			7.52	
1617	2/15/2010	Kitchen Sink	Valley		6.2	<0.025				8.11	<0.025		<0.1			7.54	
1618	2/15/2010	Kitchen Sink	Valley		7.89	<0.025				7.62	<0.025		<0.1			7.66	
1619	2/18/2010	Bathroom Sink	Valley	Yes	10.69	0.034		10.1		7.02	0.851		1			7.06	

Table S.8. Groundwater Quality Data for 1701 "Pre-Drill" Water Well Samples from Susquehanna County, Pennsylvania

Water Well ID	Sample Date	Sampling Port	Topo-graphic Location	Gas Extraction Area (Within 1 km of Active Gas Well)	Dissolved Oxygen - Field (mg/L)	Ethane ug/L	Hardness (mg/L)	Iron (mg/L)	Lead (mg/L)	Magnesium (mg/L)	Manganese (mg/L)	Mercury (mg/L)	Methane ug/L	Nitrate as N (mg/L)	Oil Grease (mg/L)	pH - Field (SU)	Potassium (mg/L)
1620	2/22/2010	Kitchen Faucet	Upland		5.03	< .025		< .05		10.4	0.207		4.8			7.39	
1621	2/27/2010	Kitchen Sink	Valley		7.06	<0.025		<0.05		5.48	<0.025		0.2			6.97	
1622	3/4/2010	Bathroom Sink	Upland	Yes	2.4	0.19				<1	<0.025		220			9.12	
1623	3/7/2010	Pressure Tank	Upland		10.56	0.03		< .05		2.92	< .025		0.78			6.44	
1624	3/28/2010	Kitchen Faucet	Upland		2.2	17		< .05		1.95	< .025		32000			7.76	
1625	4/8/2010	Outside Faucet	Upland		5.74	< .025		< .05		5.43	< .025		3.7			7.3	
1626	4/9/2010	Kitchen Sink	Valley		6.9	<0.025		<0.05		6.58	<0.025		0.19			6.83	
1627	4/10/2010	Kitchen Sink	Valley		3.1	<0.025		<0.05		8.76	<0.025		0.34			7.44	
1628	4/10/2010	Pressure Tank	Valley		5.72	0.36		<0.05		3.25	<0.025		3500			8.47	
1629	4/19/2010	Kitchen Sink	Valley		3.08	<0.025		<0.05		7.24	0.077		1.6			7.44	
1630	4/22/2010	Kitchen Sink	Valley		3.48	<0.025		<0.05		12.6	<0.025		0.16			7.45	
1631	4/22/2010	Kitchen Sink	Valley		4.42	0.21		<0.050		12.4	<0.025		12			7.53	
1632	4/22/2010	Stepmother'S House	Valley		3.55	<0.025		<0.05		12.3	<0.025		16			7.46	
1633	4/24/2010	Kitchen Faucet	Upland	Yes	6.03	< .025		< .05		5.82	< .025		1.4			7.27	
1634	4/22/2010	Outside Faucet	Valley		5.95	< .025		< .05		11	0.322		22			7.31	
1635	5/1/2010	Kitchen Faucet	Valley	Yes	4.03	< .025				9.04	0.171		11			7.24	
1636	5/1/2010	Kitchen Sink	Upland		2.51	<0.025		<0.05		9.11	<0.025		18			7.33	
1637	5/21/2010	Pressure Tank	Valley		3.43	< .025		< .05		4.78	< .025		620			6.94	
1638	6/3/2010	Kitchen Faucet	Upland		9.18	< .025		< .05		3.22	< .025		< 1			6.9	
1639	6/7/2010	Outside Spigot	Upland		10.31	<0.025		<0.05		4.72	<0.025		0.35			7.65	
1640	6/12/2010	Spigot In Garage	Valley			<0.025				2.05	<0.025		<0.1				
1641	6/13/2010	Kitchen Sink	Valley		3.37	<0.025		<0.05		7.56	<0.025		0.31			7.2	
1642	6/13/2010	Kitchen Sink	Valley		2.97	0.53							2500			8.76	
1643	6/23/2010	Kitchen Faucet	Upland		3.83	< .025		< .05		4.37	< .025		5			7.57	
1644	7/1/2010	Kitchen Faucet	Valley		2.31	< .025		< .05		8.83	< .025		0.42			7.15	

Table S.8. Groundwater Quality Data for 1701 "Pre-Drill" Water Well Samples from Susquehanna County, Pennsylvania

Water Well ID	Sample Date	Sampling Port	Topo-graphic Location	Gas Extraction Area (Within 1 km of Active Gas Well)	Dissolved Oxygen - Field (mg/L)	Ethane ug/L	Hardness (mg/L)	Iron (mg/L)	Lead (mg/L)	Magnesium (mg/L)	Manganese (mg/L)	Mercury (mg/L)	Methane ug/L	Nitrate as N (mg/L)	Oil Grease (mg/L)	pH - Field (SU)	Potassium (mg/L)
1645	7/2/2010	Kitchen Sink	Valley		4.7	<0.025							0.19			7.3	
1646	7/6/2010	Kitchen Faucet	Valley		6.57	< .025		< .05		3.14	< .025		0.12			7.68	
1647	7/6/2010	Kitchen Sink	Upland		5.25	<0.025		<0.050		6.37	<0.025		<0.100			7.53	
1648	7/6/2010	Pressure Tank	Upland		4.76	<0.025		<0.05		8.88	<0.025		0.32			6.34	
1649	7/7/2010	Kitchen Sink	Upland		3.6	<0.025				6.2	<0.025		0.24			6.06	
1650	7/7/2010	Kitchen Faucet	Valley		7.61	< .025		< .05		7.01	< .025		3.4			7.59	
1651	7/7/2010	Pressure Tank	Valley		5.47	<0.025							0.19			7.39	
1652	7/8/2010	Kitchen Sink	Upland		9.2	<0.025		<0.05		7.86	<0.025		0.11			7.93	
1653	7/9/2010	Pressure Tank	Valley		5.76	0.034		0.494		4.88	0.045		190			6.69	
1654	7/2/2010	Kitchen Faucet	Valley		7.09	< .025		< .05		5.93	0.051		70			7.69	
1655	7/9/2010	Outside Spigot	Valley		1.67	<0.025		<0.05		5.03	0.026		0.13			7.95	
1656	7/9/2010	Kitchen Sink	Valley		2.54	<0.025		<0.05		4.68	<0.025		<0.1			8.02	
1657	7/7/2010	Kitchen Faucet	Upland	Yes	2.55	0.16		< .05		4.18	< .025		470			6.55	
1658	7/7/2010	Kitchen Sink	Upland		3.74	<0.025		<0.05		5.08	<0.025		<0.100			7.78	
1659	7/8/2010	Kitchen Faucet	Valley		4.67	< .025		0.083		7.03	< .025		0.81			7.71	
1660	7/8/2010	Kitchen Sink	Upland		6.1	<0.025		<0.050		2.57	<0.025		<0.100			4.9	
1661	7/10/2010	Pressure Tank	Upland		8.11	< .025		< .05		5.18	< .025		1.2			6.89	
1662	7/10/2010	Basement Spigot Pre-Filter	Valley		1.07	0.028		<0.05		6.03	0.57		51			7.68	
1663	7/10/2010	Kitchen Faucet	Upland		1	< .025		< .05		1.21	0.757		610			6.2	
1664	7/10/2010	Pressure Tank	Upland		7.14	<0.025		<0.05		5.64	<0.025		0.37			6.94	
1665	7/11/2010	Kitchen Faucet	Upland		1.66	< .025		< .05		5.15	0.134		39			8.23	
1666	7/12/2010	Kitchen Faucet	Valley		2.88	0.1		< .05		7.28	0.137		540			7.06	
1667	7/13/2010	Basement (Pre-Filter)	Valley	Yes	1.49	< .025		< .05		3.65	< .025		56			7.72	
1668	7/13/2010	Kitchen Sink	Valley		3.44	<0.025				2.68	<0.025		0.14			6.33	
1669	7/13/2010	Deep Well	Valley		0.82	15		< .05		6.81	0.257		19000			7.84	

Table S.8. Groundwater Quality Data for 1701 "Pre-Drill" Water Well Samples from Susquehanna County, Pennsylvania

Water Well ID	Sample Date	Sampling Port	Topo-graphic Location	Gas Extraction Area (Within 1 km of Active Gas Well)	Dissolved Oxygen - Field (mg/L)	Ethane ug/L	Hardness (mg/L)	Iron (mg/L)	Lead (mg/L)	Magnesium (mg/L)	Manganese (mg/L)	Mercury (mg/L)	Methane ug/L	Nitrate as N (mg/L)	Oil Grease (mg/L)	pH - Field (SU)	Potassium (mg/L)
1670	7/28/2010	Kitchen Faucet	Valley		4.63	< .025		0.065		8.21	0.031		1.3			7.53	
1671	7/28/2010	Milkhouse Sink	Upland	Yes	4.35	<0.025		<0.05		6.46	<0.025		0.32			7.01	
1672	8/7/2010	Kitchen Sink	Upland		5.84	<0.025		<0.05		11.3	<0.025		0.13			6.82	
1673	8/8/2010	Kitchen Sink	Upland		9.57	<0.025		<0.05		6.52	<0.025		0.094			7.58	
1674	8/13/2010	Outside Hydrant	Valley		1.73	< .025		0.392		9.66	0.507		34			7.19	
1675	8/19/2010	Kitchen Faucet	Valley	Yes	2.51	< .025	47.8507						1.4	< 1		6.08	
1676	9/23/2010	Outside Spigot	Upland		7.85	0.96	82.19664	<0.05	<0.001	4.68	<0.025	0	3600	<1	<5	7.04	2.18
1677	10/10/2010		Valley	Yes	4.54	<0.025	72.5976	0.079	0.006	4.35	<0.025	0	1.5	<1	<5	5.78	1.4
1678	10/18/2010	Kitchen Sink	Valley	Yes	5.57	<0.025	128.0426		<0.001			<0.0002	0.2	<1	<5	6.92	1.21
1679	10/18/2010	Kitchen Faucet	Upland	Yes	2.96	< .025	168.2249						4.8	< 1	< 5	6.75	
1680	10/19/2010	Kitchen Faucet	Valley	Yes	4.03	0.019	61.84626						0.42	< 1	< 5	5.87	
1681	10/26/2010	Spring House	Valley	Yes	8.63	<0.025	59.89508		<0.001			<0.0002	0.37		<5	6.02	0.99
1682	10/29/2010	Kitchen Faucet	Upland		5.32	<0.025	132.526	<0.05	<0.001	7.2	<0.025	0	0.12	<1	<5	7.09	1.08
1683	10/29/2010	Kitchen Faucet	Upland		3.04	<0.025	173.7302	<0.05	<0.001	9.02	<0.025	0	<0.1	<1	<5	6.69	1.23
1684	11/11/2010	Kitchen Faucet	Valley	Yes	3.91	< .025	171.769						1.1	< 1	< 5	6.83	
1685	11/11/2010	Kitchen Faucet	Upland		2.77	0.11	51.9466						290	< 1	< 5	7.6	
1686	11/11/2010	Kitchen Faucet	Upland	Yes	6.4	<0.025	141.0862	<0.05	<0.0003	9.4	0.0025	<0.002	0.17	<1	<5	7.28	1.38
1687	12/2/2010	Kitchen Sink	Valley		4.5	<0.025	135.5074		<0.001	6.59	<0.025	<0.0002	0.34	<1	<5	7.54	1.14
1688	12/4/2010	Kitchen Sink	Valley		9.88	<0.025	36.7763	<0.05	<0.001	2.2	<0.025	0	<0.1	<1	<5	6.61	0.879
1689	12/5/2010	Kitchen Faucet	Valley	Yes	3.79	< .025	143.2799						19	< 1	< 5	6.93	
1690	12/9/2010	Kitchen Faucet	Upland		3.06	< .025	113.1812						0.61	< 1	< 5	6.59	
1691	12/11/2010	Kitchen Sink	Valley		8.67	<0.025	43.632						<0.1	<1	<5	6.27	
1692	12/14/2010	Kitchen Sink	Upland	Yes	9.51	<0.025	41.5468	<0.05	0.002	1.6	0.0029	0	0.12	<1	<5	6.85	<1
1693	12/14/2010	Kitchen Faucet	Upland	Yes	3.14	<0.025	134.8829		0			0	0.77	<1	<5	6.79	1.6
1694	12/18/2010	Kitchen Faucet	Valley	Yes	2.11	< .025	160.16						2.5	< 1	< 5	6.83	

Table S.8. Groundwater Quality Data for 1701 "Pre-Drill" Water Well Samples from Susquehanna County, Pennsylvania

Water Well ID	Sample Date	Sampling Port	Topo-graphic Location	Gas Extraction Area (Within 1 km of Active Gas Well)	Dissolved Oxygen - Field (mg/L)	Ethane ug/L	Hardness (mg/L)	Iron (mg/L)	Lead (mg/L)	Magnesium (mg/L)	Manganese (mg/L)	Mercury (mg/L)	Methane ug/L	Nitrate as N (mg/L)	Oil Grease (mg/L)	pH - Field (SU)	Potassium (mg/L)
1695	10/29/2009	Pressure Tank	Upland	Yes	7.98	0.2		< .05		9.9	< .025		2.7			7.22	
1696	11/6/2010	Kitchen Faucet	Upland			<0.025	3.3075	< .05	< .0003	< .5	< .0025	< .0002	0.61	<1	<5		< .5
1697	3/19/2009		Upland	Yes		< .025		< .05		7.03	0.03		0.49				
1698	3/25/2009		Upland			<0.025		<0.05		1.79	<0.025		0.25				
1699	3/12/2009		Upland			<0.025		0.109		5.24	<0.025		0.52				
1700	4/5/2010	Kitchen Sink, Main House	Upland		6.07	<0.025		<0.05		9.06	<0.025		0.41			7.33	
1701	6/2/2011		Valley			< 26	110	< .05	< 05	4.5	0.033	< .0002	< 26	< .05			2.2

Table S.8. Groundwater Quality Data for 1701 "Pre-Drill" Water Well Samples from Susquehanna County, Pennsylvania

Water Well ID	Sample Date	Sampling Port	Topo-graphic Location	Gas Extraction Area (Within 1 km of Active Gas Well)	Selenium (mg/L)	Silver (mg/L)	Sodium (mg/L)	Strontium (mg/L)	Sulfate (mg/L)	Sulfide (mg/L)	TDS (mg/L)	Thallium (mg/L)	TSS (mg/L)	Turbidity (NTU)	Vanadium (mg/L)
1	6/3/2011		Valley		<0.005	<0.001	2.2	0.17	11	<3	69		<4	<0.85	
2	5/27/2010	Pressure Tank	Valley				50.7	0.47			237			18.3	
3	6/29/2011		Upland		<0.005	<0.001	7.7	0.17	12	<3	140		<4	<0.85	
4	6/29/2011		Upland		< .005	< .001	8.9	0.086	< 1	< 3	61		9.2	8.9	
5	6/17/2010	Outside Spigot	Upland	Yes						< 1					
6	6/17/2010	Kitchen Sink	Valley							< 1					
7	6/17/2010	Kitchen Sink	Upland							< 1					
8	4/10/2010	Pressure Tank	Upland					0.032		1	40	< .002	< 2		< .01
9	4/10/2010	Basement At Pressure Tank	Upland					0.209		1	95	< .002	< 2		< .01
10	4/10/2010	Pressure Tank	Valley					0.177		3	60	< .002	< 2		< .01
11	4/11/2010	Outside Hydrant Directly From Well	Upland					0.038		< 1	90	< .002	2.4		< .01
12	4/11/2010		Valley					0.057		< 1	75	< .002	< 2		< .01
13	4/11/2010	Kitchen Sink	Valley					0.079		2	145	< .002	< 2		< .01
14	4/11/2010	Kitchen Sink	Upland	Yes				0.27		< 1	105	< .002	< 2		< .01
15	4/11/2010	Pressures Tank	Upland					0.065		< 1	155	< .002	< 2		< .01
16	4/11/2010	Kitchen Sink	Valley					0.786		5	155	< .002	< 2		< .01
17	4/11/2010	Kitchen Sink	Upland					0.522		< 1	125	< .002	2.8		< .01
18	4/11/2010	Outside Hydrant Direct From Well	Upland					0.273		1	135	< .002	< 2		< .01
19	4/15/2010	Outside Hydrant	Upland					0.227		< 1	95	< .002	< 2		< .01
20	4/15/2010	Kitchen Sink	Upland					0.354		< 1	180	< .002	< 2		< .01
21	4/15/2010	Outside Hydrant	Valley					0.077		1	160	< .002	2.8		< .01
22	4/15/2010	Pressure Tank	Valley					0.146		< 1	75	< .002	< 2		< .01
23	4/19/2010	Laundry Room Sink	Upland					0.17		< 1	140	< .002	< 2		< .01

Table S.8. Groundwater Quality Data for 1701 "Pre-Drill" Water Well Samples from Susquehanna County, Pennsylvania

Water Well ID	Sample Date	Sampling Port	Topo-graphic Location	Gas Extraction Area (Within 1 km of Active Gas Well)	Selenium (mg/L)	Silver (mg/L)	Sodium (mg/L)	Strontium (mg/L)	Sulfate (mg/L)	Sulfide (mg/L)	TDS (mg/L)	Thallium (mg/L)	TSS (mg/L)	Turbidity (NTU)	Vanadium (mg/L)
24	4/19/2010	Hydrant At Well Head	Valley					0.076		2	193	< .002	9.6		< .01
25	4/19/2010	Diversion Ditch	Upland	Yes				0.026		1	60		8.4	2	
26	4/22/2010	Kitchen Sink	Valley					0.246		1	273	< .002	< 2		< .01
27	4/22/2010	Kitchen Sink	Upland	Yes				0.374		< 1	153	< .002	< 2		< .01
28	4/22/2010	Basement Sink	Upland					0.264		1	207	< .002	< 2		< .01
29	6/24/2010	Pond	Upland							< 1					
30	6/24/2010	Pressure Tank	Upland												
31	5/14/2011		Upland		< .005	< .001	13	0.082	19	< 3	410		< 4	6.5	
32	6/24/2010	Kitchen Sink	Valley							< 1					
33	6/24/2010	Outside Spigot	Upland					0.435		< 1	100	< .002	< 2		< .01
34	4/25/2010	Bathroom Faucet	Upland	Yes				0.112		< 1	80	< .002	3.2		< .01
35	4/25/2010		Upland	Yes				0.311		< 1	160		< 2	< 1	
36	4/24/2010	Outside Spigot	Valley					0.477		1	507	< .002	2.5		< .01
37	4/24/2010	Kitchen Sink	Upland	Yes				0.025		5	120	< .002	< 2		< .01
38	4/24/2010		Upland					0.32		1	220	< .002	< 2		< .01
39	4/24/2010	Kitchen Bar Sink	Upland					0.253		1	313	< .002	< 2		< .01
40	4/24/2010	Outside Spigot	Upland					0.549		< 1	127	< .002	< 2		< .01
41	4/24/2010	Outside Spigot	Upland					0.329		< 1	193	< .002	< 2		< .01
42	4/29/2010	Outside Spigot	Upland					0.406		< 1	100	< .002	< 2		< .01
43	4/29/2010	Spring Culvert	Upland					< .025		< 1	35	< .002	< 2		< .01
44	4/29/2010	Basement At Pressure Tank	Valley					0.063		< 1	100	< .002	< 2		< .01
45	7/2/2010	Kitchen Sink	Valley												
46	7/2/2010	Bathroom Sink	Valley												
47	7/2/2010	Kitchen Sink	Valley												
48	7/2/2010	Kitchen Sink	Valley												
49	7/2/2010	Kitchen Sink	Upland												

Table S.8. Groundwater Quality Data for 1701 "Pre-Drill" Water Well Samples from Susquehanna County, Pennsylvania

Water Well ID	Sample Date	Sampling Port	Topo-graphic Location	Gas Extraction Area (Within 1 km of Active Gas Well)	Selenium (mg/L)	Silver (mg/L)	Sodium (mg/L)	Strontium (mg/L)	Sulfate (mg/L)	Sulfide (mg/L)	TDS (mg/L)	Thallium (mg/L)	TSS (mg/L)	Turbidity (NTU)	Vanadium (mg/L)
50	7/2/2010	Kitchen Sink	Upland												
51	7/2/2010	Kitchen Sink	Valley												
52	7/6/2010	Kitchen Sink	Upland					0.542		< 1	150	< .002	< 2		< .01
53	7/6/2010	Garage	Valley					0.435		< 1	120	< .002	3.5		< .01
54	7/6/2010	Kitchen Sink	Valley					0.11		< 1	100	< .002	< 2		< .01
55	7/6/2010	Kitchen Sink	Upland					0.529		< 1	150	< .002	< 2		< .01
56	7/6/2010	Kitchen Sink	Valley					0.957		1	205	< .002	< 2		< .01
57	7/6/2010		Valley					1.25		< 1	100	< .002	2		< .01
58	7/6/2010	Kitchen Sink	Valley					0.451		< 1	125	< .002	< 2		< .01
59	7/6/2010	Basement At Pressure Tank	Valley					0.11		< 1	95	< .002	< 2		< .01
60	7/6/2010		Upland					0.05		< 1	120	< .002	< 2		< .01
61	7/7/2010	Kitchen Sink	Valley					0.407		< 1	170	< .002	< 2		< .01
62	7/7/2010	Spring Head	Valley					0.038		< 1	80	< .002	< 2		< .01
63	7/7/2010	Kitchen Sink	Valley					0.045		1	135	< .002	< 2		< .01
64	7/7/2010	Kitchen Sink	Valley					1.45		< 1	135	< .002	2		< .01
65	7/7/2010	Kitchen Sink	Valley					0.141		< 1	80	< .002	< 2		< .01
66	7/7/2010	Basement Pressure Tank	Valley					0.258		< 1	90	< .002	< 2		< .01
67	7/7/2010	Kitchen Sink	Valley					1.45		< 1	145	< .002	< 2		< .01
68	7/8/2010	Basement Sink	Valley					0.621		< 1	135	< .002	< 2		< .01
69	7/8/2010	Kitchen Sink	Upland					0.141		< 1	120	< .002	< 2		< .01
70	7/8/2010	Kitchen Sink	Valley					0.331		< 1	160	< .002	< 2		< .01
71	7/9/2010	Kitchen Sink	Valley					0.409		< 1	165	< .002	< 2		< .01
72	7/9/2010	Kitchen Sink - Debonis Property	Valley					0.083		< 1	165	< .002	< 2		< .01
73	7/7/2010	Kitchen Sink	Valley					0.665		< 1	140	< .002	< 2		< .01
74	7/7/2010	Outside Garage Spigot	Valley					0.063		< 1	90	< .002	< 2		< .01

Table S.8. Groundwater Quality Data for 1701 "Pre-Drill" Water Well Samples from Susquehanna County, Pennsylvania

Water Well ID	Sample Date	Sampling Port	Topo-graphic Location	Gas Extraction Area (Within 1 km of Active Gas Well)	Selenium (mg/L)	Silver (mg/L)	Sodium (mg/L)	Strontium (mg/L)	Sulfate (mg/L)	Sulfide (mg/L)	TDS (mg/L)	Thallium (mg/L)	TSS (mg/L)	Turbidity (NTU)	Vanadium (mg/L)
75	7/8/2010	Kitchen Sink	Valley					0.065		< 1	180	< .002	< 2		< .01
76	7/7/2010	Kitchen Sink	Valley					0.94		< 1	130	< .002	< 2		< .01
77	7/7/2010	Kitchen Sink	Valley					0.076		< 1	45	< .002	< 2		< .01
78	7/8/2010	Kitchen Sink	Valley					0.418		5	100	< .002	2.8		< .01
79	7/8/2010	Kitchen Sink	Upland					0.665		< 1	140	< .002	< 2		< .01
80	7/8/2010	Kitchen Sink	Valley					0.405		< 1	170	< .002	< 2		< .01
81	7/8/2010	Kitchen Sink	Valley					0.316		< 1	135	< .002	< 2		< .01
82	7/9/2010		Valley	Yes				0.08		< 1	145	< .002	< 2		< .01
83	7/9/2010	Kitchen Sink	Valley					0.076		< 1	480	< .002	< 2		< .01
84	7/9/2010	Kitchen Sink	Valley					0.5		< 1	130	< .002	< 2		< .01
85	7/9/2010	Kitchen Sink	Upland					0.239		< 1	170	< .002	2		< .01
86	7/9/2010	Kitchen Sink	Valley					1.9		< 1	140	< .002	< 2		< .01
87	7/9/2010		Valley					0.067		< 1	55	< .002	23.6		< .01
88	7/10/2010		Valley					2.15		5	430	< .002	< 2		< .01
89	7/8/2010	Pressure Tank	Upland					0.041		< 1	65	< .002	< 2		< .01
90	5/20/2010	Kitchen Sink	Valley					0.095		< 1		< .002			< .01
91	7/11/2010	Kitchen Sink	Valley					0.284		< 1	170	< .002	< 2		< .01
92	7/11/2010	Barn Hose	Valley					0.061		<1	105	<0.002	7.6		<0.01
93	7/8/2010	Kitchen Sink	Upland	Yes				0.254		< 1	110	< .002	< 2		< .01
94	4/8/2009		Upland					0.299		<1	68		<2		
95	7/9/2010		Valley					0.678		< 1	130	< .002	< 2		< .01
96	7/10/2010	Kitchen Sink	Valley					0.666		< 1	165	< .002	< 2		< .01
97	7/10/2010	Kitchen Sink	Upland					< .025		< 1	65	< .002	4		< .01
98	7/12/2010	Artesian Well	Valley					0.09		< 1	100	< .002	< 2		< .01
99	7/12/2010		Valley					0.788		4	235	< .002	< 2		< .01
100	7/12/2010	Kitchen Sink	Valley					0.905		< 1	165	< .002	< 2		< .01
101	7/12/2010		Valley					2.59		< 1	725	< .002	< 2		< .01
102	7/12/2010		Valley					0.088		< 1	115	< .002	< 2		< .01
103	7/11/2010	Kitchen Sink	Valley	Yes				0.667		< 1	160	< .002	< 2		< .01

Table S.8. Groundwater Quality Data for 1701 "Pre-Drill" Water Well Samples from Susquehanna County, Pennsylvania

Water Well ID	Sample Date	Sampling Port	Topo-graphic Location	Gas Extraction Area (Within 1 km of Active Gas Well)	Selenium (mg/L)	Silver (mg/L)	Sodium (mg/L)	Strontium (mg/L)	Sulfate (mg/L)	Sulfide (mg/L)	TDS (mg/L)	Thallium (mg/L)	TSS (mg/L)	Turbidity (NTU)	Vanadium (mg/L)
104	7/12/2010	Kitchen Sink	Valley					0.071		< 1	70	< .002	< 2		< .01
105	7/13/2010		Upland					0.491		< 1	150	< .002	< 2		< .01
106	7/12/2010	Kitchen Sink	Valley					0.5		5		< .002			< .01
107	7/17/2010	Kitchen Sink	Valley					0.797		< 1	125	< .002	< 2		< .01
108	7/17/2010	Kitchen Sink	Valley					0.354		< 1	175	< .002	< 2		< .01
109	7/17/2010	Kitchen Sink	Valley					0.096		< 1	80	< .002	< 2		< .01
110	7/11/2010		Upland					0.208		< 1	130	< .002	< 2		< .01
111	7/13/2010	Kitchen Sink	Valley					0.24		< 1	85	< .002	< 2		< .01
112	7/13/2010	Kitchen Sink	Valley					0.541		< 1	155	< .002	< 2		< .01
113	7/12/2010		Valley					0.461		< 1	160	< .002	< 2		< .01
114	7/25/2011	Kitchen Sink	Upland												
115	8/17/2011	Bathroom Sink	Valley												
116	7/5/2011		Valley												
117	3/12/2009	Barn	Valley					1.32		< 1	140		< 2		
118	2/20/2009	Basement	Upland					0.034		< 1	58		< 2		
119	7/1/2010		Valley					0.151		< 1	95	< .002	< 2		< .01
120	7/1/2010		Upland					2.16		< 1	< 145	< .002	< 2		< .01
121	2/20/2009		Upland					0.086		1.2	340		< 2		
122	8/3/2010	Hand Dug Well	Upland					0.086		< 1	265	< .002	5.2		< .01
123	2/20/2009	Basement	Valley					0.169		< 1	180		< 2		
124	8/7/2010	Kitchen Sink	Upland					1.27		< 1	195	< .002	< 2		< .01
125	8/7/2010	Hand Dug Well	Upland					0.15		< 1	155	< .002	2		< .01
126	8/8/2010	Pressure Tank	Valley					0.638		<1	190	<0.002	<2		<0.01
127	8/8/2010	Kitchen Sink	Valley					< .025		< 1	265	< .002	< 2		< .01
128	8/8/2010	Bathroom Sink	Valley					< .025		< 1	260	< .002	< 2		< .01
129	8/8/2010	Kitchen Sink	Upland					0.072		< 1	125	< .002	< 2		< .01
130	7/30/2010	Bathroom Sink	Valley	Yes				0.303		< 1	110	< .002	< 2		< .01
131	8/13/2010	Outside Spigot	Upland					0.072		< 1	110	< .002	< 2		< .01
132	7/20/2010	Kitchen Sink	Valley					0.945		< 1	225	< .002	< 2		< .01
133	7/20/2010	Heefer Barn	Upland					0.284		< 1	165	< .002	< 2		< .01
134	8/11/2010	House #2	Valley					0.308		< 1	155	< .002	< 2		< .01
135	4/15/2009		Upland					0.141		< 1	40		< 2		

Table S.8. Groundwater Quality Data for 1701 "Pre-Drill" Water Well Samples from Susquehanna County, Pennsylvania

Water Well ID	Sample Date	Sampling Port	Topo-graphic Location	Gas Extraction Area (Within 1 km of Active Gas Well)	Selenium (mg/L)	Silver (mg/L)	Sodium (mg/L)	Strontium (mg/L)	Sulfate (mg/L)	Sulfide (mg/L)	TDS (mg/L)	Thallium (mg/L)	TSS (mg/L)	Turbidity (NTU)	Vanadium (mg/L)
136	8/11/2010	Main House Kitchen Sink	Valley					0.332		< 1	150	< .002	< 2		0.01
137	8/19/2010		Upland	Yes	< .005	< .005	7.43	0.299	12	< 1	107		< 2	< 1	
138	8/19/2010	Barn Well Milkhouse Sink	Upland	Yes	< .005	< .005	6.26	0.175	16	< 1	170		< 2	< 1	
139	6/24/2009	Basement Pressure Tank	Valley					0.047		< 1	135		< 2		
140	8/19/2010	Pressure Tank	Upland		< .005	< .005	15.5	1.54	7	< 1	123		< 2	< 1	
141	8/19/2010	Kitchen Sink	Upland	Yes	< .005	< .005	4.85	0.134	9	< 1	210		2	< 1	
142	8/19/2010	Spigot Under Mobile Home	Upland	Yes	< .005	< .005	8.4	0.68	7	< 1	210		< 2	< 1	
143	8/20/2010	Laundry Room Sink	Upland		< .005	< .005	1.37	1.28	7	< 1	505		< 2	< 1	
144	6/24/2009	Well Head	Valley					< .025		< 1	20		< 2		
145	6/24/2009	Kitchen Sink	Valley					0.209		< 1	< 2		< 2		
146	8/26/2010	Outside Spigot	Upland		< .005	< .005	14.4	0.135	7	< 1	190		< 2	< 1	
147	7/29/2010		Valley					2.19		< 1	395	< .002	< 2		< .01
148	7/29/2010	Kitchen Sink	Upland	Yes				0.276		10	140	< .002	44		< .01
149	8/28/2010	Pressure Tank	Valley		< .0005	< .005	19	0.625	20	< 1	225		< 2	< 1	
150	8/28/2010	Outside Spigot	Valley		< .005	< .005	29.1	0.195	14	< 1	205		3	6	
151	9/2/2010	Kitchen Sink	Upland		< .005	< .005	16.8	0.162	14	1	208		< 2	< 1	
152	6/13/2011	Kitchen Sink	Valley												
153	9/10/2010	Kitchen Faucet	Valley		< .005	< .01	41.8	0.849	11	< 1	155		< 2	< 1	
154	7/20/2010	Tenant House Kitchen Sink	Upland					0.602		< 1	140	< .002	< 2		< .01
155	2/27/2010	Kitchen Sink	Upland					0.067		< 1	116		< 2	< 1	
156	2/22/2010	Basement At Pressure Tank	Valley					0.494		< 1	112		< 2	< 1	

Table S.8. Groundwater Quality Data for 1701 "Pre-Drill" Water Well Samples from Susquehanna County, Pennsylvania

Water Well ID	Sample Date	Sampling Port	Topo-graphic Location	Gas Extraction Area (Within 1 km of Active Gas Well)	Selenium (mg/L)	Silver (mg/L)	Sodium (mg/L)	Strontium (mg/L)	Sulfate (mg/L)	Sulfide (mg/L)	TDS (mg/L)	Thallium (mg/L)	TSS (mg/L)	Turbidity (NTU)	Vanadium (mg/L)
157	9/17/2010	Kitchen Sink	Valley		< .005	< .002	98.4	0.722	< 5	4	230		< 2	< 1	
158	1/10/2010	Basement Pressure Tank	Upland					0.14		< 1	160		< 2	< 1	
159	10/3/2010	Kitchen Sink	Valley	Yes	< .005	< .0005	16.9	0.256	15	< 1	184		< 2	1	
160	1/31/2011	Kitchen Sink	Upland		< .002	< .001	6.1	0.69	9	< 1	104		< 2	< 1	
161	4/8/2009	Laundry Sink	Upland					0.345		< 1	200		< 2		
162	10/7/2010	Kitchen Sink	Upland		< .005	< .005	18.4	1.11		< 1					
163	10/7/2010	Kitchen Sink	Valley	Yes	< .005	< .005	11.7	0.119		< 1					
164	10/7/2010		Valley	Yes	< .005	< .005	14.4	0.158	14	< 1	145		< 2	6	
165	4/8/2009	Boiler Room	Upland					0.411		< 1	80		< 2		
166	7/14/2011	Kitchen Sink	Upland												
167	7/14/2011	Kitchen Sink	Upland												
168	10/8/2010	Kitchen Faucet	Valley	Yes	< .005	< .005	11.2	0.137	10	< 1	140		< 2	< 1	
169	10/8/2010	Outside Spigot	Valley	Yes	< .005	< .005	12.9	0.319	17	< 1	144		< 2	3	
170	7/22/2010	Pressure Tank	Upland	Yes				1.09		< 1	150	< .002	< 2		< .01
171	4/8/2009	Career & Tech Bldg Pressure Tank	Upland					0.437		< 1	150		< 2		
172	4/8/2009	Boiler Room	Upland					0.335		< 1	150		< 2		
173	10/1/2010	Kitchen Sink	Valley	Yes	< .005	< .005	93.8	0.52	< 5	< 1	268		< 2	< 1	
174	11/5/2009	Pressure Tank	Upland	Yes				0.334		2	132		< 2		
175	7/14/2011	Spigot On Side Of House	Valley												
176	7/14/2011	Kitchen Sink	Valley												
177	7/14/2011	Kitchen Sink	Valley												
178	7/14/2011	Kitchen Sink	Upland												
179	7/14/2011	Kitchen Sink	Upland												

Table S.8. Groundwater Quality Data for 1701 "Pre-Drill" Water Well Samples from Susquehanna County, Pennsylvania

Water Well ID	Sample Date	Sampling Port	Topo-graphic Location	Gas Extraction Area (Within 1 km of Active Gas Well)	Selenium (mg/L)	Silver (mg/L)	Sodium (mg/L)	Strontium (mg/L)	Sulfate (mg/L)	Sulfide (mg/L)	TDS (mg/L)	Thallium (mg/L)	TSS (mg/L)	Turbidity (NTU)	Vanadium (mg/L)
180	10/10/2010	Kitchen Sink Rental Property	Valley	Yes	< .005	< .005	40	0.1	28	< 1	295		< 2	< 1	
181	4/15/2009	Pressure Tank	Valley	Yes				0.435		< 1	120		< 2		
182	10/10/2010	Kitchen Sink	Valley	Yes	< .005	< .005	9	0.259	15	< 1	135		< 2	< 1	
183	11/4/2010	Kitchen Sink	Valley		< .002	< .001	7.4	0.16	13	< 1	104		< 2	< 1	
184	11/4/2010	Kitchen Sink	Upland		< .002	< .001	18.2	0.23	10	< 1	128		< 2	1	
185	3/12/2009	Basement Before Filter	Upland	Yes						< 1	236		< 2		
186	10/10/2010	Outside Spigot	Valley	Yes	< .005	< .005	9.7	0.288	12	< 1	140		< 2	1	
187	11/4/2010	Pressure Tank	Valley		< .002	< .001	69.1	0.23	7	< 1	164		< 2	< 1	
188	11/6/2010	Kitchen Sink	Valley	Yes	< .002	< .001	18.7	0.056	9	< 1	148		< 2	1	
189	11/6/2010	Outside Spigot	Valley		< .002	< .001	11.3	0.26	12	< 1	116		< 2	< 1	
190	11/7/2010	Kitchen Sink	Valley	Yes	< .002	< .001	28.4	0.12	36	< 1	264		< 2	< 1	
191	11/7/2010	Pressure Tank	Valley	Yes	< .002	< .001	18.5	0.32	17	1	196		< 2	1	
192	11/7/2010	Kitchen Sink	Valley	Yes	< .002	< .001	36.7	0.12	24	< 1	228		< 2	< 1	
193	10/13/2010	Kitchen Faucet	Valley	Yes	< .005	< .005	9.24	0.294	13	< 1	175		< 2	1	
194	10/13/2010	Pressure Tank	Valley	Yes	< .005	< .005	14	0.492	15	< 1	170		< 2	1	
195	10/13/2010	Pressure Tank	Valley	Yes	< .005	< .005	22.6	0.077	21	< 1	210		< 2	< 1	
196	10/13/2010	Kitchen Sink	Valley	Yes	< .005	< .005	8.27	0.333	17	< 1	165		< 2	< 1	
197	10/11/2010	Kitchen Sink	Valley	Yes	< .005	< .005	8.7	0.071	8	< 1	170		< 2	< 1	
198	10/18/2010	Bathroom Sink	Valley		<0.005	<0.005	15.9	0.096	25	<1	240		4.6	<1	
199	10/2/2010	Kitchen Sink Faucet	Upland		< .005	< .005	19.3	0.513	< 5	< 1	148		< 2	1	

Table S.8. Groundwater Quality Data for 1701 "Pre-Drill" Water Well Samples from Susquehanna County, Pennsylvania

Water Well ID	Sample Date	Sampling Port	Topo-graphic Location	Gas Extraction Area (Within 1 km of Active Gas Well)	Selenium (mg/L)	Silver (mg/L)	Sodium (mg/L)	Strontium (mg/L)	Sulfate (mg/L)	Sulfide (mg/L)	TDS (mg/L)	Thallium (mg/L)	TSS (mg/L)	Turbidity (NTU)	Vanadium (mg/L)
200	10/2/2010	Pressure Tank	Valley		< .005	< .005	13.8	0.26	9	< 1	136		< 2	< 1	
201	10/2/2010	Bathroom Faucet	Valley		< .005	< .005	13.5	0.3	8	< 1	112		< 2	1	
202	10/15/2010	Kitchen Sink	Valley	Yes	< .005	< .005	11.5	0.134	14	< 1	115		< 2	< 1	
203	4/19/2009		Valley	Yes				0.039		<1	184		<2		
204	4/4/2008		Upland					0.031		<1	40		<2		
205	10/19/2010	Kitchen Sink	Valley	Yes	< .005	< .005	54.6	0.739	16	< 1	160		< 2	0.128	
206	10/19/2010	Kitchen Sink	Valley	Yes	< .005	< .005	8.62	0.252	18	< 1	160		< 2	1	
207	10/19/2010	Hand Dug Well	Valley	Yes	< .005	< .005	2.06	0.028	9	1	65		< 2	3	
208	10/20/2010	Kitchen Sink	Valley	Yes	< .005	< .005	9.05	0.196	14	< 1	175		< 2	1	
209	10/26/2010	Kitchen Sink	Valley	Yes	< .005	< .005	10.1	0.143	15	< 1	180		< 2	2	
210	10/26/2010	Outside Spigot	Valley		< .005	< .005	14	0.448	13	< 1	250		< 2	6	
211	7/14/2011	Kitchen Sink	Upland												
212	8/1/2011	Kitchen Sink	Valley												
213	10/26/2010		Valley	Yes	< .005	< .005	18.4	0.106	16	< 1	210		< 2	1	
214	10/28/2010	Kitchen Sink	Valley	Yes	< .005	< .005	15.5	0.254	14	< 1	200		< 2	1	
215	7/17/2010	Outside Spigot	Valley					0.98		< 1	135	< .002	< 2		< .01
216	10/28/2010	Basement Pressure Tank	Valley		< .005	< .005	56.1	1.44	12	< 1	220		< 2	< 1	
217	10/28/2010	Pressure Tank	Upland		< .005	< .005	19.4	0.645	14	< 1	220		< 2	< 1	
218	10/28/2010	Bar Sink	Upland		< .005	< .005	3.35	0.043	13	< 1	150		< 2	< 1	
219	10/29/2010	Outside Spigot	Valley	Yes	< .005	< .005	56.3	1.14	12	< 1	300		< 2	< 1	
220	10/29/2010		Valley		< .005	< .005	15.8	1.11	12	< 1	180		< 2	< 1	
221	10/29/2010	Outside Sink	Valley		< .005	< .005	54.5	0.078	14	< 1	240		< 2	< 1	
222	10/29/2010	Kitchen Faucet	Upland		< .005	< .005	15.2	0.119	14	< 1	190		< 2	< 1	
223	11/10/2010	Kitchen Sink	Upland		< .002	< .001	16.8	1	9	< 1	180		< 2	1	

Table S.8. Groundwater Quality Data for 1701 "Pre-Drill" Water Well Samples from Susquehanna County, Pennsylvania

Water Well ID	Sample Date	Sampling Port	Topo-graphic Location	Gas Extraction Area (Within 1 km of Active Gas Well)	Selenium (mg/L)	Silver (mg/L)	Sodium (mg/L)	Strontium (mg/L)	Sulfate (mg/L)	Sulfide (mg/L)	TDS (mg/L)	Thallium (mg/L)	TSS (mg/L)	Turbidity (NTU)	Vanadium (mg/L)
224	11/10/2010	Kitchen Sink	Valley		< .002	< .001	7.14	0.27	9	1	128		< 2	1	
225	9/28/2010		Upland		< .005	< .005	17.4	0.679	22	< 1	200		2.8	6	
226	11/11/2010	Outside Spigot	Valley	Yes	< .002	< .001	13.9	0.49	16	< 1	193		< 2	1	
227	11/11/2010	Kitchen Faucet	Valley	Yes	< .002	< .001	9.6	0.13	5	< 1	127		< 2	< 1	
228	9/28/2010	Kitchen Sink	Valley		< .005	< .005	3.6	0.06	10	< 1	100		< 2	< 1	
229	11/17/2010	Kitchen Sink	Valley	Yes	< .002	< .001	18.2	0.71	18	< 1	173		< 2	< 1	
230	11/17/2010	Kitchen Sink	Upland		< .002	< .001	14.1	0.8	30	< 10	187		22	21	
231	11/17/2010	Kitchen Sink	Upland		< .002	< .001	1.86	0.021	7	< 1	40		< 2	< 1	
232	12/4/2010	Kitchen Sink	Valley	Yes	< .005	< .005	30.1	0.489	35	2	260		< 2	< 1	
233	12/4/2010	Kitchen Sink	Valley	Yes	< .005	< .005	1.69	0.054	10	< 1	213		< 2	2	
234	12/4/2010	Basement At Pressure Tank	Valley	Yes	< .005	< .005	13.8	0.11	15	< 1	173		< 2	1	
235	12/4/2010	Kitchen Sink	Valley	Yes	< .005	< .005	16.7	0.2	20	< 1	227		< 2	1	
236	12/5/2010	Kitchen Sink	Upland	Yes	< .005	< .005	1.96	0.031	11	< 1	73		< 2	1	
237	12/5/2010	Kitchen Faucet	Valley	Yes	< .005	< .005	14.3	0.485	16	< 1	173		< 2	< 1	
238	12/5/2010	Barn At Ceiling Spigot	Valley	Yes	< .005	< .005	22.5	1.35	9	< 1	160		< 2	< 1	
239	2/17/2009	Kitchen Sink	Valley	Yes				0.358		< 1	139		< 2		
240	12/8/2010	Kitchen Sink	Valley	Yes	< .002	< .001	21	0.7	20	< 1	140		< 2	< 1	
241	12/8/2010	Kitchen Sink	Upland		< .002	< .001	12	0.034	8	< 1	20		< 2	1	
242	12/8/2010	Kitchen Sink	Valley	Yes	< .002	< .001	1.6	0.019	7	< 1	40		< 2	1	
243	12/8/2010	Hand Dug Well	Upland		< .002	< .001	21	0.075	17	< 1	127		< 2	1	
244	2/17/2009	Kitchen Sink	Valley	Yes				0.149		< 1	106		< 2		
245	4/4/2009	Well Head	Valley	Yes				0.34		< 1	120		14		

Table S.8. Groundwater Quality Data for 1701 "Pre-Drill" Water Well Samples from Susquehanna County, Pennsylvania

Water Well ID	Sample Date	Sampling Port	Topo-graphic Location	Gas Extraction Area (Within 1 km of Active Gas Well)	Selenium (mg/L)	Silver (mg/L)	Sodium (mg/L)	Strontium (mg/L)	Sulfate (mg/L)	Sulfide (mg/L)	TDS (mg/L)	Thallium (mg/L)	TSS (mg/L)	Turbidity (NTU)	Vanadium (mg/L)
246	12/11/2010	Kitchen Sink	Valley		< .002	< .001	17	0.92	9	1	53		< 2	< 1	
247	12/11/2010		Upland		< .002	< .001	22	0.097	8	< 1	107		< 2	< 1	
248	12/11/2010	Kitchen Sink	Valley		< .002	< .001	8.4	1.1	6	< 1	100		< 2	1	
249	3/16/2009	Basement At Pressure Tank	Valley	Yes				0.078		< 1	113		< 2		
250	12/17/2010	Pressure Tank	Upland		0.002	0.001	4.2	0.033	14	< 5	93		2.8	2	
251	12/17/2010	Pressure Tank	Upland		< .002	< .001	5.2	0.096	14	< 5	73		23	16	
252	12/18/2010	Basement Pressure Tank	Valley	Yes	< .002	< .001	23	0.15	22	< 5	200		4	1	
253	6/24/2009	Kitchen Sink	Upland	Yes				0.269		< 1	140		< 2		
254	12/18/2010	Kitchen Sink	Valley	Yes	< .002	< .001	22	0.62	< 5	< 5	153		3.2	3	
255	3/19/2009	Kitchen Sink	Valley	Yes				0.875		< 1	200		< 2		
256	3/19/2009	Kitchen Sink	Upland	Yes				0.127		< 1	193		< 2		
257	12/14/2010	Kitchen Sink	Valley	Yes	< .002	< .001	13	0.49	13	< 1	147		< 2	< 1	
258	3/4/2009	Kitchen Sink	Upland					0.073		< 1	150		< 2		
259	12/14/2010	Hydrant	Valley	Yes	< .002	< .001	10	0.52	14	< 25	153		62	30	
260	12/14/2010	Kitchen Sink	Valley	Yes	< .002	< .001	19	0.74	8	< 1	153		2	< 1	
261	12/14/2010	Bathtub Faucet	Valley	Yes	< .002	< .001	17	0.29	19	< 1	207		2.8	< 1	
262	2/23/2011		Valley	Yes	< .002	< .001	8.9	0.2	10	< 1	145		< 2	1	
263	12/19/2010		Valley	Yes	< .002	< .001	13	0.29	17	< 1	246.7		< 2	< 1	
264	12/19/2010	Kitchen Sink	Valley		< .002	< .001	39	0.89	7	2	133		< 2	< 1	
265	12/19/2010	Kitchen Faucet	Valley		< .002	< .001	4.1	0.05	9	< 1	53		< 2	< 1	

Table S.8. Groundwater Quality Data for 1701 "Pre-Drill" Water Well Samples from Susquehanna County, Pennsylvania

Water Well ID	Sample Date	Sampling Port	Topo-graphic Location	Gas Extraction Area (Within 1 km of Active Gas Well)	Selenium (mg/L)	Silver (mg/L)	Sodium (mg/L)	Strontium (mg/L)	Sulfate (mg/L)	Sulfide (mg/L)	TDS (mg/L)	Thallium (mg/L)	TSS (mg/L)	Turbidity (NTU)	Vanadium (mg/L)
266	12/19/2010	School Art Studio Bathroom Sink	Valley	Yes	< .002	< .001	11	0.27	19	< 1	153		< 2	2	
267	2/26/2009		Upland					0.078		<1	80		<2		
268	1/27/2011	Downstairs Kitchen Sink	Valley		< .002	< .001	79	0.14	9	< 1	284		< 2	1	
269	1/27/2011	Kitchen Sink	Valley		< .002	< .001	5.5	0.22	8	< 1	56		< 2	< 1	
270	1/27/2011	Kitchen Sink	Valley		< .002	< .001	4.9	0.12	8	< 1	72		< 2	1	
271	3/4/2009	Kitchen Sink	Upland					0.42		< 1	172		< 2		
272	2/11/2011	Kitchen Sink	Upland	Yes	< .002	< .001	17	1.8	22	< 1	180		< 2	< 1	
273	2/11/2011	Kitchen Sink	Upland		< .002	< .001	8	0.19	17	< 1	340		< 2	< 1	
274	2/11/2011	Kitchen Sink	Upland		< .002	< .001	60	0.79	5	< 1	153		< 2	< 1	
275	2/11/2011	Kitchen Sink	Upland	Yes	< .002	< .001	31	1.7	35	< 1	380		< 2	< 1	
276	2/11/2011	In Milkhouse, At Bottom Of Pressure Tank	Upland		< .002	< .001	17	1.7	23	< 1	153		< 2	< 1	
277	2/4/2011	Pressure Tank	Valley	Yes	< .002	0.001	17	0.24	14	< 1	140		< 2	5	
278	2/8/2011	Bathtub	Valley		< .002	< .001	6.1	0.29	20	< 1	167		< 2	2	
279	3/4/2009	Kitchen Sink	Upland					0.063		< 1	166		< 2		
280	2/8/2011	Kitchen Sink	Upland		< .002	< .001	6.8	0.13	22	1	180		< 2	< 1	
281	10/19/2010	Kitchen Sink	Valley	Yes	< .005	< .005	8.55	0.553	17	< 1	155		< 2	< 1	
282	2/8/2011	Kitchen Sink, Small Spigot On Right Hand Side Of S	Valley		< .002	< .001	8.4	0.28	8	1	127		< 2	1	
283	2/11/2011	Kitchen Sink	Upland	Yes	< .002	< .001	110	0.033	29	< 1	347		< 2	< 1	
284	2/11/2011	Kitchen Sink	Upland		< .002	< .001	6.6	0.66	11	< 1	127		< 2	1	

Table S.8. Groundwater Quality Data for 1701 "Pre-Drill" Water Well Samples from Susquehanna County, Pennsylvania

Water Well ID	Sample Date	Sampling Port	Topo-graphic Location	Gas Extraction Area (Within 1 km of Active Gas Well)	Selenium (mg/L)	Silver (mg/L)	Sodium (mg/L)	Strontium (mg/L)	Sulfate (mg/L)	Sulfide (mg/L)	TDS (mg/L)	Thallium (mg/L)	TSS (mg/L)	Turbidity (NTU)	Vanadium (mg/L)
285	2/8/2011	Rental (Adam Vibbard) Kitchen Sink	Upland		< .002	< .001	15	1.4	11	< 1	160		< 2	< 1	
286	2/8/2011	Kitchen Sink	Upland		< .002	< .001	11	0.094	8	< 1	120		< 2	< 1	
287	1/13/2011	Kitchen Sink	Valley	Yes				0.67		< 1	125		< 2		
288	2/15/2011	Pressure Tank	Upland		< .002	< .001	17	1.3	9	< 1	133		< 2	1	
289	2/15/2011	Laundry Room Faucet	Upland		< .002	< .001	39	1.3	6	< 1	140		< 2	< 1	
290	3/4/2009	Kitchen Sink	Valley					0.058		< 1	216		< 2		
291	2/14/2011	Kitchen Sink	Upland		< .002	< .001	13	1.4	11	< 1	207		< 2	< 1	
292	2/15/2011	Kitchen Faucet	Valley		< .002	< .001	100	0.15	16	< 1	420		2	1	
293	2/14/2011	Barn Milkhouse Sink	Valley		< .002	< .001	8.6	0.14	10	< 1	147		< 2	1	
294	2/15/2011	Milkhouse Faucet	Upland	Yes	< .002	< .001	25	1.1	15	< 1	160		< 2	1	
295	2/15/2011	1St Floor Kitchen Faucet	Upland	Yes	< .002	< .001	8.3	0.32	11	< 1	140		< 2	< 1	
296	2/17/2011	Pressure Tank In Basement	Upland	Yes	< .002	< .001	24	0.83	45	< 1	233		< 2	1	
297	3/4/2009	1St Floor Mens Room Sink	Valley					0.126		< 1	146		< 2		
298	3/21/2011		Valley		0.013	< .001	458	7.6	1.4	< 3	1390		< 4	2.8	
299	3/21/2011		Upland	Yes	< .005	< .001	8.4	0.27	11.2	< 3	304		< 4	4.8	
300	3/22/2011		Valley	Yes	< .005	< .001	7	0.48	11.4	< 3	145		< 4	< 1	
301	3/4/2009	Kitchen Sink	Valley					0.195		< 1	142		< 2		
302	12/10/2010	Garage Faucet	Upland	Yes	< .002	< .001	23	0.31	12	< 1	207		< 2	1	
303	12/10/2010	Kitchen Faucet	Upland	Yes	< .002	< .001	9.4	0.58	10	< 1	93		< 2	< 1	

Table S.8. Groundwater Quality Data for 1701 "Pre-Drill" Water Well Samples from Susquehanna County, Pennsylvania

Water Well ID	Sample Date	Sampling Port	Topo-graphic Location	Gas Extraction Area (Within 1 km of Active Gas Well)	Selenium (mg/L)	Silver (mg/L)	Sodium (mg/L)	Strontium (mg/L)	Sulfate (mg/L)	Sulfide (mg/L)	TDS (mg/L)	Thallium (mg/L)	TSS (mg/L)	Turbidity (NTU)	Vanadium (mg/L)
304	12/10/2010	Kitchen Sink	Valley	Yes	< .002	< .001	13	0.21	16	< 1	113		< 2	< 1	
305	12/10/2010		Upland	Yes	< .002	< .001	22	0.21	11	< 1	140		< 2	4	
306	12/10/2010	Pressure Tank	Upland		< .002	< .001	25	0.1	17	< 1	193		< 2	< 1	
307	12/10/2010	Kitchen Faucet	Valley		< .002	< .001	1.5	0.028	12	< 1	53		< 2	< 1	
308	12/14/2010	Kitchen Sink	Valley	Yes	< .002	< .001	9.6	0.22	11	< 1	153		< 2	< 1	
309	3/12/2009	Kitchen Sink Faucet	Upland					0.057		< 1	92		< 2		
310	12/20/2010	Kitchen Sink	Upland		< .002	< .001	18	1.3	22	< 1	220		< 2	< 1	
311	12/20/2010	Pressure Tank	Valley		< .002	< .001	28	0.67	13	2	207		5.6	3	
312	12/20/2010	Basement At Pressure Tank	Valley		< .002	< .001	24	0.4	16	< 1	247		< 2	1	
313	12/20/2010	Kitchen Sink	Upland		< .002	< .001	4.8	0.21	11	< 1	147		< 2	2	
314	12/20/2010	Kitchen Sink	Upland		< .002	< .001	5.9	0.21	10	< 1	153		< 2	1	
315	3/4/2009	Pressure Tank In Basement	Upland					0.143		< 1	168		< 2		
316	12/20/2010	Bathtub Faucet	Upland		< .002	< .001	6.7	0.47	11	< 1	133		< 2	1	
317	4/15/2009	Horse Barn Faucet	Valley	Yes				0.109		< 1	170		< 2		
318	1/6/2011	Kitchen Sink	Upland	Yes	< .002	< .001	34	0.29	9	< 1	132		< 2	< 1	
319	4/19/2009	Kitchen Sink Faucet	Valley	Yes				0.172		< 1	180		< 2		
320	4/8/2009	Barn After Filter	Valley					0.053		< 1	96		2.8		
321	4/12/2011	Cwt Of Kitchen Sink	Valley		< .005	< .001	7	0.12	9	< 3	177		< 4	< 1	
322	4/13/2011	Well	Upland	Yes	< .005	< .001	17.2	0.5	4.5	< 3	162		< 4	2.1	
323	4/13/2011	Kitchen Sink	Valley	Yes	< .005	< .001	3.2	0.064	10.2	< 3	112		< 4	< 1	
324	4/13/2011	Kitchen Sink Cwt	Upland		< .005	< .001	10.1	0.34	13.2	1	162		9.2	3	

Table S.8. Groundwater Quality Data for 1701 "Pre-Drill" Water Well Samples from Susquehanna County, Pennsylvania

Water Well ID	Sample Date	Sampling Port	Topo-graphic Location	Gas Extraction Area (Within 1 km of Active Gas Well)	Selenium (mg/L)	Silver (mg/L)	Sodium (mg/L)	Strontium (mg/L)	Sulfate (mg/L)	Sulfide (mg/L)	TDS (mg/L)	Thallium (mg/L)	TSS (mg/L)	Turbidity (NTU)	Vanadium (mg/L)
325	4/13/2011	Kitchen Sink Cwt	Upland		< .005	< .001	27	0.61	18.8	0.72	178		< 4	< 1	
326	3/12/2009	Basement Before Filter	Upland	Yes				0.085		< 1	104		< 2		
327	4/14/2011	Farmhouse Kitchen Sink Cwt	Valley		< .005	< .001	6.9	1	23.3	< 3	205		< 4	< 1	
328	4/15/2011	Kitchen Sink Cwt	Upland		< .005	< .001	7.8	0.15	15	< 3	132		< 4	2.4	
329	4/18/2011	Pressure Tank In Basement	Valley		< .005	< .001	46.6	0.058	14.9	< 3	233		< 4	< 1	
330	4/18/2011	Kitchen Sink	Upland		< .005	< .001	8.4	0.34	28.8	< 3	170		< 4	< 1	
331	4/19/2011	Kitchen Faucet	Upland		< .005	< .001	16.3	0.19	22.7	< 3	165		< 4	< 1	
332	4/22/2011	Mary Steele Kitchen Sink	Valley	Yes	< .005	< .001	7.1	0.049	12	< 3	150		< 4	< 1	
333	4/26/2011	Kicthen Sink	Valley	Yes	< .005	< .001	8.4	0.26	14.5	< 3	154		< 4	< 1	
334	4/27/2011	Well 2	Upland	Yes	< .005	< .001	13.5	0.32	27.5	< 3	204		< 4	< 1	
335	4/28/2011	Basement Pressure Tank	Valley	Yes	< .005	< .001	14.7	0.094	13.7	< 3	150		15.2	14.9	
336	4/28/2011	Pressure Tank In Basement On E Side Of House	Valley	Yes	< .005	< .001	12.6	0.12	12.3	< 3	162		< 4	< 1	
337	5/2/2011		Upland	Yes	<0.005	<0.001	10.1	1.3	13.8	<3	130		<4	<1	
338	5/2/2011		Upland	Yes	<0.005	<0.001	24.5	0.071	14	<3	108		<4	<1	
339	5/3/2011	Kitchen Sink	Valley	Yes	< .005	< .001	10.4	0.27	15.9	< 3	212		< 4	< 1	
340	5/4/2011	Kitchen Cwt	Valley	Yes	< .005	< .001	6.8	0.17	9.8	< 3	248		< 4	< 1	
341	5/4/2011	Kitchen Sink	Valley	Yes	< .005	< .001	6.5	0.19	15.1	< 3	218		< 4	< 1	
342	5/4/2011	Outside Hose Bib On The East Side Of Home	Valley	Yes	< .005	< .001	16.4	0.25	12.8	< 3	337		< 4	2.2	
343	5/4/2011	Outdoor Spigot	Valley	Yes	< .005	< .001	9.9	0.2	12.9	< 3	242		< 4	< 1	
344	2/26/2009	Kitchen Sink	Upland					0.225		< 1	80		< 2		

Table S.8. Groundwater Quality Data for 1701 "Pre-Drill" Water Well Samples from Susquehanna County, Pennsylvania

Water Well ID	Sample Date	Sampling Port	Topo-graphic Location	Gas Extraction Area (Within 1 km of Active Gas Well)	Selenium (mg/L)	Silver (mg/L)	Sodium (mg/L)	Strontium (mg/L)	Sulfate (mg/L)	Sulfide (mg/L)	TDS (mg/L)	Thallium (mg/L)	TSS (mg/L)	Turbidity (NTU)	Vanadium (mg/L)
345	5/4/2011	School Art Studio Bathroom Sink	Valley	Yes	< .005	< .001	11.4	0.29	15.3	< 3	284		< 4	3.2	
346	5/4/2011	Pressure Tank	Valley	Yes	< .005	< .001	11.1	0.21	14.2	< 3	282		6	21.4	
347	5/5/2011	Kitchen Sink Cwt	Upland	Yes	< .005	< .001	44.6	0.057	29.9	0.64	248		< 4	< 1	
348	5/5/2011	Kitchen Sink	Valley	Yes	< .005	< .001	29.2	0.12	24.5	< 3	227		< 4	< 1	
349	5/5/2011	Pressure Tank	Valley	Yes	< .005	< .001	17.9	0.078	20.3	< 3	257		4	10.6	
350	5/5/2011	Kitchen Sink Cwt	Valley	Yes	< .005	< .001	11.4	0.067	15	< 3	175		< 4	< 1	
351	5/5/2011	Pressure Tank	Valley	Yes	< .005	< .001	21.1	0.21	21.5	< 3	225		< 4	10.1	
352	5/6/2011	Pressure Tank	Valley	Yes	< .005	< .001	23.5	0.11	14.8	< 3	219		< 4	1.3	
353	5/6/2011	Kitchen Sink	Valley	Yes	< .005	< .001	9.1	0.55	17.4	< 3	144		< 4	< 1	
354	5/7/2011	Pressure Tank In Basement	Valley	Yes	< .005	< .001	12.7	0.43	12.8	< 3	217		4.8	24.1	
355	5/7/2011	Bathroom Sink Ctw	Valley	Yes	< .005	< .001	17.1	0.2	20.4	< 3	256		< 4	< 1	
356	5/7/2011	Backroom Sink	Valley	Yes	< .005	< .001	11.8	0.19	12.7	< 3	220		< 4	3	
357	5/7/2011	Pressure Tank In Basement	Upland	Yes	< .005	< .001	19.9	0.19	21.4	< 3	222		< 4	< 1	
358	5/7/2011	Kitchen Sink	Valley	Yes	< .005	< .001	14.4	0.14	16.1	< 3	574		< 4	2.4	
359	5/7/2011	Pressure Tank	Valley	Yes	< .005	< .001	10.6	0.08	21	< 3	233		< 4	< 1	
360	5/9/2011	Pressure Tank	Valley	Yes	< .005	< .001	13.9	0.092	16.7	< 3	190		< 4	3.2	
361	5/9/2011	Basement Pressure Tank	Valley	Yes	< .005	< .001	15.8	0.17	18.8	< 3	179		8.4	28.9	
362	5/9/2011	Well	Valley	Yes	< .005	< .001	20.6	0.041	20.4	< 3	147		< 4	< 1	
363	5/9/2011	Kitchen Sink	Valley	Yes	< .005	< .001	14.9	0.23	19.9	< 3	242		< 4	< 1	

Table S.8. Groundwater Quality Data for 1701 "Pre-Drill" Water Well Samples from Susquehanna County, Pennsylvania

Water Well ID	Sample Date	Sampling Port	Topo-graphic Location	Gas Extraction Area (Within 1 km of Active Gas Well)	Selenium (mg/L)	Silver (mg/L)	Sodium (mg/L)	Strontium (mg/L)	Sulfate (mg/L)	Sulfide (mg/L)	TDS (mg/L)	Thallium (mg/L)	TSS (mg/L)	Turbidity (NTU)	Vanadium (mg/L)
364	5/9/2011	Outdoor Hose Bib On South Side Of Gas Station	Valley	Yes	< .005	< .001	20.1	0.14	16	< 3	215		< 4	1.9	
365	5/9/2011	Outside Spigot	Valley	Yes	< .005	< .001	12.2	0.39	15.9	< 3	171		< 4	< 1	
366	5/9/2011	Pressure Tank	Upland	Yes	< .005	< .001	11.6	0.058	15.8	< 3	161		6	31.3	
367	5/9/2011	Pressure Tank	Valley	Yes	< .005	< .001	18.3	0.075	21.1	< 3	208		< 4	< 1	
368	5/9/2011	Outside Faucet	Valley	Yes	< .005	< .001	19.3	0.14	15.1	< 3	187		< 4	5.9	
369	5/10/2011	Kitchen Sink	Upland	Yes	< .005	< .001	12	0.053	8.3	< 3	91		< 4	3.3	
370	5/10/2011	Inside Faucet	Valley	Yes	< .005	< .001	16.4	0.11	12.7	< 3	235		< 4	3	
371	5/10/2011	Inside Faucet	Valley	Yes	< .005	< .001	15.9	0.14	13.6	< 3	224		< 4	1.2	
372	5/11/2011		Valley	Yes	< .005	< .001	13.8	0.028	9.6	< 3	144		6.4	5.9	
373	5/11/2011	Pressure Tank In Basement	Valley	Yes	< .005	< .001	24	0.23	15.1	0.96	245		< 4	6.2	
374	5/11/2011		Valley	Yes	< .005	< .001	19.1	0.081	13.2	1.2	247		< 4	2.8	
375	5/11/2011	Kitchen Sink	Valley	Yes	< .005	< .001	11.2	0.044	12.4	0.88	171		< 4	1	
376	5/11/2011	Outside Bib	Valley	Yes	< .005	< .001	16.1	0.33	19.8	0.64	234		< 4	1.2	
377	5/11/2011	Kitchen Sink	Valley	Yes	< .005	< .001	12.4	0.19	17.6	0.96	233		< 4	3.6	
378	5/11/2011	Pressure Tank In Basement	Valley	Yes	< .005	< .001	21.5	0.09	17.8	0.72	266		< 4	1.2	
379	5/12/2011		Valley	Yes	< .005	< .001	9.9	0.26	15.8	< 3	188		< 4	1.5	

Table S.8. Groundwater Quality Data for 1701 "Pre-Drill" Water Well Samples from Susquehanna County, Pennsylvania

Water Well ID	Sample Date	Sampling Port	Topo-graphic Location	Gas Extraction Area (Within 1 km of Active Gas Well)	Selenium (mg/L)	Silver (mg/L)	Sodium (mg/L)	Strontium (mg/L)	Sulfate (mg/L)	Sulfide (mg/L)	TDS (mg/L)	Thallium (mg/L)	TSS (mg/L)	Turbidity (NTU)	Vanadium (mg/L)
380	5/12/2011	Outside Faucet	Valley	Yes	< .005	< .001	17	0.053	12.9	< 3	203		< 4	< 1	
381	5/12/2011	Basement At Pressure Tank	Valley	Yes	< .005	< .001	11.6	0.1	15.6	< 3	164		< 4	< 1	
382	5/12/2011	Pressure Tank	Valley	Yes	< .005	< .001	178	0.19	19.3	< 3	1090		< 4	3.5	
383	5/12/2011	Kitchen Sink	Valley	Yes	< .005	< .001	19.9	0.13	16.3	< 3	227		< 4	1.3	
384	5/13/2011	Pressure Tank Rental Property	Valley	Yes	< .005	< .001	27	0.065	21.5	< 3	241		< 4	< 1	
385	5/13/2011	Pressure Tank	Valley	Yes	< .005	< .001	15.1	0.26	19.6	< 3	191		< 4	< 1	
386	5/13/2011	Kitchen Sink	Valley	Yes	< .005	< .001	19.8	0.053	10.3	< 3	176		< 4	< 1	
387	5/13/2011	Outside Faucet	Valley	Yes	< .005	< .001	27.5	0.47	25.4	< 3	262		< 4	3	
388	1/31/2011	Kitchen Sink	Valley		< .002	< .001	10	0.17	9	< 1	140		< 2	< 1	
389	1/8/2011	Kitchen Sink	Upland	Yes	< .002	< .001	8.9	0.68	8	< 1	167		< 2	< 1	
390	1/8/2011	Kitchen Sink	Valley		< .002	< .001	2.4	0.044	6	< 1	80		< 2	2	
391	1/4/2011	Kitchen Sink	Valley	Yes	< .002	< .001	6.4	0.038	12	< 1	133		< 2	1	
392	2/17/2011	Outside Spigot	Upland		< .002	< .001	82	0.19	5	< 1	187		< 2	< 1	
393	2/22/2011	Kitchen Sink	Upland	Yes	< .002	< .001	14	1.3	19	< 1	227		< 2	< 1	
394	2/22/2011	Pressure Tank In Barn, Old Milkhouse	Upland		< .002	< .001	13	0.45	24	< 1	173		< 2	1	
395	2/22/2011	Rental Kitchen Sink	Upland		< .002	< .001	34	0.63	34	< 1	273		< 2	1	
396	1/8/2011	Basement At Pressure Tank	Valley	Yes	< .002	< .001	4.7	0.24	11	< 1	120		6.4	4	
397	1/31/2011	Kitchen Sink	Valley		< .002	< .001	2.4	0.039	< 5	< 1	72		35	< 1	

Table S.8. Groundwater Quality Data for 1701 "Pre-Drill" Water Well Samples from Susquehanna County, Pennsylvania

Water Well ID	Sample Date	Sampling Port	Topo-graphic Location	Gas Extraction Area (Within 1 km of Active Gas Well)	Selenium (mg/L)	Silver (mg/L)	Sodium (mg/L)	Strontium (mg/L)	Sulfate (mg/L)	Sulfide (mg/L)	TDS (mg/L)	Thallium (mg/L)	TSS (mg/L)	Turbidity (NTU)	Vanadium (mg/L)
398	1/9/2011	Hand Dug Well West Of House	Upland	Yes	< .002	< .001	2	0.022	13	< 1	44		11.6	12	
399	1/4/2011	Pressure Tank	Upland		< .002	< .001	8.4	0.22	9	< 1	166		< 2	< 1	
400	3/12/2009	Kitchen Sink	Valley					0.543		< 1	128		2		
401	1/31/2011	Kitchen Sink	Upland	Yes	< .002	< .001	9.4	0.11	< 5	< 1	180		3	< 1	
402	2/22/2011	Upstairs Kitchen Sink	Upland		< .002	< .001	18	0.16	9	< 1	113		< 2	1	
403	1/31/2011	Kitchen Sink	Upland	Yes	< .002	< .001	4.7	0.094	< 5	< 1	140		< 2	< 1	
404	1/31/2011	Kitchen Sink	Upland		< .002	< .001	71	0.64	< 5	< 1	208		< 2	< 1	
405	1/4/2011	Hand Dug Well	Upland		< .002	< .001	1	0.01	7	< 1	60		2.4	1	
406	2/22/2011	Basement Of Rental At Pressure Tank	Upland		< .002	< .001	34	0.21	< 5	< 1	227		3.2	3	
407	1/8/2011	Basement At Pressure Tank	Upland		< .002	< .001	4.6	0.086	9	< 1	128		4.4	< 1	
408	1/8/2011	Kithcen Sink	Upland		< .002	< .001	4.7	0.053	10	< 1	120		< 2	< 1	
409	1/14/2011	Laundry Room Sink	Upland		< .002	< .001	7	0.2	9	< 1	148		2.8	< 1	
410	3/12/2009	Kitchen Sink	Valley					0.32		< 1	136		< 2		
411	1/31/2011	Kitchen Sink	Valley		< .002	< .001	28	0.48	< 5	< 1	148		< 2	< 1	
412	2/22/2011	Breakroom Sink- Lower Level	Upland		< .002	< .001	18	1.2	13	< 1	213		< 2	< 1	
413	3/16/2009	Kitchen Sink	Valley					0.584		< 1	173		< 2		
414	1/8/2011	Kitchen Sink	Valley	Yes	< .002	< .001	22	0.089	10	< 1	176		< 2	7	
415	1/4/2011	Ktichen Sink	Upland		< .002	< .001	3.3	0.11	8	< 1	86.7		< 2	2	
416	1/31/2011	Kitchen Sink	Valley	Yes	< .002	< .001	4.8	0.22	< 5	< 1	104		< 2	< 1	
417	1/10/2011	Hand Dug Well	Upland		< .002	< .001	2.3	0.0027	15	< 1	44		2.4	1	
418	2/22/2011	Kitchen Sink	Valley		< .002	< .001	15	1.2	< 5	< 1	73		< 2	1	

Table S.8. Groundwater Quality Data for 1701 "Pre-Drill" Water Well Samples from Susquehanna County, Pennsylvania

Water Well ID	Sample Date	Sampling Port	Topo-graphic Location	Gas Extraction Area (Within 1 km of Active Gas Well)	Selenium (mg/L)	Silver (mg/L)	Sodium (mg/L)	Strontium (mg/L)	Sulfate (mg/L)	Sulfide (mg/L)	TDS (mg/L)	Thallium (mg/L)	TSS (mg/L)	Turbidity (NTU)	Vanadium (mg/L)
419	1/10/2011	Kitchen Sink	Valley		< .002	< .001	21	1.2	15	< 1	232		< 2	1	
420	1/4/2011		Upland		< .002	< .001	3.9	0.18	10	< 1	140		< 2	< 1	
421	2/17/2011	Kitchen Faucet On 2Nd Floor	Upland		< .002	< .001	4.6	0.35	12	< 1	87		< 2	< 1	
422	1/4/2011		Upland	Yes	< .002	< .001	19	1.7	< 5	< 1	186		< 2	< 1	
423	1/7/2011	Milkhouse Faucet	Valley	Yes	< .002	< .001	7.9	0.35	16	< 1	213		< 2	< 1	
424	1/7/2011	Kitchen Sink Rental	Upland	Yes	< .002	< .001	8.8	1	11	< 1	126		< 2	3	
425	10/8/2009		Upland					0.068		< 2	85		9.5		
426	3/13/2009	Kitchen Sink	Valley					1.44		< 1	132		< 2		
427	3/12/2009	Kitchen Sink	Upland					0.044		< 1	24		< 2		
428	6/24/2009		Upland	Yes				< .025		< 1	165		< 2		
429	3/4/2009		Upland					0.063		<1	166		<2		
430	6/13/2011	Barn Spigot	Upland												
431	6/14/2011	Kitchen Sink Overlooking Road	Upland												
432	6/14/2011	Pressure Tank	Valley												
433	6/16/2011	Kitchen Sink On Island In Kitchen	Valley												
434	6/16/2011	Kitchen Sink	Valley												
435	6/16/2011	In Barn Milkhouse- At Sink	Valley												
436	6/16/2011	Outside Spigot- Next To Porch At End Of Driveway	Upland												
437	6/16/2011	Kitchen Sink On Lake Road Side Of House Under Window	Valley												
438	6/17/2011	Kitchen Sink	Valley												

Table S.8. Groundwater Quality Data for 1701 "Pre-Drill" Water Well Samples from Susquehanna County, Pennsylvania

Water Well ID	Sample Date	Sampling Port	Topo-graphic Location	Gas Extraction Area (Within 1 km of Active Gas Well)	Selenium (mg/L)	Silver (mg/L)	Sodium (mg/L)	Strontium (mg/L)	Sulfate (mg/L)	Sulfide (mg/L)	TDS (mg/L)	Thallium (mg/L)	TSS (mg/L)	Turbidity (NTU)	Vanadium (mg/L)
439	6/23/2011	Kitchen Sink	Valley												
440	6/23/2011	Pressure Tank	Upland												
441	6/23/2011	Pressure Tank	Valley												
442	6/24/2011		Valley												
443	6/24/2011	Kitchen Sink	Upland												
444	6/24/2011	Kitchen Sink	Valley												
445	6/24/2011	Kitchen Sink	Upland												
446	6/24/2011	Shed Spigot	Valley												
447	6/13/2011	Kitchen Faucet	Upland												
448	6/15/2011	Kitchen Sink Near Rear Of House	Upland												
449	6/15/2011	Kitchen Sink On South Side Of House	Upland												
450	6/15/2011	Kitchen Sink On Wall Closest To Red Barn Behind House	Upland												
451	6/15/2011	Kitchen Sink	Valley												
452	6/15/2011	Outside Spigot- On The North Side Of The Residence	Upland												
453	6/15/2011		Upland												
454	3/25/2009	Kitchen Sink	Upland					0.86		< 1	200		< 2		
455	6/27/2011	Greenhouse Sink	Valley												
456	4/15/2009	Kitchen Sink	Valley					0.103		< 1	84		< 2		
457	4/19/2009	Basement Pressure Tank	Valley					0.048		< 1	135		< 2		
458	6/13/2011	Outside Hose Bib	Valley	Yes	< .005	< .001	3.2	0.051	15	< 3	110		< 4	1.7	

Table S.8. Groundwater Quality Data for 1701 "Pre-Drill" Water Well Samples from Susquehanna County, Pennsylvania

Water Well ID	Sample Date	Sampling Port	Topo-graphic Location	Gas Extraction Area (Within 1 km of Active Gas Well)	Selenium (mg/L)	Silver (mg/L)	Sodium (mg/L)	Strontium (mg/L)	Sulfate (mg/L)	Sulfide (mg/L)	TDS (mg/L)	Thallium (mg/L)	TSS (mg/L)	Turbidity (NTU)	Vanadium (mg/L)
459	6/13/2011	Outside Cold Water Hose Bib	Upland	Yes	< .005	< .001	15	0.16	15	< 3	200		< 4	< .85	
460	6/16/2011	Bailed Directly From Well	Upland		< .005	< .001	3.3	0.028	3.4	< 3	93		< 4	15	
461	2/26/2009	Mary Steele Kitchen Sink	Valley					0.047		< 1	70		< 2		
462	6/16/2011		Upland		<0.005	<0.001	8.9	0.33	10	<3	130		<4	<0.85	
463	6/20/2011	Kitchen Sink Cwt On Nw Side Of House	Upland		< .005	< .001	4.4	0.054	15	< 3	120		< 4	< .85	
464	6/20/2011	Well	Upland		< .005	< .001	6.8	0.038	10	< 3	110		< 4	1.5	
465	1/25/2009		Valley					0.075		< 1	< 2		< 2		
466	6/20/2011		Upland	Yes	< .005	< .001	31	1.1	14	< 3	190		< 4	< .85	
467	6/21/2011	At Pressure Tank In Basement	Upland	Yes	< .005	< .001	10	0.037	9.7	< 3	130		< 4	< .85	
468	6/22/2011	Inside Hose Bib Located In The Garage	Valley		<0.005	<0.001	3.1	0.096	12	<3	93		<2	<0.85	
469	4/1/2009		Valley					0.233		< 1	112		< 2		
470	6/22/2011	Outside Hose	Valley		< .005	< .001	13	0.3	10	< 3	160		< 4	1.8	
471	6/22/2011	Well	Valley	Yes	< .005	< .001	25	0.65	30	< 3	220		< 4	< .85	
472	6/22/2011	Kitchen Sink Cwt	Valley		< .005	< .001	6	0.22	4.2	< 3	120		< 4	0.95	
473	6/23/2011	Pipe In Basement	Upland		< .005	< .001	0.93	0.021	4.1	< 3	110		< 4	0.97	
474	4/1/2009		Valley					0.105		< 1	208		< 2		
475	6/23/2011	Outside Spigot	Valley		< .005	< .001	22	0.64	15	< 3	160		< 4	< .85	
476	4/15/2009	Kitchen Sink	Upland					2.16		< 1	248		< 2		
477	6/24/2011	Kitchen Sink	Valley		< .005	< .001	10	0.57	8.4	< 3	150		53	11	
478	6/24/2011	Outside Hose Bib On The W End Of Home	Valley	Yes	< .005	< .001	22	0.1	15	< 3	220		6.8	< .85	
479	4/15/2009	Kitchen Sink	Upland					2.16		< 1	196		< 2		

Table S.8. Groundwater Quality Data for 1701 "Pre-Drill" Water Well Samples from Susquehanna County, Pennsylvania

Water Well ID	Sample Date	Sampling Port	Topo-graphic Location	Gas Extraction Area (Within 1 km of Active Gas Well)	Selenium (mg/L)	Silver (mg/L)	Sodium (mg/L)	Strontium (mg/L)	Sulfate (mg/L)	Sulfide (mg/L)	TDS (mg/L)	Thallium (mg/L)	TSS (mg/L)	Turbidity (NTU)	Vanadium (mg/L)
480	3/16/2009	Pressure Tank	Upland					0.458		< 1	113		< 2		
481	6/27/2011	Sample Collected From An Outside Spigot Located On The North Side Of The House.	Valley		< .005	< .001	13	0.39	8.2	< 3	140		< 4	< .85	
482	6/27/2011	Kitchen Sink	Valley		< .005	< .001	11	0.83	11	< 3	110		< 4	< .85	
483	6/28/2011	Dip Sample Directly From Well. Well Is Locatedon The South End Of Property Inside Of Electric Fence. It Is Covered With Metal Sheeting And An Old Car Hood Frame.	Upland		< .005	< .001	41	0.088	3	< 3	260		< 4	1.4	
484	6/28/2011	Kitchen Sink Cwt	Valley		< .005	< .001	17	0.38	12	< 3	160		< 4	< .85	
485	6/29/2011	Well	Upland		< .005	< .001	2.4	0.096	10	< 3	70		69	60	
486	6/29/2011	Laundry Room Sink	Valley		< .005	< .001	15	0.95	14	< 3	150		< 4	< .85	
487	6/29/2011	Dug Well	Valley		< .005	< .001	2.8	0.029	13	< 3	69		< 4	< .85	
488	6/29/2011		Valley		< .005	< .001	3	0.046	15	< 3	97		< 4	< .85	
489	6/30/2011	Pressure Tank	Upland		< .005	< .001	2.8	0.044	14	< 3	120		< 4	14	
490	7/1/2011	Outside Hose Bib	Valley		< .005	< .001	19	0.5	9.3	< 3	170		< 4	3.2	
491	2/12/2009		Valley					0.224		< 1	264		< 2		
492	5/7/2009		Valley					0.248		<1	124		15.6		
493	4/26/2009		Valley					0.468		< 1	910		2.8		

Table S.8. Groundwater Quality Data for 1701 "Pre-Drill" Water Well Samples from Susquehanna County, Pennsylvania

Water Well ID	Sample Date	Sampling Port	Topo-graphic Location	Gas Extraction Area (Within 1 km of Active Gas Well)	Selenium (mg/L)	Silver (mg/L)	Sodium (mg/L)	Strontium (mg/L)	Sulfate (mg/L)	Sulfide (mg/L)	TDS (mg/L)	Thallium (mg/L)	TSS (mg/L)	Turbidity (NTU)	Vanadium (mg/L)
494	7/5/2011	Kitchen Sink	Valley		< .005	< .001	6.2	0.36	13	< 3	120		< 4	0.91	
495	7/5/2011	Samples Collected By Dipping 1 Inch Plastic Bailer Down The Well.	Upland		< .005	< .001	4.1	0.11	7.6	< 3	120		770	1700	
496	7/5/2011	Outside Hose Bib On South Of House	Valley		< .005	< .001	5.1	0.24	3.9	< 3	160		< 4	3.6	
497	7/6/2011	Pressure Tank In Basement	Upland		< .005	< .001	6.5	0.33	11	< 3	150		< 4	< .85	
498	7/8/2011	Outside Faucet	Upland		< .005	< .001	34	< .005	8.4	< 3	140		< 4	1.2	
499	5/14/2011	Kitchen Sink	Valley	Yes	< .005	< .001	18	0.63	18	< 3	310		< 4	< 1	
500	5/3/2009	Bathroom Sink	Upland					0.391		< 1	144		< 2		
501	5/19/2011	Outside Spigot	Valley	Yes	< .005	< .001	27	0.055	20	< 3	160		< 4	< .85	
502	5/25/2011	Pressure Tank	Valley	Yes	< .005	< .001	25	0.41	19	< 3	250		< 4	7.3	
503	5/26/2011	Pressure Tank	Valley	Yes	< .005	< .001	31	0.11	32	< 3	330		< 4	< .85	
504	10/26/2009		Valley					0.155		< 1	128		< 2		
505	5/26/2011	Outside Hose Bib Cold	Valley	Yes	< .005	< .001	14	0.2	16	< 3	270		< 4	< .85	
506	10/26/2009		Valley	Yes				0.316		< 1	147		< 2		
507	6/1/2011	Outside Hose	Upland		< .005	< .001	24	0.061	12	< 3	230		15	66	
508	6/1/2011	Pressure Tank	Valley		< .005	< .001	12	0.14	10	< 3	180		< 4	< .85	
509	6/2/2011	Pressure Tank In Old Creamery	Upland		< .005	< .001	5	0.08	9.5	< 3	88		< 4	< 1	
510	10/26/2009	Pressure Tank	Valley	Yes				0.525		< 1	113		< 2		
511	6/6/2011	Dug Well 20 Feet From Se Corner Of House	Upland		< .005	< .001	9.7	0.11	26	< 3	250		< 4	3.5	
512	10/26/2009	Pressure Tank	Upland	Yes				0.148		< 1	207		< 2		

Table S.8. Groundwater Quality Data for 1701 "Pre-Drill" Water Well Samples from Susquehanna County, Pennsylvania

Water Well ID	Sample Date	Sampling Port	Topo-graphic Location	Gas Extraction Area (Within 1 km of Active Gas Well)	Selenium (mg/L)	Silver (mg/L)	Sodium (mg/L)	Strontium (mg/L)	Sulfate (mg/L)	Sulfide (mg/L)	TDS (mg/L)	Thallium (mg/L)	TSS (mg/L)	Turbidity (NTU)	Vanadium (mg/L)
513	6/13/2011	Kitchen Sink	Valley												
514	6/13/2011	Hand Dug Well	Upland												
515	6/13/2011	Main Kitchen Sink	Upland												
516	6/13/2011	Basement Pressure Tank	Valley												
517	6/13/2011	Exterior Faucet On South Side Of House Between Two Green Garage Doors	Upland												
518	6/14/2011	Kitchen Sink	Valley												
519	6/14/2011	Kitchen Sink	Upland												
520	6/14/2011	Pressure Tank	Upland												
521	6/14/2011	Kitchen Sink	Valley												
522	10/26/2009	Basement At Pressure Tank	Upland	Yes				0.385		< 1	160		< 2		
523	6/14/2011	Kitchen Faucet	Valley												
524	6/14/2011	Kitchen Sink	Valley												
525	6/14/2011	Kitchen Sink	Upland												
526	6/14/2011	Kitchen Sink	Valley												
527	6/14/2011	Pressure Tank	Valley												
528	6/15/2011		Upland												
529	6/15/2011	Kitchen Sink	Upland												
530	6/15/2011	Spigot At Well House	Upland												
531	6/15/2011	Kitchen Sink	Upland												
532	6/15/2011	Kitchen Sink	Upland												
533	6/15/2011	Kitchen Sink	Upland												

Table S.8. Groundwater Quality Data for 1701 "Pre-Drill" Water Well Samples from Susquehanna County, Pennsylvania

Water Well ID	Sample Date	Sampling Port	Topo-graphic Location	Gas Extraction Area (Within 1 km of Active Gas Well)	Selenium (mg/L)	Silver (mg/L)	Sodium (mg/L)	Strontium (mg/L)	Sulfate (mg/L)	Sulfide (mg/L)	TDS (mg/L)	Thallium (mg/L)	TSS (mg/L)	Turbidity (NTU)	Vanadium (mg/L)
534	6/16/2011	Kitchen Sink	Upland												
535	6/16/2011	Pressure Tank	Valley												
536	6/16/2011	Kitchen Sink Overlooking Blanding Road	Upland												
537	6/16/2011	Kitchen Sink On South Side Of House	Upland												
538	6/16/2011	Kitchen Sink	Upland												
539	6/16/2011	Kitchen Sink	Upland												
540	6/16/2011	Wellhead From Hose	Upland												
541	6/16/2011	Kitchen Sink	Valley												
542	6/17/2011	Kitchen Sink	Upland												
543	6/17/2011	Pressure Tank In Basement On Tingley Lake Side Of Road	Valley												
544	6/20/2011	Pressure Tank	Valley												
545	6/21/2011	Kitchen Sink	Valley												
546	6/21/2011	Kitchen Sink	Upland												
547	6/21/2011	Kitchen Sink	Upland												
548	4/26/2009	Kitchen Sink	Valley					0.271		< 1	85		< 2		
549	6/21/2011	Kitchen Sink	Upland												
550	6/21/2011		Valley												
551	6/22/2011	Kitchen Sink	Upland												
552	6/22/2011	Basement Pressure Tank	Valley												
553	6/22/2011	Kitchen Sink	Upland												
554	6/22/2011	Kitchen Sink	Valley												

Table S.8. Groundwater Quality Data for 1701 "Pre-Drill" Water Well Samples from Susquehanna County, Pennsylvania

Water Well ID	Sample Date	Sampling Port	Topo-graphic Location	Gas Extraction Area (Within 1 km of Active Gas Well)	Selenium (mg/L)	Silver (mg/L)	Sodium (mg/L)	Strontium (mg/L)	Sulfate (mg/L)	Sulfide (mg/L)	TDS (mg/L)	Thallium (mg/L)	TSS (mg/L)	Turbidity (NTU)	Vanadium (mg/L)
555	6/22/2011		Upland												
556	6/22/2011	Kitchen Sink	Upland												
557	6/23/2011	Pressure Tank	Valley												
558	6/23/2011	Pressure Tank	Valley												
559	6/23/2011	Kitchen Sink	Valley												
560	6/23/2011	Kitchen Sink	Valley												
561	6/23/2011		Valley												
562	6/23/2011		Upland												
563	10/27/2009	Kitchen Sink - Ann'S House	Valley	Yes				0.134		< 1	170		< 2		
564	6/24/2011	Spigot	Upland												
565	6/24/2011	Kitchen Sink	Upland												
566	6/24/2011	Kitchen Sink	Upland												
567	6/24/2011	Kitchen Sink	Upland												
568	6/24/2011	Kitchen Sink	Valley												
569	6/24/2011	Kitchen Sink	Valley												
570	6/24/2011	Kitchen Sink	Upland												
571	6/24/2011	Kitchen Sink	Upland												
572	6/24/2011		Upland												
573	6/27/2011	Kitchen Sink	Valley												
574	6/27/2011		Valley												
575	6/27/2011	House Kitchen Sink	Valley												
576	6/27/2011		Valley												
577	6/27/2011	Kitchen Sink	Upland												
578	6/27/2011	Kitchen Sink	Upland												
579	6/27/2011	Outside Spigot	Valley												
580	6/28/2011	Kitchen Sink	Valley												
581	6/28/2011		Upland												
582	6/28/2011	Outside Spigot	Upland												
583	6/28/2011		Valley												

Table S.8. Groundwater Quality Data for 1701 "Pre-Drill" Water Well Samples from Susquehanna County, Pennsylvania

Water Well ID	Sample Date	Sampling Port	Topo-graphic Location	Gas Extraction Area (Within 1 km of Active Gas Well)	Selenium (mg/L)	Silver (mg/L)	Sodium (mg/L)	Strontium (mg/L)	Sulfate (mg/L)	Sulfide (mg/L)	TDS (mg/L)	Thallium (mg/L)	TSS (mg/L)	Turbidity (NTU)	Vanadium (mg/L)
584	6/28/2011	Kitchen Sink	Upland												
585	6/28/2011		Valley												
586	10/27/2009	Pressure Tank	Upland					0.099		< 1	170		< 2		
587	6/28/2011	Kitchen Sink	Valley												
588	6/28/2011	Basement Pressure Tank	Upland												
589	6/29/2011	Well Spigot	Upland												
590	6/29/2011		Valley												
591	6/29/2011		Upland												
592	6/29/2011		Valley												
593	6/29/2011		Valley												
594	6/29/2011		Upland												
595	6/29/2011	House Spigot	Valley												
596	6/29/2011	Kitchen Sink (Dennis)	Upland												
597	6/30/2011	Kitchen Sink	Valley												
598	10/27/2009	Pressure Tank	Valley					0.298		< 1	88		< 2		
599	6/30/2011	Pressure Tank	Valley												
600	6/30/2011		Valley												
601	6/30/2011	Kitchen Sink	Upland												
602	6/30/2011	Pressure Tank	Valley												
603	6/30/2011		Valley												
604	6/30/2011		Valley												
605	6/30/2011		Valley												
606	6/30/2011	Sink In Bays Of Garage	Upland												
607	10/27/2009	Pressure Tank	Upland					0.386		3	120		4		
608	6/30/2011	Basement Pressure Tank	Valley												
609	6/30/2011	Outside Spigot	Upland												
610	6/30/2011		Upland												
611	6/30/2011	Kitchen Sink	Upland												
612	6/30/2011	Sink In Barn	Valley												
613	7/1/2011	Kitchen Sink	Upland												

Table S.8. Groundwater Quality Data for 1701 "Pre-Drill" Water Well Samples from Susquehanna County, Pennsylvania

Water Well ID	Sample Date	Sampling Port	Topo-graphic Location	Gas Extraction Area (Within 1 km of Active Gas Well)	Selenium (mg/L)	Silver (mg/L)	Sodium (mg/L)	Strontium (mg/L)	Sulfate (mg/L)	Sulfide (mg/L)	TDS (mg/L)	Thallium (mg/L)	TSS (mg/L)	Turbidity (NTU)	Vanadium (mg/L)
614	7/1/2011	Kitchen Sink	Upland												
615	7/1/2011	Kitchen Sink	Upland												
616	7/1/2011	Kitchen Sink	Upland												
617	7/1/2011	Kitchen Sink	Valley												
618	7/1/2011	Outside Spigot	Upland												
619	7/1/2011		Valley												
620	7/1/2011	Outside Spigot	Valley												
621	7/1/2011	Wellhead Spigot	Valley												
622	7/5/2011	Bathroom Sink	Valley												
623	7/5/2011	Kitchen Sink	Upland												
624	7/5/2011	Basement Pressure Tank	Upland												
625	7/5/2011		Valley												
626	7/5/2011	Kitchen Sink	Valley												
627	10/27/2009	Pressure Tank	Valley					0.501		16	205		< 2		
628	7/5/2011	Kitchen Sink	Upland												
629	7/5/2011	Outside Spigot	Valley												
630	7/5/2011	Kitchen Sink	Upland												
631	7/5/2011	Kitchen Sink	Valley												
632	7/5/2011	Outside Spigot	Valley												
633	7/5/2011	Kitchen Sink	Valley												
634	7/5/2011	Kitchen Sink	Valley												
635	7/5/2011	Kitchen Sink Main Camp	Valley												
636	7/5/2011	Pressure Tank	Valley												
637	7/5/2011	Kitchen Sink	Upland												
638	7/5/2011	Kitchen Faucet	Valley												

Table S.8. Groundwater Quality Data for 1701 "Pre-Drill" Water Well Samples from Susquehanna County, Pennsylvania

Water Well ID	Sample Date	Sampling Port	Topo-graphic Location	Gas Extraction Area (Within 1 km of Active Gas Well)	Selenium (mg/L)	Silver (mg/L)	Sodium (mg/L)	Strontium (mg/L)	Sulfate (mg/L)	Sulfide (mg/L)	TDS (mg/L)	Thallium (mg/L)	TSS (mg/L)	Turbidity (NTU)	Vanadium (mg/L)
639	7/5/2011	Kitchen Sink	Valley												
640	7/5/2011	Kitchen Sink	Upland												
641	7/5/2011	Kitchen Sink	Upland												
642	7/6/2011	Kitchen Sink	Upland												
643	7/6/2011	Kitchen Sink	Valley												
644	7/6/2011	Kitchen Sink	Upland												
645	7/6/2011		Upland												
646	7/6/2011	Farm Sink	Valley												
647	7/6/2011		Upland												
648	7/6/2011	Kitchen Sink	Valley												
649	7/6/2011		Valley												
650	7/6/2011	Kitchen Sink	Valley												
651	7/6/2011	Kitchen Sink	Valley												
652	7/6/2011	Basement Pressure Tank	Valley												
653	7/6/2011	House Well	Upland												
654	7/6/2011	Outside Spigot	Valley												
655	7/7/2011	Pressure Tank In Basement	Upland												
656	7/7/2011	Kitchen Sink	Upland												
657	7/7/2011	Outside Faucet	Upland												
658	7/7/2011	Kitchen Sink	Valley												
659	7/7/2011	Kitchen Sink	Upland												
660	7/7/2011	Kitchen Sink	Upland												
661	7/7/2011	Outside Faucet	Valley												
662	7/7/2011	Garage Spigot	Valley												
663	7/7/2011	Kitchen Sink	Valley												
664	7/7/2011	Pressure Tank	Upland												
665	7/7/2011	Kitchen Sink	Upland												

Table S.8. Groundwater Quality Data for 1701 "Pre-Drill" Water Well Samples from Susquehanna County, Pennsylvania

Water Well ID	Sample Date	Sampling Port	Topo-graphic Location	Gas Extraction Area (Within 1 km of Active Gas Well)	Selenium (mg/L)	Silver (mg/L)	Sodium (mg/L)	Strontium (mg/L)	Sulfate (mg/L)	Sulfide (mg/L)	TDS (mg/L)	Thallium (mg/L)	TSS (mg/L)	Turbidity (NTU)	Vanadium (mg/L)
666	7/7/2011	Kitchen Sink	Valley												
667	7/7/2011	Kitchen Sink	Upland												
668	7/7/2011	Kitchen Sink	Upland												
669	7/7/2011	Bunk House Sink	Upland												
670	7/7/2011	Spigot At Well Head	Valley												
671	7/8/2011	Kitchen Sink	Valley												
672	7/8/2011	Kitchen Faucet	Upland												
673	7/8/2011	Pressure Tank	Valley												
674	7/8/2011	Kitchen Faucet	Upland												
675	7/8/2011	Kitchen Faucet	Upland												
676	7/8/2011	Kitchen Sink	Upland												
677	7/8/2011	Kitchen Sink	Upland												
678	7/8/2011	Kitchen Sink	Valley												
679	7/8/2011	Spigot In Cooling Barn	Upland												
680	7/8/2011	Kitchen Sink	Upland												
681	7/8/2011	Shop Sink	Upland												
682	7/8/2011	Kitchen Sink	Upland												
683	7/9/2011	Kitchen Sink	Upland												
684	7/11/2011	Spigot On Front Of House	Valley												
685	7/11/2011	Spigot Near Water Fountain	Valley												
686	7/11/2011	Outside Spigot	Upland												
687	7/11/2011	Kitchen Sink	Valley												
688	7/11/2011	Kitchen Sink	Valley												
689	7/11/2011	Kitchen Sink	Valley												

Table S.8. Groundwater Quality Data for 1701 "Pre-Drill" Water Well Samples from Susquehanna County, Pennsylvania

Water Well ID	Sample Date	Sampling Port	Topo-graphic Location	Gas Extraction Area (Within 1 km of Active Gas Well)	Selenium (mg/L)	Silver (mg/L)	Sodium (mg/L)	Strontium (mg/L)	Sulfate (mg/L)	Sulfide (mg/L)	TDS (mg/L)	Thallium (mg/L)	TSS (mg/L)	Turbidity (NTU)	Vanadium (mg/L)
690	11/5/2009	Pressure Tank	Upland	Yes				0.369		< 1	148		< 2		
691	7/11/2011	Kitchen Sink	Upland												
692	7/11/2011	Kitchen Sink	Upland												
693	7/11/2011	Kitchen Sink	Upland												
694	7/11/2011	Hand Dug Well	Upland												
695	7/11/2011	Kitchen Sink	Valley												
696	7/12/2011	Bathroom Sink Downstairs	Valley												
697	7/12/2011	Kitchen Sink	Upland												
698	7/12/2011	Kitchen Faucet	Valley												
699	7/12/2011	Kitchen Sink	Valley												
700	7/12/2011	Kitchen Sink	Valley												
701	7/12/2011	Pressure Tank	Upland												
702	7/12/2011	Kitchen Sink	Upland												
703	7/12/2011	Spigot Before Filter	Valley												
704	7/12/2011	Kitchen Sink	Valley												
705	7/12/2011		Valley												
706	7/12/2011	Pressure Tank	Upland												
707	7/12/2011	Inside Faucet	Upland												
708	7/12/2011	Kitchen Sink	Upland												
709	7/12/2011	Spigot	Upland												
710	7/12/2011	Spigot	Upland												
711	7/12/2011	Kitchen Sink	Valley												
712	7/12/2011	Kitchen Faucet	Upland												
713	7/12/2011	Kitchen Faucet	Valley												
714	7/13/2011	Kitchen Sink	Valley												
715	7/13/2011	Kitchen Sink	Valley												

Table S.8. Groundwater Quality Data for 1701 "Pre-Drill" Water Well Samples from Susquehanna County, Pennsylvania

Water Well ID	Sample Date	Sampling Port	Topo-graphic Location	Gas Extraction Area (Within 1 km of Active Gas Well)	Selenium (mg/L)	Silver (mg/L)	Sodium (mg/L)	Strontium (mg/L)	Sulfate (mg/L)	Sulfide (mg/L)	TDS (mg/L)	Thallium (mg/L)	TSS (mg/L)	Turbidity (NTU)	Vanadium (mg/L)
716	7/13/2011	Kitchen Sink	Upland												
717	7/13/2011	Spigot	Valley												
718	7/13/2011	Spigot On Well	Valley												
719	7/13/2011	Barn Spigot	Valley												
720	7/13/2011	Kitchen Sink	Valley												
721	7/13/2011	Garage Spigot	Upland												
722	7/13/2011	Kitchen Sink	Valley												
723	7/13/2011	Kitchen Sink	Valley												
724	7/8/2011		Upland												
725	7/8/2011	Kitchen Sink	Upland												
726	4/13/2011	Milkhouse Faucet Barn Well	Valley		< .002	< .001	75	0.42	< 5	5	193		< 2	< 1	
727	4/13/2011	Tenant'S Pressure Tank	Upland		< .002	< .001	2.6	0.032	16	< 1	127		< 2	< 1	
728	4/13/2011	Outside Spigot On Garage	Upland		< .002	< .001	23	0.88	14	< 1	120		27	21	
729	4/26/2011	Pressure Tank	Valley		< .002	< .001	16	0.75	24	< 1	260		13	3	
730	6/17/2011	Spigot On Front Of House	Upland												
731	6/17/2011	Kitchen Sink	Valley												
732	6/17/2011	Spigot Inside Of Shed On Right	Upland												
733	6/17/2011	Kitchen Sink	Valley												
734	6/17/2011	Pressure Tank	Valley												
735	6/20/2011	Kitchen Sink	Upland												
736	6/20/2011	Pressure Tank	Valley												
737	6/20/2011	Kitchen Sink	Upland												
738	6/20/2011	Kitchen Sink	Valley												
739	6/20/2011	Kitchen Sink	Valley												

Table S.8. Groundwater Quality Data for 1701 "Pre-Drill" Water Well Samples from Susquehanna County, Pennsylvania

Water Well ID	Sample Date	Sampling Port	Topo-graphic Location	Gas Extraction Area (Within 1 km of Active Gas Well)	Selenium (mg/L)	Silver (mg/L)	Sodium (mg/L)	Strontium (mg/L)	Sulfate (mg/L)	Sulfide (mg/L)	TDS (mg/L)	Thallium (mg/L)	TSS (mg/L)	Turbidity (NTU)	Vanadium (mg/L)
740	6/20/2011	Kitchen Sink	Valley												
741	6/21/2011	Kitchen Sink	Upland												
742	6/21/2011	Kitchen Sink	Valley												
743	6/21/2011	Kitchen Sink	Valley												
744	6/21/2011	Kitchen Sink	Valley												
745	6/21/2011		Valley												
746	6/21/2011	Outside Spigot	Valley												
747	6/21/2011	Kitchen Sink	Valley												
748	6/21/2011	Wellhead Spigot	Valley												
749	6/21/2011	Kitchen Sink	Upland												
750	6/21/2011		Upland												
751	6/29/2011	Kitchen Sink	Upland												
752	6/29/2011	Barn Spigot	Valley												
753	7/8/2011	Well 2 On South Side Of House	Upland												
754	7/8/2011	Kitchen Sink	Upland												
755	7/8/2011	Kichen Sink	Valley												
756	7/9/2011	Kitchen Sink	Valley												
757	7/9/2011	Spring	Upland												
758	7/11/2011	Kitchen Sink	Valley												
759	7/11/2011	Kitchen Sink	Valley												
760	7/11/2011	Kitchen Sink	Valley												
761	11/5/2009	Outside Faucet	Valley	Yes				0.608		3	142		3.2		
762	7/12/2011	Kitchen Sink	Valley												
763	7/12/2011	Kitchen Sink	Valley												
764	7/14/2011	Kitchen Sink	Upland												
765	7/14/2011	Kitchen Sink	Upland												
766	7/14/2011	Kitchen Sink	Valley												

Table S.8. Groundwater Quality Data for 1701 "Pre-Drill" Water Well Samples from Susquehanna County, Pennsylvania

Water Well ID	Sample Date	Sampling Port	Topo-graphic Location	Gas Extraction Area (Within 1 km of Active Gas Well)	Selenium (mg/L)	Silver (mg/L)	Sodium (mg/L)	Strontium (mg/L)	Sulfate (mg/L)	Sulfide (mg/L)	TDS (mg/L)	Thallium (mg/L)	TSS (mg/L)	Turbidity (NTU)	Vanadium (mg/L)
767	7/14/2011	Spigot On Side Of House	Valley												
768	7/14/2011	Kitchen Sink	Valley												
769	7/14/2011	Kitchen Sink	Valley												
770	7/14/2011	Kitchen Sink	Valley												
771	7/14/2011	Kitchen Sink	Upland												
772	7/14/2011	Kitchen Sink	Valley												
773	7/14/2011	Spigot	Valley												
774	7/15/2011	Kitchen Sink	Valley												
775	7/15/2011	Kitchen Sink	Valley												
776	7/15/2011	Kitchen Sink	Upland												
777	7/15/2011	Kitchen Sink	Upland												
778	7/15/2011	Outside Faucet	Valley												
779	7/15/2011	Kitchen Sink	Valley												
780	7/15/2011	Spigot East Of House	Valley												
781	7/15/2011	Outside Faucet	Upland												
782	7/15/2011	Outside Faucet	Upland												
783	7/15/2011	Kitchen Sink	Upland												
784	7/15/2011	Kitchen Sink	Valley												
785	7/15/2011	Kitchen Sink	Valley												
786	7/15/2011	Kitchen Sink	Valley												
787	7/15/2011	Kitchen Sink	Valley												
788	7/15/2011	Kitchen Sink	Valley												
789	7/15/2011	Kitchen Sink	Valley												
790	7/15/2011	Kitchen Sink	Valley												
791	7/15/2011	Kitchen Sink	Upland												

Table S.8. Groundwater Quality Data for 1701 "Pre-Drill" Water Well Samples from Susquehanna County, Pennsylvania

Water Well ID	Sample Date	Sampling Port	Topo-graphic Location	Gas Extraction Area (Within 1 km of Active Gas Well)	Selenium (mg/L)	Silver (mg/L)	Sodium (mg/L)	Strontium (mg/L)	Sulfate (mg/L)	Sulfide (mg/L)	TDS (mg/L)	Thallium (mg/L)	TSS (mg/L)	Turbidity (NTU)	Vanadium (mg/L)
792	7/15/2011	Spigot On Rear Of House	Valley												
793	7/15/2011	Kitchen Sink	Valley												
794	7/15/2011	Kitchen Sink	Valley												
795	7/16/2011	Kitchen Faucet	Upland												
796	7/16/2011	Kitchen Faucet	Valley												
797	7/16/2011	Kitchen Faucet	Upland												
798	7/16/2011	Kitchen Faucet	Upland												
799	7/16/2011	Kitchen Sink	Upland												
800	7/17/2011	Outside Spigot In Back Of House	Valley												
801	7/17/2011	Outside Spigot On Front Of House	Valley												
802	7/17/2011	Well	Upland												
803	7/17/2011	Blue Well 40Ft From Road	Upland												
804	7/17/2011	Kitchen Faucet	Upland												
805	7/17/2011	Spigot From Garage	Valley												
806	7/18/2011	Outside Spigot	Upland												
807	7/18/2011	Kitchen Sink	Upland												
808	7/18/2011	Outside Spigot	Upland												
809	7/18/2011	Kitchen Sink	Valley												
810	7/18/2011	Kitchen Sink	Upland												
811	7/18/2011	Kitchen Sink	Valley												
812	7/18/2011	Kitchen Sink	Valley												
813	7/19/2011		Upland												
814	7/19/2011	Garage Sink	Valley												
815	7/19/2011	Kitchen Sink	Valley												

Table S.8. Groundwater Quality Data for 1701 "Pre-Drill" Water Well Samples from Susquehanna County, Pennsylvania

Water Well ID	Sample Date	Sampling Port	Topo-graphic Location	Gas Extraction Area (Within 1 km of Active Gas Well)	Selenium (mg/L)	Silver (mg/L)	Sodium (mg/L)	Strontium (mg/L)	Sulfate (mg/L)	Sulfide (mg/L)	TDS (mg/L)	Thallium (mg/L)	TSS (mg/L)	Turbidity (NTU)	Vanadium (mg/L)
816	7/19/2011	Barn 2 Pressure Tank	Valley												
817	7/19/2011	Spigot On Side Of Barn	Upland												
818	7/19/2011	Well Head	Valley												
819	11/23/2009		Upland	Yes				0.225		< 1	108		< 2	< 1	
820	7/19/2011	Kitchen Sink	Valley												
821	7/19/2011	Kitchen Sink	Valley												
822	7/19/2011	Kitchen Sink	Valley												
823	7/19/2011	Kitchen Sink	Valley												
824	7/20/2011	Outside Faucet	Upland												
825	7/20/2011	Inside Spigot	Upland												
826	7/20/2011	Kitchen Sink	Upland												
827	7/20/2011	Kitchen Sink	Valley												
828	7/20/2011	Kitchen Sink	Upland												
829	7/20/2011	Kitchen Sink	Valley												
830	7/20/2011	Outside Spigot	Upland												
831	7/20/2011	Kitchen Sink	Valley												
832	7/20/2011	Kitchen Sink	Valley												
833	7/20/2011	Outside Faucet	Valley												
834	7/20/2011	Kitchen Sink	Upland												
835	7/20/2011	Kitchen Sink	Upland												
836	7/21/2011	Outside Faucet	Upland												
837	7/21/2011	Spigot In Front Of House	Upland												
838	7/21/2011	Kitchen Sink	Valley												
839	7/21/2011	Kitchen Sink	Upland												
840	7/21/2011	Kitchen Sink	Upland												

Table S.8. Groundwater Quality Data for 1701 "Pre-Drill" Water Well Samples from Susquehanna County, Pennsylvania

Water Well ID	Sample Date	Sampling Port	Topo-graphic Location	Gas Extraction Area (Within 1 km of Active Gas Well)	Selenium (mg/L)	Silver (mg/L)	Sodium (mg/L)	Strontium (mg/L)	Sulfate (mg/L)	Sulfide (mg/L)	TDS (mg/L)	Thallium (mg/L)	TSS (mg/L)	Turbidity (NTU)	Vanadium (mg/L)
841	7/21/2011	Kitchen Sink	Valley												
842	7/21/2011	Kitchen Sink	Valley												
843	7/21/2011	Kitchen Sink	Upland												
844	7/21/2011	Pressure Tank	Upland												
845	7/21/2011	Basement Sink	Upland												
846	7/21/2011	Kitchen Sink	Upland												
847	7/21/2011	Spigot In Rear Of House	Upland												
848	7/21/2011	Kitchen Sink	Valley												
849	7/21/2011	Kitchen Sink	Valley												
850	7/22/2011	Kitchen Sink	Valley												
851	7/22/2011	Outside Spigot	Valley												
852	7/22/2011	Kitchen Sink	Valley												
853	7/22/2011	Kitchen Sink	Valley												
854	7/22/2011	Kitchen Sink	Valley												
855	7/22/2011	Pipe Overflow	Valley												
856	7/22/2011	Kitchen Sink	Valley												
857	7/22/2011	Pressure Tank In Garage	Valley												
858	11/5/2009	Basement At Pressure Tank	Valley	Yes				0.515		< 1	156		< 2		
859	7/22/2011	Outside Spigot	Valley												
860	7/22/2011	Kitchen Faucet	Valley												
861	7/22/2011	Spigot Under Porch	Valley												
862	7/22/2011	Kitchen Sink	Valley												
863	7/22/2011	Pressure Tank In Garage	Valley												

Table S.8. Groundwater Quality Data for 1701 "Pre-Drill" Water Well Samples from Susquehanna County, Pennsylvania

Water Well ID	Sample Date	Sampling Port	Topo-graphic Location	Gas Extraction Area (Within 1 km of Active Gas Well)	Selenium (mg/L)	Silver (mg/L)	Sodium (mg/L)	Strontium (mg/L)	Sulfate (mg/L)	Sulfide (mg/L)	TDS (mg/L)	Thallium (mg/L)	TSS (mg/L)	Turbidity (NTU)	Vanadium (mg/L)
864	7/22/2011	Kitchen Sink	Upland												
865	7/22/2011	Kitchen Sink	Upland												
866	7/22/2011	Kitchen Sink	Upland												
867	11/5/2009	Kitchen Sink	Upland	Yes				< .025		< 1	178		< 2		
868	7/22/2011	Kitchen Sink	Valley												
869	7/22/2011	Pressure Tank	Valley												
870	7/25/2011	Kitchen Sink	Valley												
871	7/25/2011	Kitchen Sink	Valley												
872	7/25/2011	Kitchen Sink	Upland												
873	7/25/2011	Kitchen Sink	Upland												
874	7/25/2011	Kitchen Sink	Upland												
875	7/25/2011	Kitchen Sink	Upland												
876	7/25/2011	Warehouse At Quarry	Upland												
877	7/25/2011	Kitchen Sink	Valley												
878	7/25/2011	Kitchen Sink	Upland												
879	7/25/2011	Kitchen Sink	Valley												
880	1/3/2010	Pressure Tank	Upland	Yes				0.594		< 1	132		< 2	< 1	
881	7/25/2011	Kitchen Sink	Valley												
882	7/25/2011	Pressure Tank	Valley												
883	7/26/2011	Kitchen Sink	Valley												
884	7/26/2011	Kitchen Sink	Valley												
885	7/26/2011	Kitchen Sink	Valley												
886	7/26/2011	Kitchen Sink	Valley												
887	7/26/2011	Kitchen Sink	Valley												
888	12/22/2009	Pressure Tank	Upland	Yes				0.046		< 1	64		< 2	< 1	

Table S.8. Groundwater Quality Data for 1701 "Pre-Drill" Water Well Samples from Susquehanna County, Pennsylvania

Water Well ID	Sample Date	Sampling Port	Topo-graphic Location	Gas Extraction Area (Within 1 km of Active Gas Well)	Selenium (mg/L)	Silver (mg/L)	Sodium (mg/L)	Strontium (mg/L)	Sulfate (mg/L)	Sulfide (mg/L)	TDS (mg/L)	Thallium (mg/L)	TSS (mg/L)	Turbidity (NTU)	Vanadium (mg/L)
889	7/26/2011	Kitchen Sink	Upland												
890	7/26/2011	Pipe From Well	Valley												
891	7/26/2011	Outside Spigot	Upland												
892	7/26/2011	Outside Spigot	Valley												
893	7/26/2011	Outside Spigot	Upland												
894	7/26/2011	Kitchen Sink	Valley												
895	12/15/2009	Pressure Tank	Upland					0.291		< 1	184		< 2	< 1	
896	7/26/2011	Outside Spigot	Upland												
897	7/26/2011	Kitchen Sink	Valley												
898	7/26/2011	Outside Spigot	Upland												
899	7/26/2011	Kitchen Sink	Upland												
900	7/26/2011	Kitchen Sink	Upland												
901	7/27/2011	Kitchen Sink	Upland												
902	7/27/2011	Outside Faucet	Valley												
903	7/27/2011	Basement Spigot	Valley												
904	12/17/2009	Pressure Tank	Valley	Yes				1.6		6	120		< 2	< 1	
905	7/27/2011	Kitchen Sink	Valley												
906	7/27/2011	Kitchen Sink	Valley												
907	7/27/2011	Kitchen Sink	Upland												
908	7/27/2011	Pipe Overflow	Valley												
909	7/27/2011	Pressure Tank	Upland												
910	7/27/2011	Kitchen Sink	Upland												
911	7/27/2011	Kitchen Sink	Upland												
912	7/27/2011	Outside Spigot	Valley												
913	7/27/2011	Kitchen Sink	Valley												

Table S.8. Groundwater Quality Data for 1701 "Pre-Drill" Water Well Samples from Susquehanna County, Pennsylvania

Water Well ID	Sample Date	Sampling Port	Topo-graphic Location	Gas Extraction Area (Within 1 km of Active Gas Well)	Selenium (mg/L)	Silver (mg/L)	Sodium (mg/L)	Strontium (mg/L)	Sulfate (mg/L)	Sulfide (mg/L)	TDS (mg/L)	Thallium (mg/L)	TSS (mg/L)	Turbidity (NTU)	Vanadium (mg/L)
914	7/27/2011	Kitchen Sink	Upland												
915	7/27/2011	Kitchen Sink	Upland												
916	7/28/2011	Kitchen Sink	Upland												
917	7/28/2011	Kitchen Sink	Valley												
918	7/28/2011	Kitchen Sink	Upland												
919	7/28/2011	Kitchen Sink	Upland												
920	7/28/2011	Kitchen Sink	Valley												
921	7/28/2011	Outside Spigot	Valley	Yes											
922	7/29/2011	Kitchen Sink	Valley												
923	12/14/2009	Basement At Pressure Tank	Valley	Yes				0.319		< 1	164		< 2	< 1	
924	12/17/2009	Kitchen Sink	Valley					0.514		< 1	152		< 2	< 1	
925	12/13/2009	Pressure Tank	Valley					0.034		< 1	56		< 2	< 1	
926	7/29/2011	Kitchen Sink	Valley												
927	7/29/2011	Bathroom Sink	Valley												
928	7/29/2011	Kitchen Sink	Upland												
929	7/29/2011	Spigot On Side Of House	Upland												
930	7/29/2011	Bathroom Sink	Upland												
931	12/21/2009	Pressure Tank	Valley	Yes				0.468		< 1	140		< 2	1	
932	7/29/2011	Spigot On Side Of House	Upland												
933	7/30/2011	Kitchen Sink	Valley												
934	7/30/2011	Outside Spigot	Valley												
935	7/30/2011	Kitchen Sink	Upland												
936	7/30/2011	Basement Sink	Upland												
937	7/30/2011	Outside Spigot	Valley												

Table S.8. Groundwater Quality Data for 1701 "Pre-Drill" Water Well Samples from Susquehanna County, Pennsylvania

Water Well ID	Sample Date	Sampling Port	Topo-graphic Location	Gas Extraction Area (Within 1 km of Active Gas Well)	Selenium (mg/L)	Silver (mg/L)	Sodium (mg/L)	Strontium (mg/L)	Sulfate (mg/L)	Sulfide (mg/L)	TDS (mg/L)	Thallium (mg/L)	TSS (mg/L)	Turbidity (NTU)	Vanadium (mg/L)
938	7/30/2011	Presssure Tank	Valley												
939	7/30/2011	Kitchen Sink	Upland												
940	7/30/2011	Kitchen Sink	Upland												
941	8/1/2011	Outside Spigot	Valley												
942	8/1/2011	Kitchen Sink	Upland												
943	8/1/2011	Kitchen Sink	Valley												
944	8/1/2011	Kitchen Sink	Valley												
945	8/1/2011	Pressure Tank	Upland	Yes											
946	8/1/2011	Outside Spigot	Valley												
947	12/21/2009	Presssure Tank-Tenants House	Upland					0.569		< 1	124		< 2	< 1	
948	8/1/2011	Kitchen Sink	Upland												
949	8/1/2011	Spigot On Garage	Valley												
950	8/1/2011	Spigot On South Side Of House	Valley												
951	8/1/2011	Shop Sink	Upland												
952	8/1/2011	Pressure Tank	Valley												
953	8/2/2011	Kitchen Sink	Upland												
954	8/2/2011	Kitchen Sink	Valley												
955	8/2/2011	Kitchen Sink	Valley												
956	8/2/2011	Kitchen Sink	Valley												
957	8/2/2011	Kitchen Sink	Upland												
958	8/2/2011	Pressure Tank	Valley												
959	8/2/2011	Kitchen Sink	Upland												
960	8/2/2011	Kitchen Sink	Upland												

Table S.8. Groundwater Quality Data for 1701 "Pre-Drill" Water Well Samples from Susquehanna County, Pennsylvania

Water Well ID	Sample Date	Sampling Port	Topo-graphic Location	Gas Extraction Area (Within 1 km of Active Gas Well)	Selenium (mg/L)	Silver (mg/L)	Sodium (mg/L)	Strontium (mg/L)	Sulfate (mg/L)	Sulfide (mg/L)	TDS (mg/L)	Thallium (mg/L)	TSS (mg/L)	Turbidity (NTU)	Vanadium (mg/L)
961	8/2/2011	Spigot Across The Road From Office Building	Valley												
962	8/2/2011	Kitchen Sink	Upland												
963	8/5/2011	Kitchen Sink	Valley												
964	8/5/2011	Kitchen Sink	Valley												
965	8/5/2011	Spigot On Side Of House	Valley												
966	8/5/2011	Spigot In Shed Behind House	Upland												
967	8/5/2011	Pressure Tank	Valley												
968	8/5/2011	Kitchen Sink	Upland	Yes											
969	8/5/2011	Outside Spigot	Valley												
970	12/15/2009	Pressure Tank	Upland	Yes				0.122		< 1	118		20.5	2	
971	8/5/2011	Kitchen Sink	Upland												
972	8/5/2011	Kitchen Sink	Upland												
973	8/5/2011	Outside Spigot	Valley												
974	8/5/2011	Outside Spigot	Valley												
975	8/5/2011	Spigot On Side Of House	Upland												
976	8/5/2011	Spigot On Side Of House	Upland												
977	8/5/2011	Kitchen Sink	Upland												
978	8/5/2011	Spigot On Side Of House	Upland												
979	8/5/2011	Well	Upland												
980	8/5/2011	Pressure Tank	Upland												
981	8/5/2011	Kitchen Sink	Upland												

Table S.8. Groundwater Quality Data for 1701 "Pre-Drill" Water Well Samples from Susquehanna County, Pennsylvania

Water Well ID	Sample Date	Sampling Port	Topo-graphic Location	Gas Extraction Area (Within 1 km of Active Gas Well)	Selenium (mg/L)	Silver (mg/L)	Sodium (mg/L)	Strontium (mg/L)	Sulfate (mg/L)	Sulfide (mg/L)	TDS (mg/L)	Thallium (mg/L)	TSS (mg/L)	Turbidity (NTU)	Vanadium (mg/L)
982	8/5/2011	Kitchen Faucet	Valley												
983	1/4/2010	Well	Valley	Yes				0.831		6	160		< 2	< 1	
984	8/5/2011	Outside Faucet	Valley												
985	8/5/2011	Kitchen Sink	Upland												
986	8/8/2011	Pressure Tank	Valley												
987	8/8/2011	Kitchen Sink	Valley												
988	8/8/2011	Kitchen Sink	Valley												
989	8/8/2011		Upland												
990	8/8/2011	Kitchen Sink	Valley												
991	8/8/2011	Kitchen Sink	Upland												
992	8/8/2011	Kitchen Sink	Upland												
993	8/8/2011	Outside Spigot	Upland												
994	8/8/2011	Kitchen Sink	Valley												
995	8/8/2011	Kitchen Sink	Valley												
996	8/8/2011	Spigoton Side Of House	Upland												
997	8/8/2011	Sink In Garage	Upland												
998	8/8/2011	Kitchen Sink	Upland												
999	8/8/2011		Valley												
1000	8/8/2011	Outside Spigot	Upland												
1001	8/9/2011	Basement Sink	Upland												
1002	8/9/2011	Kitchen Sink	Valley												
1003	8/9/2011	Pressure Tank	Valley												
1004	8/9/2011	Pressure Tank	Valley												
1005	8/9/2011	Kitchen Sink	Valley												
1006	8/9/2011	Outside Spigot	Valley												
1007	12/14/2009	Basement At Pressure Tank	Valley					0.512		4	152		< 2	1	

Table S.8. Groundwater Quality Data for 1701 "Pre-Drill" Water Well Samples from Susquehanna County, Pennsylvania

Water Well ID	Sample Date	Sampling Port	Topo-graphic Location	Gas Extraction Area (Within 1 km of Active Gas Well)	Selenium (mg/L)	Silver (mg/L)	Sodium (mg/L)	Strontium (mg/L)	Sulfate (mg/L)	Sulfide (mg/L)	TDS (mg/L)	Thallium (mg/L)	TSS (mg/L)	Turbidity (NTU)	Vanadium (mg/L)
1008	8/9/2011	Bathroom Sink	Valley												
1009	8/9/2011	Outside Spigot	Valley												
1010	8/9/2011	Kitchen Sink	Upland												
1011	8/9/2011	Kitchen Sink	Upland												
1012	8/9/2011	Kitchen Sink	Upland												
1013	8/9/2011	Kitchen Sink	Valley												
1014	8/9/2011	Kitchen Sink	Upland												
1015	8/9/2011	Kitchen Sink	Upland												
1016	8/9/2011	Kitchen Sink	Upland												
1017	7/11/2011	Spigot On Side Of House	Upland												
1018	7/12/2011	Bathroom Sink	Valley												
1019	7/12/2011	Sink In Rear Of Far Building	Valley												
1020	7/21/2011	Basement Sink	Upland												
1021	7/22/2011	Kitchen Faucet	Upland												
1022	7/25/2011	Spigot Outside Barn	Valley												
1023	7/25/2011	Kitchen Sink	Upland												
1024	7/26/2011	Outside Spigot	Upland												
1025	7/28/2011		Valley												
1026	8/1/2011	Kitchen Sink	Valley												
1027	8/1/2011	Kitchen Sink	Valley												
1028	8/1/2011	Outside Spigot	Upland												
1029	8/2/2011	Pipe Overflow	Valley												
1030	8/2/2011	Kitchen Faucet	Valley												

Table S.8. Groundwater Quality Data for 1701 "Pre-Drill" Water Well Samples from Susquehanna County, Pennsylvania

Water Well ID	Sample Date	Sampling Port	Topo-graphic Location	Gas Extraction Area (Within 1 km of Active Gas Well)	Selenium (mg/L)	Silver (mg/L)	Sodium (mg/L)	Strontium (mg/L)	Sulfate (mg/L)	Sulfide (mg/L)	TDS (mg/L)	Thallium (mg/L)	TSS (mg/L)	Turbidity (NTU)	Vanadium (mg/L)
1031	8/3/2011	Garage Building Next To House	Valley												
1032	8/3/2011	Kitchen Sink	Upland												
1033	8/3/2011	Outside Faucet	Upland												
1034	8/3/2011	Kitchen Faucet	Valley												
1035	12/15/2009	Well House	Valley	Yes				0.968		4	408		< 2	< 1	
1036	8/3/2011	Pressure Tank	Upland												
1037	8/3/2011	Pressure Tank	Upland												
1038	8/3/2011	Kitchen Sink	Valley												
1039	8/3/2011	Outside Faucet	Valley	Yes											
1040	8/3/2011	Outside Faucet	Valley												
1041	8/3/2011	Outside Faucet	Upland	Yes											
1042	8/3/2011	Kitchen Sink	Upland												
1043	8/3/2011	Pressure Tank	Valley												
1044	1/3/2010	Pressure Tank	Valley					0.051		< 1	76		< 2	2	
1045	8/3/2011	Spigot	Valley												
1046	8/3/2011	Barn Sink	Valley												
1047	8/3/2011	Kitchen Sink	Valley												
1048	8/3/2011	Kitchen Sink	Valley												
1049	8/3/2011	Outside Spigot	Valley												
1050	8/3/2011	Kitchen Sink	Upland												
1051	8/3/2011	Kitchen Sink	Upland												
1052	8/3/2011	Kitchen Sink	Upland												
1053	8/3/2011	Kitchen Sink	Valley												
1054	8/3/2011	Pressure Tank In Basement	Upland												
1055	8/3/2011	Kitchen Sink	Valley												

Table S.8. Groundwater Quality Data for 1701 "Pre-Drill" Water Well Samples from Susquehanna County, Pennsylvania

Water Well ID	Sample Date	Sampling Port	Topo-graphic Location	Gas Extraction Area (Within 1 km of Active Gas Well)	Selenium (mg/L)	Silver (mg/L)	Sodium (mg/L)	Strontium (mg/L)	Sulfate (mg/L)	Sulfide (mg/L)	TDS (mg/L)	Thallium (mg/L)	TSS (mg/L)	Turbidity (NTU)	Vanadium (mg/L)
1056	8/3/2011	Kitchen Sink	Upland												
1057	8/4/2011	Kitchen Sink	Valley												
1058	8/4/2011	Outside Spigot	Upland												
1059	8/4/2011	Outside Spigot	Upland												
1060	12/21/2009	Laundry Sink	Upland					0.385		< 1	224		< 2	< 1	
1061	8/4/2011	Spigot On Side Of House	Upland												
1062	8/4/2011	Spigot Behind House	Valley												
1063	8/4/2011	Kitchen Sink	Valley												
1064	8/4/2011	Kitchen Sink	Valley												
1065	8/4/2011	Kitchen Sink	Valley												
1066	8/4/2011	Outside Spigot	Upland												
1067	8/4/2011	Kitchen Sink	Upland												
1068	8/4/2011	Spigot In Rear Of Building	Valley	Yes											
1069	4/4/2009	Bathroom Sink First Floor	Valley					0.057		< 1	64		< 2		
1070	12/28/2009	Kitchen Sink	Upland					0.706		< 1	132		< 2	< 1	
1071	8/4/2011	Pressure Tank	Valley	Yes											
1072	8/4/2011	Kitchen Sink	Valley	Yes											
1073	8/4/2011	Kitchen Sink	Valley	Yes											
1074	8/4/2011	Spigot In Warehouse	Upland												
1075	8/4/2011	Kitchen Sink	Upland												
1076	8/4/2011	Kitchen Sink	Upland												
1077	8/4/2011	Kitchen Sink	Valley												

Table S.8. Groundwater Quality Data for 1701 "Pre-Drill" Water Well Samples from Susquehanna County, Pennsylvania

Water Well ID	Sample Date	Sampling Port	Topo-graphic Location	Gas Extraction Area (Within 1 km of Active Gas Well)	Selenium (mg/L)	Silver (mg/L)	Sodium (mg/L)	Strontium (mg/L)	Sulfate (mg/L)	Sulfide (mg/L)	TDS (mg/L)	Thallium (mg/L)	TSS (mg/L)	Turbidity (NTU)	Vanadium (mg/L)
1078	1/10/2010	Little Farm House Basement Pressure Tank	Upland					0.066		< 1	252		< 2	< 1	
1079	8/4/2011	Kitchen Sink	Upland												
1080	8/4/2011	Spigot On Side Of House	Valley												
1081	8/4/2011	Kitchen Sink	Upland												
1082	8/4/2011	Kitchen Sink	Valley												
1083	7/11/2011	Outside Hose Bib	Upland	Yes	<0.005	<0.001	32	0.12	13	<3	170		<4	<0.85	
1084	7/11/2011	Basement At Pressure Tank	Upland	Yes	< .005	< .001	91	0.14	14	< 3	280		< 4	< .85	
1085	12/21/2009		Valley	Yes				0.036		< 1	68		< 2	< 1	
1086	7/11/2011	Bathroom Sink First Floor	Valley	Yes	< .005	< .001	4.9	0.1	19	< 3	150		< 4	< .85	
1087	7/12/2011	Outside Hose Bib	Upland	Yes	< .005	< .001	7.1	0.21	6.6	< 3	110		< 4	< .85	
1088	7/12/2011	Kitchen Sink	Valley	Yes	< .005	< .001	11	0.4	14	< 3	160		< 4	< .85	
1089	7/12/2011	Pressure Tank	Valley	Yes	< .005	< .001	12	0.61	11	< 3	140		< 4	5.9	
1090	12/21/2009	Pressure Tank	Valley	Yes				2.97		4	896		< 2	< 1	
1091	7/13/2011	Pressure Tank In Basement	Upland		< .005	< .001	18	1.4	9.4	< 3	230		< 4	< .85	
1092	7/13/2011	Well	Valley		< .005	< .001	11	0.3	6.7	< 3	160		< 4	< .85	
1093	7/13/2011	Outside Hose Bib	Upland	Yes	< .005	< .001	4.7	0.097	10	< 3	130		< 4	0.91	
1094	12/14/2009	Pressure Tank In Basement	Valley	Yes				0.083		< 1	168		3.2	3	
1095	7/13/2011	Well	Valley	Yes	< .005	< .001	8.1	0.25	6.5	< 3	130		< 4	< .85	
1096	7/13/2011	Pressure Tank	Valley	Yes	< .005	< .001	2.6	0.04	10	< 3	100		< 4	< .85	
1097	1/3/2010	Spigot Under House	Upland	Yes				0.335		< 1	112		< 2	< 1	
1098	12/17/2009	Pressure Tank	Upland					1		< 1	160		< 2	< 1	

Table S.8. Groundwater Quality Data for 1701 "Pre-Drill" Water Well Samples from Susquehanna County, Pennsylvania

Water Well ID	Sample Date	Sampling Port	Topo-graphic Location	Gas Extraction Area (Within 1 km of Active Gas Well)	Selenium (mg/L)	Silver (mg/L)	Sodium (mg/L)	Strontium (mg/L)	Sulfate (mg/L)	Sulfide (mg/L)	TDS (mg/L)	Thallium (mg/L)	TSS (mg/L)	Turbidity (NTU)	Vanadium (mg/L)
1099	1/5/2010	Kitchen Sink	Valley	Yes				5.44		< 1	676		< 2	1	
1100	7/18/2011	Outside Faucet	Valley	Yes	< .005	< .001	3.2	0.045	13	< 3	110		< 4	< .85	
1101	12/13/2009		Upland					0.762		< 1	164		< 2	< 1	
1102	7/19/2011	Inside Faucet	Valley	Yes	< .005	< .001	12	0.35	13	< 3	250		< 4	< .85	
1103	7/20/2011	Kitchen Sink Cold Water Tap	Upland		< .005	< .001	3.3	0.2	9	< 3	77		< 4	< .85	
1104	7/20/2011	Pressure Tank	Upland		< .005	< .001	7.4	0.92	12	< 3	160		< 4	< .85	
1105	12/28/2009	Pressure Tank	Upland					2.41		< 1	236		19.6	< 1	
1106	7/20/2011	Frost Free Hydrant 2 Feet From Well Head	Upland		< .005	< .001	13	0.46	12	< 3	120		< 4	< .85	
1107	7/20/2011	Inside Faucet	Upland		< .005	< .001	10	0.36	12	< 3	390		< 4	< .85	
1108	12/13/2009	Pressure Tank	Upland					1.09		< 1	156		< 2	< 1	
1109	12/28/2009	Pressure Tank	Upland					< .025		12	224		< 2	< 1	
1110	7/26/2011	Pressure Tank In Gun Shop	Valley		< .005	< .001	69	0.51	2.6	< 3	220		< 4	< .85	
1111	12/16/2009	Kitchen Sink	Valley	Yes				0.036		< 1	84		< 2	< 1	
1112	7/26/2011	Inside Faucet	Valley		< .005	< .001	34	0.84	13	< 3	200		< 4	< .85	
1113	7/27/2011	Outside Spigot Near Large Rock Slabs	Valley		< .005	< .001	44	0.79	7.4	< 3	160		< 4	< .85	
1114	12/13/2009	Pressure Tank	Upland	Yes				0.557		< 1	124		< 2	< 1	
1115	7/27/2011	Outside Faucet	Upland		< .005	< .001	6.3	0.17	13	< 3	190		< 4	0.93	
1116	7/27/2011	Kitchen Sink Cold Water Tap	Valley	Yes	< .005	< .001	10	0.19	8.4	< 3	180		< 4	1.8	
1117	1/11/2010	Pressure Tank	Upland					1.45		3	116		< 2	< 1	
1118	7/27/2011	Kitchen Sink Faucet	Upland		< .005	< .001	4.5	0.1	7.8	< 3	140		< 4	< .85	
1119	7/27/2011	Outside Hose Bib	Upland		< .005	< .001	8.4	0.42	8.4	< 3	190		< 4	< .85	
1120	7/28/2011	Kitchen Sink	Valley		< .005	< .001	14	0.13	12	< 3	130		< 4	< .85	

Table S.8. Groundwater Quality Data for 1701 "Pre-Drill" Water Well Samples from Susquehanna County, Pennsylvania

Water Well ID	Sample Date	Sampling Port	Topo-graphic Location	Gas Extraction Area (Within 1 km of Active Gas Well)	Selenium (mg/L)	Silver (mg/L)	Sodium (mg/L)	Strontium (mg/L)	Sulfate (mg/L)	Sulfide (mg/L)	TDS (mg/L)	Thallium (mg/L)	TSS (mg/L)	Turbidity (NTU)	Vanadium (mg/L)
1121	7/28/2011	Inside Faucet	Valley		< .005	< .001	13	0.42	11	< 3	140		< 4	1.8	
1122	7/28/2011	Outside Faucet	Valley		< .005	< .001	11	0.63	8.7	< 3	120		< 4	< .85	
1123	7/29/2011	Pressure Tank	Upland	Yes	< .005	< .001	9.1	0.079	17	< 3	150		< 4	< .85	
1124	7/29/2011	Kitchen Sink	Valley		< .005	< .001	2.3	0.023	6.6	< 3	66		< 4	< .85	
1125	7/29/2011	Pressure Tank	Valley		< .005	< .001	3.8	0.16	15	< 3	120		6.4	18	
1126	8/1/2011	Outside Faucet	Upland		< .005	< .001	10	0.39	9.8	< 3	110		< 4	< .85	
1127	8/1/2011	Outside Faucet	Valley		< .005	< .001	1.5	0.034	5.8	< 3	29		< 4	0.91	
1128	8/1/2011	Pressure Tank At Basement	Upland	Yes	< .005	< .001	9.6	0.32	12	< 3	130		< 4	< .85	
1129	8/2/2011		Upland	Yes	<0.005	<0.001	12	0.072	17	<3	150		<4	8.6	
1130	12/28/2009	Kitchen Sink	Valley					0.063		< 1	88		< 2	< 1	
1131	8/3/2011	Kitchen Sink	Upland	Yes	<.005	< .001	17	0.18	19	<3	200		<4	<0.85	
1132	8/3/2011	Pressure Tank	Valley		< .005	< .001	17	0.39	< 1	< 3	110		< 4	6.6	
1133	10/22/2009		Upland	Yes				0.319		< 1	136				
1134	12/28/2009	Garage Sink	Upland					0.747		< 1	300		< 2	< 1	
1135	8/4/2011	Bathroom Sink Cwt On South End	Upland		< .005	< .001	5.6	0.13	7.6	< 3	120		36	1.4	
1136	8/4/2011	Bailed Well	Upland	Yes	< .005	< .001	2.7	0.043	15	< 3	130		< 4	< .85	
1137	8/4/2011	Kitchen Sink	Upland	Yes	< .005	< .001	11	0.65	17	< 3	170		< 4	< .85	
1138	8/4/2011	Pressure Tank	Valley	Yes	< .005	< .001	19	0.14	24	< 3	270		< 4	< .85	
1139	5/19/2011		Valley		<0.005	<0.001	26	0.34	4	<3	150		<4	<0.85	
1140	7/25/2011	Kitchen Sink	Valley		< .005	< .001	29	0.52	14	< 3	180		< 4	< .85	
1141	7/27/2011	Outside Spigot Under Back Deck	Valley		< .005	< .001	12	0.45	7.6	< 3	140		5.2	16	
1142	8/2/2011	Pressure Tank	Valley		< .005	< .001	6.2	0.12	9.3	< 3	140		< 4	1	

Table S.8. Groundwater Quality Data for 1701 "Pre-Drill" Water Well Samples from Susquehanna County, Pennsylvania

Water Well ID	Sample Date	Sampling Port	Topo-graphic Location	Gas Extraction Area (Within 1 km of Active Gas Well)	Selenium (mg/L)	Silver (mg/L)	Sodium (mg/L)	Strontium (mg/L)	Sulfate (mg/L)	Sulfide (mg/L)	TDS (mg/L)	Thallium (mg/L)	TSS (mg/L)	Turbidity (NTU)	Vanadium (mg/L)
1143	8/2/2011	Outside Faucet	Upland		< .005	< .001	4.1	0.051	8.5	< 3	120		< 4	< .85	
1144	1/3/2010	Kitchen Sink	Upland	Yes				0.233		< 1	140		< 2	< 1	
1145	12/13/2009		Upland	Yes				0.25		< 1	172		< 2	< 1	
1146	1/10/2010	Spring House	Upland					0.088		< 1	304		< 2	< 1	
1147	1/5/2010	Pressure Tank	Upland					< .025		< 1	60		< 2	1	
1148	5/10/2011	Kitchen Sink	Valley		< .005	< .005	63.4	0.473	9	< 1	240		< 2	1	
1149	2/1/2010	Pressure Tank	Upland					0.058		< 1	104		< 2	< 1	
1150	8/20/2009		Valley					0.366		4	706		13.2		
1151	12/6/2009	Washroom Sink	Valley					0.044		< 1	76		< 2	< 1	
1152	1/17/2010	Kitchen Sink	Valley					1.33		< 1	216		< 2	< 1	
1153	1/14/2010	Basement At Pressure Tank	Upland					0.33		< 1	152		2.4	< 1	
1154	1/17/2010	Kitchen Sink	Upland					0.335		< 1	172		< 2	< 1	
1155	1/17/2010	Pressure Tank	Valley					0.035		< 1	60		< 2	< 1	
1156	12/11/2010	Kitchen Sink	Valley		<0.002	<0.001	21	39	8	<1	40		<2	1	
1157	8/4/2011	Spigot Back Yard	Upland												
1158	8/4/2011	Kitchen Sink	Valley												
1159	8/4/2011	Pressure Tank	Valley												
1160	8/10/2011		Upland		< .002	< .001	53	3.2	< 5	< 1	332		< 2	< 1	
1161	8/10/2011	Kitchen Sink	Valley												
1162	8/10/2011	Kitchen Sink	Upland												
1163	8/10/2011	Spigot On Side Of House	Upland												
1164	8/10/2011	Outside Spigot	Valley												
1165	8/10/2011	Kitchen Sink	Valley												

Table S.8. Groundwater Quality Data for 1701 "Pre-Drill" Water Well Samples from Susquehanna County, Pennsylvania

Water Well ID	Sample Date	Sampling Port	Topo-graphic Location	Gas Extraction Area (Within 1 km of Active Gas Well)	Selenium (mg/L)	Silver (mg/L)	Sodium (mg/L)	Strontium (mg/L)	Sulfate (mg/L)	Sulfide (mg/L)	TDS (mg/L)	Thallium (mg/L)	TSS (mg/L)	Turbidity (NTU)	Vanadium (mg/L)
1166	8/10/2011	Spigot On Side Of Shed	Valley												
1167	8/10/2011	Outside Spigot	Upland												
1168	8/10/2011	Kitchen Sink	Upland												
1169	8/10/2011	Outside Spigot	Upland												
1170	8/10/2011	Kitchen Sink	Upland												
1171	8/10/2011	Dug Well	Upland												
1172	8/10/2011	Shop Sink	Upland												
1173	12/10/2009	Kitchen Sink	Valley	Yes				0.804		4	192		< 2	3	
1174	8/11/2011	Kitchen Sink	Upland												
1175	8/11/2011	Kitchen Sink	Upland												
1176	8/11/2011	Outside Spigot	Valley												
1177	8/11/2011	Kitchen Sink	Upland												
1178	8/14/2011	Outside Spigot	Valley												
1179	8/15/2011	Hand Dug Well	Upland												
1180	8/16/2011	Kitchen Sink	Upland												
1181	1/14/2010	Basement Pressure Tank	Valley					< .025		< 1	24		< 2	< 1	
1182	8/16/2011	Dug Well	Upland												
1183	8/16/2011	Kitchen Sink	Valley												
1184	2/1/2010	Elementary Boiler Room	Upland	Yes				0.36		< 1	192		< 2	< 1	
1185	8/17/2011	Kitchen Sink	Valley												
1186	8/19/2011	Kitchen Sink	Upland												
1187	8/19/2011	Kitchen Sink	Valley												
1188	8/29/2011	Outside Faucet	Valley		< .005	< .001	7.9	0.26	9.94	< .1	138		< 1	< 1	
1189	1/14/2010	Basement At Pressure Tank	Valley					0.717		< 1	148		2	1	
1190	9/1/2011	Outside Faucet	Upland												
1191	9/1/2011	Hydrant	Upland												

Table S.8. Groundwater Quality Data for 1701 "Pre-Drill" Water Well Samples from Susquehanna County, Pennsylvania

Water Well ID	Sample Date	Sampling Port	Topo-graphic Location	Gas Extraction Area (Within 1 km of Active Gas Well)	Selenium (mg/L)	Silver (mg/L)	Sodium (mg/L)	Strontium (mg/L)	Sulfate (mg/L)	Sulfide (mg/L)	TDS (mg/L)	Thallium (mg/L)	TSS (mg/L)	Turbidity (NTU)	Vanadium (mg/L)
1192	8/2/2011	Pressure Tank	Upland	Yes	< .005	< .001	90	0.035	11	< 3	240		< 4	3.4	
1193	1/17/2010	Garage Sink	Upland					0.668		< 1	180		< 2	< 1	
1194	8/10/2011	Outside Faucet	Valley	Yes	< .005	< .001	11	0.7	8.3	< 3	170		26	14	
1195	8/10/2011	Inside Faucet	Upland	Yes	< .005	< .001	22	3	3.6	< 3	150		< 4	< .85	
1196	1/13/2010	Kitchen Sink	Upland					0.03		< 1	180		< 2	< 1	
1197	2/3/2010	Sink In Milkhouse Barn	Upland	Yes				0.285		< 1	188		2	< 1	
1198	8/15/2011	Outside Faucet	Upland	Yes	< .005	< .001	8.9	0.41	12	< 3	150		< 4	< .85	
1199	1/12/2010	Pressure Tank	Upland					0.084		< 1	100		< 2	1	
1200	1/18/2010	Kitchen Sink	Upland					0.038		< 1	80		< 2	< 1	
1201	8/22/2011	Barn Spigot	Upland												
1202	8/22/2011	Kitchen Sink	Valley												
1203	8/22/2011	Kitchen Sink	Upland												
1204	8/22/2011	Kitchen Sink	Valley												
1205	8/22/2011	Kitchen Sink	Upland												
1206	8/23/2011	Outside Spigot	Valley												
1207	1/23/2010	Basement At Pressure Tank	Upland					0.515		< 1	140		< 2	< 1	
1208	1/13/2010		Valley					1		< 1	1044		< 2	< 1	
1209	6/23/2011	Kitchen Sink	Valley	Yes	< .005	< .001	17	0.43	13	< 3	150		< 4	< .85	
1210	1/13/2010	Kitchen Sink	Upland					0.562		< 1	132		< 2	< 1	
1211	8/5/2011	Inside Faucet	Upland	Yes	< .005	< .001	9.8	0.45	7.6	< 3	20		< 4	< .85	
1212	8/12/2011	Pressure Tank	Valley		<0.005	<0.001	10	0.45	11	<3	180		<4	<0.85	
1213	12/6/2009	Kitchen Sink	Upland					0.287		< 1	210		< 2	< 1	
1214	8/12/2011	Inside Faucet	Upland		< .005	< .001	4	0.16	12	< 3	150		< 4	0.96	
1215	8/21/2011	Outside Faucet	Upland		< .005	< .001	13	1.2	12	< 3	160		29	82	
1216	8/23/2011	Inside Faucet	Upland		< .005	< .001	19	1	14	< 3	190		< 4	< .85	

Table S.8. Groundwater Quality Data for 1701 "Pre-Drill" Water Well Samples from Susquehanna County, Pennsylvania

Water Well ID	Sample Date	Sampling Port	Topo-graphic Location	Gas Extraction Area (Within 1 km of Active Gas Well)	Selenium (mg/L)	Silver (mg/L)	Sodium (mg/L)	Strontium (mg/L)	Sulfate (mg/L)	Sulfide (mg/L)	TDS (mg/L)	Thallium (mg/L)	TSS (mg/L)	Turbidity (NTU)	Vanadium (mg/L)
1217	8/23/2011	Inside Faucet	Upland	Yes	< .005	< .001	20	1.2	9.9	< 3	140		< 4	< .85	
1218	8/24/2011	Pressure Tank	Upland	Yes	< .005	< .001	19	0.11	15	< 3	98		28	10	
1219	8/24/2011	Inside Faucet	Valley		< .005	< .001	7.7	0.24	6.8	< 3	120		< 4	2.5	
1220	1/18/2010	Basement Pressure Tank	Upland					0.465		< 1	252		< 2	< 1	
1221	8/25/2011	Inside Faucet	Upland		< .005	< .001	6.4	0.4	8.4	< 3	170		< 4	0.99	
1222	8/26/2011	Well	Upland	Yes	< .005	< .001	5.6	0.067	1.7	< 3	120		69	45	
1223	8/29/2011	Kitchen Sink	Upland		< .005	< .001	12	0.61	7.8	< 3	130		< 4	< .85	
1224	1/11/2010	Kitchen Sink	Upland					0.641		< 1	140		< 2	2	
1225	1/18/2010	Pressure Tank In Basement	Valley	Yes				0.684		3	136		< 2	< 1	
1226	1/24/2010	Pressure Tank	Upland	Yes				0.76		3	152		< 2	< 1	
1227	8/30/2011	Pressure Tank	Upland		< .005	< .001	9.6	0.27	9.3	< 3	150		< 4	8	
1228	8/30/2011	Outside Hose Bib	Valley		< .005	< .001	14	0.66	9.1	< 3	170		< 4	< .85	
1229	8/10/2011	Pressure Tank Before Treatment	Valley		< .005	< .001	85	0.56	2.6	< 3	260		< 4	1.1	
1230	1/10/2010	Little House By Pond Spring	Valley					0.046		< 1	156		< 2	< 1	
1231	1/10/2010	Pressure Tank	Valley					0.806		3	164		2	< 1	
1232	1/16/2010	Basement Pressure Tank	Upland					0.957		< 1	136		< 2	< 1	
1233	7/19/2011	Kitchen Sink	Upland												
1234	8/24/2011	Kitchen Sink	Valley												
1235	8/24/2011	Inside Faucet	Valley												
1236	1/16/2010	Basement At Pressure Tank	Upland					0.086		< 1	108		< 2	< 1	
1237	1/16/2010	Basement Pressure Tank	Upland					0.452		< 1	128		< 2	< 1	

Table S.8. Groundwater Quality Data for 1701 "Pre-Drill" Water Well Samples from Susquehanna County, Pennsylvania

Water Well ID	Sample Date	Sampling Port	Topo-graphic Location	Gas Extraction Area (Within 1 km of Active Gas Well)	Selenium (mg/L)	Silver (mg/L)	Sodium (mg/L)	Strontium (mg/L)	Sulfate (mg/L)	Sulfide (mg/L)	TDS (mg/L)	Thallium (mg/L)	TSS (mg/L)	Turbidity (NTU)	Vanadium (mg/L)
1238	8/26/2011	Kitchen Sink	Valley												
1239	8/27/2011	Kitchen Sink	Upland												
1240	8/27/2011	Kitchen Sink	Valley												
1241	1/21/2010	Kitchen Sink	Upland					0.367		< 1	144		< 2	< 1	
1242	8/27/2011	Kitchen Faucet	Valley												
1243	1/23/2010	Pressre Tank In Barn	Upland	Yes				0.455		< 1	216		< 2	< 1	
1244	9/1/2011	Kitchen Sink	Valley												
1245	1/23/2010	Basement At Pressure Tank	Upland	Yes				0.22		< 1	300		< 2	< 1	
1246	9/1/2011	Well In Field	Upland												
1247	8/10/2011	Kitchen Sink	Upland												
1248	8/10/2011	Kitchen Sink	Upland												
1249	8/11/2011	Pressure Tank	Valley												
1250	1/14/2010	Basement At Pressure Tank	Upland					0.213		< 1	208		< 2	< 1	
1251	2/3/2010	Holding Tank	Upland					0.028		< 1	64		< 2	< 1	
1252	8/18/2011	Kitchen Sink	Valley	Yes	< .005	< .001	12	0.42	17	< 3	150		< 4	< .85	
1253	1/31/2010	Basement At Pressure Tank	Upland					0.063		< 1	96		< 2	< 1	
1254	1/24/2010	Kitchen Sink	Upland					0.29		< 1	172		< 2	< 1	
1255	1/21/2010	Pressure Tank	Valley	Yes				0.5		4	124		< 2	< 1	
1256	2/12/2009		Upland	Yes				0.072		<1	148		<2		
1257	4/8/2009		Upland					0.322		<1	88		<2		
1258	1/16/2010	Basement Pressure Tank	Upland	Yes				0.271		< 1	148		< 2	< 1	
1259	1/24/2010	Pressure Tank - Drilled Well	Valley	Yes				0.078		< 1	136		< 2	< 1	
1260	1/31/2010	Crawl Space At Pressure Tank	Valley					0.67		< 1	192		< 2	< 1	
1261	1/31/2010	Pressure Tank	Valley					< .025		< 1	64		< 2	2	

Table S.8. Groundwater Quality Data for 1701 "Pre-Drill" Water Well Samples from Susquehanna County, Pennsylvania

Water Well ID	Sample Date	Sampling Port	Topo-graphic Location	Gas Extraction Area (Within 1 km of Active Gas Well)	Selenium (mg/L)	Silver (mg/L)	Sodium (mg/L)	Strontium (mg/L)	Sulfate (mg/L)	Sulfide (mg/L)	TDS (mg/L)	Thallium (mg/L)	TSS (mg/L)	Turbidity (NTU)	Vanadium (mg/L)
1262	1/24/2010	Outside Spigot	Upland					0.314		< 1	152		< 2	< 1	
1263	1/24/2010	Kitchen Sink	Upland					0.314		< 1	152		< 2	< 1	
1264	1/23/2010	Pressure Tank In Basement	Valley	Yes				< .025		< 1	40		< 2	< 1	
1265	1/18/2010	Basement Pressure Tank	Upland					0.759		< 1	124		< 2	< 1	
1266	1/10/2010	Basement Pressure Tank	Upland					< .025		< 1	236		< 2	1	
1267	1/11/2010	Pressure Tank	Upland					0.18		< 1	92		< 2	< 1	
1268	12/9/2009	Basement At Pressure Tank	Upland	Yes				1.41		< 1	176		< 2	3	
1269	1/18/2010	Pressure Tank	Upland					0.393		< 1	160		< 2	< 1	
1270	1/31/2010		Upland	Yes				0.292		< 1	192		< 2	< 1	
1271	8/7/2009	Kitchen Sink	Valley					0.035		< 1	73		< 2		
1272	1/12/2010	Kitchen Sink At Ashley Sarnosky'S	Upland					0.036		< 1	56		< 2	< 1	
1273	2/21/2010	Kitchen Sink	Upland					0.504		< 1	128		< 2	< 1	
1274	8/6/2009		Upland	Yes				1.01		< 1	240		< 2		
1275	2/15/2010	Pressure Tank	Upland					0.562		< 1	200		< 2	1	
1276	2/13/2010	Basement At Pressure Tank	Valley					0.098		< 1	224		< 2	< 1	
1277	2/27/2010	Basement At Spigot	Valley					0.096		< 1	124		< 2	< 1	
1278	2/18/2010	Pressure Tank	Upland	Yes				0.385		< 1	160		< 2	< 1	
1279	2/16/2010	Kitchen Sink	Upland					1.28		< 1	124		< 2	< 1	
1280	2/18/2010	Kitchen Sink	Upland	Yes				0.279		< 1	180		< 2	< 1	
1281	2/18/2010	Kitchen Sink	Upland					0.456		< 1	168		< 2	< 1	
1282	2/18/2010	Kitchen Sink	Upland					0.221		< 1	164		< 2	< 1	

Table S.8. Groundwater Quality Data for 1701 "Pre-Drill" Water Well Samples from Susquehanna County, Pennsylvania

Water Well ID	Sample Date	Sampling Port	Topo-graphic Location	Gas Extraction Area (Within 1 km of Active Gas Well)	Selenium (mg/L)	Silver (mg/L)	Sodium (mg/L)	Strontium (mg/L)	Sulfate (mg/L)	Sulfide (mg/L)	TDS (mg/L)	Thallium (mg/L)	TSS (mg/L)	Turbidity (NTU)	Vanadium (mg/L)
1283	2/22/2010	Rental House Basement At Pressure Tank	Upland					0.167		< 1	148		< 2	< 1	
1284	2/16/2010	Kitchen Sink	Upland					0.226		< 1	152		< 2	< 1	
1285	2/21/2010	Kitchen Sink	Upland					0.788		< 1	196		< 2	< 1	
1286	2/18/2010	Pressure Tank	Valley					0.154		< 20	162		114	65	
1287	2/6/2010	Basement At Pressure Tank	Valley					0.061		< 1	136		< 2	< 1	
1288	2/13/2010	Pressure Tank	Upland					0.194		< 1	220		< 2	< 1	
1289	2/1/2010	Basement Sink	Valley	Yes				0.992		< 1	144		< 2	< 1	
1290	2/21/2010	Pressure Tank	Upland					0.048		< 1	140		< 2	< 1	
1291	2/21/2010	Barn Well	Upland					0.078		< 1	436		2	1	
1292	2/16/2010	Kitchen Sink	Upland					0.256		< 1	108		< 2	< 1	
1293	2/22/2010	Hand Dug Well	Upland					0.037		< 1	128		< 2	< 1	
1294	2/16/2010	Bathroom Sink	Upland	Yes				0.202		< 1	128		< 2	< 1	
1295	2/16/2010	Pressure Tank	Upland					0.129		< 1	152		< 2	< 1	
1296	2/7/2010	Main House Pressure Tank	Valley					0.122		< 1	156		< 2	1	
1297	2/7/2010	Rental Property Pressure Tank	Upland					0.093		< 1	368		< 2	< 1	
1298	2/13/2010	Kitchen Sink	Upland					1.08		< 1	168		< 2	< 1	
1299	2/13/2010	Well House	Upland					0.091		< 1	296		< 2	< 1	
1300	8/6/2009		Valley					0.031		< 1	96		7.6		
1301	2/7/2010	Barn - Pump	Upland					0.055		< 1	136		< 2	< 1	
1302	2/7/2010	Pressure Tank	Upland					0.106		< 1	120		< 2	< 1	
1303	2/7/2010	Pressure Tank	Upland					0.403		< 1	76		< 2	< 1	
1304	2/6/2010	Pressure Tank	Valley					0.452		< 1	332		4.4	8	
1305	2/6/2010	Basement At Pressure Tank	Valley					0.167		< 1	188		< 2	< 1	

Table S.8. Groundwater Quality Data for 1701 "Pre-Drill" Water Well Samples from Susquehanna County, Pennsylvania

Water Well ID	Sample Date	Sampling Port	Topo-graphic Location	Gas Extraction Area (Within 1 km of Active Gas Well)	Selenium (mg/L)	Silver (mg/L)	Sodium (mg/L)	Strontium (mg/L)	Sulfate (mg/L)	Sulfide (mg/L)	TDS (mg/L)	Thallium (mg/L)	TSS (mg/L)	Turbidity (NTU)	Vanadium (mg/L)
1306	2/6/2010	Pressure Tank	Valley					0.157		< 1	104		< 2	< 1	
1307	2/15/2010	Outside Hydrant	Upland	Yes				0.258		< 1	148		< 2	< 1	
1308	2/27/2010	Hand Dug Well 1 Back Of House	Valley					0.046		< 1	132		< 2	< 1	
1309	2/27/2010	Hand Dug Well 2 Front Of House	Valley					0.053		< 5	140		< 2	< 1	
1310	2/7/2010	Outside Faucet	Valley					2.05		4	184		2.4	7	
1311	2/6/2010	Basement At Pressure Tank	Valley					0.118		< 1	136		< 2	< 1	
1312	2/21/2010	Kitchen Sink	Valley					0.639		< 1	132		< 2	< 1	
1313	8/6/2009		Valley					0.306		< 1	212		< 2		
1314	2/22/2010	Milkhouse Sink	Upland					0.181		< 1	180		< 2	< 1	
1315	2/28/2010	Pressure Tank	Valley	Yes				0.279		< 1	172		< 2	1	
1316	2/13/2010	Basement Men'S Room Sink	Upland					0.385		< 1	792		2	1	
1317	2/28/2010	Outside Spigot	Valley					0.313		< 1	108		< 2	1	
1318	2/28/2010	Kitchen Sink	Upland					0.255		< 1	156		< 2	< 1	
1319	2/27/2010	Basement Pressure Tank	Valley					0.084		< 5	252		3.6	10	
1320	3/16/2010	Kitchen Sink	Valley	Yes				< .025		< 1	68		< 2	< 1	
1321	3/11/2010	Kitchen Sink	Upland	Yes				0.093		< 1	110		< 2	< 1	
1322	3/18/2010	Kitchen Sink	Upland					0.198		< 1	428	< .002	4		< .01
1323	8/11/2011	Kitchen Sink	Upland	Yes											
1324	8/11/2011	Kitchen Sink	Valley												
1325	8/11/2011	Kitchen Sink	Upland												
1326	8/11/2011	Kitchen Sink	Upland												
1327	8/11/2011	Kitchen Sink	Upland												
1328	8/11/2011	Kitchen Sink	Upland												

Table S.8. Groundwater Quality Data for 1701 "Pre-Drill" Water Well Samples from Susquehanna County, Pennsylvania

Water Well ID	Sample Date	Sampling Port	Topo-graphic Location	Gas Extraction Area (Within 1 km of Active Gas Well)	Selenium (mg/L)	Silver (mg/L)	Sodium (mg/L)	Strontium (mg/L)	Sulfate (mg/L)	Sulfide (mg/L)	TDS (mg/L)	Thallium (mg/L)	TSS (mg/L)	Turbidity (NTU)	Vanadium (mg/L)
1329	8/11/2011	Kitchen Sink	Upland												
1330	8/6/2009		Valley					0.163		< 1	292		< 2		
1331	8/11/2011	Kitchen Sink	Upland												
1332	8/11/2011	Kitchen Sink	Valley												
1333	8/11/2011	Kitchen Sink	Upland												
1334	8/11/2011	Kitchen Sink	Valley												
1335	8/12/2011	Kitchen Sink	Valley												
1336	8/12/2011	Kitchen Sink	Valley												
1337	8/12/2011	Kitchen Sink	Valley												
1338	8/12/2011	Kitchen Sink	Valley												
1339	8/12/2011	Kitchen Sink	Valley												
1340	5/13/2010		Upland	Yes						< 1	125		< 2		
1341	8/12/2011	Pressure Tank	Upland												
1342	8/12/2011	Kitchen Sink	Valley												
1343	8/12/2011	Bathroom Sink	Valley												
1344	8/14/2011	Outside Spigot	Valley												
1345	8/14/2011	Kitchen Sink	Upland												
1346	5/13/2010		Upland					1.29		< 1	85	< .002	< 2		< .01
1347	8/14/2011	Kitchen Sink	Valley												
1348	8/14/2011	Kitchen Sink	Upland												
1349	8/14/2011	Kitchen Sink	Upland												
1350	8/14/2011	Pressure Tank	Upland												
1351	8/14/2011	Kitchen Sink	Upland												
1352	8/15/2011	Kitchen Sink	Upland												
1353	8/15/2011	Barn Sink	Upland												
1354	8/15/2011	Kitchen Sink	Valley												
1355	8/15/2011	Kitchen Sink	Valley												
1356	8/15/2011	Sink In Barn	Valley												

Table S.8. Groundwater Quality Data for 1701 "Pre-Drill" Water Well Samples from Susquehanna County, Pennsylvania

Water Well ID	Sample Date	Sampling Port	Topo-graphic Location	Gas Extraction Area (Within 1 km of Active Gas Well)	Selenium (mg/L)	Silver (mg/L)	Sodium (mg/L)	Strontium (mg/L)	Sulfate (mg/L)	Sulfide (mg/L)	TDS (mg/L)	Thallium (mg/L)	TSS (mg/L)	Turbidity (NTU)	Vanadium (mg/L)
1357	8/16/2011	Kitchen Sink	Upland												
1358	8/16/2011	Outside Spigot	Upland												
1359	8/16/2011	Kitchen Sink	Upland												
1360	8/16/2011	Kitchen Sink	Upland												
1361	8/16/2011	Kitchen Sink	Valley												
1362	8/16/2011	Spigot On Side Of House	Upland												
1363	8/17/2011	Kitchen Sink	Upland												
1364	8/17/2011	Outside Spigot	Valley												
1365	8/17/2011	Kitchen Sink	Upland												
1366	8/17/2011	Kitchen Sink	Valley												
1367	8/17/2011	Kitchen Sink	Valley												
1368	8/17/2011	Kitchen Sink	Valley												
1369	8/18/2011	Kitchen Sink	Upland												
1370	2/8/2010	Basement At Pressure Tank	Upland	Yes				1.2		< 1	200		< 2	< 1	
1371	8/18/2011	Kitchen Sink	Valley												
1372	8/18/2011	Kitchen Sink	Valley												
1373	8/18/2011	Kitchen Sink	Valley												
1374	8/18/2011	Barn Sink	Upland												
1375	8/18/2011	Barn Sink	Valley												
1376	3/8/2010	Kitchen Sink	Valley	Yes				0.209		< 1	165		< 2	< 1	
1377	8/18/2011	Kitchen Sink	Upland												
1378	8/19/2011	Kitchen Sink	Upland												
1379	8/19/2011	Kitchen Sink	Upland												
1380	8/19/2011	Kitchen Sink	Upland												
1381	8/17/2009		Valley					0.41		< 1	253		< 2		
1382	8/19/2011		Valley												

Table S.8. Groundwater Quality Data for 1701 "Pre-Drill" Water Well Samples from Susquehanna County, Pennsylvania

Water Well ID	Sample Date	Sampling Port	Topo-graphic Location	Gas Extraction Area (Within 1 km of Active Gas Well)	Selenium (mg/L)	Silver (mg/L)	Sodium (mg/L)	Strontium (mg/L)	Sulfate (mg/L)	Sulfide (mg/L)	TDS (mg/L)	Thallium (mg/L)	TSS (mg/L)	Turbidity (NTU)	Vanadium (mg/L)
1383	8/19/2011	Kitchen Sink	Valley												
1384	8/22/2011	Kitchen Sink	Upland												
1385	8/22/2011	Kitchen Sink	Upland												
1386	8/23/2011	Outside Spigot	Upland												
1387	8/23/2011	Barn Outside Spigot	Upland												
1388	8/23/2011	Outside Spigot	Upland												
1389	3/4/2010	Post Office - Bathroom Sink	Upland	Yes				2.42		< 1	255		< 2	< 1	
1390	8/23/2011	Outside Spigot	Valley												
1391	8/23/2011	Outside Spigot	Upland												
1392	8/24/2011	Kitchen Sink	Upland												
1393	8/24/2011	Kitchen Sink	Upland												
1394	8/24/2011	Kitchen Sink	Valley												
1395	8/24/2011	Kitchen Sink	Upland												
1396	8/24/2011	Kitchen Sink	Valley												
1397	8/24/2011	Inside Faucet	Upland												
1398	8/24/2011	Kitchen Sink	Upland												
1399	8/24/2011	Kitchen Sink	Valley												
1400	3/8/2010	Basement At Pressure Tank	Upland					0.057		< 1	125		< 2	3	
1401	8/24/2011	Kitchen Sink	Upland												
1402	8/24/2011	Kitchen Sink	Upland												
1403	8/24/2011	Kitchen Sink	Upland												
1404	8/24/2011	Bathroom Sink	Valley												
1405	8/24/2011	Kitchen Sink	Valley												
1406	8/25/2011	Kitchen Sink	Valley												

Table S.8. Groundwater Quality Data for 1701 "Pre-Drill" Water Well Samples from Susquehanna County, Pennsylvania

Water Well ID	Sample Date	Sampling Port	Topo-graphic Location	Gas Extraction Area (Within 1 km of Active Gas Well)	Selenium (mg/L)	Silver (mg/L)	Sodium (mg/L)	Strontium (mg/L)	Sulfate (mg/L)	Sulfide (mg/L)	TDS (mg/L)	Thallium (mg/L)	TSS (mg/L)	Turbidity (NTU)	Vanadium (mg/L)
1407	8/25/2011	Kitchen Sink	Valley												
1408	8/25/2011	Kitchen Sink	Valley												
1409	8/25/2011	Downstairs Sink	Valley												
1410	8/25/2011	Kitchen Sink	Upland												
1411	8/25/2011	Kitchen Sink	Valley												
1412	8/25/2011	Kitchen Sink	Valley												
1413	8/26/2011	Kitchen Sink	Upland												
1414	8/26/2011	Outside Spigot	Valley												
1415	8/26/2011	Kitchen Sink	Upland												
1416	8/26/2011	Kitchen Sink	Valley												
1417	8/26/2011	Kitchen Sink	Upland												
1418	8/26/2011	Kitchen Sink	Upland												
1419	8/26/2011	Barn Sink	Valley												
1420	8/27/2011	Kitchen Sink	Upland												
1421	8/27/2011	Kitchen Sink	Upland												
1422	10/30/2009	Basement At Pressure Tank	Upland	Yes				0.068		< 1	100		< 2		
1423	8/31/2011	Kitchen Sink	Upland												
1424	8/31/2011	Kitchen Sink	Valley												
1425	8/31/2011	Spigot On Well	Valley												
1426	8/31/2011	Kitchen Sink	Upland												
1427	9/1/2011	Kitchen Sink	Valley												
1428	9/1/2011	Spigot Side Of House	Valley												
1429	9/2/2011	Kitchen Sink	Upland												
1430	9/2/2011	Spigot On Well	Valley												
1431	3/7/2010	Kitchen Sink	Valley					< .025		< 1	285		< 2	< 1	

Table S.8. Groundwater Quality Data for 1701 "Pre-Drill" Water Well Samples from Susquehanna County, Pennsylvania

Water Well ID	Sample Date	Sampling Port	Topo-graphic Location	Gas Extraction Area (Within 1 km of Active Gas Well)	Selenium (mg/L)	Silver (mg/L)	Sodium (mg/L)	Strontium (mg/L)	Sulfate (mg/L)	Sulfide (mg/L)	TDS (mg/L)	Thallium (mg/L)	TSS (mg/L)	Turbidity (NTU)	Vanadium (mg/L)
1432	9/2/2011	Kitchen Sink	Upland												
1433	9/2/2011	Kitchen Sink	Valley												
1434	9/2/2011	Bathroom Sink	Upland												
1435	9/2/2011	Kitchen Sink	Upland												
1436	9/2/2011	Well Spigot	Upland												
1437	3/8/2010	Basement At Pressure Tank	Upland					0.096		< 1	230		< 2	< 1	
1438	9/2/2011	Kitchen Sink	Valley												
1439	9/7/2011	Kitchen Sink	Upland												
1440	9/7/2011	Kitchen Sink	Upland												
1441	9/7/2011	Kitchen Sink	Valley												
1442	9/7/2011	Kitchen Sink	Upland												
1443	9/8/2011	Kitchen Faucet	Valley												
1444	9/8/2011	Kitchen Sink	Valley												
1445	9/8/2011	Outside Spigot	Upland												
1446	9/8/2011	Kitchen Sink	Valley												
1447	9/8/2011	Kitchen Sink	Upland												
1448	9/8/2011	Kitchen Sink	Upland												
1449	9/8/2011	Kitchen Sink	Upland												
1450	3/8/2010	Basement Pressure Tank	Upland					1.18		< 1	160		< 2	< 1	
1451	8/20/2009		Upland	Yes				1.64		< 1	180		< 2		
1452	3/7/2010	Pressure Tank	Upland					0.347		< 1	125		< 2	< 1	
1453	3/7/2010	Kitchen Sink	Upland					0.194		< 1	164		2.8	< 1	
1454	3/11/2010	Pressure Tank	Valley					0.31		< 1	200		< 2	< 1	
1455	5/1/2010	Kitchen Sink	Valley					0.608		< 1	140	< .002	< 2		< .01
1456	5/1/2010	Kitchen Sink	Valley					< .025		< 1	48	< .002	< 2		< .01

Table S.8. Groundwater Quality Data for 1701 "Pre-Drill" Water Well Samples from Susquehanna County, Pennsylvania

Water Well ID	Sample Date	Sampling Port	Topo-graphic Location	Gas Extraction Area (Within 1 km of Active Gas Well)	Selenium (mg/L)	Silver (mg/L)	Sodium (mg/L)	Strontium (mg/L)	Sulfate (mg/L)	Sulfide (mg/L)	TDS (mg/L)	Thallium (mg/L)	TSS (mg/L)	Turbidity (NTU)	Vanadium (mg/L)
1457	5/3/2010	Outside Spigot On Garage	Upland	Yes				< .025		< 1	42	< .002	2.4		< .01
1458	5/6/2010	Kitchen Sink	Upland					0.05		< 1	105	< .002	< 2		< .01
1459	5/6/2010	Kitchen Sink	Valley	Yes				0.907		6	350	< .002	6.7		< .01
1460	5/7/2010	Kitchen Sink	Valley	Yes				1.04		1	140	< .002	< 2		< .01
1461	8/24/2011	Kitchen Sink	Valley		< .005	< .001	6.2	0.18	9.8	< 3	110		< 4	1.5	
1462	8/24/2011	Inside Faucet	Valley		< .005	< .001	11	0.32	9.4	< 3	140		< 4	< .85	
1463	8/24/2011	Pressure Tank Hose Bib	Upland		< .005	< .001	11	0.24	7	< 3	100		< 4	< .85	
1464	5/7/2010	Outside Spigot	Upland					0.212		< 1	65	< .002	< 2		< .01
1465	8/30/2011	Inside Faucet	Valley		< .005	< .001	25	0.31	12	< 3	190		< 4	4.5	
1466	5/13/2010	Kitchen Sink	Valley					0.139		< 1	95	< .002	< 2		< .01
1467	8/30/2011	Pressure Tank	Valley		< .005	< .001	18	0.34	8.9	< 3	140		< 4	2.9	
1468	5/13/2010	Kitchen Sink	Upland					0.289		< 1	125	< .002	< 2		< .01
1469	5/13/2010	Outside Spigot	Upland					0.102		< 1	155	< .002	< 2		< .01
1470	8/30/2011	Inside Faucet	Upland		< .005	< .001	12	0.62	12	< 3	110		< 4	< .85	
1471	7/7/2009	Kitchen Sink	Upland					0.227		< 1	52		< 2		
1472	5/20/2010	Kitchen Sink	Upland					0.778		< 1		< .002			< .01
1473	5/20/2010	Basement At Pressure Tank	Upland					0.137				< .002			< .01
1474	5/20/2010	Pressure Tank- Quarry Top Well	Upland							< 1					
1475	5/20/2010	Pressure Tank At Trailer Park	Upland					0.725		< 1		< .002			0.01
1476	5/20/2010	Quarry Sulfur Well	Upland					0.724		< 1		< .002			< .01
1477	5/20/2010	Spigot In Garage	Upland					0.07		< 1		< .002			< .01
1478	3/16/2010	Outside Spigot	Upland					0.073		< 1	104		< 2	< 1	

Table S.8. Groundwater Quality Data for 1701 "Pre-Drill" Water Well Samples from Susquehanna County, Pennsylvania

Water Well ID	Sample Date	Sampling Port	Topo-graphic Location	Gas Extraction Area (Within 1 km of Active Gas Well)	Selenium (mg/L)	Silver (mg/L)	Sodium (mg/L)	Strontium (mg/L)	Sulfate (mg/L)	Sulfide (mg/L)	TDS (mg/L)	Thallium (mg/L)	TSS (mg/L)	Turbidity (NTU)	Vanadium (mg/L)
1479	4/19/2009	Kitchen Sink	Upland					0.122		< 1	135		< 2		
1480	3/18/2010	Kitchen Sink	Upland					0.025		< 1	64	< .002	< 2		< .01
1481	3/18/2010	Pressure Tank	Upland	Yes				0.047		< 1	104	< .002	2		< .01
1482	3/22/2010	Kitchen Sink	Valley	Yes				0.072		1	80	< .002	6		< .01
1483	1/13/2011		Valley	Yes	< .002	< .001	13	0.65	< 5	< 1	132		< 2	< 1	
1484	3/25/2010	Basement At Pressure Tank	Upland					0.232		< 1	148	< .002	< 2		< .01
1485	3/25/2010	Hand Dug Well	Valley					< .025		< 1	40	< .002	< 2		< .01
1486	6/14/2011	Directly From Pressure Tank Cwt In Sw Corner Of Ba	Upland	Yes	< .005	< .001	3.5	0.11	8.8	< 3	92		< 4	25	
1487	6/14/2011	Kitchen Sink	Upland		< .005	< .001	13	0.41	17	< 3	170		< 4	< .85	
1488	6/14/2011	Hose Bib	Upland		< .005	< .001	4.2	0.043	37	< 3	160		< 4	1.3	
1489	3/25/2010	Spring At Overflow Pipe	Valley					< .026		< 1	64	< .002	< 2		< .01
1490	6/14/2011	Outside Hose Bib On Sw End Of Home	Valley		< .005	< .001	11	0.85	11	< 3	180		< 4	2.2	
1491	3/28/2010	Basement At Pressure Tank	Upland					0.859		4	224	< .002	9.2		< .01
1492	1/13/2011		Upland	Yes	<0.0020	<0.0010	9.6	0.36	10	<1	192		<2	<1	
1493	6/16/2011	Wash Tub In Garage	Upland		< .005	< .001	12	0.51	16	< 3	160		< 4	< .85	
1494	6/16/2011	Kitchen Sink	Valley		< .005	< .001	32	0.07	13	< 3	170		< 4	< .85	
1495	6/27/2011	Bathroom Sink Body Shop	Valley	Yes	< .005	< .001	9.5	0.48	8.8	< 3	120		< 4	< .85	
1496	6/28/2011	Garage Sink	Valley		< .005	< .001	5.4	0.12	12	< 3	130		< 4	< .85	
1497	6/28/2011	Kitchen Sink	Upland		< .005	< .001	3	0.15	16	< 3	160		< 4	< .85	

Table S.8. Groundwater Quality Data for 1701 "Pre-Drill" Water Well Samples from Susquehanna County, Pennsylvania

Water Well ID	Sample Date	Sampling Port	Topo-graphic Location	Gas Extraction Area (Within 1 km of Active Gas Well)	Selenium (mg/L)	Silver (mg/L)	Sodium (mg/L)	Strontium (mg/L)	Sulfate (mg/L)	Sulfide (mg/L)	TDS (mg/L)	Thallium (mg/L)	TSS (mg/L)	Turbidity (NTU)	Vanadium (mg/L)
1498	3/18/2010	Kitchen Sink	Upland					0.826		< 1	188	< .002	< 2		< .01
1499	5/21/2010	Kitchen Sink	Upland	Yes				0.445				< .002			< .01
1500	5/21/2010	Hand Dug #1	Upland	Yes				< .025				< .002			< .01
1501	7/6/2011	Kitchen Sink	Upland		< .005	< .001	5	0.37	15	< 3	120		< 4	< .85	
1502	7/6/2011	Kitchen Sink	Upland		< .005	< .001	6.6	0.51	6.5	< 3	80		< 4	< .85	
1503	5/21/2010	Hand Dug Well #2	Upland	Yes				< .025				< .002			< .01
1504	7/6/2011	Kitchen Sink	Valley		< .005	< .001	5	0.12	4	< 3	88		< 4	< .85	
1505	7/6/2011	Kitchen Sink	Upland		< .005	< .001	6.2	0.58	13	< 3	150		< 4	8.3	
1506	7/7/2011	Pressure Tank Tap	Valley		< .005	< .001	5.3	0.048	14	< 3	110		< 4	1.1	
1507	5/21/2010		Valley					0.805				< .002			< .01
1508	7/14/2011	Outdoor Spigot	Upland		< .005	< .001	7.7	0.53	12	< 3	160		< 4	< .85	
1509	7/16/2011	Pressure Tank	Valley		< .005	< .001	17	0.33	8.5	< 3	130		< 4	17	
1510	5/22/2010	Spare Well	Upland					0.041				< .002			< .01
1511	5/22/2010	Kitchen Sink	Upland					0.095				< .002			< .01
1512	7/18/2011	Outside Hose Bib	Upland		< .005	< .001	7	0.17	11	< 3	130		16	21	
1513	7/18/2011	Hydrant Northwest Of Well	Upland		< .005	< .001	23	1.6	13	< 3	200		< 4	< .85	
1514	7/18/2011		Valley	Yes	< .005	< .001	5.5	0.055	16	< 3	110		< 4	< .85	
1515	5/16/2011	Pressure Tank	Upland		<0.005	<0.001	5.5	0.047	12	<3	110		<4	<0.85	
1516	5/16/2011	Slop Sink In Barn	Upland		< .005	< .001	4.7	0.042	12	< 3	96		< 4	2	
1517	5/16/2011	Pressure Tank	Upland		< .005	< .001	12	0.27	9.6	< 3	150		< 4	2.2	
1518	5/16/2011	Outdoor Spigot	Upland		< .005	< .001	1.5	0.034	7.6	< 3	60		< 4	4.6	
1519	7/25/2011	Kitchen Sink Cold Water Tap	Upland		< .005	< .001	8.5	0.17	14	< 3	160		< 4	1.3	
1520	5/17/2011	Outside Spigot	Upland		< .005	< .001	6.7	0.32	10	< 3	130		< 4	< .85	
1521	5/17/2011	Kitchen Sink	Valley		< .005	< .001	27	0.69	5.6	< 3	150		< 4	< .85	

Table S.8. Groundwater Quality Data for 1701 "Pre-Drill" Water Well Samples from Susquehanna County, Pennsylvania

Water Well ID	Sample Date	Sampling Port	Topo-graphic Location	Gas Extraction Area (Within 1 km of Active Gas Well)	Selenium (mg/L)	Silver (mg/L)	Sodium (mg/L)	Strontium (mg/L)	Sulfate (mg/L)	Sulfide (mg/L)	TDS (mg/L)	Thallium (mg/L)	TSS (mg/L)	Turbidity (NTU)	Vanadium (mg/L)
1522	5/17/2011	Kitchen Sink	Valley		< .005	< .001	20	0.48	5.5	< 3	130		< 4	< .85	
1523	5/17/2011	Outdoor Spigot	Upland		< .005	< .001	5.3	0.36	9	< 3	< 10		< 4	< .85	
1524	5/17/2011	Garage Pressure Tank	Upland		< .005	< .001	5.7	0.084	7.8	< 3	110		< 4	1.4	
1525	3/31/2010	Spigot On Porch	Valley					< .025		< 1	200	< .002	2.8		< .01
1526	5/17/2011	Pressure Tank Spigot	Upland		< .005	< .001	4.2	0.17	9.9	< 3	110		< 4	< .85	
1527	3/31/2010	Hydrant At Well Head	Upland					0.348		< 1	128	< .002	< 2		< .01
1528	8/4/2011	Inside Faucet	Valley		< .005	< .001	11	0.51	14	< 3	150		< 4	< .85	
1529	8/9/2011	Outside Faucet	Upland		<0.005	<0.001	9.6	0.3	12	<3	120		<4	<0.85	
1530	3/28/2010	Prssure Tank At Fitzsimon'S House	Upland					0.026		< 1	76	< .002	< 2		< .01
1531	5/18/2011	Kitchen Sink	Upland		<0.005	<0.001	5.5	0.081	9.4	<3	130		<4	<0.85	
1532	5/18/2011	Kitchen Sink	Upland		< .005	< .001	36	0.21	7.6	< 3	130		< 4	< .85	
1533	5/18/2011	Kitchen Sink	Upland		< .005	< .001	3.6	0.036	14	< 3	62		< 4	1.1	
1534	5/18/2011	Kitchen Sink	Upland		<0.005	<0.001	13	0.19	9.8	<3	100		<4	<0.85	
1535	3/28/2010	Pressure Tank In Furnace Room	Upland					0.074		< 1	< 2	< .002	112		< .01
1536	5/19/2011	Bathroom Sink Body Shop	Valley	Yes	< .005	< .001	9	0.38	8.9	< 3	130		< 4	0.97	
1537	4/1/2010	Pressure Tank	Upland					0.1		< 1	160	< .002	< 2		< .01
1538	5/24/2011	Basement Pressure Tank	Valley		< .005	< .001	96	0.21	1.8	< 3	230		< 4	< .85	
1539	5/24/2011	Kitchen Sink	Upland		< .005	< .001	13	0.79	10	< 3	150		< 4	< .85	
1540	4/5/2010	Kitchen Sink	Upland					0.035		< 1	100	< .002	7.2		< .01
1541	4/5/2010	Kitchen Sink Father'S	Upland					0.624		1	148	< .002	3.2		< .01

Table S.8. Groundwater Quality Data for 1701 "Pre-Drill" Water Well Samples from Susquehanna County, Pennsylvania

Water Well ID	Sample Date	Sampling Port	Topo-graphic Location	Gas Extraction Area (Within 1 km of Active Gas Well)	Selenium (mg/L)	Silver (mg/L)	Sodium (mg/L)	Strontium (mg/L)	Sulfate (mg/L)	Sulfide (mg/L)	TDS (mg/L)	Thallium (mg/L)	TSS (mg/L)	Turbidity (NTU)	Vanadium (mg/L)
1542	5/24/2011	Pressure Tank	Valley	Yes	< .005	< .001	15	0.14	15	< 3	180		4	5	
1543	4/1/2010	Kitchen Sink	Upland					0.087		< 1	124	< .002	< 2		< .01
1544	4/1/2010	Kitchen Sink	Upland					0.135		< 1	140	< .002	< 2		< .01
1545	6/1/2011	Kitchen Sink	Upland		< .005	< .001	15	1.1	9.8	< 3	160		< 4	< .85	
1546	4/1/2010	Kitchen Sink	Upland					0.355		< 1	168	< .002	< 2		< .01
1547	6/2/2011	Township Bldg Garage Slop Sink	Valley		< .005	< .001	31	1.4	12	< 3	190		< 4	< .85	
1548	6/3/2011	Outside Spigot	Valley		< .005	< .001	65	1	9.2	< 3	190		< 4	< .85	
1549	6/3/2011	Pressure Tank	Valley	Yes	<0.005	<0.001	13	0.47	15	<3	170		<4	<0.85	
1550	6/9/2011	Outside Spigot	Valley	Yes	< .005	< .001	8.8	0.19	13	< 3	150		< 4	< .85	
1551	6/9/2011	Kitchen Sink	Upland		< .005	< .001	13	0.71	11	< 3	200		< 4	< .85	
1552	6/10/2011	Pressure Tank (Basement Back Left Closet)	Valley	Yes	< .005	< .001	7.6	0.57	16	< 3	170		< 4	< .85	
1553	6/10/2011		Valley		<0.005	<0.001	24	0.64	19	<3	210		<4	<0.85	
1554	3/28/2010	Outside Spigot	Upland					< .025		15	196	< .002	48.4		< .01
1555	4/9/2010	Basement Pressure Tank	Upland					0.084		< 1	65	< .002	2		< .01
1556	4/9/2010	Kitchen Sink	Valley	Yes				0.055		3	55	< .002	< 2		< .01
1557	4/9/2010	Basement Pressure Tank	Valley					0.4		1	200	< .002	2		< .01
1558	5/27/2010	Garage Sink	Valley					0.008				< .002			< .01
1559	5/27/2010		Upland					0.112				< .002			< .01
1560	5/27/2010	Outside Spigot	Upland					0.124				< .002			< .01
1561	6/3/2010	Main House Kitchen Sink	Upland												
1562	6/4/2010	Outside Hydrant	Upland												
1563	6/4/2010	Kitchen Sink	Upland	Yes											

Table S.8. Groundwater Quality Data for 1701 "Pre-Drill" Water Well Samples from Susquehanna County, Pennsylvania

Water Well ID	Sample Date	Sampling Port	Topo-graphic Location	Gas Extraction Area (Within 1 km of Active Gas Well)	Selenium (mg/L)	Silver (mg/L)	Sodium (mg/L)	Strontium (mg/L)	Sulfate (mg/L)	Sulfide (mg/L)	TDS (mg/L)	Thallium (mg/L)	TSS (mg/L)	Turbidity (NTU)	Vanadium (mg/L)
1564	6/4/2010	Kitchen Sink	Upland												
1565	6/4/2010	Kitchen Sink	Valley												
1566	6/13/2010	Kitchen Sink	Valley							< 1					
1567	6/13/2010	Hydrant On Well	Valley							< 1					
1568	6/13/2010	Kitchen Sink	Valley							< 1					
1569	6/13/2010	Kitchen Sink	Upland							< 1					
1570	6/13/2010		Valley							< 1					
1571	6/13/2010	Kitchen Sink	Valley							< 1					
1572	6/12/2010	Pressure Tank	Upland							<1					
1573	6/7/2010	Outside Spigot	Upland												
1574	6/7/2010	Spring Pipe	Upland							< 1					
1575	6/7/2010	Kitchen Sink	Upland							< 1					
1576	6/10/2010	Pressure Tank	Upland					0.13		< 1	110	< .002	< 2		< .01
1577	6/10/2010	Kitchen Sink	Upland							< 1					
1578	6/10/2010	Pressure Tank	Upland							< 1					
1579	6/10/2010	Kitchen Sink	Upland							< 1					
1580	6/10/2010	Kitchen Sink	Valley							< 1					
1581	6/10/2010		Upland							< 1					
1582	6/10/2010	Kitchen Sink	Upland							< 1					
1583	3/25/2009		Valley	Yes				<0.025		<1	53		<2		
1584	6/10/2010	Kitchen Sink	Upland							< 1					
1585	6/12/2010	Pressure Tank	Upland							< 1					
1586	6/12/2010	Kitchen Faucet	Upland												
1587	6/12/2010	Bathroom Faucet	Valley												
1588	6/14/2010	At Pressure Tank	Valley							< 1					
1589	6/14/2010	Kitchen Sink	Upland							< 1					
1590	4/4/2009		Valley					0.036		< 1	48		< 2		
1591	6/2/2011	Dug Well	Upland	Yes	<0.005	<0.001	2.7	0.034	9.9	<3	59		<4	1.1	

Table S.8. Groundwater Quality Data for 1701 "Pre-Drill" Water Well Samples from Susquehanna County, Pennsylvania

Water Well ID	Sample Date	Sampling Port	Topo-graphic Location	Gas Extraction Area (Within 1 km of Active Gas Well)	Selenium (mg/L)	Silver (mg/L)	Sodium (mg/L)	Strontium (mg/L)	Sulfate (mg/L)	Sulfide (mg/L)	TDS (mg/L)	Thallium (mg/L)	TSS (mg/L)	Turbidity (NTU)	Vanadium (mg/L)
1592	3/4/2009		Valley					0.181		< 1	120		< 2		
1593	4/19/2009		Upland					0.295		< 1	140		< 2		
1594	9/1/2009		Upland	Yes				0.298		< 1	128		< 2		
1595	7/19/2011		Upland												
1596	2/20/2009	Barn Sink	Valley					1.22		<1	147		<2.0		
1597	2/26/2009	Barn Sink	Upland							<1	130		<2		
1598	11/9/2009	Pressure Tank	Valley	Yes				0.358		< 1	80		< 2		
1599	12/6/2009	Kitchen Faucet	Upland					0.058		< 1	56		< 2	< 1	
1600	12/6/2009	Kitchen Faucet	Valley	Yes				0.035		< 1	60		< 2	< 1	
1601	12/6/2009	Pressure Tank	Valley	Yes				0.043		< 1	92		< 2	< 1	
1602	12/6/2009	Kitchen Faucet	Upland					0.178		< 1	172		< 2	< 1	
1603	12/15/2009	Kitchen Sink	Upland					0.131		<1	72		<2	<1	
1604	12/17/2009	Pressure Tank	Upland					0.072		< 1	12		< 2	< 1	
1605	1/11/2010	Pressure Tank	Upland					0.083		< 1	84		< 2	< 1	
1606	1/11/2010	Kitchen Faucet	Upland					0.072		< 1	120		< 2	< 1	
1607	1/11/2010	Kitchen Sink	Upland					0.058		<1	40		<2	3	
1608	1/11/2010	Pressure Tank	Upland					0.052		< 1	20		< 2	< 1	
1609	1/11/2010	Pressure Tank	Upland					0.387		< 1	164		< 2	< 1	
1610	1/11/2010	Pressure Tank	Upland					0.251		< 1	116		< 2	< 1	
1611	1/12/2010	Spigot Into House	Upland	Yes				0.252		<1	140		25.2	4	
1612	1/16/2010	Pressure Tank	Upland					1.06		< 1	172		< 2	< 1	
1613	1/21/2010	Kitchen Sink	Upland	Yes				0.478		<1	136		<2	<1	
1614	2/4/2010	Pressure Tank	Valley					0.169		<1	108		2.4	2	
1615	2/4/2010	Kitchen Sink	Upland					0.116		<1	48		<2	<1	
1616	2/6/2010	Kitchen Sink	Valley					0.176		<1	136		<2	<1	
1617	2/15/2010	Kitchen Sink	Valley					0.673		<1	180		<2	<1	
1618	2/15/2010	Kitchen Sink	Valley					0.656		<1	188		<2	<1	
1619	2/18/2010	Bathroom Sink	Valley	Yes				0.176		<5	168		68.8	15	

Table S.8. Groundwater Quality Data for 1701 "Pre-Drill" Water Well Samples from Susquehanna County, Pennsylvania

Water Well ID	Sample Date	Sampling Port	Topo-graphic Location	Gas Extraction Area (Within 1 km of Active Gas Well)	Selenium (mg/L)	Silver (mg/L)	Sodium (mg/L)	Strontium (mg/L)	Sulfate (mg/L)	Sulfide (mg/L)	TDS (mg/L)	Thallium (mg/L)	TSS (mg/L)	Turbidity (NTU)	Vanadium (mg/L)
1620	2/22/2010	Kitchen Faucet	Upland					0.864		< 1	192		< 2	< 1	
1621	2/27/2010	Kitchen Sink	Valley					0.067		<1	116		<2	<1	
1622	3/4/2010	Bathroom Sink	Upland	Yes				0.091		14	225		3.6	3	
1623	3/7/2010	Pressure Tank	Upland					0.048		< 1	56		2.8	< 1	
1624	3/28/2010	Kitchen Faucet	Upland					0.455		< 1	180		< 2	< 1	
1625	4/8/2010	Outside Faucet	Upland					0.809		< 1	132	< .002	< 2		< .01
1626	4/9/2010	Kitchen Sink	Valley					0.085		1	116		<2	<1	
1627	4/10/2010	Kitchen Sink	Valley					0.212		1	110	<0.002	<2		<0.01
1628	4/10/2010	Pressure Tank	Valley					0.328		3	190	<0.002	<2		<0.010
1629	4/19/2010	Kitchen Sink	Valley					0.05		<1	153	<0.002	2.8		<0.01
1630	4/22/2010	Kitchen Sink	Valley					0.255		<1	253	<0.002	<1		<0.01
1631	4/22/2010	Kitchen Sink	Valley					0.244		1	226	<0.002	<2		<0.010
1632	4/22/2010	Stepmother' S House	Valley					0.249		<1	260	<0.002	<2		<0.01
1633	4/24/2010	Kitchen Faucet	Upland	Yes				0.462		1	213	< .002	< 2		< .01
1634	4/22/2010	Outside Faucet	Valley					0.222		2	253	< .002	< 2		< .01
1635	5/1/2010	Kitchen Faucet	Valley	Yes				0.123		< 1	215	< .002	< 2		< .01
1636	5/1/2010	Kitchen Sink	Upland					0.278		<1	178	<0.002	<2		<0.01
1637	5/21/2010	Pressure Tank	Valley					0.06		< 1	140	< .002	5.6		< .01
1638	6/3/2010	Kitchen Faucet	Upland					0.158			115	< .002	< 2		< .01
1639	6/7/2010	Outside Spigot	Upland					1.97		<1	215	<0.002	<2		<0.01
1640	6/12/2010	Spigot In Garage	Valley					0.056		<1	185	<0.002	<2		<0.016
1641	6/13/2010	Kitchen Sink	Valley					0.668		<1	<2.0	<0.002	<2.0		<0.01
1642	6/13/2010	Kitchen Sink	Valley							<1	190		<2		
1643	6/23/2010	Kitchen Faucet	Upland					0.991			135	< .002	< 2		< .01
1644	7/1/2010	Kitchen Faucet	Valley					0.211		< 1	125	< .002	< 2		0.01

Table S.8. Groundwater Quality Data for 1701 "Pre-Drill" Water Well Samples from Susquehanna County, Pennsylvania

Water Well ID	Sample Date	Sampling Port	Topo-graphic Location	Gas Extraction Area (Within 1 km of Active Gas Well)	Selenium (mg/L)	Silver (mg/L)	Sodium (mg/L)	Strontium (mg/L)	Sulfate (mg/L)	Sulfide (mg/L)	TDS (mg/L)	Thallium (mg/L)	TSS (mg/L)	Turbidity (NTU)	Vanadium (mg/L)
1645	7/2/2010	Kitchen Sink	Valley												
1646	7/6/2010	Kitchen Faucet	Valley					0.188		< 1	110	< .002	< 2		< .01
1647	7/6/2010	Kitchen Sink	Upland					1.16		<1	140	<0.002	<2		<0.010
1648	7/6/2010	Pressure Tank	Upland					0.193		<1	200	<0.002	<2		<0.01
1649	7/7/2010	Kitchen Sink	Upland					0.392		<1	175	<0.002	<2		<0.01
1650	7/7/2010	Kitchen Faucet	Valley					0.49		< 1	195	< .002	< .2		< .01
1651	7/7/2010	Pressure Tank	Valley							<1	180		<2		
1652	7/8/2010	Kitchen Sink	Upland					0.315		<1	145	<0.002	<2		<0.01
1653	7/9/2010	Pressure Tank	Valley					0.531		7	115	< .002	< 2		< .01
1654	7/2/2010	Kitchen Faucet	Valley					0.322		< 1	100	< .002	< 2		< .01
1655	7/9/2010	Outside Spigot	Valley					0.738		<1	135	<0.002	<2		<0.010
1656	7/9/2010	Kitchen Sink	Valley					0.376		<1	150	<0.002	<2.0		<0.01
1657	7/7/2010	Kitchen Faucet	Upland	Yes				0.009		< 1	100	< .002	< 2		< .01
1658	7/7/2010	Kitchen Sink	Upland					0.242		<1	205	<0.002	<2		<0.010
1659	7/8/2010	Kitchen Faucet	Valley					0.301		< 1	105	< .002	< 2		< .01
1660	7/8/2010	Kitchen Sink	Upland					0.049		<1	115	<0.002	<2.0		<0.010
1661	7/10/2010	Pressure Tank	Upland					0.144		< 1	105	< .002	< 2		< .01
1662	7/10/2010	Basement Spigot Pre-Filter	Valley					0.184		<1	140	<0.002	4.4		<0.01
1663	7/10/2010	Kitchen Faucet	Upland					< .025		< 1	65	< .002	4		< .01
1664	7/10/2010	Pressure Tank	Upland					0.252		<1	110	<0.002	<2		<0.01
1665	7/11/2010	Kitchen Faucet	Upland					0.706		< 1	130	< .002	< 2		< .01
1666	7/12/2010	Kitchen Faucet	Valley					0.738		6	140	< .002	< 2		< .01
1667	7/13/2010	Basement (Pre-Filter)	Valley	Yes				0.154		< 1	110	< .002	< 2		< .01
1668	7/13/2010	Kitchen Sink	Valley					0.068		<1	55	<0.002	<2		<0.01
1669	7/13/2010	Deep Well	Valley					0.712		< 5		< .002			< .01

Table S.8. Groundwater Quality Data for 1701 "Pre-Drill" Water Well Samples from Susquehanna County, Pennsylvania

Water Well ID	Sample Date	Sampling Port	Topo-graphic Location	Gas Extraction Area (Within 1 km of Active Gas Well)	Selenium (mg/L)	Silver (mg/L)	Sodium (mg/L)	Strontium (mg/L)	Sulfate (mg/L)	Sulfide (mg/L)	TDS (mg/L)	Thallium (mg/L)	TSS (mg/L)	Turbidity (NTU)	Vanadium (mg/L)
1670	7/28/2010	Kitchen Faucet	Valley					0.278		< 1	136	< .002	< 2		< .01
1671	7/28/2010	Milkhouse Sink	Upland	Yes				0.119		<1	140	<0.002	2		<0.01
1672	8/7/2010	Kitchen Sink	Upland					0.091		<1	185	<0.002	<2		<0.01
1673	8/8/2010	Kitchen Sink	Upland					0.043		<1	105	<0.002	<2		<0.01
1674	8/13/2010	Outside Hydrant	Valley					0.124		< 1	195	< .002	4		< .01
1675	8/19/2010	Kitchen Faucet	Valley	Yes					17	< 1	135		< 2	< 1	
1676	9/23/2010	Outside Spigot	Upland		<0.005	<0.005	19.8	1.95	<5	<1	152		<2	<1	
1677	10/10/2010		Valley	Yes	<0.005	<0.005	16.1	0.053	13	<1	90		<2	<1	
1678	10/18/2010	Kitchen Sink	Valley	Yes	<0.005	<0.005	11.8	0.219	20	<1	180		<2	1	
1679	10/18/2010	Kitchen Faucet	Upland	Yes					14	< 1	190			< 1	
1680	10/19/2010	Kitchen Faucet	Valley	Yes					13	< 1	150		< 2	< 1	
1681	10/26/2010	Spring House	Valley	Yes	<0.005	<0.005	2.75	0.04	12	<1	60		<2	<1	
1682	10/29/2010	Kitchen Faucet	Upland		<0.005	<0.005	15.1	0.138	14	<1	180		<2	<1	
1683	10/29/2010	Kitchen Faucet	Upland		<0.005	<0.005	13.6	0.168	23	<1	280		<2	<1	
1684	11/11/2010	Kitchen Faucet	Valley	Yes					28	< 1	240		< 2	1	
1685	11/11/2010	Kitchen Faucet	Upland						8	< 1	140		< 2	< 1	
1686	11/11/2010	Kitchen Faucet	Upland	Yes	<0.002	<0.001	12.6	0.39	18	<1	173		<2	<1	
1687	12/2/2010	Kitchen Sink	Valley		<0.005	<0.005	12.3	0.172	18	<1	140		<2	<1	
1688	12/4/2010	Kitchen Sink	Valley		<0.005	<0.005	1.48	0.034	8	<1	60		<2	1	
1689	12/5/2010	Kitchen Faucet	Valley	Yes					14	< 5	153		11	30	
1690	12/9/2010	Kitchen Faucet	Upland						9	< 1	87		< 2	< 1	
1691	12/11/2010	Kitchen Sink	Valley						7	<1	60		3.2	1	
1692	12/14/2010	Kitchen Sink	Upland	Yes	<0.002	<0.001	3.8	94	10	<1	80		<2	<1	
1693	12/14/2010	Kitchen Faucet	Upland	Yes	<0.002	<0.001	21	0.41	17	<1	213		2.4	<1	
1694	12/18/2010	Kitchen Faucet	Valley	Yes					26	< 1	213		< 2	1	

Table S.8. Groundwater Quality Data for 1701 "Pre-Drill" Water Well Samples from Susquehanna County, Pennsylvania

Water Well ID	Sample Date	Sampling Port	Topo-graphic Location	Gas Extraction Area (Within 1 km of Active Gas Well)	Selenium (mg/L)	Silver (mg/L)	Sodium (mg/L)	Strontium (mg/L)	Sulfate (mg/L)	Sulfide (mg/L)	TDS (mg/L)	Thallium (mg/L)	TSS (mg/L)	Turbidity (NTU)	Vanadium (mg/L)
1695	10/29/2009	Pressure Tank	Upland	Yes				0.717		< 1	315		< 2		
1696	11/6/2010	Kitchen Faucet	Upland		< .002	< .001	59.9	0.013	12	< 1	124		< 2	< 1	
1697	3/19/2009		Upland	Yes				0.88		< 1	140		< 2		
1698	3/25/2009		Upland					0.093		<1	73		<2		
1699	3/12/2009		Upland					0.181		<1	140		3.2		
1700	4/5/2010	Kitchen Sink, Main House	Upland					0.261		<1	152	<0.002	2		<0.010
1701	6/2/2011		Valley		< .005	< .001	25	1.3	12	< 3	180		< 4	< .85	
